# Kinetics and Mechanism of the Enantioselective Passerini Multicomponent Reaction Catalyzed by Chiral Ionic Liquids

**DOI:** 10.1021/acsomega.5c13327

**Published:** 2026-04-16

**Authors:** Juliana Santos, Pedro P. De Castro, Saulo T. A. Passos, Virginia Camila Rufino Ferreira, Fabrício Machado, Daniel M. Araújo, Hélio F. Dos Santos, Brenno A. D. Neto, Giovanni W. Amarante

**Affiliations:** † Chemistry Department, 28113 Federal University of Juiz de Fora, Campus Martelos, Juiz de Fora, Minas Gerais 36036-900, Brazil; ‡ Pharmacy Department, Federal University of Juiz de Fora, Campus Governador Valadares, Governador Valadares, Minas Gerais 35010-180, Brazil; § Laboratory of Medicinal and Technological Chemistry, University of Brasília, Chemistry Institute (IQ-UnB), 28127 Campus Universitário Darcy Ribeiro, Brasília, Distrito Federal 70910-900, Brazil; ∥ Universidade Estadual de Goiás, Molecular Sciences Graduate Program, Anápolis, GO 75132-400, Brazil

## Abstract

Herein, we present the development of an enantioselective methodology for the classic Passerini three-component reaction, utilizing the asymmetric counteranion-directed catalysis (ACDC) effect combined with an ionic liquid catalyst to harness the ionic liquid effect. By combining both the ACDC and ionic liquid effects, the desired Passerini products were obtained in yields of up to 93% and with enantiomeric ratios of 94:6. To the best of our knowledge, this marks the first report of an asymmetric protocol for the classic Passerini reaction utilizing ACDC effects. DLS experiments provided insights into the catalyst’s effect through the formation of ionic aggregates and highlighted the importance of the solvent in achieving higher yields. NMR investigations revealed that the reaction mechanism proceeds through the formation of nitrilium and imidate species as key intermediates, followed by a Mumm rearrangement. Theoretical calculations confirmed S-isomer selectivity, with van der Waals forces stabilizing the transition state and the ionic liquid crucial to chiral catalysis. The first kinetics experiments conducted for this multicomponent reaction (MCR) provided additional support for these findings.

## Graphical abstract

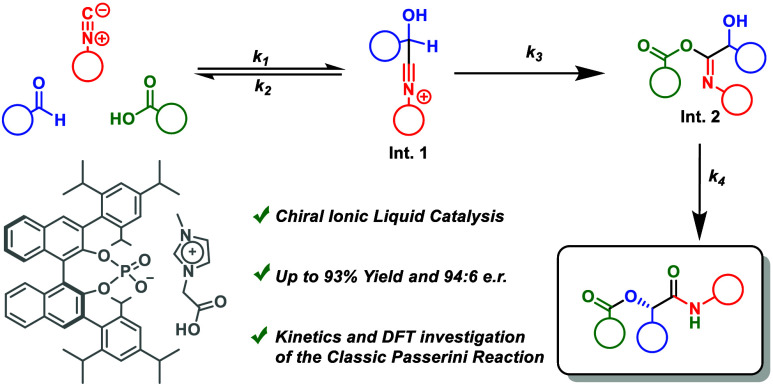

## Introduction

Over the last few decades, multicomponent reactions (MCRs) have garnered increasing interest from the synthetic organic and medicinal chemistry communities.^1^ These reactions offer an atom-economic pathway for the one-step synthesis of compounds and serve as powerful tools in diversity-oriented synthesis, high-throughput screening, and combinatorial chemistry.^2,3^ Among isocyanide-based MCRs (also known as IMCRs), the Passerini and Ugi reactions stand out as significant transformations for the preparation of peptidomimetic derivatives and have recently been applied in the total synthesis of several natural products.^4–8^ However, a major challenge in these reactions is controlling enantioselectivity,^9–11^ primarily due to the low steric demands of isocyanides.^12^

In recent times, possibly inspired by the centenary celebration of the Passerini reaction, new variations of this transformation have emerged by modifying one of its components.^13^ For instance, substituting the carboxylic acid component with a boronic acid has enabled the synthesis of α-hydroxyketones in good to excellent yields via a Passerini-type approach.^14^ Other noteworthy synthetic advancements include utilizing phthalimide derivatives as the acid component^15^ and achieving the first asymmetric protocol conducted in water.^16^

The classic Passerini reaction involves the synthesis of α-acyloxyamides through the reaction among aldehydes, isocyanides, and carboxylic acids, with the addition of an isocyanide to an aldehyde being widely accepted as the key step for achieving chirality. In the early 2000s, several groups reported examples of asymmetric Passerini-type reactions.^17–19^ The milestone came in 2003 when Dömling’s group reported the first example of an enantioselective classic Passerini three-component reaction. Their extensive catalyst screening revealed that a chiral titanium complex could produce Passerini products with up to 71:29 enantiomeric ratio (e.r.), as seen in Table 1 (entry 1).^20^ Over the following years, the use of other Lewis acids, such as copper (Table 1, entry 2)^21^ and aluminum^22^ (Table 1, entry 3) complexes, was also described and allowed the development of the first catalytic protocols and the attainment, in a few cases, of excellent enantioselectivities.

**Table 1:**
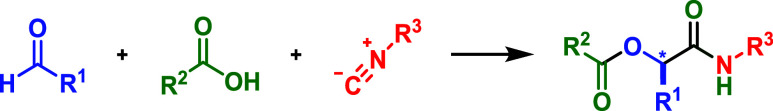

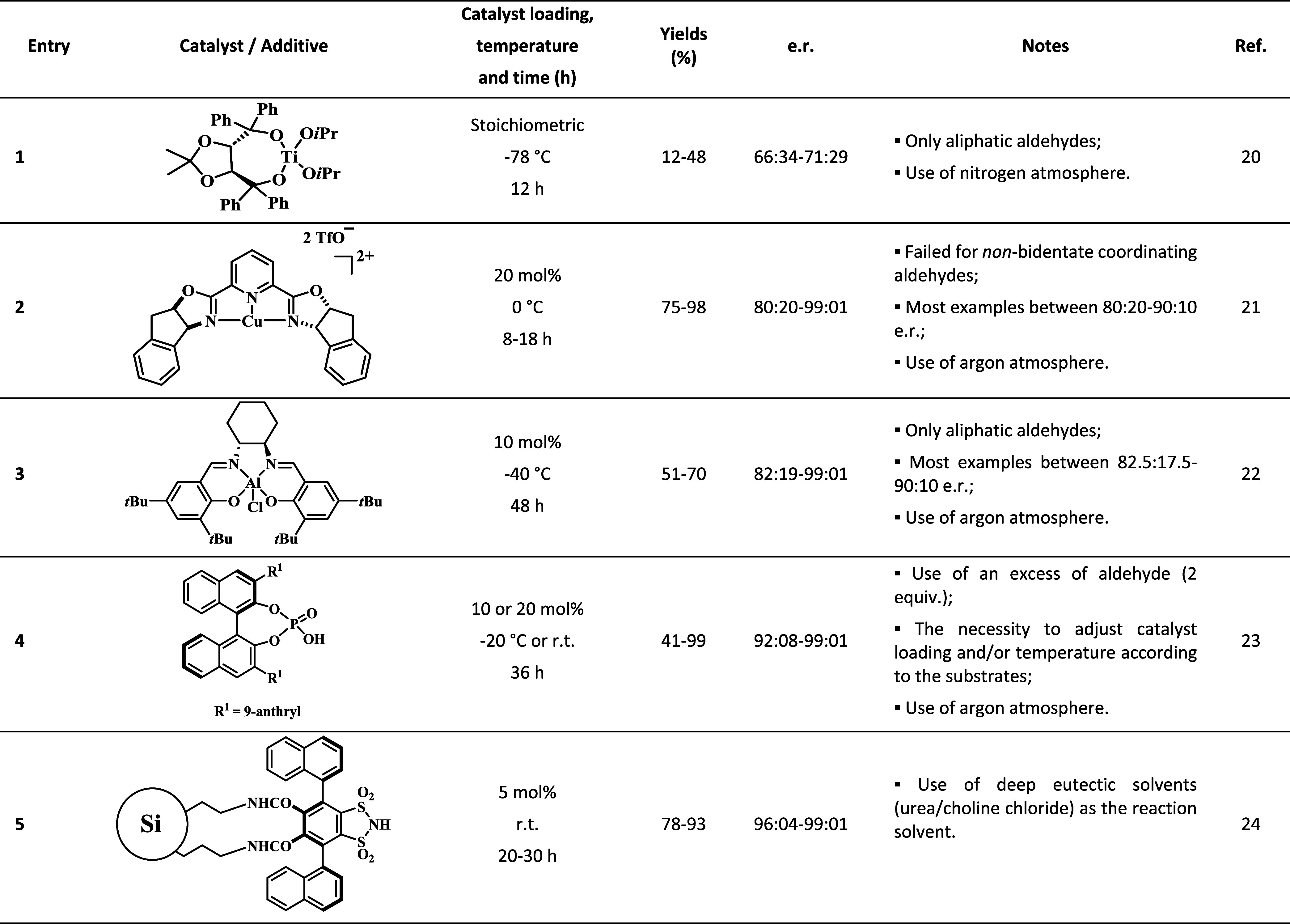
Literature Reports on the Enantioselective Classic Passerini Reaction

The first general protocol for a high enantioselective Passerini reaction was described by Tan’s group using chiral phosphoric acid catalysis (Table 1, entry 4).^23^ Although outstanding results are presented in this study, the methodology still presents some drawbacks, such as the long reaction times, the need to alter the reaction conditions according to the substrates, and the considerable decrease in e.r. when using nonsterically bulky substrates, especially for the carboxylic acid substrate. Finally, by using a chiral heterogeneous silica-supported catalyst and a deep eutectic solvent, Dughera’s group achieved α-acyloxycarboxamide adducts in high yields and e.r. (Table 1, entry 5).^24^

Despite significant advancements in the enantioselective Passerini reaction, a strong need for further development of the synthetic protocols remains. In this context, one of the most promising strategies involves the use of the unexplored asymmetric counteranion-directed catalysis (ACDC) effect. This approach relies on chiral induction through weak and noncovalent interactions between a provided chiral anion and cationic reaction intermediates.^25–27^ ACDC has proven successful in various asymmetric transformations, including the Povarov^28^ and Mannich^29^ reactions, as well as the ring opening of azetidiniums^30,31^ and diaryliodonium salts.^32,33^

Our research group has also harnessed the synergistic potential of the ACDC effect and the ionic liquid effect^34^ in devising an efficient enantioselective protocol for the Biginelli reaction.^35^ We propose that combining the ionic liquid effect with the ACDC effect-based methodology for the classic three-component Passerini reaction could yield the desired α-acyloxyamide derivatives with high yields and stereoselectivities. This strategy could, in principle, complement existing methods and overcome their limitations. In addition to the challenges described, the mechanism of the Passerini reaction (Scheme 1) has been the subject of numerous controversies.^36,37^ While only a few studies have delved into investigating the actual mechanism of the Passerini IMCR,^38^ the most compelling evidence has emerged from theoretical calculations.^39,40^ Therefore, it presents numerous opportunities for experimental approaches to further probe this intricate mechanism.

**Scheme 1:**
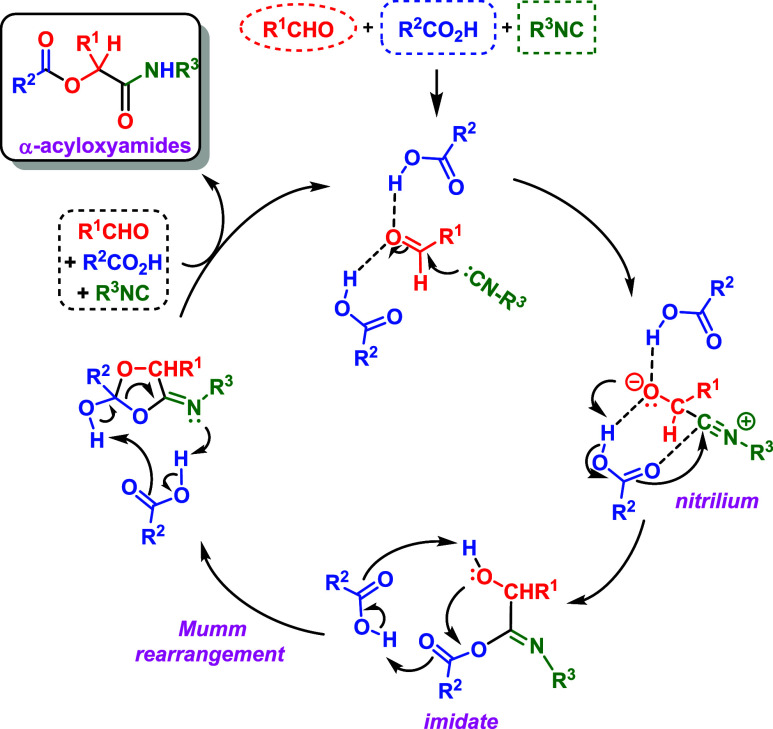
Proposed and Generally Accepted Mechanism of the Classic Multicomponent Passerini Transformation^a^

a Note that the most compelling evidence for this MCR is based on theoretical calculations.

In this study, we present our results in developing a protocol for the enantioselective Passerini MCR, leveraging the full potential of the ionic liquid effect with a chiral anion. Additionally, we present our experimental mechanistic investigation findings, supported by kinetic reaction monitoring of the multicomponent transformation and a theoretical investigation of the enantioselective step.

## Results and Discussion

### Reaction Optimization

The development of the asymmetric ACDC methodology for the Passerini reaction commenced with the preparation of distinct chiral ionic catalysts capable of combining the ACDC and ionic liquid effects. These catalysts featured various chiral phosphate derivatives as anions and substituted imidazolium as cations, employing a simple and direct methodology previously established by our research group^35^ and detailed in the Experimental Section of this work.

We initially attempted to synthesize the desired Passerini product using benzaldehyde, 4-chlorobenzoic acid, and *tert*-butyl isocyanide as substrates. Given that in nonpolar solvents, ionic species of opposite charge, such as those of the chiral catalyst, tend to remain as ion pairs or small ionic aggregates,^41^ toluene was selected as the first solvent. This could, in principle, leverage the ionic liquid effect by facilitating the formation of closely associated chiral ion pairs through a simple solvent effect. Employing catalyst 1, the desired Passerini product was isolated in 18% yield and 64:36 e.r. after 2 h at 26 °C (Table 2, entry 1). Encouragingly, extending the reaction time to 8 h significantly improved the results (Table 2, entry 2), yielding the product in 87% yield and e.r. of 90:10.

**Table 2:**
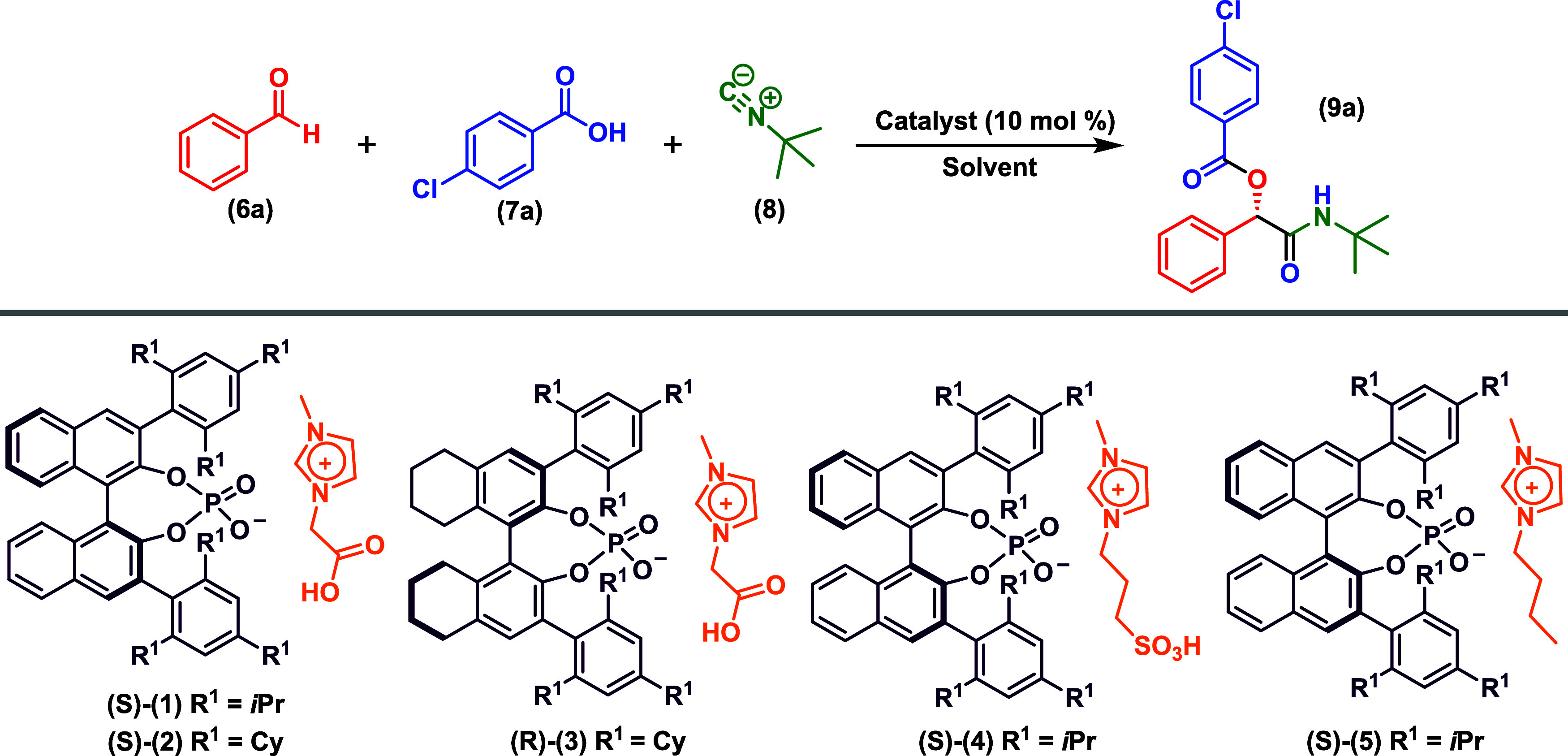
Optimization of Reaction Conditions

entry	solvent	time (h)	*T* (°C)	catalyst	yield (%)^b^	e.r.^c^
1	toluene	2	26	1	18	64:36
2	toluene	8	26	1	87	90:10
3	chloroform	8	26	1	44	54:46
4	dichloromethane	8	26	1	32	50:50
5	fluorobenzene	8	26	1	65	68:32
6	heptane	8	26	1	42	67:33
7	cyclohexane	8	26	1	75	88:12
8	pentane	8	26	1	93	94:06
9	pentane	8	26	2	90	89:11
10	pentane	8	26	3	99	17:83
11	pentane	8	26	4	66	93:07
12	pentane	8	26	5	46	76:24
13	pentane	8	0	1	58	85:15
14	pentane	8	35	1	57	86:14
15	pentane	8	26	none	10	50:50
16	pentane	8	26	*(S)*-TRIP	90	86:14

a Unless otherwise stated, reactions were conducted using benzaldehyde (0.11 mmol), 4-chlorobenzoic acid (0.10 mmol), tert-butyl isocyanide (0.10 mmol), and chiral ionic liquid catalyst (10 mol %) in 0.5 mL of solvent.

b Isolated yield.

c Determined using enantiodiscriminating HPLC.

Subsequently, we evaluated a variety of solvents under different reaction conditions (Table 2, entries 3–7). Solvents previously utilized in asymmetric Passerini protocols, such as chloroform and dichloromethane, yielded poor results (54:46 e.r. and a racemic mixture, respectively). Conversely, solvents with lower dielectric constants, such as fluorobenzene, heptane, and cyclohexane, yielded moderate to good yields and e.r. values (up to 75% yield and 88:12 e.r.). The most favorable outcome was achieved using pentane, yielding 93% product and 94:6 e.r. (Table 2, entry 8). These results showed the importance of choosing the right solvent. The right solvent strengthens the chiral anion effect and allows the formation of small ionic aggregates. These aggregates may improve the transfer of chirality from the counterion of the ionic liquid catalyst. This creates a synergistic effect between the chiral anion and the cationic Passerini intermediates, through the ionic liquid effect, which stabilizes these charged species.

Evaluation of other catalysts (Table 2, entries 9–12) showed decreased enantioselectivity, though still achieving relatively good results. Altering the reaction conditions (Table 2, entries 13 and 14) resulted in significant drops in both yield and enantiomeric ratio. Conducting the reaction without a catalyst (Table 2, entry 15) revealed a competing background reaction, yielding the racemic product in 10% yield. Finally, the commercially available phosphoric acid *(S)*-TRIP (Table 2, entry 16) provided only a moderate enantiomeric ratio (e.r. = 90% and 86:14, respectively).

A few factors likely contributed to the efficiency achieved with ionic liquid catalyst 1 under optimized conditions. First, the catalyst design effectively displays an ionic liquid effect. The chiral anion, derived from a sterically hindered phosphoric acid, is known for its ability to transfer chirality in various reaction types.^42–46^ Second, the solvent choice is essential to favor the formation of chiral aggregates based on the natural assembly process described for imidazolium ionic liquids.^47–49^ Apolar environments, as shown elsewhere,^50^ can also be advantageous for the classical Passerini reaction. This is likely because these environments can favor the reaction pathway by reducing unwanted interactions between the reactants and polar solvent molecules. Third, the presence of carboxylic acid in the catalyst’s cation structure offers two potential advantages. One benefit is that it acts as a Bronsted acid, which can be beneficial for the Passerini reaction.^1,19^ Additionally, this carboxylic acid group might also promote the subsequent fast Mumm rearrangement, as suggested by Morokuma and co-workers^40^ and detailed in Scheme 1. And finally, as a fourth hypothesis, there is the possibility of the catalyst existing in two tautomeric forms: the first one as a complex between phosphoric acid and imidazolium, and the second one as an ionic pair between phosphate anion and imidazolium cation. By considering the catalyst as a complex, there would be greater contact between the reactants with the chiral part of the catalyst, consequently providing greater asymmetric induction. Although ion pairs are typically favored in imidazolium ionic liquids,^41^ H-bond complexes are also observed,^51^ enabling a complex intramolecular network of the developed catalyst.

### Formation of Nanoaggregates of Catalyst 1

To better support our proposition of the ionic liquid effect, we conducted dynamic light scattering (DLS) experiments to observe the formation of chiral nanoaggregates of catalyst 1 in the presence of a nonpolar solvent. For a fair comparison, we also measured the size of the ionic aggregates in a polar solvent, which in principle would form far smaller aggregates. Considering that catalyst 1 is not soluble in hydrocarbons, we initially prepared a DMSO stock solution and then diluted it in the respective organic solvents for the experiment (details in the Experimental Section). The DLS measurements (Figure 1) indicated the formation of large aggregates in the nonpolar solvent and small aggregates in the polar solvents. This suggests that the reaction occurs within the large nanoaggregate environment, firmly pointing to the importance of the ionic liquid effect for achieving both high yields and selectivities in the classical Passerini multicomponent transformation.

**Figure 1:**
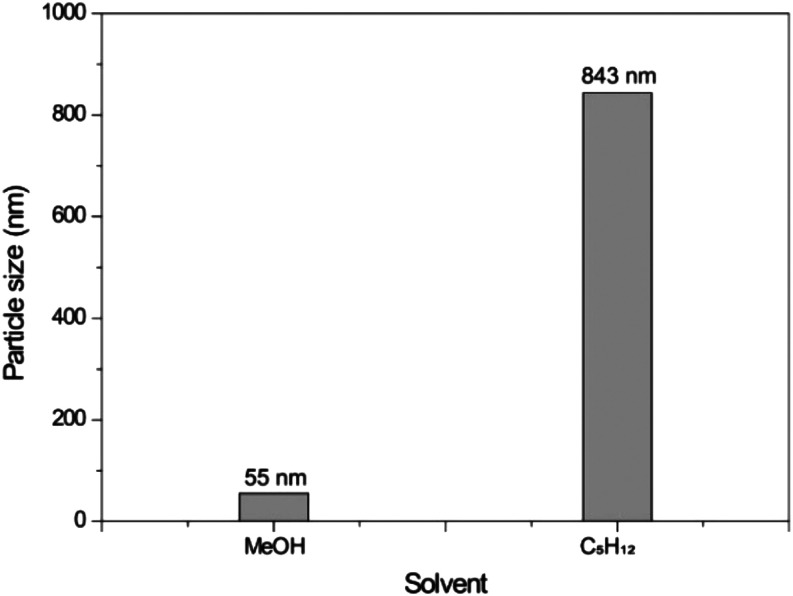
Nanoaggregate sizes by DLS experiments in nonpolar and polar solvents for the ionic liquid catalyst 1.

### Applicability of This Methodology to Various Aldehyde Components

After optimizing the reaction conditions and indicating the catalyst behavior, we explored their applicability to various aldehyde components (Scheme 2). Aromatic aldehydes substituted at the para position, including halogens, were generally well tolerated and yielded Passerini products with up to 81% yield and e.r. of 93:7. Evaluation of the electron-withdrawing group meta-chloro resulted in product 9f with a yield of 93% and e.r. of 80:20. Although aliphatic aldehydes were also viable, as evidenced by the formation of product 9g, a notable decrease in both yields and e.r. was observed. The presence of a para-substituent in the carboxylic acid component appears crucial for both selectivity and reactivity, as illustrated by the formation of products 9i and 9j using benzoic acid and 2-acetylbenzoic acid, respectively. Despite the substrate scope showing some limitations at present, it is important to emphasize the conceptual development of the ACDC/ionic liquid effect in the classic Passerini reaction. The absolute configuration of product 9j was determined through comparison of enantiodiscriminating HPLC analysis with literature data.^23^ The configuration of the other compounds was inferred by analogy.

**Scheme 2:**
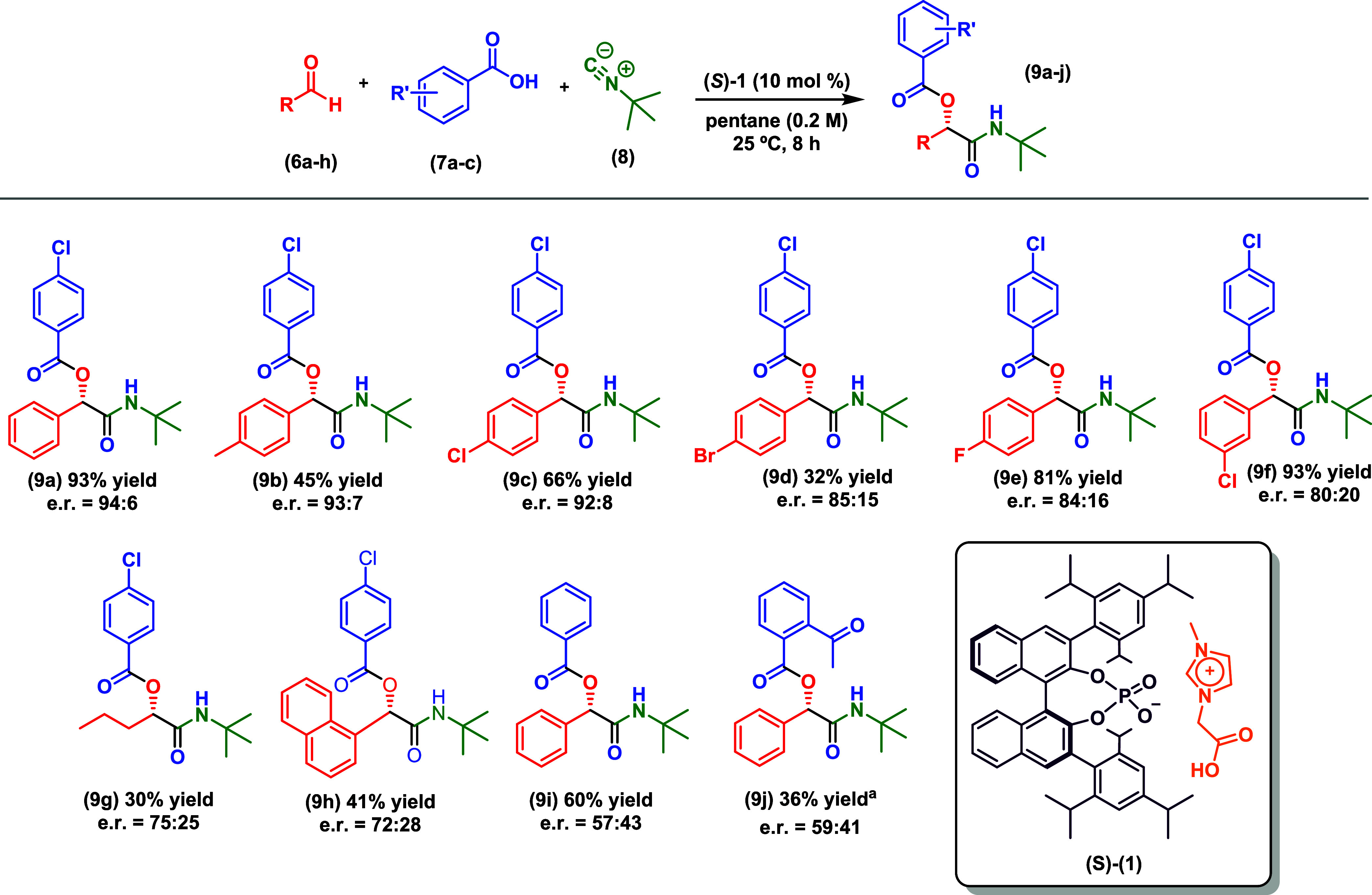
Scope of Asymmetric Passerini Reaction (Reactions were carried out employing 0.11 mmol of the aldehyde (6a–h), 0.10 mmol of the carboxylic acid (7a–c), and 0.10 mmol tert-butyl isocyanide). ^a^ This reaction was carried out using 20 mol % of *(S)*-1

### Kinetic Modeling Based on Experimental Observations

To propose a plausible reaction mechanism and understand the enantiodiscriminating step in the developed ionic liquid effect-based protocol for the Passerini reaction, we conducted a kinetic study using NMR analyses. The catalyzed reaction was monitored using ^1^H NMR, enabling real-time analysis of reagent consumption, formation/consumption of intermediates, and formation of the final adduct, as depicted in Figure 2. To validate the chemical shifts of intermediates, additional reactions between each pair of components were performed (i.e., reactions between the isocyanide and the carboxylic acid, the isocyanide and the aldehyde, and the aldehyde and the carboxylic acid; see Figures S42–S44 in the Supporting Information). A polar and aprotic solvent that does not display any overlay with the monitored signals could be selected. Based on these experiments, a kinetic model was proposed to elucidate the transformation, as outlined in Scheme 3 and codified in Table 3.

**Figure 2:**
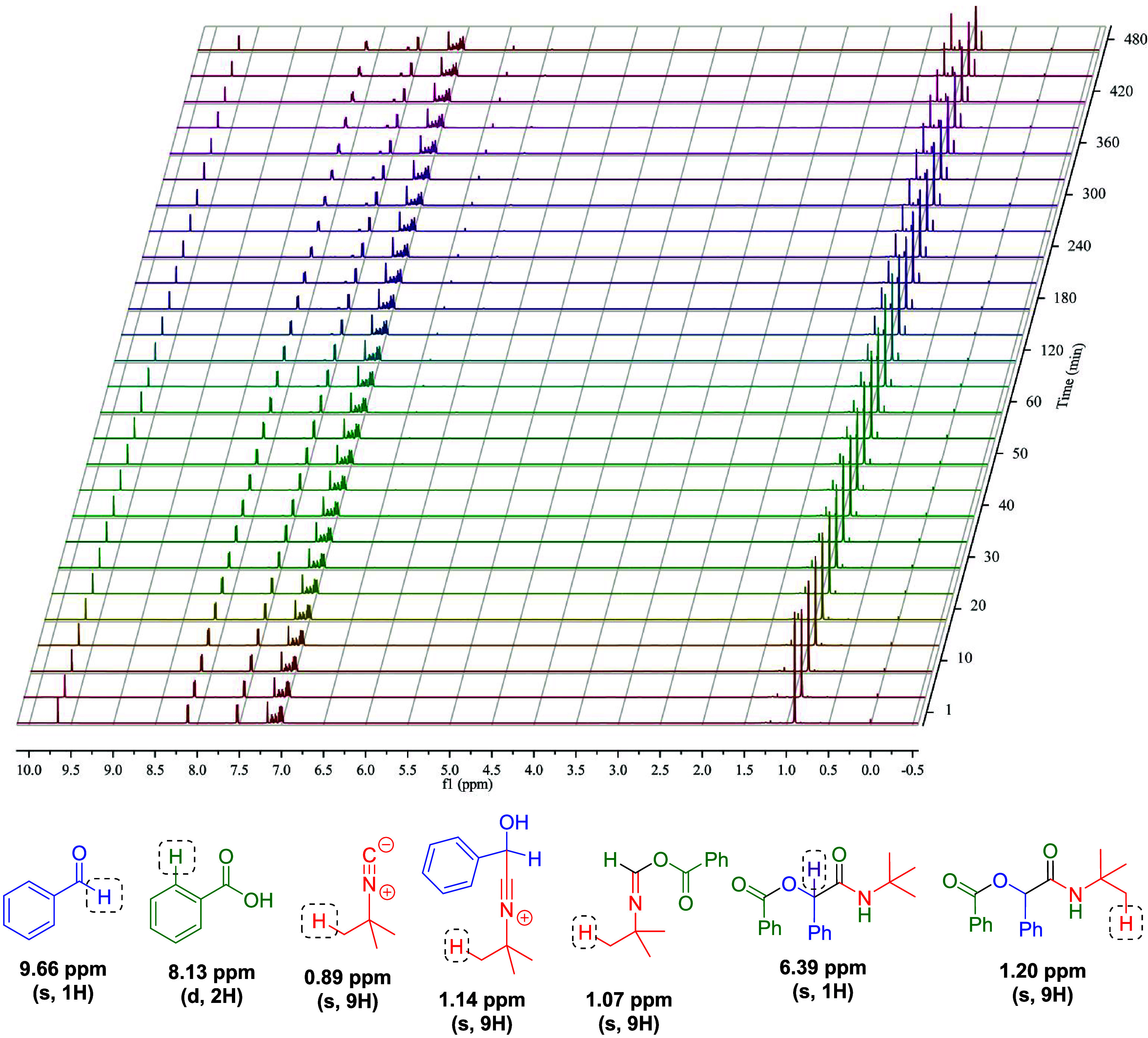
^1^H NMR (600 MHz, C_6_D_6_) reaction monitoring of the enantioselective catalyzed Passerini reaction during 8 h. ^a^ Initial conditions: aldehyde (0.11 mmol), isocyanide (0.10 mmol), carboxylic acid (0.10 mmol), and 0.5 mL of C_6_D_6_ and 10 mol % of the catalyst (1).

**Scheme 3:**
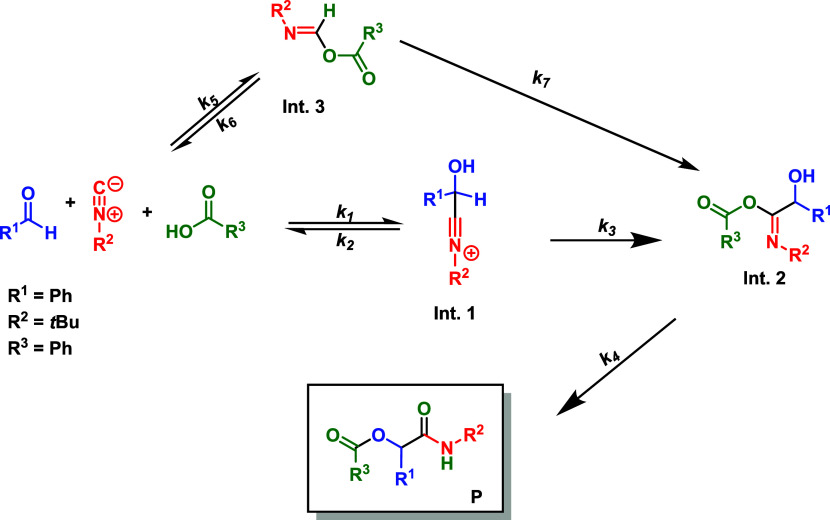
Kinetic Model and Mechanism Proposed to Explain the Catalyzed Enantioselective Passerini Reaction Promoted by Catalyst 1

**Table 3:** Components and Codes for the Kinetic Model Proposed in This Work

component	model code
benzaldehyde	*C*_*A*_
*tert*-butyl isocyanide	*C*_*B*_
benzoic acid	*C*_*C*_
**Int. 1**	*C*_*D*_
**Int. 2**	*C*_*E*_
**Int. 3**	*C*_*F*_
product (**P**)	*C*_*G*_

The differential mass balance to describe the kinetic behavior of the catalyzed Passerini multicomponent reaction, based on the kinetic mechanism expressed in Scheme 3, is provided as follows

1 $$\frac{dC_{A}}{\mathrm{dt}}=-\left(k_{1}+k_{5}\right)C_{A}C_{B}C_{C}+k_{2}C_{D}+k_{6}C_{F}$$

2 $$\frac{dC_{B}}{\mathrm{dt}}=\frac{dC_{A}}{\mathrm{dt}}$$

3 $$\frac{dC_{C}}{\mathrm{dt}}=\frac{dC_{A}}{\mathrm{dt}}$$

4 $$\frac{dC_{D}}{\mathrm{dt}}=k_{1}C_{A}C_{B}C_{C}-\left(k_{2}+k_{3}\right)C_{D}$$

5 $$\frac{dC_{E}}{\mathrm{dt}}=k_{3}C_{D}-k_{4}C_{E}+k_{7}C_{F}$$

6 $$\frac{dC_{F}}{\mathrm{dt}}=k_{5}C_{A}C_{B}C_{C}-\left(k_{6}+k_{7}\right)C_{F}$$

7 $$\frac{dC_{G}}{\mathrm{dt}}=k_{4}C_{E}$$

where *C*_*i*_ denotes the molar concentration of component *i* (N.B., the subscript *i* can assume the letter *A* to indicate benzaldehyde, while *B* corresponds to *tert*-butyl isocyanide, *C* is related to benzoic acid, *D* is for intermediate 1 (**Int. 1**), *E* is for intermediate 2 (**Int. 2**), whereas *F* is reserved for intermediate 3 (**Int. 3**), and *G* is related to the reaction product); *k*_*j*_ corresponds to the kinetic model constants (e.g., *j* from 1 to 7). Considering the volume constant, the molar fraction (*y*_*i*_) of component *i* can be expressed as

8 $$y_{i}=\frac{N_{i}}{\sum_{i}N_{i}}=\frac{C_{i}}{\sum_{i}C_{i}}$$

where *N*_*i*_ and *C*_*i*_ are the number of moles and the molar concentration of the component *i*, respectively.

To evaluate and predict the reaction behavior, the differential model equations were numerically solved using the Adams-Moulton multistep method,^52^ along with a numerical procedure for estimating the kinetic model parameters using direct search particle swarm optimization (PSO),^53–55^ implemented in FORTRAN (Intel Fortran Compiler 19.0 for Windows). The numerical tasks, comprising both parameter estimation and the solution of the set of ordinary differential equations, were carried out iteratively as a sequential optimization problem.^56,57^ For the differential kinetic model (eqs 1–7), the optimization problem can be defined as eq 9

9 $$\min_{\mathrm{k̲}}I=\sum_{i=1}^{\mathrm{NE}}\sum_{i=1}^{\mathrm{NC}}{\left(y_{i,l}^{m}-y_{i,l}^{e}\right)}^{2}$$

where *y*_*i,l*_^*m*^ is the output model represented by the concentration of the component *i* at the experimental condition *l*, *y*_*i,l*_^*e*^ is the experimental data corresponding to the component *i* at the experimental condition *l*, NE is related to the number of experiments, and NC corresponds to the number of components associated with the experimental data.

A standard sequential optimization procedure used for determining kinetic constants was based on PSO (eqs 10–12)^55^ to identify the best candidates for the kinetic model by minimizing eq 9. This population-based search optimization method considers *n* particles (candidates) moving along a multidimensional search space, exchanging information with other particles during the iterative process to find the minimum of the objective function.

10 $$v_{k}^{\left(i+1\right)}=w^{\left(i\right)}v_{k}^{\left(i\right)}+c_{1}\lambda \left(x_{k}^{\mathrm{best}}-x_{k}^{\left(i\right)}\right)+c_{2}\mu \left(x_{\mathrm{global}}-x_{k}^{\left(i\right)}\right)$$

11 $$x_{k}^{\left(i+1\right)}=x_{k}^{\left(i\right)}+v_{k}^{\left(i+1\right)}$$

where *c*_1_ and *c*_2_ are particle acceleration constants, λ and μ are random numbers with uniform distribution in the interval [0, 1], *x*_*k*_^best^ is the best position of particle *k* in the swarm, *x*_global_ corresponds to the best position found considering all particles in the swarm, *k* denotes the particle, *i* is related to the iteration number, *v*_*k*_^(*i*+1)^ corresponds to the velocity and *x*_*k*_^(*i*+1)^ is the position of each particle *k* in the swarm are stochastically, which are updated at each iteration *i*.

The inertia weight (*w*), used to ensure the convergence of particles to the optimal solution during the search, assumes values generally in the interval from 0.9 to 0.4. This function is represented in the present work as a nonlinear decreasing sigmoidal function (eq 12), as follows

12 $$w^{\left(i\right)}=w_{\max}-\frac{\left(w_{\max}-w_{\min}\right)}{1+e^{\left[-\xi_{1}\left(i-\xi_{2}\right)\right]}}$$

where *w*_max_ and *w*_min_ are the maximum and minimum values of the inertia weight *w* ^(*i*)^ in the interaction *i*, respectively, *i*_max_ is the maximum number of iterations (starting from iteration 1), and ξ_1_ and ξ_2_ are related to *i*_max_ in the following way ξ_1_ = 10/*i*_max_ and ξ_2_ = *i*_max_/2.

The estimated kinetic constants obtained from the sequential procedure are presented in Table 4. As illustrated in Figure 3, the proposed differential model accurately predicts the experimental concentration profiles of the reaction product and intermediate species.

**Table 4:** Estimated Kinetic Model Constants Based on a Sequential Numerical Procedure^a^

kinetic constant	parameter value
*k*_1_ (L^2^·mol^–2^·min^–1^)	0.0004
*k*_2_ (min^–1^)	0.3509
*k*_3_ (min^–1^)	0.0805
*k*_4_ (min^–1^)	0.8150
*k*_5_ (L^2^·mol^–2^·min^–1^)	0.0003
*k*_6_ (min^–1^)	0.1054
*k*_G,expt._ (L^2^·mol^–2^·min^–1^)	7.065 × 10^–5^

a *k*_7_ from Scheme 3 is zero.

**Figure 3:**
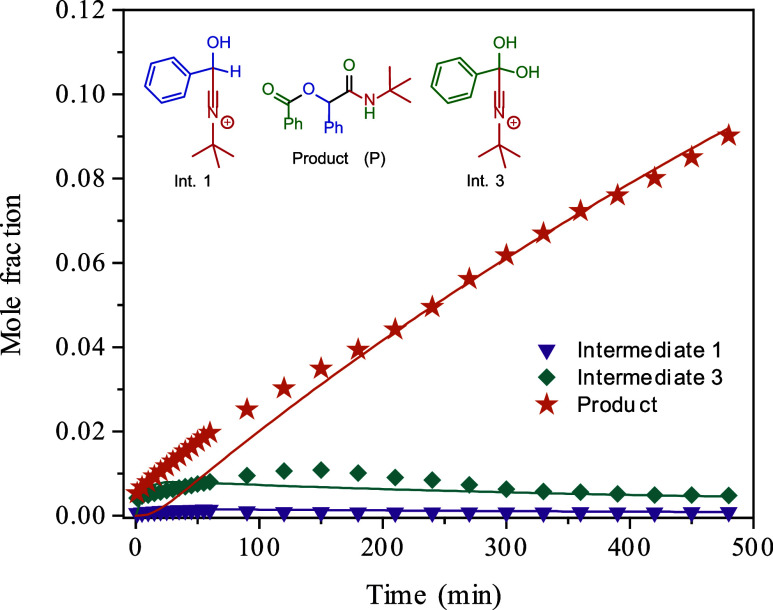
Differential model predictions based on a sequential optimization procedure. The solid lines represent the model prediction of the mole fraction of the components in the reaction system, while the symbols denote the experimental data. The mole fractions are estimated based on the NMR area (integral) of the signals.

The rate equation is written as *v* = *k*_4_ *C*_E_, and steady-state approximation is used for I1 (*C*_*D*_), I2 (*C*_*E*_), and I3 (*C*_*F*_).

13 $$\frac{dC_{D}}{\mathrm{dt}}=0∴k_{1}C_{A}C_{B}C_{C}-k_{2}C_{D}-k_{3}C_{D}=0∴C_{D}=\frac{k_{1}}{k_{2}+k_{3}}C_{A}C_{B}C_{C}$$

14 $$\frac{dC_{E}}{\mathrm{dt}}=0∴k_{3}C_{D}-k_{4}C_{E}+k_{7}C_{F}=0∴C_{E}=\frac{k_{3}}{k_{4}}C_{D}+\frac{k_{7}}{k_{4}}C_{F}$$

15 $$\frac{dC_{F}}{\mathrm{dt}}=0∴k_{5}C_{A}C_{B}C_{C}-\left(k_{6}+k_{7}\right)C_{F}=0∴C_{F}=\frac{k_{5}}{k_{6}+k_{7}}C_{A}C_{B}C_{C}$$

From previous equations, the global rate law is obtained.

16 $$v=\left[\frac{k_{3}k_{1}}{\left(k_{2}+k_{3}\right)}+\frac{k_{7}k_{5}}{\left(k_{6}+k_{7}\right)}\right]C_{A}C_{B}C_{C}$$

where $k_{G,\mathrm{calc}.}=\left[\frac{k_{3}k_{1}}{\left(k_{2}+k_{3}\right)}+\frac{k_{7}k_{5}}{\left(k_{6}+k_{7}\right)}\right]$ is the predicted global rate constant. Using *k*_7_ = 0 and the data from Table 3, *k*_*G*,calc._ = 7.464 × 10^–5^ L^2^ mol^–2^ min^–1^. This value is in very good agreement with the experimental global rate constant *k*_G,expt._ 7.065 × 10^–5^ L^2^ mol^–2^ min^–1^, supporting the proposed mechanism (Schemes 3 and 4). In this proposed kinetic model, *k*_7_ is set to zero because the transformation from **Int. 3** to **Int. 2** is mechanistically implausible, and no evidence supporting this transformation has been found. The data also suggest that **Int. 3** is a dead end in the reaction pathway, with the equilibrium shifting back to the reactants, thus supporting the proposed mechanism. This is illustrated in Scheme 4 and further supported by the data in Table 4 and Figure 3.

**Scheme 4:**
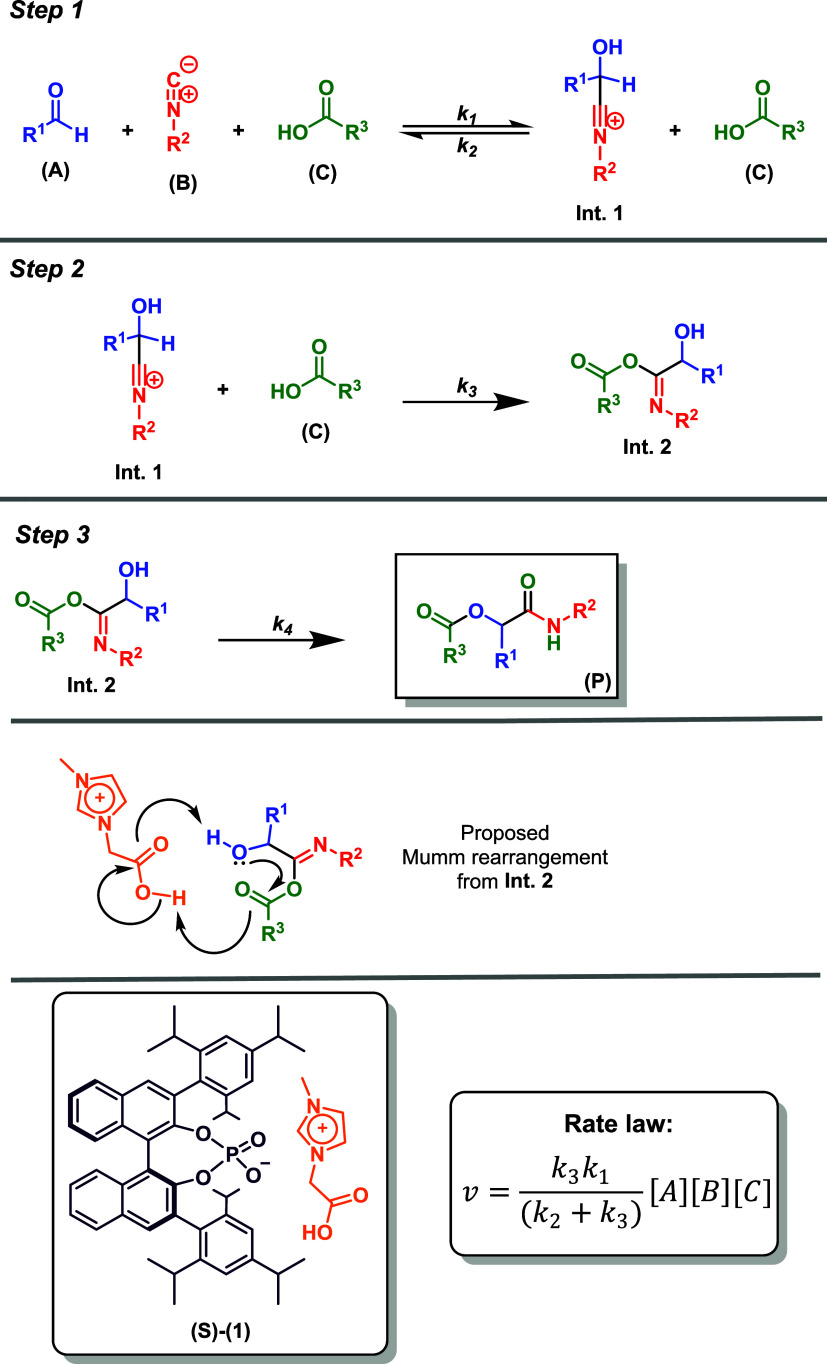
Kinetic Model and Mechanism Proposed to Explain the Catalyzed Enantioselective Passerini Reaction^a^

a Step 1: Isocyanide addition to the aldehyde. Step 2: **Int**. **1** reaction with carboxylic acid. Step 3: Proposed Mumm rearrangement from **Int**. **2** with the catalyst intervention.

From the data in Table 4, a mechanism can be proposed involving three steps (Scheme 4) to explain the Passerini adduct formation. Initially, the isocyanide addition to the aldehyde affords **Int. 1**, which is in turn trapped by the carboxylic acid and forms **Int. 2**. From **Int. 2**, a fast Mumm rearrangement takes place, likely facilitated by the presence of the carboxylic acid moiety in the cationic part of the chiral ionic liquid catalyst. The rate law proved to be independent of the Mumm rearrangement step (*k*_4_), which is in accordance with the predicted fast transformation of **Int. 2** into the final Passerini product.

The complex equilibria involved in the reagents, such as acid dimer formation, hinder more precise monitoring by NMR, but even though the kinetic model shows an excellent response in predicting their behavior. Considering the reactants consumption, the kinetic model provides a relatively good prediction of their behavior, as demonstrated in Figure S46 (Supporting Information). On the other hand, the more complex model, which considers the formation of all intermediates and the final product, accurately predicts the reaction behavior, as depicted in Figure 3 and indicated in Scheme 4. The obtained constants (Table 4) indicate the rapid consumption of **Int. 2** (Mumm rearrangement), leading to the formation of the final product (**P**) and hindering its monitoring by NMR. The presence of the carboxylic acid in the structures of catalyst **1** likely facilitates the rearrangement Scheme 4, highlighting the dual role of the ionic liquid catalyst, where both the cation and the anion are involved. This outcome is not surprising, as highly specific conditions are required to characterize such a transient intermediate.^58^

Additionally, the kinetic model suggests a preference toward the reagents when **Int. 3** (Scheme 3) is formed, firmly indicating a preference for the reaction pathway through **Int. 1**, as corroborated by the calculated constants in Table 4. These observations align with the NMR monitoring (Figures 1 and 2) of the reaction, where the formation of **Int. 1** is clearly observed earlier than **Int. 3**. The experimental data and kinetic constants strongly indicate that **Int. 3** is indeed a dead end in the reaction, and the equilibrium shifts back toward the reagents to proceed through the proposed three steps (Scheme 4). Overall, all the kinetic data suggests that the reaction is not readily promoted, which helps to explain the previously reported low yields in several studies and the time required for the reaction to proceed.

### High-Resolution ESI-MS­(/MS) Reaction Monitoring

To investigate the role of the catalyst in this truncated multicomponent transformation, high-resolution electrospray (tandem) mass spectrometry (ESI-MS­(/MS)) reaction monitoring was applied, yielding intriguing results (Figure 4). To avoid any ambiguity and enhance detection, the strategy of charge-tagged reagents was employed.^59^ In this context, we used 4-aminobenzoic acid, which yields a 4-ammonium derivative that is particularly prone to MS detection, especially under Bronsted acidic catalysis. Additionally, 4-bromobenzaldehyde was utilized to take advantage of the characteristic isotopic pattern of bromine-containing derivatives.^60–62^ With these established conditions, compelling evidence was obtained to enable unequivocal detection of the reaction intermediates and products formed (Figure 4).

**Figure 4:**
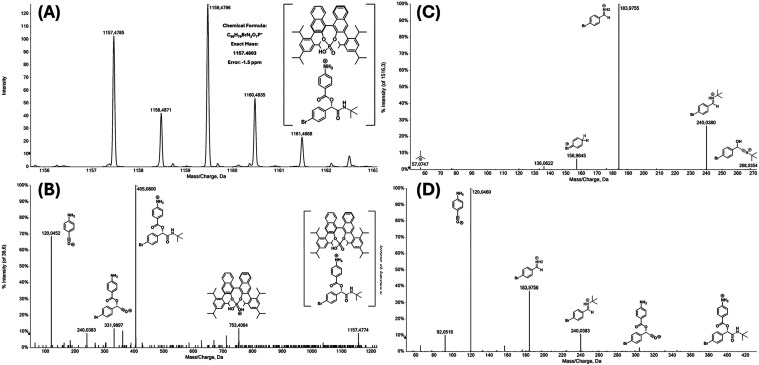
High-resolution ESI-MS­(/MS) monitoring of the Passerini MCR. (A) Exact mass of the supramolecular structure of *m*/*z* 1157 and its isotopic distribution. (B) ESI­(+)-MS/MS of the ion of *m*/*z* 1157. (C) ESI­(+)-MS/MS of the ion (nitrilium intermediate) of *m*/*z* 268. (D) ESI­(+)-MS/MS of the ion (Passerini adduct) of *m*/*z* 405.

One significant finding was the detection and characterization of an unprecedented supramolecular structure of *m*/*z* 1157, with an error margin as low as 1.5 ppm (Figure 4A). This result strongly supports the composition of the complex, which includes the Passerini adduct and the chiral phosphoric acid, as expected for H-bond complexes of imidazolium derivatives. Structural characterization of this signal was achieved via tandem MS/MS (Figure 4B), and the observed fragmentation pattern fully corroborates the proposed supramolecular structure. Additionally, the nitrilium intermediate (*m*/*z* 268) and the final Passerini adduct (*m*/*z* 405) were structurally confirmed through MS/MS experiments (Figure 4C,D, respectively).

The detection of the significant signal of *m*/*z* 1157 in the presence of the chiral inductor suggests catalyst involvement at various stages of the reaction. This is particularly noteworthy since the Mumm rearrangement is rapid, and, as described in previous studies^58^ detecting and characterizing the intermediate preceding the Mumm rearrangement requires specific conditions.

### Theoretical Investigation of the Reaction Mechanism

We have investigated the reaction mechanism considering the ion pair catalyzing the reaction between benzaldehyde, 4-chlorobenzoic acid, and *tert*-butyl isocyanide. The complete reaction mechanisms are presented in Figures 5 and 6, for the formation of (*S*)- and (*R*)-isomers, respectively.

**Figure 5:**
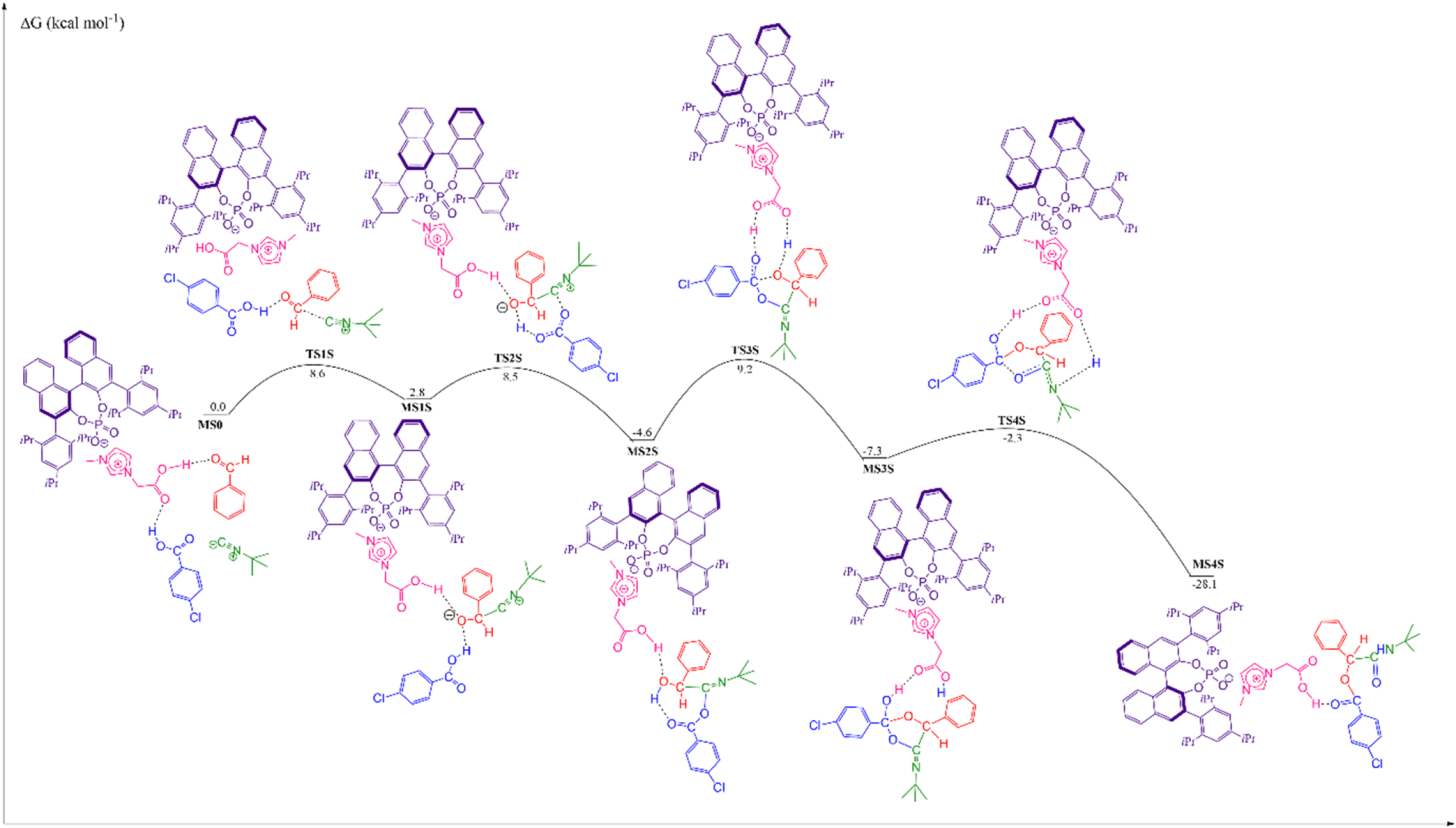
Free energy profile for the Passerini reaction between benzaldehyde, 4-chlorobenzoic acid, and *tert*-butyl isocyanide catalyzed by a chiral ion pair, referring to the formation of the S-isomer.

**Figure 6:**
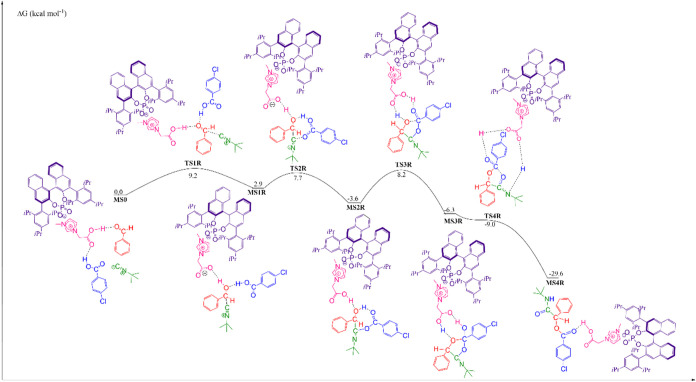
Free energy profile for the Passerini reaction between benzaldehyde, 4-chlorobenzoic acid, and *tert*-butyl isocyanide catalyzed by a chiral ion pair, referring to the formation of the R-isomer.

As can be seen in the Figures 5 and 6, for the first step, the enantioselective step, the transition state (TS) structures (that differ only the attack face), have in common the nucleophilic attack of isocyanide to the carbonyl carbon of benzaldehyde, simultaneously the carbonyl oxygen is stabilized by two hydrogen bonds, the first one with imidazolium ion from the catalyst structure, and the second one with the 4-chlorobenzoic acid. The free energy barrier for the formation of S-isomer, TS1S, is 8.6 kcal mol^–1^, and that for the formation of R-isomer, TS1R, is 9.2 kcal mol^–1^, a difference of 0.6 kcal mol^–1^ favoring the formation of S-isomer (72%), equivalent to an excess enantiomeric of 43%. This result is in line with the experimental result (94% of the S-isomer), where the enantioselective excess obtained (88%) is equivalent to a difference in Gibbs free energy of 1.6 kcal mol^–1^ between the transition states. After passing through the TS1, there is the formation of first intermediate, for the S-isomer, MS1S, with a free energy in solution of 2.8 kcal mol^–1^, and for the R-isomer, MS1R, the free energy in solution is 2.9 kcal mol^–1^.

In the next step of the reaction, there is the formation of a bond between a carbon atom from isocyanide and an oxygen atom of the 4-chlorobenzoic acid, with simultaneous transfer of a proton from the carboxylic acid to the oxygen atom of benzaldehyde. For the S-isomer, the free energy barrier of TS2S is 8.5 kcal mol^–1^, and for the product, MS2S, the solution free energy is −4.6 kcal mol^–1^. For the R-isomer, the free energy barrier of TS2R is 7.7 kcal mol^–1^, and for the product, MS2R, the solution free energy is −3.6 kcal mol^–1^.

In the third step, the rate-determining step, we observe the formation of a bond between the oxygen atom from benzaldehyde with the carbon atom from 4-chlorobenzoic acid, and the imidazolium ion catalyzing the transfer of a proton from the oxygen of benzaldehyde to the oxygen of 4-chlorobenzoic acid. For the S-isomer, the free energy barrier of TS3S is 9.2 kcal mol^–1^ in relation to MS0, but in relation to MS2S, intermediate with lower energy, the absolute barrier is 13.8 kcal mol^–1^. The product, MS3S, has a solution free energy of −7.3 kcal mol^–1^ in relation to MS0. For the R-isomer, the free energy barrier of TS3R is 8.2 kcal mol^–1^ in relation to MS0, but in relation to MS2R, intermediate with the lower energy, the absolute barrier is 11.8 kcal mol^–1^. The product, MS3R, has a solution free energy of −6.3 kcal mol^–1^ in relation to MS0.

Finally, in the last step, the breaking of a bond between carbon and oxygen atoms is observed, and the transfer of a proton from the hydroxyl group to the imine group is catalyzed by the chiral ion pair, allowing the formation of the product and reconstitution of the catalyst. For the S-isomer, TS4S has a free energy barrier of −2.3 kcal mol^–1^ in relation to MS0 and 5.0 kcal mol^–1^ in relation to MS3S, and the product, MS4S, has a solution free energy of −28.1 kcal mol^–1^. For the R-isomer, TS4R has a free energy barrier of −9.0 kcal mol^–1^ in relation to MS0 and −2.7 kcal mol^–1^ in relation to MS3R, and the product, MS4R, has a solution free energy of −29.6 kcal mol^–1^.

To obtain more detailed information about the intermolecular interactions present in the transition state structures of the enantioselective step, we use the NCI (noncovalent interactions) index, with the results shown in Figures 7 and 8. In both figures, we can see the van der Waals interaction between the aromatic ring of benzaldehyde and the chiral backbone from the Binol derivative (green region). In Figure 7, we can observe a hydrogen bond between the hydrogen atom of the hydroxyl group of the imidazolium ion and the oxygen atom of benzaldehyde, with a distance of 1.43 Å and a second hydrogen bond between 4-chlorobenzoic acid and benzaldehyde, with a distance of 1.61 Å. Both hydrogen bonds are in the blue region (λ_2_ < 0). In Figure 8, there is a hydrogen bond between the hydrogen atom of the hydroxyl group of the imidazolium ion and the oxygen atom of the phosphate anion, with a bond distance of 1.51 Å, and the second hydrogen bond between 4-chlorobenzoic acid and benzaldehyde, with a bond distance of 1.43 Å. Furthermore, there is one more attractive interaction, which is present only in TS1R, between the carbon of isocyanide and the carbonyl carbon of benzaldehyde, which can be seen as a blue ring around the carbon–carbon bond.

**Figure 7:**
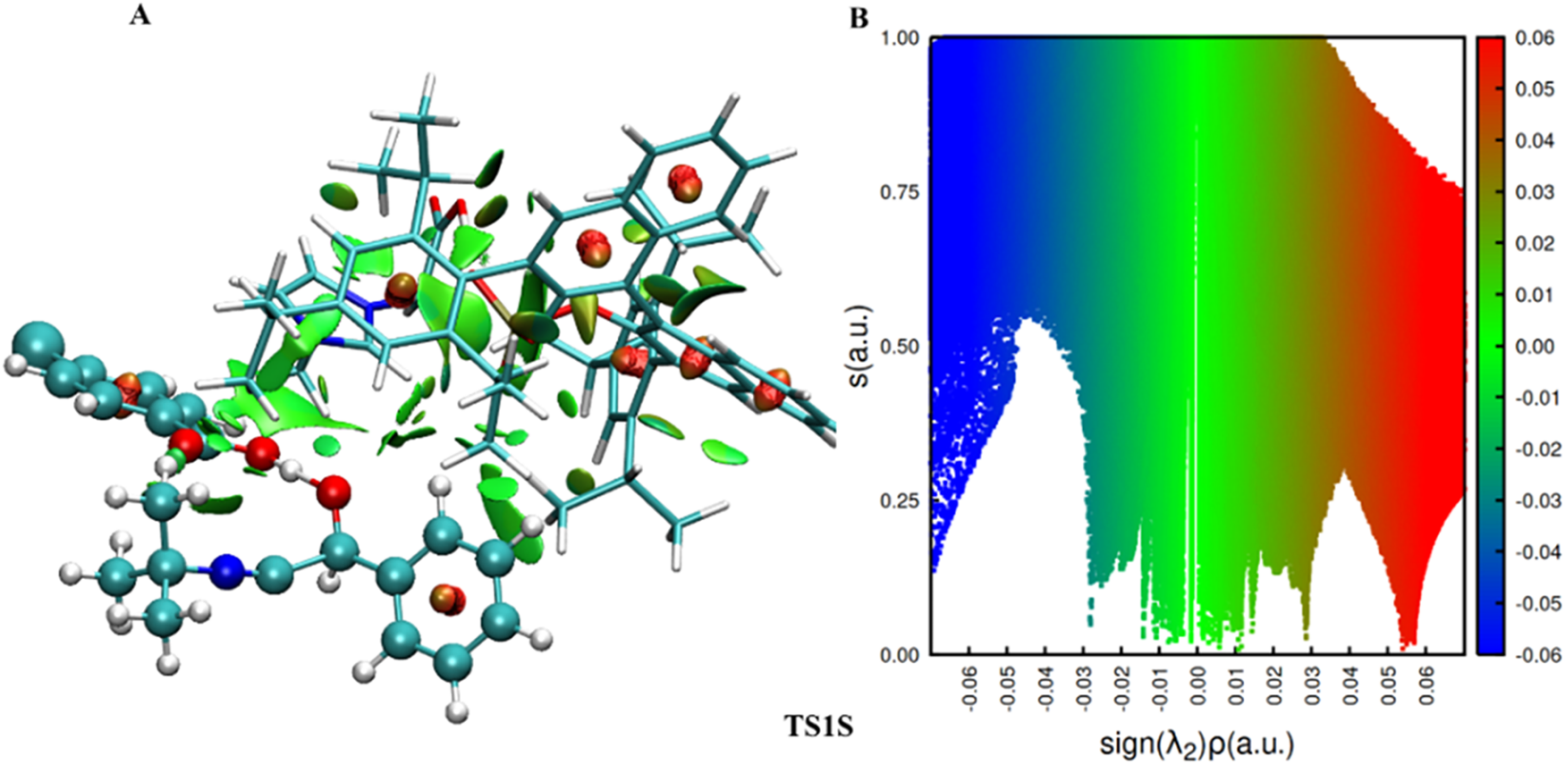
NCI analysis for the transition state structure involved in the first step for *(S)*-enantiomer pathway. (A) Isosurface colored based on the sign of λ_2_ρ: blue (strong attractive), green (weak attractive), red (repulsive); (B) reduced density gradient (s­(r)) versus the product of density (ρ) by the sign of the second eigenvalue of the Laplacian of the density (λ_2_).

**Figure 8:**
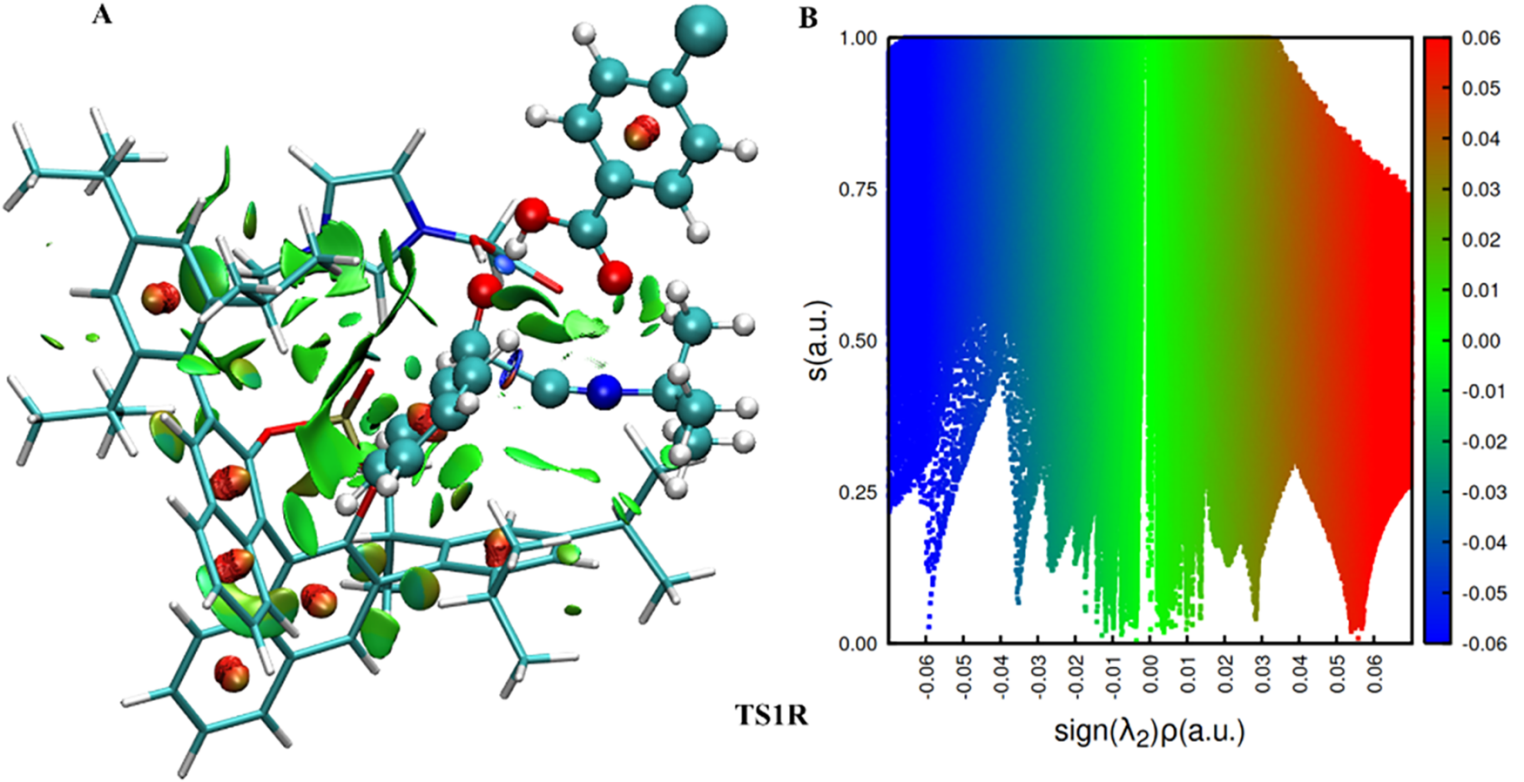
NCI analysis for the transition state structure involved in the first step for (*R*)-enantiomer pathway. (A) Isosurface colored based on the sign of λ_2_ρ: blue (strong attractive), green (weak attractive), red (repulsive); (B) reduced density gradient (s­(r)) versus the product of density (ρ) by the sign of the second eigenvalue of the Laplacian of the density (λ_2_).

## Conclusions

In this study, we developed an enantioselective methodology for the Passerini multicomponent reaction by leveraging the complex ionic liquid effect, encompassing various possibilities, including the ACDC effect. Despite some limitations, this strategy enabled the synthesis of α-acyloxyamide derivatives with high yields (up to 93%) and excellent enantiomeric ratios (94:6) under mild conditions. Our findings highlight the pivotal role of chiral ionic liquids in stabilizing reaction intermediates and facilitating chiral induction through the formation of an ion pair system. In addition, the formation of nanoaggregates may be involved as the main ionic liquid effect, as indicated by dynamic light scattering (DLS) experiments.

A comprehensive kinetic study, supported by NMR analyses, provided insight into the reaction mechanism, demonstrating that the process proceeds via the formation of nitrilium and imidate intermediates, followed by a Mumm rearrangement. The kinetic parameters obtained align with previous theoretical predictions and further substantiate the proposed mechanistic pathway. Additionally, high-resolution ESI-MS­(/MS) monitoring confirmed key intermediates and revealed the involvement of supramolecular interactions in the catalytic process.

Computational studies further corroborated the enantioselective step, demonstrating the preferential formation of the *S*-isomer and the role of van der Waals interactions in dictating TS stabilization. The observed agreement between experimental and theoretical enantiomeric excess reinforces the validity of our approach and points to the essential role of the complex ionic liquid effect in the chiral catalyst.

Overall, this work not only advances the understanding of the enantioselective Passerini MCR but also introduces a robust and generalizable strategy for asymmetric multicomponent transformations using chiral ionic liquid catalysis. These findings open new avenues for the development of chiral synthetic methodologies with broad applications in organic synthesis and pharmaceutical chemistry.

## Method

### General Remarks

All purchased chemicals were employed without further purification. Thin-layer chromatography (TLC) was performed on TLC plates (silica gel 60 F254) and visualized by a UV lamp. Melting points were recorded on an MQAPF-301-Microquimica digital apparatus. Column chromatography was performed using 230–400 mesh silica gel. The ^1^H and ^13^C NMR spectra were recorded at 500 and 125 MHz, respectively. Chemical shifts for ^1^H and ^13^C NMR were reported as δ (parts per million) relative to residual signals of the solvent (CDCl_3_ or DMSO-*d*_6_). Chemical shifts are reported employing the following peak abbreviation pattern: br, broad; s, singlet; d, doublet; dd, double doublet; m, multiplet. High-resolution mass spectra were acquired in the positive-ion mode using a time-of-flight (TOF) mass spectrometer equipped with an ESI source. Enantioselectivities were determined by chiral stationary phase HPLC analysis (Shimadzu HPLC, using a column Chiralpak IC).

### Dynamic Light Scattering (DLS) Experiments

A He–Ne laser with a wavelength of 633 nm and a power of 4 mW was used as the light source. Initially, a stock solution of the catalyst in DMSO at a concentration of 560 μM was prepared. This stock solution was then diluted to a concentration of 2.5 μM in the respective organic solvents. The solutions were placed in a quartz cuvette with a final volume of 1 mL. Adjustments were made in the equipment’s software for the refractive index, dielectric constant, and viscosity values of the respective organic solvents.

### General Method for the Preparation of the Catalysts

#### Catalysts (**1**–**5**) Were Prepared According to the Literature Method in Quantitative Yields^35^

##### Catalyst (*S*)-(**1**)

^1^H NMR (500 MHz, DMSO-*d*_6_): δ 9.08 (s, 1H), 8.04 (d, 2H, *J* = 8.2 Hz), 7.91 (s, 2H), 7.69 (s, 2H), 7.46 (t, 2H, *J* = 7.3 Hz), 7.32 (t, 2H, *J* = 7.5 Hz), 7.12 (s, 2H), 7.06–7.04 (m, 4H), 5.11 (s, 2H), 3.88 (s, 3H), 2.94–2.89 (m, 2H), 2.82–2.76 (m, 2H), 2.57–2.52 (m, 2H), 1.26 (d, 12H, *J* = 6.8 Hz), 1.15 (dd, 12H, *J* = 14.1, 6.8 Hz), 1.07 (d, 2H, *J* = 6.7 Hz), 0.88 (d, 2H, *J* = 6.7 Hz). ^13^C {1H} NMR (125 MHz, DMSO-*d*_6_): δ 168.6, 148.0, 147.9, 146.9, 138.0, 132.9, 132.6, 130.6, 128.9, 126.8, 126.1, 125.6, 124.2, 123.7, 122.1, 121.2, 120.2, 50.1, 36.3, 34.1, 31.1, 30.7, 26.7, 25.1, 24.6, 24.5, 23.7, 23.4. HRMS (ESI-TOF) *m*/*z* in the positive-ion mode [M]^+^ calcd for [C_6_H_9_N_2_O_2_H]^+^ 141.0664; found 141.0660. HRMS (ESI-TOF) *m*/*z* in the negative ion mode [M]^−^ calcd for [C_50_H_56_O_4_P]^−^ 751.3922; found 751.3943.

##### Catalyst **(** ***S*** **)-(2)**

^1^H NMR (500 MHz, DMSO-*d*_6_): δ ^1^H NMR (500 MHz, DMSO-*d*_6_): δ 9.10 (s, 1H), 7.95 (d, 1H, *J* = 8.1 Hz), 7.70 (s, 3H), 7.37 (t, 1H, *J* = 7.1 Hz), 7.24 (t, 1H, *J* = 7.2 Hz), 7.09 (d, 1H, *J* = 8.5 Hz), 7.03 (s, 1H), 6.92 (s, 1H), 5.07 (s, 2H), 3.88 (s, 3H), 2.71–2.66 (m, 1H), 2.14 (d, 1H, *J* = 8.7 Hz), 1.99–1.06 (m, 34H), 0.67–0.63 (m, 2H). ^13^C {1H} NMR (125 MHz, DMSO-*d*_6_): δ 167.2, 146.5, 145.9, 145.4, 137.5, 133.8, 132.1, 130.0, 129.5, 128.0, 125.5, 125.3, 124.1, 123.7, 123.1, 121.9, 121.6, 120.6, 49.8, 44.1, 41.0, 40.7, 40.0, 36.3, 35.8, 34.8, 34.1, 34.0, 32.4, 32.1, 27.0, 26.7, 26.5, 26.4, 25.9, 25.7.

##### Catalyst **(** ***R*** **)-(3)**

^1^H NMR (500 MHz, CDCl_3_): δ 9.16 (s, 0.21H), 7.77 (s, 0.12H), 7.69 (s, 2H), 7.46 (t, 2H, *J* = 7.3 Hz), 7.32 (t, 2H, *J* = 7.5 Hz), 7.12 (s, 2H), 7.06–7.04 (m, 4H), 5.11 (s, 2H), 3.88 (s, 3H), 2.94–2.89 (m, 2H), 2.82–2.76 (m, 2H), 2.57–2.52 (m, 2H), 1.26 (d, 12H, *J* = 6.8 Hz), 1.15 (dd, 12H, *J* = 14.1, 6.8 Hz), 1.07 (d, 2H, *J* = 6.7 Hz), 0.88 (d, 2H, *J* = 6.7 Hz). ^13^C {1H} NMR (125 MHz, CDCl_3_): δ 168.6, 148.0, 147.9, 146.9, 138.0, 132.9, 132.6, 130.6, 128.9, 126.8, 126.1, 125.6, 124.2, 123.7, 122.1, 121.2, 120.2, 50.1, 36.3, 34.1, 31.1, 30.7, 26.7, 25.1, 24.6, 24.5, 23.7, 23.4.

##### Catalyst **(** ***S*** **)-(4)**

Spectral data are in accordance with those described in the literature.^35^

##### Catalyst **(** ***S*** **)-(5)**

^1^H NMR (500 MHz, DMSO-*d_6_*): δ 9.17 (s, 1H), 8.07 (d, 2H, *J* = 8.2 Hz), 7.77 (s, 1H), 7.14 (s, 0.2H), 7.04 (s, 0.2H), 6.69 (s, 2H), 6.63 (s, 2H), 6.59 (s, 2H), 4.52 (s, 0.4H), 3.59 (s, 1H), 2.61–2.44 (m, 7H), 2.21–2.07 (m, 7H), 1.95 (t, 3H, *J* = 11.7 Hz), 1.48–0.71 (m, 58H). ^13^C {1H} NMR (125 MHz, DMSO-*d*_6_): δ 146.2, 145.9, 145.4, 144.3, 144.2, 135.8, 132.7, 132.3, 131.5, 128.6, 126.3, 123.2, 121.6, 120.7, 56.9, 50.2, 44.1, 41.1, 41.0, 36.5, 35.7, 34.5, 34.1, 34.0, 33.0, 32.4, 28.5, 27.2, 26.8, 26.6, 26.4, 26.3, 26.1, 25.8, 25.7, 25.6, 22.3, 22.2.

### General Procedure for the Asymmetric Counteranion-Directed Catalyzed Passerini Reaction

To a 1.5 mL vial containing a freshly prepared chiral catalyst (*S*)-**1** (0.01 mmol, 0.1 equiv), 0.5 mL of pentane was added. Under magnetic stirring, to this suspension, the aldehyde was added (0.11 mmol, 1.1 equiv) and the mixture was stirred for 5 min. Then, the corresponding carboxylic acid (0.10 mmol, 1.0 equiv) and *tert*-butyl isocyanide (0.10 mmol, 1.0 equiv) were added, and the reaction mixture was vigorously stirred at 26 °C for 8 h in a sealed tube. The reaction crude was transferred with H_2_O/DCM to a separatory funnel and extracted with DCM for two times (2 × 20 mL). The combined organic layers were concentrated under reduced pressure to give the crude Passerini products **9a**–**i**. After HPLC analysis, the crude reaction mixtures were purified through flash chromatography on silica gel (hexanes/EtOAc = 3:1).

(*S*)*-2-*(*tert-Butylamino*)*-2-oxo-1-phenylethyl 4-chlorobenzoate* (**9a**) was obtained in 93% yield (0.032 g) as a white solid.^63^ ^1^H NMR (500 MHz, CDCl_3_): δ 8.02 (d, 2H, *J* = 8.5 Hz), 7.51 (d, 2H, *J* = 7.2 Hz), 7.44 (d, 2H, *J* = 8.5 Hz), 7.37–7.41 (m, 3H), 6.17 (s, 1H), 5.86 (br, 1H), 1.35 (s, 9H). ^13^C {1H} NMR (125 MHz, CDCl_3_): δ 167.2, 164.4, 140.2, 135.8, 131.3, 129.2, 129.0, 128.0, 127.6, 76.4, 51.8, 28.8.

(*S*)*-2-*(*tert-Butylamino*)*-2-oxo-1-*(*p-tolyl*)*ethyl 4-chlorobenzoate* (**9b**) was obtained in 51% yield (0.016 g) as a white solid. mp 151.8–153.0 °C. FT-IR (νmax, cm^–1^): 3288 (NH), 1720, 1654 (CO). ^1^H NMR (500 MHz, CDCl_3_): δ 8.01 (d, 2H, *J* = 8.5 Hz), 7.43 (d, 2H, *J* = 8.6 Hz), 7.39 (d, 2H, *J* = 8.0 Hz), 7.20 (d, 2H, *J* = 7.9 Hz), 6.14 (s, 1H), 5.81 (br, 1H), 2.35 (s, 3H), 1.35 (s, 9H). ^13^C {1H} NMR (125 MHz, CDCl_3_): δ 167.4, 164.4, 140.1, 139.2, 132.9, 131.3, 129.7, 129.1, 128.1, 127.7, 76.3, 51.8, 28.8, 21.4. HRMS (ESI-TOF) *m*/*z*: [M + Na]^+^ calcd for C_20_H_22_ClNNaO_3_, 382.1186; found, 382.1177.

(*S*)*-2-*(*tert-Butylamino*)*-1-*(*4-chlorophenyl*)*-2-oxoethyl 4-chlorobenzoate* (**9c**) was obtained in 66% yield (0.025 g) as a white solid. mp 144.7–145.6 °C. FT-IR (ν_max_, cm^–1^): 3286 (NH), 1724, 1652 (CO). ^1^H NMR (500 MHz, CDCl_3_): δ 8.00 (d, 2H, *J* = 8.5 Hz), 7.46–7.44 (m, 4H), 7.36 (d, 2H, *J* = 8.3 Hz), 6.14 (s, 1H), 5.91 (br, 1H), 1.35 (s, 9H). ^13^C {1H} NMR (125 MHz, CDCl_3_): δ 166.8, 164.2, 140.4, 135.2, 134.3, 131.2, 129.2, 129.1, 129.0, 127.7, 75.3, 51.9, 28.8. HRMS (ESI-TOF) *m*/*z*: [M + Na]^+^ calcd for C_19_H_19_Cl_2_NNaO_3_, 402.0640; found, 402.0639.

(*S*)*-2-*(*tert-Butylamino*)*-1-*(*4-bromophenyl*)*-2-oxoethyl 4-chlorobenzoate* (**9d**) was obtained in 32% yield (0.014 g) as a white solid. mp 151.1–151.7 °C. FT-IR (ν_max_, cm^–1^): 3282 (NH), 1724, 1654 (CO). ^1^H NMR (500 MHz, CDCl_3_): δ 8.50 (d, 2H, *J* = 8.5 Hz), 7.53 (d, 2H, *J* = 8.4 Hz), 7.45 (d, 2H, *J* = 8.5 Hz), 7.38 (d, 2H, *J* = 8.4 Hz), 6.12 (s, 1H), 5.89 (br, 1H), 1.36 (s, 9H). ^13^C {1H} NMR (125 MHz, CDCl_3_): δ 166.8, 164.2, 140.5, 134.8, 132.2, 131.3, 129.3, 129.2, 127.7, 123.4, 75.7, 51.9, 28.8. HRMS (ESI-TOF) *m*/*z*: [M + Na]^+^ calcd for C_19_H_19_BrClNNaO_3_, 446.0135; found, 446.0125.

(*S*)*-2-*(*tert-Butylamino*)*-1-*(*4-fluorophenyl*)*-2-oxoethyl 4-chlorobenzoate* (**9e**) was obtained in 81% yield (0.029 g) as a white solid. mp 169.7–170.2 °C. FT-IR (ν_max_, cm^–1^): 3268 (NH), 1728, 1652 (CO). ^1^H NMR (500 MHz, CDCl_3_): δ 8.00 (d, 2H, *J* = 8.6 Hz), 7.50–7.44 (m, 4H), 7.08 (t, 2H, *J* = 8.6 Hz), 6.15 (s, 1H), 5.89 (br, 1H), 1.36 (s, 9H). ^13^C {1H} NMR (125 MHz, CDCl_3_): δ 167.1, 164.3, 164.2, 162.2, 140.4, 131.2, 129.6, 129.5, 129.2, 127.8, 116.1, 115.9, 75.6, 51.9, 28.8. HRMS (ESI-TOF) *m*/*z*: [M + Na]^+^ calcd for C_19_H_19_ClFNNaO_3_, 386.0935; found, 386.0934.

(*S*)*-2-*(*tert-Butylamino*)*-1-*(*3-chlorophenyl*)*-2-oxoethyl 4-chlorobenzoate* (**9f**) was obtained in 93% yield (0.036 g) as a beige solid. mp 130.5–131.3 °C. FT-IR (ν_max_, cm^–1^): 3299 (NH), 1724, 1656 (CO). ^1^H NMR (500 MHz, CDCl_3_): δ 8.02 (d, 2H, *J* = 8.7 Hz), 7.49–7.46 (m, 3H), 7.40 (d, 1H, *J* = 6.5 Hz), 7.34–7.32 (m, 2H), 6.13 (s, 1H), 5.91 (br, 1H), 1.36 (s, 9H). ^13^C {1H} NMR (125 MHz, CDCl_3_): δ 166.6, 164.1, 140.5, 137.7, 134.8, 131.3, 130.2, 129.4, 129.2, 127.6, 125.9, 75.6, 51.9, 28.8. HRMS (ESI-TOF) *m*/*z*: [M + Na]^+^ calcd for C_19_H_20_Cl_2_NO_3_, 380.0820; found, 380.0810.

(*S*)*-1-*(*tert-Butylamino*)*-1-oxopentan-2-yl benzoate* (**9g**) was obtained in 30% yield (0.011 g) as a white solid. mp 83.1–84.1 °C. FT-IR (ν_max_, cm^–1^): 3295 (NH), 1724, 1656 (CO). ^1^H NMR (500 MHz, CDCl_3_): δ 8.99 (d, 2H, *J* = 8.7 Hz), 7.46 (d, 2H, *J* = 8.6 Hz), 5.81 (br, 1H), 5.26 (t, 1H, *J* = 6.0 Hz), 1.95–1.91 (m, 2H), 1.47–1.40 (m, 2H), 1.35 (s, 9H), 0.99–0.92 (m, 3H). ^13^C {1H} NMR (125 MHz, CDCl_3_): δ 168.9, 164.7, 140.2, 131.2, 129.2, 128.1, 75.1, 51.5, 34.1, 28.8, 18.4, 13.9. HRMS (ESI-TOF) *m*/*z*: [M + H]^+^ calcd for C_16_H_23_ClNO_3_, 312.1366; found, 312.1366.

(*S*)*-2-*(*tert-Butylamino*)*-1-*(*1-naphthyl*)*-2-oxoethyl 4-chlorobenzoate* (**9h**) was obtained in 41% yield (0.016 g) as a beige solid. mp 120.5–121.0 °C. FT-IR (ν_max_, cm^–1^): 3315 (NH), 1726, 1670 (CO). ^1^H NMR (500 MHz, CDCl_3_): δ 8.26 (d, 1H, *J* = 8.3 Hz), 8.03 (d, 2H, *J* = 8.3 Hz), 7.92–7.93 (m, 2H), 7.70 (d, 1H, *J* = 7.0 Hz), 7.50–7.63 (m, 3H), 7.42 (d, 2H, *J* = 8.3 Hz), 6.87 (s, 1H), 5.69 (s, 1H), 1.33 (s, 9H). ^13^C {1H} NMR (125 MHz, CDCl_3_): δ 167.5, 164.7, 140.1, 134.3, 131.7, 131.5, 131.4, 130.4, 129.1, 129.0, 128.1, 127.9, 126.4, 125.4, 124.0, 74.8, 51.9, 28.7. HRMS (ESI-TOF) *m*/*z*: [M + H]^+^ calcd for C_23_H_23_ClNO_3_, 396.1366; found, 396.1378.

(*S*)*-2-*(*tert-Butylamino*)*-2-oxo-1-phenylethyl benzoate* (**9i**) was obtained in 60% yield (0.019 g) as a white solid.^24^ ^1^H NMR (500 MHz, CDCl_3_): δ 8.09 (d, 2H, *J* = 7.4 Hz), 7.60 (t, 1H, *J* = 7.4 Hz), 7.53 (d, 2H, *J* = 7.1 Hz), 7.47 (t, 2H, *J* = 7.7 Hz), 7.35–7.41 (m, 3H), 6.22 (s, 1H), 6.02 (br, 1H), 1.36 (s, 9H). ^13^C {1H} NMR (125 MHz, CDCl_3_): δ 167.5, 165.0, 136.0, 133.7, 129.9, 129.5, 128.9, 128.7, 127.5, 76.2, 51.7, 28.8.

(*S*)*-2-*(*tert-Butylamino*)*-2-oxo-1-phenylethyl 2-acetylbenzoate* (**9j**) was obtained in 36% yield (0.014 g) as a colorless oil.^23^ ^1^H NMR (500 MHz, CDCl_3_): δ 7.85 (dd, 1H, *J* = 7.5 Hz, *J* = 0.6 Hz), 7.53–7.59 (m, 3H), 7.45 (dd, 2H, *J* = 7.8 Hz, *J* = 1.7 Hz), 7.36–7.37 (m, 3H), 6.49 (br, 1H), 6.16 (s, 1H), 1.42 (s, 9H). ^13^C {1H} NMR (125 MHz, CDCl_3_): δ 203.0, 167.4, 166.1, 141.3, 135.8, 132.2, 131.0, 130.5, 129.1, 128.8, 127.9, 127.1, 76.9, 51.9, 29.9, 28.8.

### Theoretical Methodology—Electronic Structure Calculations

The enantioselective step for the Passerini reaction was computationally investigated. Geometry optimization and harmonic frequencies calculations were conducted at the X3LYP^64^/def2-SVP^65^ (ma-def2-SVP^66^ for O and N atoms) level of theory. To obtain more accurate values of electronic energy, some single point energy calculations were performed with the M06-2X^67^ functional and def2-TZVPP (ma-def2-TZVPP for O and N atoms) basis set. The solvation free energy for pentane was obtained by single point energy calculations at the SMD^68^/X3LYP/def2-SVP (ma-def2-SVP for O and N atoms) level of theory. Finally, the solution free energy can be determined, according to eq 17

17 $$G_{\mathrm{sol}}=E_{\mathrm{ele}}+G_{T}+\Delta G_{\mathrm{solv}}+1.89\,\;\mathrm{kcal}{\;\mathrm{mol}}^{-1}$$

where *G*_sol_ refers to the solution free energy. The first term on the right side of eq 17 is the electronic energy obtained at the M06–2X/def2-TZVPP (ma-def2-TZVPP for O and N atoms) level of theory; the second term refers to the nuclear contribution to translation, rotation and vibration, obtained at the X3LYP/def2-SVP (ma-def2-SVP for O and N atoms) level of theory; the third term refers to the solvation free energy obtained at SMD/X3LYP/def2-SVP (ma-def2-SVP for O and N atoms) level of theory; and the last term refers to the correction of the change in the standard state from 1 atm to 1 mol L^–1^. All the calculations were done with the Orca 5 program.^69–71^

To visualize the intermolecular interactions, we used the NCI (Noncovalent Interaction) index.^72^ The NCI index uses the reduced density gradient (RDG) to identify the intermolecular interactions and can generate two graphs. The first one is a 3D graphic, which is the plot of RDG vs density (ρ). And the second one, a 2D graphic, that makes use of the sign of the second density Hessian eigenvalue multiplied by the density (sign­(λ_2)­ρ) to differentiate favorable and unfavorable intermolecular interactions. To visualize the 3D graphic, we have used VMD.

### Calculation of Enantiomeric Excess (% ee)

For the Passerini reaction investigated here, the first step is enantioselective determination. To calculate the enantiomeric excess, the rate constants corresponding to the formation of the *R* (k_*R*_) and *S* (k_*S*_) isomers were determined, according to eq 18

18 $$k_{R\left(S\right)}\left(T\right)=\frac{k_{b}T}{h}e^{{\Delta G}_{\mathrm{TS}}^{‡}/\mathrm{RT}}$$

where Δ*G*_*TS*_^‡^ is the activation free energy. The enantiomeric excess (%ee) can be determined by eq 19

19 $$\%\mathrm{ee}=\frac{k_{S}-k_{R}}{k_{S}+k_{R}}\times 100\%$$

## Supplementary Material



## Data Availability

The data underlying this study are available in the published article and its Supporting Information.

## Notes

Details for all experimental procedures, additional optimization experiments, copies of ^1^H, ^13^C­{^1^H}, and IR spectra for all compounds, HRMS and UV spectra, computational details, relative and absolute energies, reaction energy profiles, and Cartesian coordinates of all computed structures (PDF)

## References

[1] Dömling A., Wang W., Wang K. (2012). Chemistry
and Biology Of Multicomponent Reactions. Chem.
Rev..

[2] Cioc R. C., Estévez V., van der Niet D. J., Vande Velde C. M. L., Turrini N. G., Hall M., Faber K., Ruijter E., Orru R. V. A. (2017). Stereoselective
Synthesis of Functionalized Bicyclic
Scaffolds by Passerini 3-Center-2-Component Reactions of Cyclic Ketoacids. Eur. J. Org. Chem..

[3] Zhi S., Ma X., Zhang W. (2019). Consecutive Multicomponent Reactions for the Synthesis
of Complex Molecules. Org. Biomol. Chem..

[4] Vishwanatha T. M., Giepmans B., Goda S. K., Dömling A. (2020). Tubulysin
Synthesis Featuring Stereoselective Catalysis and Highly Convergent
Multicomponent Assembly. Org. Lett..

[5] Owens T. D., Araldi G.-L., Nutt R. F., Semple J. E. (2001). Concise Total Synthesis
of the Prolyl Endopeptidase Inhibitor Eurystatin A via a Novel Passerini
Reaction–Deprotection–Acyl Migration Strategy. Tetrahedron Lett..

[6] Hosokawa S., Nakanishi K., Udagawa Y., Maeda M., Sato S., Nakano K., Masuda T., Ichikawa Y. (2020). Total Synthesis of
Exigurin: The Ugi Reaction in a Hypothetical Biosynthesis of Natural
Products. Org. Biomol. Chem..

[7] Brown A. L., Churches Q. I., Hutton C. A. (2015). Total Synthesis
of Ustiloxin D Utilizing
an Ammonia–Ugi Reaction. J. Org. Chem..

[8] Isaacson J., Kobayashi Y. (2009). An Ugi Reaction in the Total Synthesis of (−)-Dysibetaine. Angew. Chem., Int. Ed..

[9] Wang Q., Wang D.-X., Wang M.-X., Zhu J. (2018). Still Unconquered:
Enantioselective Passerini and Ugi Multicomponent Reactions. Acc. Chem. Res..

[10] Rocha R. O., Rodrigues M. O., Neto B. A. D. (2020). Review on the Ugi Multicomponent
Reaction Mechanism and the Use of Fluorescent Derivatives as Functional
Chromophores. ACS Omega.

[11] Zhang J., Yu P., Li S., Sun H., Xiang S., Wang J. J., Houk K. N., Tan B. (2018). Asymmetric
Phosphoric Acid–Catalyzed
Four-Component Ugi Reaction. Science.

[12] Moni L., Banfi L., Basso A., Martino E., Riva R. (2016). Diastereoselective
Passerini Reaction of Biobased Chiral Aldehydes: Divergent Synthesis
of Various Polyfunctionalized Heterocycles. Org. Lett..

[13] Banfi L., Basso A., Lambruschini C., Moni L., Riva R. (2021). The 100 Facets
of the Passerini Reaction. Chem. Sci..

[14] Yang K., Zhang F., Fang T., Li C., Li W., Song Q. (2021). Passerini-Type Reaction of Boronic
Acids Enables α-Hydroxyketones
Synthesis. Nat. Commun..

[15] Li J., Zheng Q., Dömling A. (2024). Exploring
Phthalimide as the Acid
Component in the Passerini Reaction. Org. Lett..

[16] Mousapour M., Hassani S. A. M., Shirini F. (2022). First Asymmetric Synthesis of Passerini-Type
Condensation Products in Water Using Pregabalin: A Chiral Amino Acid
for the Efficient Asymmetric Induction. ChemistrySelect.

[17] Denmark S. E., Fan Y. (2003). The First Catalytic,
Asymmetric α-Additions of Isocyanides.
Lewis-Base-Catalyzed, Enantioselective Passerini-Type Reactions. J. Am. Chem. Soc..

[18] Frey R., Galbraith S. G., Guelfi S., Lamberth C., Zeller M. (2003). First Examples
of a HighlyStereoselective Passerini Reaction: A New Access to Enantiopure
Mandelamides. Synlett.

[19] Yue T., Wang M.-X., Wang D.-X., Masson G., Zhu J. (2009). Catalytic
Asymmetric Passerini-Type Reaction: Chiral Aluminum–Organophosphate-Catalyzed
Enantioselective α-Addition of Isocyanides to Aldehydes. J. Org. Chem..

[20] Kusebauch U., Beck B., Messer K., Herdtweck E., Dömling A. (2003). Massive Parallel Catalyst Screening:
Toward Asymmetric
MCRs. Org. Lett..

[21] Andreana P. R., Liu C. C., Schreiber S. L. (2004). Stereochemical
Control of the Passerini
Reaction. Org. Lett..

[22] Wang S.-X., Wang M.-X., Wang D.-X., Zhu J. (2008). Catalytic Enantioselective
Passerini Three-Component Reaction. Angew. Chem.,
Int. Ed..

[23] Zhang J., Lin S.-X., Cheng D.-J., Liu X.-Y., Tan B. (2015). Phosphoric
Acid-Catalyzed Asymmetric Classic Passerini Reaction. J. Am. Chem. Soc..

[24] Antenucci A., Marra F., Dughera S. (2021). Silica Gel-Immobilised
Chiral 1,2-Benzenedisulfonimide:
A Brønsted Acid Heterogeneous Catalyst for Enantioselective Multicomponent
Passerini Reaction. RSC Adv..

[25] Mahlau M., List B. (2013). Asymmetric Counteranion-Directed
Catalysis: Concept, Definition, and Applications. Angew. Chem., Int. Ed..

[26] Parmar D., Sugiono E., Raja S., Rueping M. (2014). Complete Field Guide
to Asymmetric BINOL-Phosphate Derived Brønsted Acid and Metal
Catalysis: History and Classification by Mode of Activation; Brønsted
Acidity, Hydrogen Bonding, Ion Pairing, and Metal Phosphates. Chem. Rev..

[27] Ramos L. M., Rodrigues M. O., Neto B. A. D. (2019). Mechanistic Knowledge and Noncovalent
Interactions as the Key Features for Enantioselective Catalysed Multicomponent
Reactions: A Critical Review. Org. Biomol. Chem..

[28] Yu X.-L., Kuang L., Chen S., Zhu X.-L., Li Z.-L., Tan B., Liu X.-Y. (2016). Counteranion-Controlled
Unprecedented Diastereo- and
Enantioselective Tandem Formal Povarov Reaction for Construction of
Bioactive Octahydro-Dipyrroloquinolines. ACS
Catal..

[29] Yin Z., Guo J., Zhang R., Hu X., Borovkov V. (2020). Direct Asymmetric
Three-Component
Mannich Reaction Catalyzed by Chiral Counteranion-Assisted Silver. J. Org. Chem..

[30] Qian D., Chen M., Bissember A. C., Sun J. (2018). Counterion-Induced
Asymmetric Control in Ring-Opening of Azetidiniums: Facile Access
to Chiral Amines. Angew. Chem., Int. Ed..

[31] Qian D., Chen M., Bissember A. C., Sun J. (2018). Counterion-Induced
Asymmetric Control in Ring-Opening of Azetidiniums: Facile Access
to Chiral Amines. Angew. Chem..

[32] Zhang X., Zhao K., Li N., Yu J., Gong L., Gu Z. (2020). Atroposelective Ring Opening of Cyclic Diaryliodonium Salts with
Bulky Anilines Controlled by a Chiral Cobalt­(III) Anion. Angew. Chem., Int. Ed..

[33] Zhang X., Zhao K., Li N., Yu J., Gong L., Gu Z. (2020). Atroposelective Ring Opening of Cyclic
Diaryliodonium Salts with
Bulky Anilines Controlled by a Chiral Cobalt­(III) Anion. Angew. Chem..

[34] Neto B. A. D., Rocha R. O., Lapis A. A. M. (2022). What
Do We Know about the Ionic Liquid
Effect in Catalyzed Multicomponent Reactions?: A Critical Review. Curr. Opin. Green Sustainable Chem..

[35] Alvim H. G. O., Pinheiro D. L. J., Carvalho-Silva V. H., Fioramonte M., Gozzo F. C., da Silva W. A., Amarante G. W., Neto B. A. D. (2018). Combined Role of the Asymmetric Counteranion-Directed
Catalysis (ACDC) and Ionic Liquid Effect for the Enantioselective
Biginelli Multicomponent Reaction. J. Org. Chem..

[36] Rodrigues M. O., Eberlin M. N., Neto B. A. D. (2021). How and Why to Investigate Multicomponent
Reactions Mechanisms? A Critical Review. Chem.
Rec..

[37] Alvim H. G. O., da Silva Júnior E. N., Neto B. A. D. (2014). What Do We Know
about Multicomponent Reactions? Mechanisms and Trends for the Biginelli,
Hantzsch, Mannich, Passerini and Ugi MCRs. RSC
Adv..

[38] Ramozzi R., Morokuma K. (2015). Revisiting the Passerini Reaction Mechanism: Existence
of the Nitrilium, Organocatalysis of Its Formation, and Solvent Effect. J. Org. Chem..

[39] Zhang H.-R., Gao G., Zhang Q., Tian X.-C., Yuan W.-J., Zhao Z.-B., Yan C.-X., Wang J.-J., Tu C.-Z., Xie D., Zhou P.-P., Yang Z. (2024). Mechanism and Origin of Enantioselectivity
for Asymmetric Passerini Reaction in the Synthesis of α-Acyloxyamide
Catalyzed by Chiral Phosphoric Acid. Mol. Catal..

[40] Maeda S., Komagawa S., Uchiyama M., Morokuma K. (2011). Finding Reaction Pathways
for Multicomponent Reactions: The Passerini Reaction Is a Four-Component
Reaction. Angew. Chem., Int. Ed..

[41] Stassen H. K., Ludwig R., Wulf A., Dupont J. (2015). Imidazolium Salt Ion
Pairs in Solution. Chem. - Eur. J..

[42] Harada S., Hirose S., Takamura M., Furutani M., Hayashi Y., Nemoto T. (2024). Silver­(I)/Dirhodium­(II)
Catalytic Platform for Asymmetric
N–H Insertion Reaction of Heteroaromatics. J. Am. Chem. Soc..

[43] Jhang Y.-J., Zhelavskyi O., Nagorny P. (2023). Enantioselective Parallel Kinetic
Resolution of Aziridine-Containing Quinoxalines via Chiral Phosphoric
Acid-Catalyzed Transfer Hydrogenation. Org.
Lett..

[44] Chaudhari M. B., Gupta P., Llanes P., Pericàs M. A. (2023). Polymer-Supported
Phosphoric-Acid Catalysed Enantioselective Pictet-Spengler Cyclisation
for the Synthesis of Quaternary Tryptolines in Batch/Continuous Flow. Adv. Synth. Catal..

[45] Lazzarotto M., Hartmann P., Pletz J., Belaj F., Kroutil W., Payer S. E., Fuchs M. (2021). Asymmetric
Allylation Catalyzed by
Chiral Phosphoric Acids: Stereoselective Synthesis of Tertiary Alcohols
and a Reagent-Based Switch in Stereopreference. Adv. Synth. Catal..

[46] Zhang L., Zhu R., Feng A., Zhao C., Chen L., Feng G., Liu L. (2020). Redox Deracemization
of β,γ-Alkynyl α-Amino Esters. Chem. Sci..

[47] Dupont J., Leal B. C., Lozano P., Monteiro A. L., Migowski P., Scholten J. D. (2024). Ionic Liquids in Metal, Photo-, Electro-, and (Bio)
Catalysis. Chem. Rev..

[48] Li X., Song X., Li L., Wei Y., Liu G., Xia Q. (2023). Effect of Different Counterions on
the Self-Assembly Structures and
Properties of Imidazole Based Ionic Liquids Surfactant: A Molecular
Dynamics Study. J. Mol. Liq..

[49] Kuznetsova D. A., Kuznetsov D. M., Amerhanova S. K., Buzmakova E. V., Lyubina A. P., Syakaev V. V., Nizameev I. R., Kadirov M. K., Voloshina A. D., Zakharova L. Y. (2022). Cationic Imidazolium Amphiphiles
Bearing a Methoxyphenyl Fragment: Synthesis, Self-Assembly Behavior,
and Antimicrobial Activity. Langmuir.

[50] Paprocki D., Koszelewski D., Walde P., Ostaszewski R. (2015). Efficient
Passerini Reactions in an Aqueous Vesicle System. RSC Adv..

[51] Watanabe H., Doi H., Saito S., Matsugami M., Fujii K., Kanzaki R., Kameda Y., Umebayashi Y. (2016). Hydrogen Bond in Imidazolium Based
Protic and Aprotic Ionic Liquids. J. Mol. Liq..

[52] Coker, A. K. Fortran Programs for Chemical Process Design, Analysis, and Simulation, 1st ed.; Gulf Professional Publishing: Houston, 1995.

[53] Shi, Y. ; Eberhart, R. A Modified Particle Swarm Optimizer. In 1998 IEEE International Conference on Evolutionary Computation Proceedings. IEEE World Congress on Computational Intelligence (Cat. No. 98TH8360); IEEE pp 69–73 10.1109/ICEC.1998.699146.

[54] Kennedy, J. ; Eberhart, R. Particle Swarm Optimization. In Proceedings of ICNN’95 - International Conference on Neural Networks; IEEE 4, pp 1942–1948 10.1109/ICNN.1995.488968.

[55] Eberhart, R. C. ; Shi, Y. ; Kennedy, J. Swarm Intelligence, 1st ed.; Morgan Kaufmann: San Francisco, 2001.

[56] Vassiliadis V. S., Sargent R. W. H., Pantelides C. C. (1994). Solution
of a Class of Multistage
Dynamic Optimization Problems. 1. Problems without Path Constraints. Ind. Eng. Chem. Res..

[57] Vassiliadis V. S., Sargent R. W. H., Pantelides C. C. (1994). Solution
of a Class of Multistage
Dynamic Optimization Problems. 2. Problems with Path Constraints. Ind. Eng. Chem. Res..

[58] Iacobucci C., Reale S., Aschi M., Oomens J., Berden G., De Angelis F. (2018). An Unprecedented
Retro-Mumm Rearrangement Revealed
by ESI-MS/MS, IRMPD Spectroscopy, and DFT Calculations. Chem. - Eur. J..

[59] Limberger J., Leal B. C., Monteiro A. L., Dupont J. (2015). Charge-Tagged Ligands:
Useful Tools for Immobilising Complexes and Detecting Reaction Species
during Catalysis. Chem. Sci..

[60] Ma T., Shen Z., Li H., Li A., Feng Q., Sun Y., Deng S. (2020). Effect of H-Bonding
on Brønsted Acid Ionic Liquids
Catalyzed In Situ Transesterification of Wet Algae. ACS Sustainable Chem. Eng..

[61] Yu M., Liu J., Cao X., Wei C., Liang H., Gong C., Ju Z. (2024). Structures and Hydrogen
Bonds of -SO3H Functionalized Acid Ionic
Liquids. J. Mol. Liq..

[62] Li K., Yan Y., Zhao J., Lei J., Jia X., Mushrif S. H., Yang Y. (2016). Understanding the Role
of Hydrogen Bonding in Brønsted Acidic
Ionic Liquid-Catalyzed Transesterification: A Combined Theoretical
and Experimental Investigation. Phys. Chem.
Chem. Phys..

[63] Polindara-García L. A., Juaristi E. (2016). Synthesis of Ugi 4-CR and Passerini 3-CR Adducts under
Mechanochemical Activation. Eur. J. Org. Chem..

[64] Xu X., Zhang Q., Muller R. P., Goddard W. A. (2005). An Extended Hybrid
Density Functional (X3LYP) with Improved Descriptions of Nonbond Interactions
and Thermodynamic Properties of Molecular Systems. J. Chem. Phys..

[65] Weigend F., Ahlrichs R. (2005). Balanced Basis Sets
of Split Valence, Triple Zeta Valence
and Quadruple Zeta Valence Quality for H to Rn: Design and Assessment
of Accuracy. Phys. Chem. Chem. Phys..

[66] Zheng J., Xu X., Truhlar D. G. (2011). Minimally
Augmented Karlsruhe Basis Sets. Theor. Chem.
Acc..

[67] Zhao Y., Truhlar D. G. (2008). The M06 Suite of Density Functionals
for Main Group
Thermochemistry, Thermochemical Kinetics, Noncovalent Interactions,
Excited States, and Transition Elements: Two New Functionals and Systematic
Testing of Four M06-Class Functionals and 12 Other Function. Theor. Chem. Acc..

[68] Marenich A. V., Cramer C. J., Truhlar D. G. (2009). Universal
Solvation Model Based on
Solute Electron Density and on a Continuum Model of the Solvent Defined
by the Bulk Dielectric Constant and Atomic Surface Tensions. J. Phys. Chem. B.

[69] Neese F., Wennmohs F., Becker U., Riplinger C. (2020). The ORCA Quantum
Chemistry Program Package. J. Chem. Phys..

[70] Neese F. (2018). Software Update:
The ORCA Program System, Version 4.0. WIREs
Comput. Mol. Sci..

[71] Neese F. (2012). The ORCA Program
System. WIREs Comput. Mol. Sci..

[72] Contreras-García J., Johnson E. R., Keinan S., Chaudret R., Piquemal J.-P., Beratan D. N., Yang W. (2011). NCIPLOT: A Program for Plotting Noncovalent
Interaction Regions. J. Chem. Theory Comput..

