# Supplementary material for: Kinetics and Mechanism of the Enantioselective Passerini Multicomponent Reaction Catalyzed by Chiral Ionic Liquids
Source: ACS Omega. 2026 Apr 16;11(16):24281–97. doi: 10.1021/acsomega.5c13327 (PMC13129842; doi:10.1021/acsomega.5c13327)
Supplement: Supplementary file 1 [file ao5c13327_si_001.pdf]

Supporting Information for

Kinetics and Mechanism of the Enantioselective Passerini Multicomponent Reaction Catalyzed by Chiral Ionic Liquids

Juliana Santos,[a,‡] Pedro P. De Castro,[b,‡] Saulo T. A. Passos,[c,‡] Virginia Camila Rufino Ferreira,[a] Fabrício Machado,[c] Daniel M. Araújo,[a] Hélio F. Dos Santos,[,a] Brenno A. D. Neto,[*,c] and Giovanni W. Amarante[*,a]*

[a] Department of Chemistry, Federal University of Juiz de Fora, Rua José Lourenço Kelmer, Campus Universitário São Pedro, Juiz de Fora, Minas Gerais 36036-900, Brazil.

[b] Department of Pharmacy, Federal University of Juiz de Fora - Campus Governador Valadares, 35010-180, Governador Valadares – MG, Brazil.

[c] Laboratory of Medicinal and Technological Chemistry, University of Brasília, Chemistry Institute (IQ-UnB), Campus Universitário Darcy Ribeiro, Brasília, Distrito Federal 70910-900, Brazil.

‡ These authors equally contributed to this study.

* E-mail: helio.santos@ufjf.br, brenno.ipi@gmail.com, giovanni.amarante@ufjf.br

Table of Contents

S1. Additional optimization of reaction conditions **3**

S2. Chiral HPLC traces for Table S1 entries **4**

S3. Chiral HPLC traces for Table 2 entries **6**

S4. Chiral HPLC traces for the final products **12**

S5. Kinetic study database **22**

S6. Copies of IR, ^{1}H and ^{13}C {1H} NMR spectra for all compounds **27**

S7. Copies of HRMS (ESI-TOF) spectra for all novel compounds **52**

S8. Determination of the absolute configuration of major Passerini products **55**

S9. Tables with reaction and activation thermodynamic properties. **57**

S10. Coordinate the optimized structures. **58**

S1. Additional optimization of reaction conditions

Table S1. Optimization of reaction molar concentration

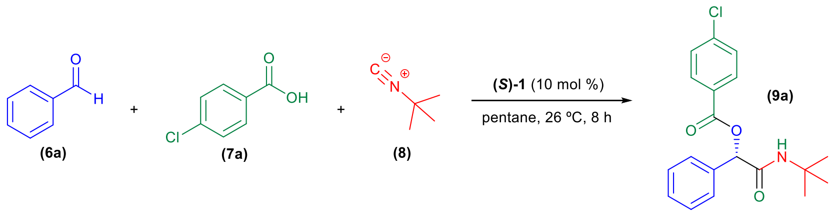

Entry	Concentration of reaction[a]	Yield (%)[b]	e.r.[c]
**1**	0.8 M	51	68:32
**2**	0.4 M	55	68:32
**3**	0.2 M	93	94:06
**4**	0.1 M	53	92:08
**5**	0.05 M	ND	70:30

[a] In relation to **8**; [b] Isolated yield; [c] Determined using enantiodiscriminating HPLC; ND: not determined.

S2. Chiral HPLC traces for Table S1 entries

Figure S1. HPLC traces of entry 1 – Table S1

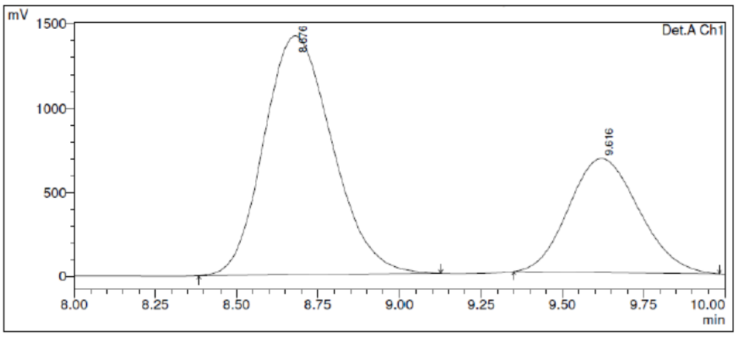

Peak#	Ret. Time	Area	Height	Area %	Height %
1	8.676	20814772	1417182	67.047	67.605
2	9.616	10230150	679101	32.953	32.395
Total		31044922	2096283	100.000	100.000

Figure S2. HPLC traces of entry 2 – Table S1

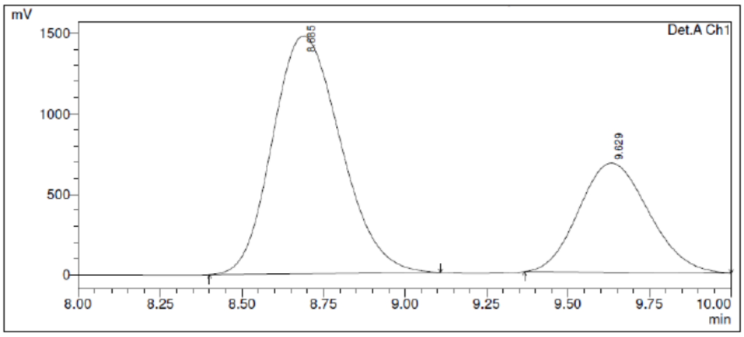

Peak#	Ret. Time	Area	Height	Area %	Height %
1	8.685	21518337	1477384	67.954	68.531
2	9.629	10147483	678391	32.046	31.469
Total		31665821	2155775	100.000	100.000

Figure S3. HPLC traces of entry 3 – Table S1

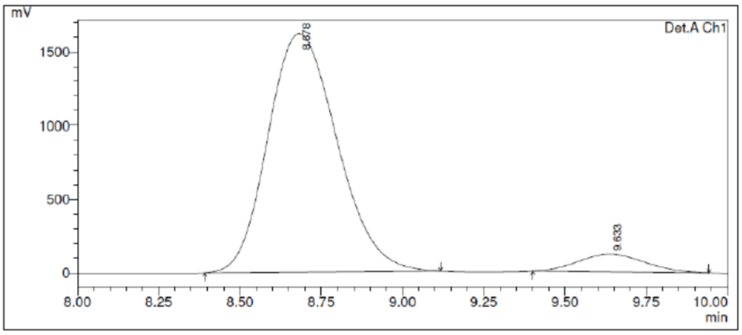

Peak#	Ret. Time	Area	Height	Area %	Height %
1	8.678	23997731	1619910	93.332	93.050
2	9.633	1714623	120994	6.668	6.950
Total		25712354	1740904	100.000	100.000

Figure S4. HPLC traces of entry 4 – Table S1

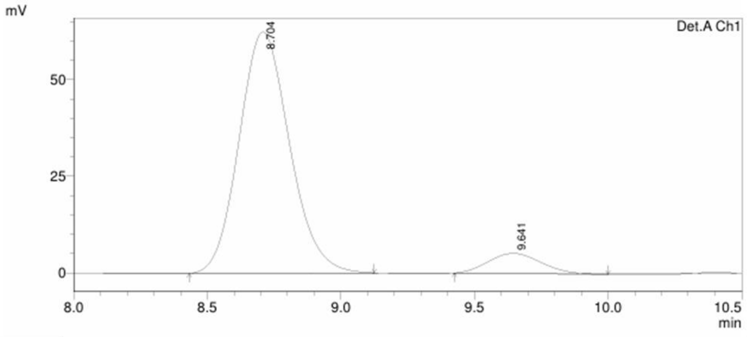

Peak#	Ret. Time	Area	Height	Area %	Height %
1	8.704	818146	62377	91.889	92.276
2	9.641	72215	5221	8.111	7.724
Total		890361	67598	100.000	100.000

Figure S5. HPLC traces of entry 5 – Table S1

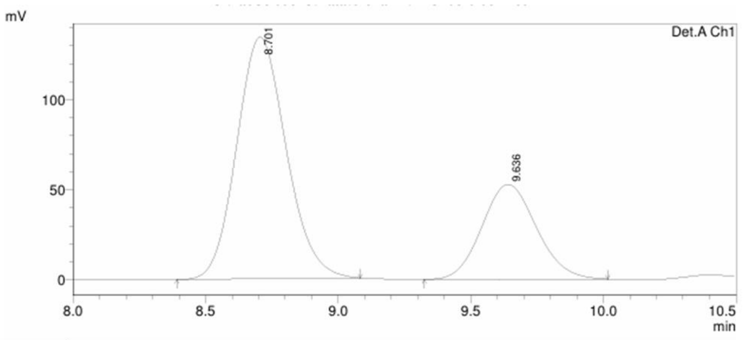

Peak#	Ret. Time	Area	Height	Area %	Height %
1	8.701	1741320	134191	69.849	71.811
2	9.636	751648	52675	30.151	28.189
Total		2492968	186866	100.000	100.000

S3. Chiral HPLC traces for Table 2 entries

Figure S6. HPLC traces of entry 1 – Table 2

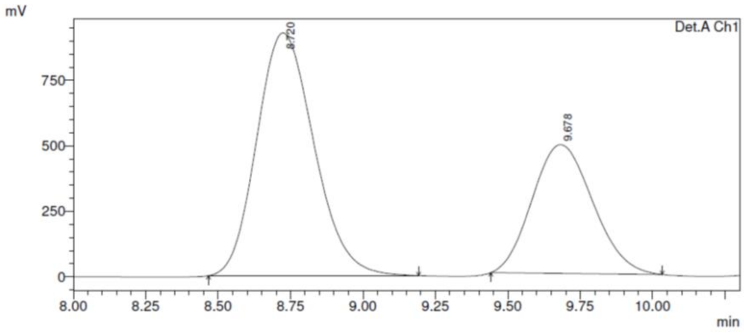

Peak#	Ret. Time	Area	Height	Area %	Height %
1	8.720	12749303	927195	64.000	65.354
2	9.678	7171381	491531	36.000	34.646
Total		19920684	1418726	100.000	100.000

Figure S7. HPLC traces of entry 2 – Table 2

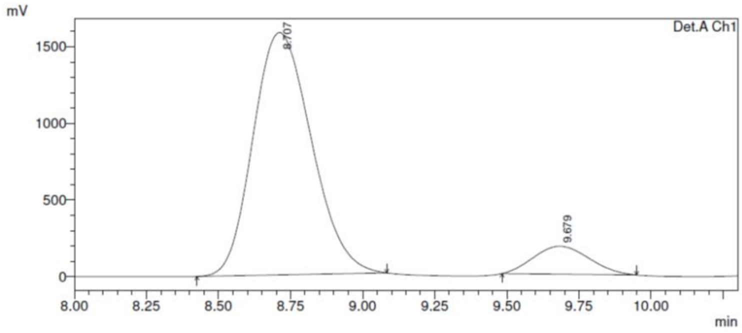

Peak#	Ret. Time	Area	Height	Area %	Height %
1	8.707	22299604	1581697	90.127	89.686
2	9.679	2442760	181904	9.873	10.314
Total		24742365	1763601	100.000	100.000

Figure S8. HPLC traces of entry 3 – Table 2

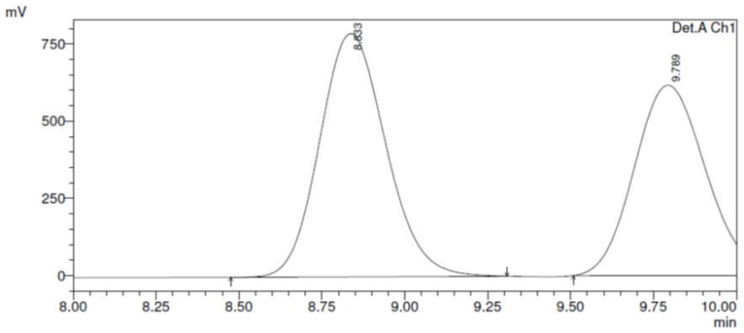

Peak#	Ret. Time	Area	Height	Area %	Height %
1	8.833	10953864	787053	54.150	56.087
2	9.789	9274897	616211	45.850	43.913
Total		20228761	1403264	100.000	100.000

Figure S9. HPLC traces of entry 4 – Table 2

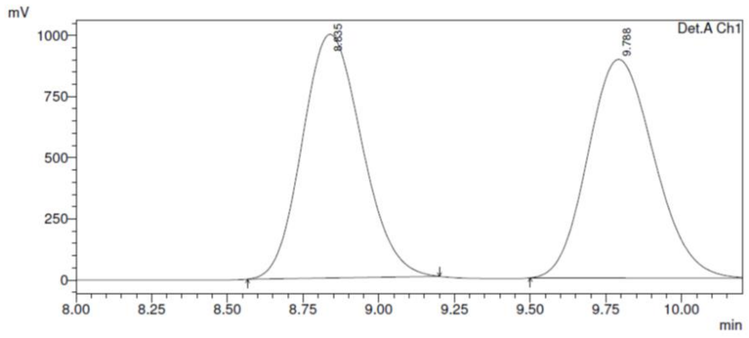

Peak#	Ret. Time	Area	Height	Area %	Height %
1	8.835	13646915	995659	50.036	52.684
2	9.788	13627281	894225	49.964	47.316
Total		27274196	1889884	100.000	100.000

Figure S10. HPLC traces of entry 5 – Table 2

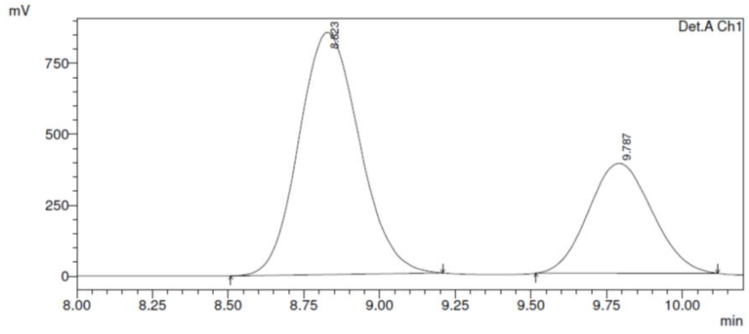

Peak#	Ret. Time	Area	Height	Area %	Height %
1	8.823	11840517	851967	67.590	68.768
2	9.787	5677701	386927	32.410	31.232
Total		17518217	1238894	100.000	100.000

Figure S11. HPLC traces of entry 6 – Table 2

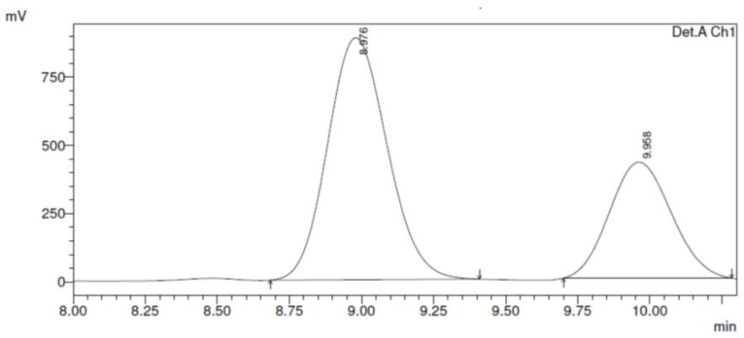

Peak#	Ret. Time	Area	Height	Area %	Height %
1	8.976	12753892	885700	66.795	67.601
2	9.958	6340112	424493	33.205	32.399
Total		19094005	1310193	100.000	100.000

Figure S12. HPLC traces of entry 7 – Table 2

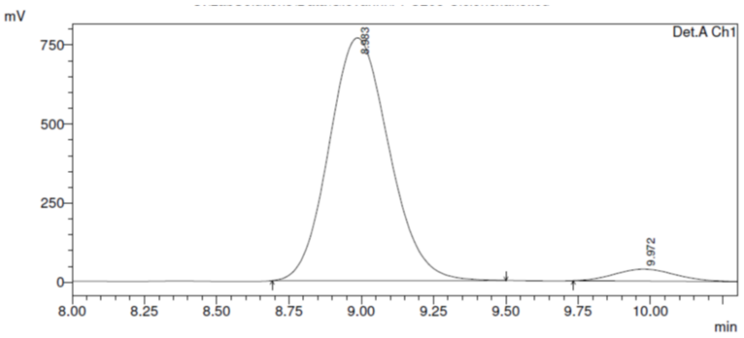

Peak#	Ret. Time	Area	Height	Area %	Height %
1	8.983	11053960	766954	95.269	95.301
2	9.972	548941	37817	4.731	4.699
Total		11602901	804770	100.000	100.000

Figure S13. HPLC traces of entry 8 – Table 2

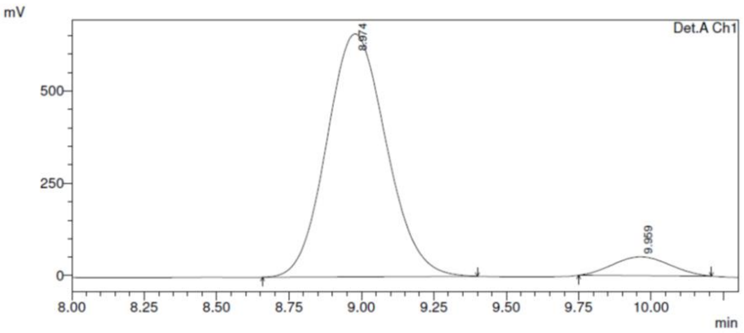

Peak#	Ret. Time	Area	Height	Area %	Height %
1	8.974	9428231	658575	93.131	92.823
2	9.959	695355	50922	6.869	7.177
Total		10123586	709497	100.000	100.000

Figure S14. HPLC traces of entry 9 – Table 2

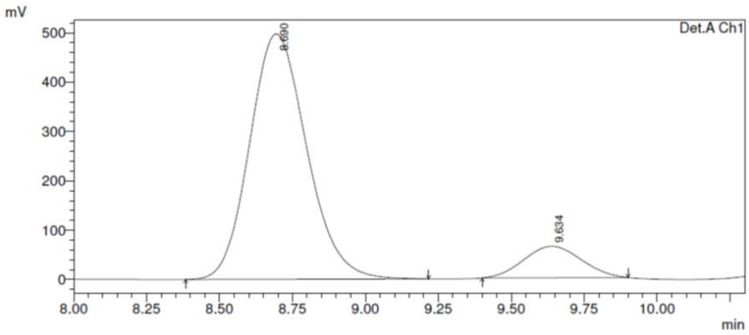

Peak#	Ret. Time	Area	Height	Area %	Height %
1	8.690	6763796	497175	88.523	88.614
2	9.634	876957	63882	11.477	11.386
Total		7640753	561057	100.000	100.000

Figure S15. HPLC traces of entry 10 – Table 2

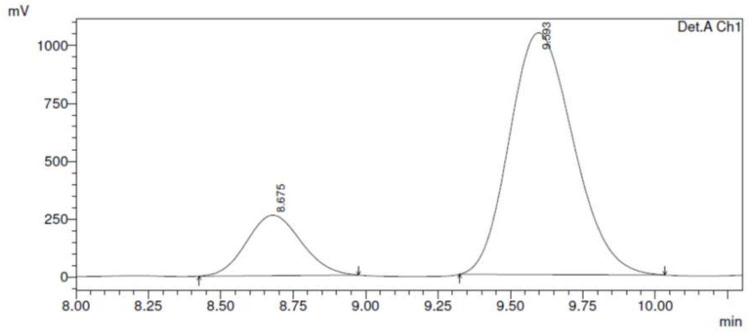

Peak#	Ret. Time	Area	Height	Area %	Height %
1	8.675	3434573	260697	17.633	19.973
2	9.593	16043178	1044573	82.367	80.027
Total		19477751	1305269	100.000	100.000

Figure S16. HPLC traces of entry 11 – Table 2

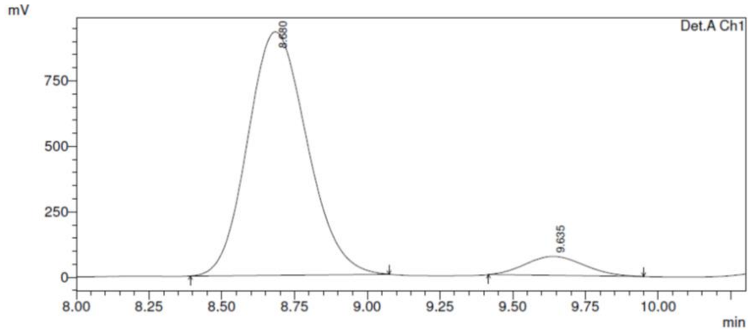

Peak#	Ret. Time	Area	Height	Area %	Height %
1	8.680	13169954	928478	92.849	92.772
2	9.635	1014258	72340	7.151	7.228
Total		14184211	1000819	100.000	100.000

Figure S17. HPLC traces of entry 12 – Table 2

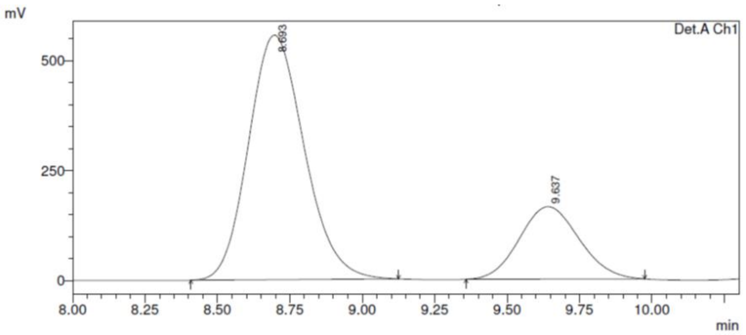

Peak#	Ret. Time	Area	Height	Area %	Height %
1	8.693	7462595	556632	75.892	77.126
2	9.637	2370629	165090	24.108	22.874
Total		9833224	721722	100.000	100.000

Figure S18. HPLC traces of entry 13 – Table 2

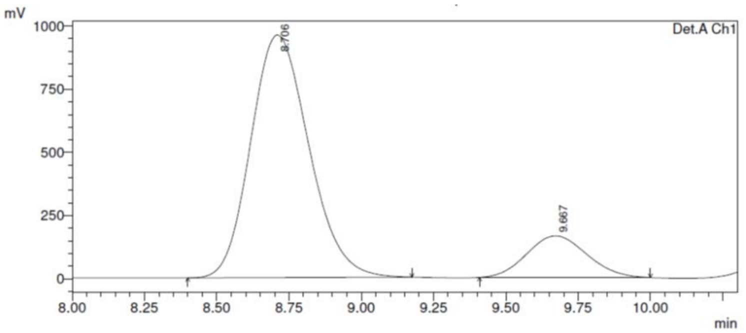

Peak#	Ret. Time	Area	Height	Area %	Height %
1	8.706	13433147	960296	84.935	85.361
2	9.667	2382591	164691	15.065	14.639
Total		15815738	1124987	100.000	100.000

Figure S19. HPLC traces of entry 14 – Table 2

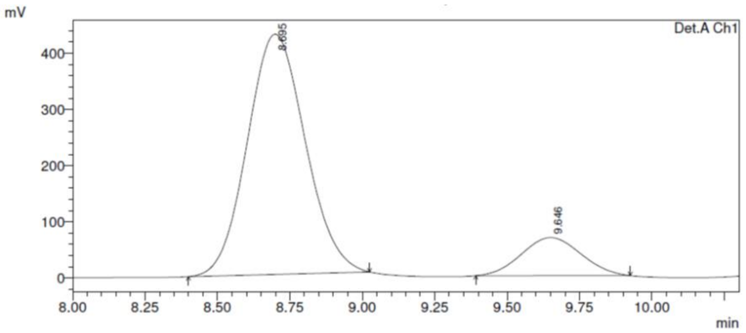

Peak#	Ret. Time	Area	Height	Area %	Height %
1	8.695	5952992	427577	86.007	86.325
2	9.646	968512	67731	13.993	13.675
Total		6921504	495308	100.000	100.000

Figure S20. HPLC traces of entry 15 – Table 2

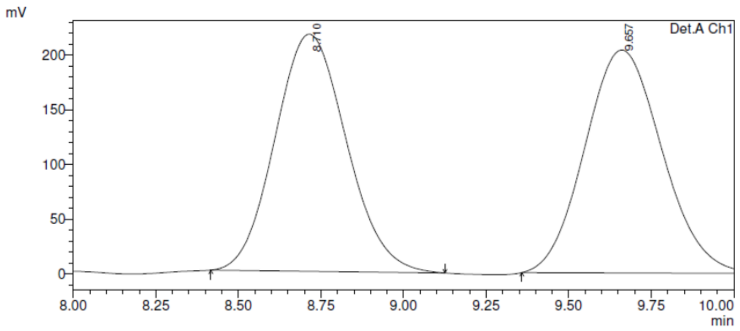

Peak#	Ret. Time	Area	Height	Area %	Height %
1	8.710	3256067	216517	49.577	51.538
2	9.657	3311633	203595	50.423	48.462
Total		6567700	420113	100.000	100.000

Figure S21. HPLC traces of entry 16 – Table 2

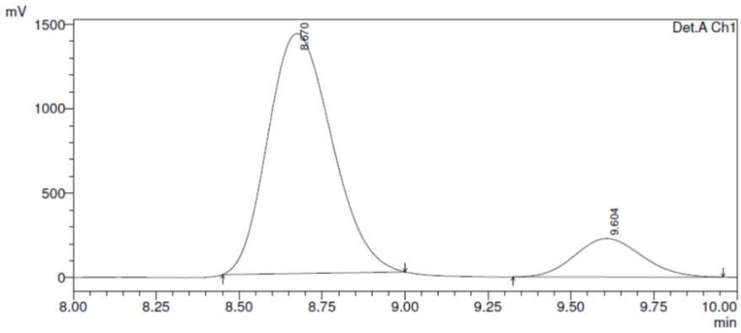

Peak#	Ret. Time	Area	Height	Area %	Height %
1	8.670	19056214	1422234	85.570	86.182
2	9.604	3213429	228043	14.430	13.818
Total		22269643	1650278	100.000	100.000

S4. Chiral HPLC traces for the final products

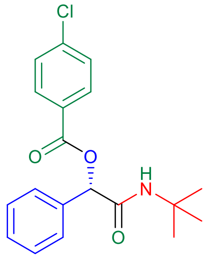

(9a)

Figure S22. HPLC traces of 9a – racemic

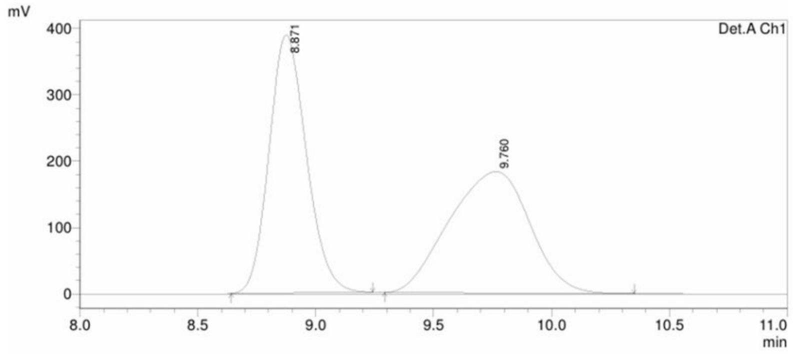

Peak#	Ret. Time	Area	Height	Area %	Height %
1	8.871	4282617	389378	49.618	68.029
2	9.760	4348514	182990	50.382	31.971
Total		8631132	572367	100.000	100.000

Figure S23. HPLC traces of 9a – asymmetric ACDC

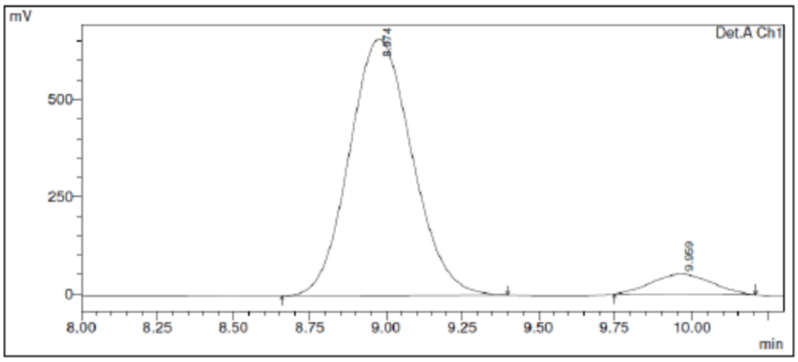

Peak#	Ret. Time	Area	Height	Area %	Height %
1	8.974	9428231	658575	93.131	92.823
2	9.959	695355	50922	6.869	7.177
Total		10123586	709497	100.000	100.000

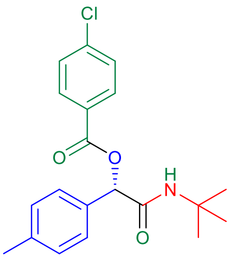

(9b)

Figure S24. HPLC traces of 9b – racemic

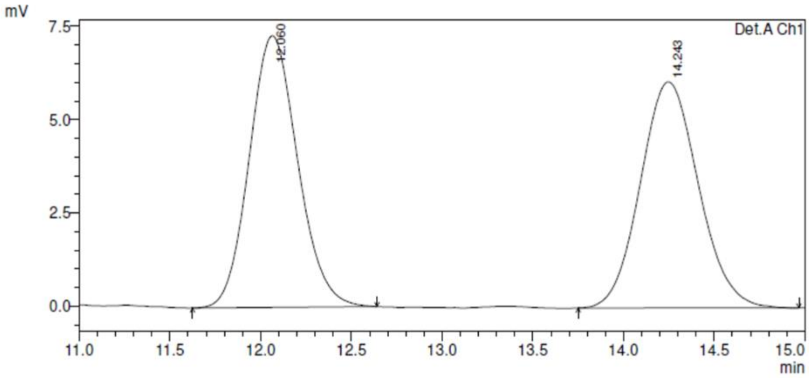

Peak#	Ret. Time	Area	Height	Area %	Height %
1	12.060	134951	7272	50.008	54.571
2	14.243	134908	6054	49.992	45.429
Total		269859	13325	100.000	100.000

Figure S25. HPLC traces of 9b – asymmetric ACDC

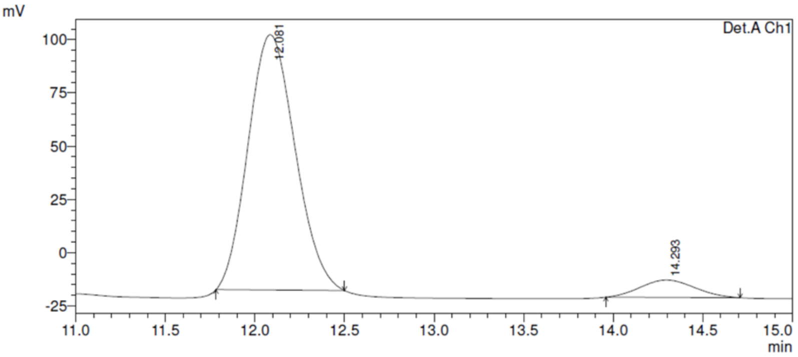

Peak#	Ret. Time	Area	Height	Area %	Height %
1	12.081	2189382	119868	92.711	93.622
2	14.293	172119	8166	7.289	6.378
Total		2361502	128034	100.000	100.000

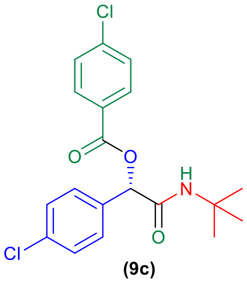

(9c)

Figure S26. HPLC traces of 9c – racemic

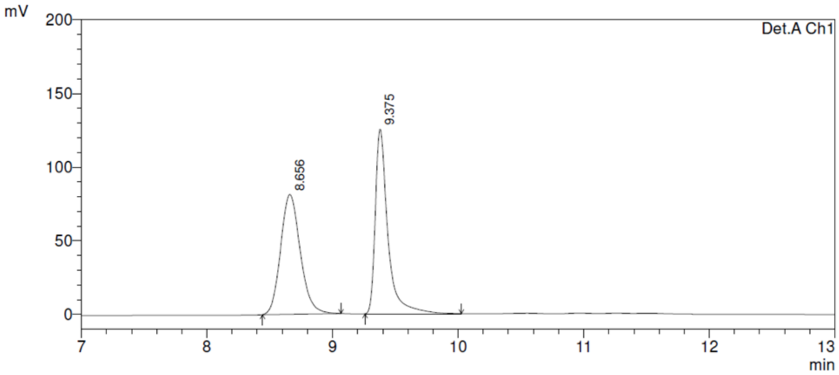

Peak#	Ret. Time	Area	Height	Area %	Height %
1	8.656	885200	81401	50.386	39.397
2	9.375	871635	125215	49.614	60.603
Total		1756835	206616	100.000	100.000

Figure S27. HPLC traces of 9c – asymmetric ACDC

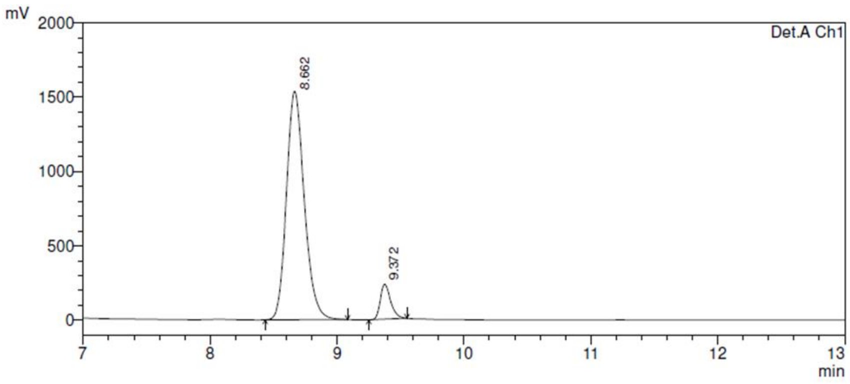

Peak#	Ret. Time	Area	Height	Area %	Height %
1	8.662	15038000	1537505	91.822	86.694
2	9.372	1339312	235973	8.178	13.306
Total		16377312	1773477	100.000	100.000

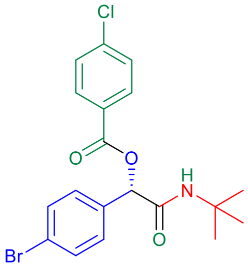

(9d)

Figure S28. HPLC traces of 9d – racemic

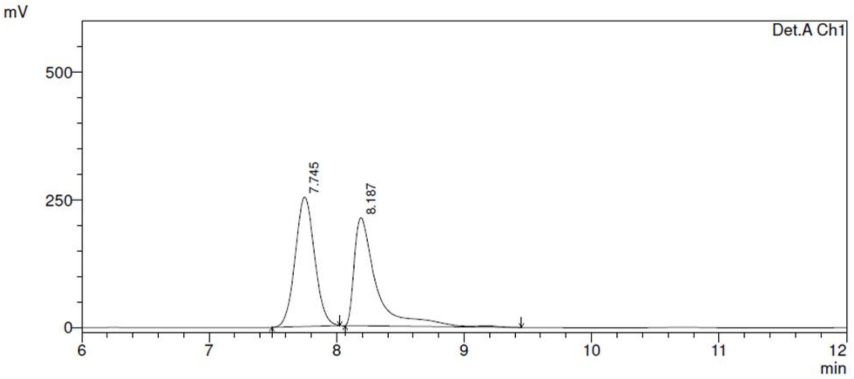

Peak#	Ret. Time	Area	Height	Area %	Height %
1	7.745	2680566	253399	51.039	54.465
2	8.187	2571393	211854	48.961	45.535
Total		5251958	465253	100.000	100.000

Figure S29. HPLC traces of 9d – asymmetric ACDC

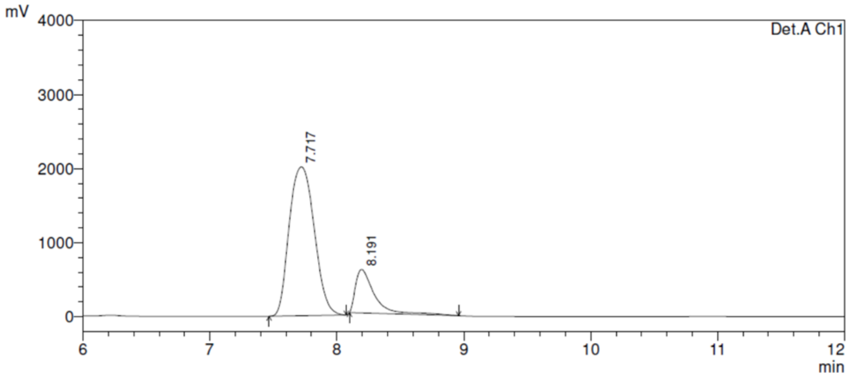

Peak#	Ret. Time	Area	Height	Area %	Height %
1	7.717	27703919	2012693	81.866	77.383
2	8.191	6136646	588243	18.134	22.617
Total		33840565	2600937	100.000	100.000

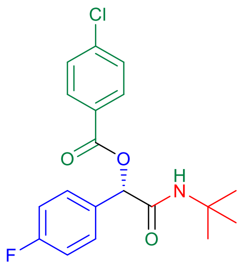

(9e)

Figure S30. HPLC traces of 9e – racemic

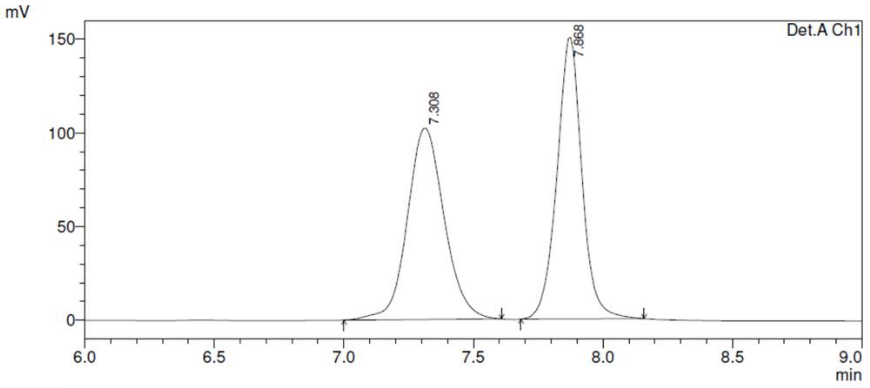

Peak#	Ret. Time	Area	Height	Area %	Height %
1	7.308	1012301	102158	50.062	40.451
2	7.868	1009780	150393	49.938	59.549
Total		2022081	252551	100.000	100.000

Figure S31. HPLC traces of 9e – asymmetric ACDC

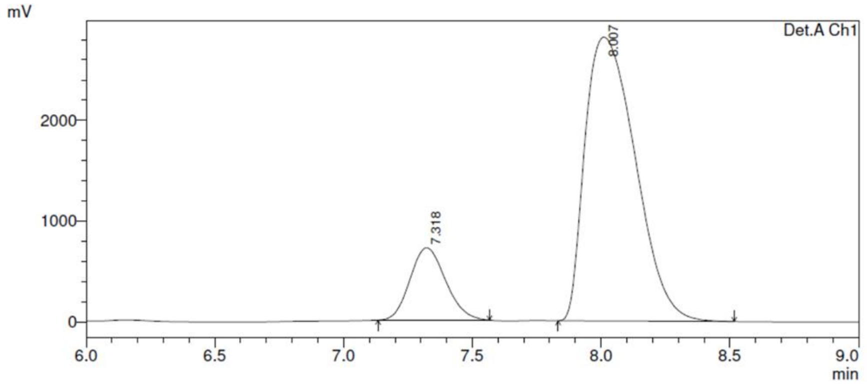

Peak#	Ret. Time	Area	Height	Area %	Height %
1	7.318	6932135	718599	15.316	20.340
2	8.007	38327844	2814345	84.684	79.660
Total		45259980	3532944	100.000	100.000

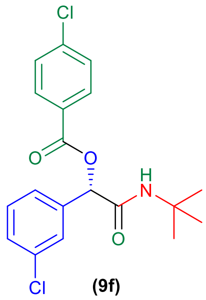

(9f)

Figure S32. HPLC traces of 9f – racemic

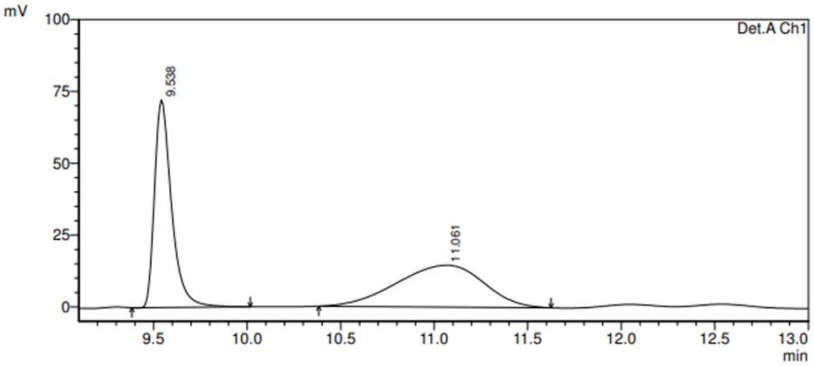

Peak#	Ret. Time	Area	Height	Area %	Height %
1	9.538	458567	72205	49.365	83.210
2	11.061	470370	14570	50.635	16.790
Total		928937	86775	100.000	100.000

Figure S33. HPLC traces of 9f – asymmetric ACDC

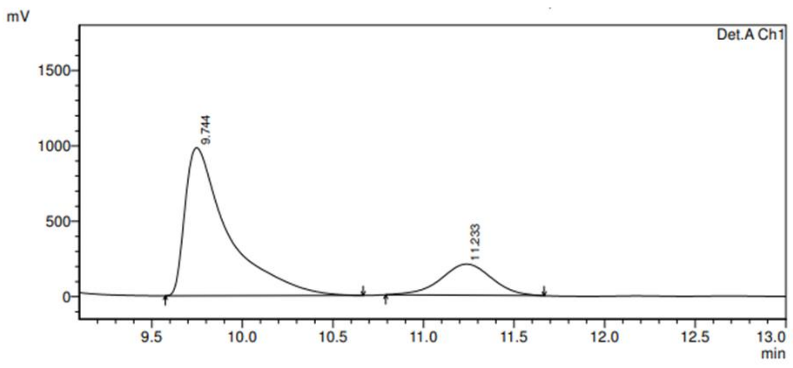

Peak#	Ret. Time	Area	Height	Area %	Height %
1	9.744	16291147	981841	80.181	82.618
2	11.233	4026909	206574	19.819	17.382
Total		20318056	1188415	100.000	100.000

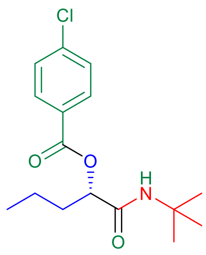

(9g)

Figure S34. HPLC traces of 9g – racemic

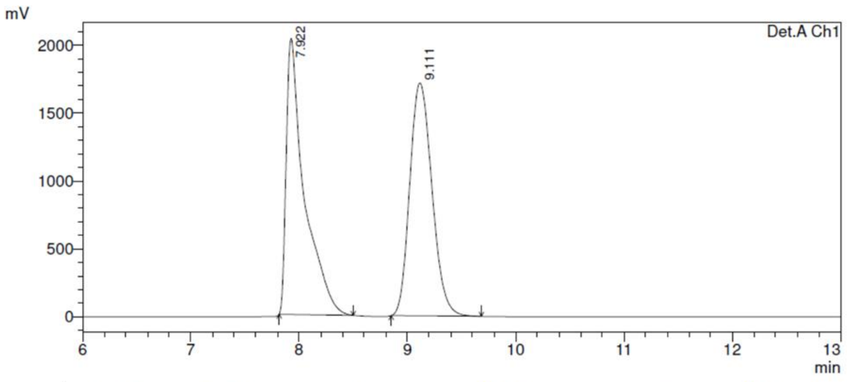

Peak#	Ret. Time	Area	Height	Area %	Height %
1	7.922	23589175	2033347	49.044	54.237
2	9.111	24508948	1715632	50.956	45.763
Total		48098123	3748979	100.000	100.000

Figure S35. HPLC traces of 9g – asymmetric ACDC

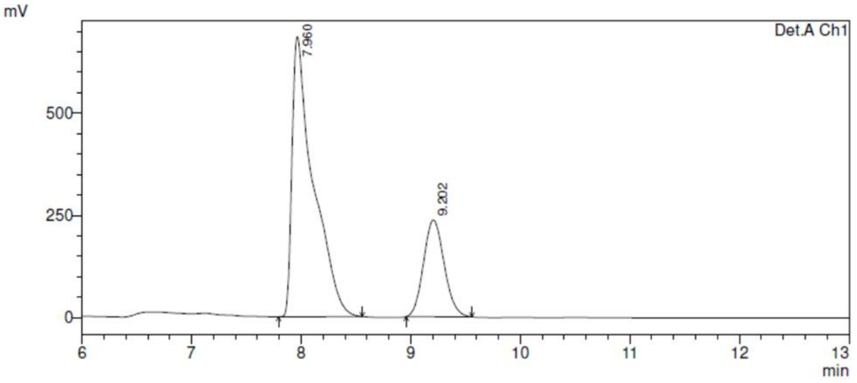

Peak#	Ret. Time	Area	Height	Area %	Height %
1	7.960	9247932	685505	74.880	74.333
2	9.202	3102446	236702	25.120	25.667
Total		12350379	922207	100.000	100.000

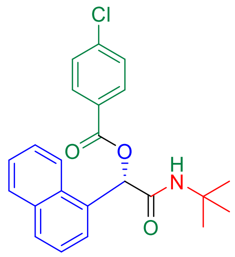

(9h)

Figure S36. HPLC traces of 9h – racemic

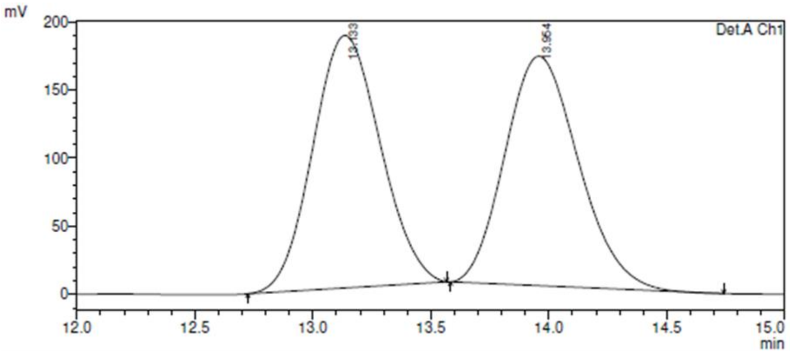

Peak#	Ret. Time	Area	Height	Area %	Height %
1	13.133	3699094	185503	50.290	52.398
2	13.954	3656480	168524	49.710	47.602
Total		7355574	354028	100.000	100.000

Figure S37. HPLC traces of 9h – asymmetric ACDC

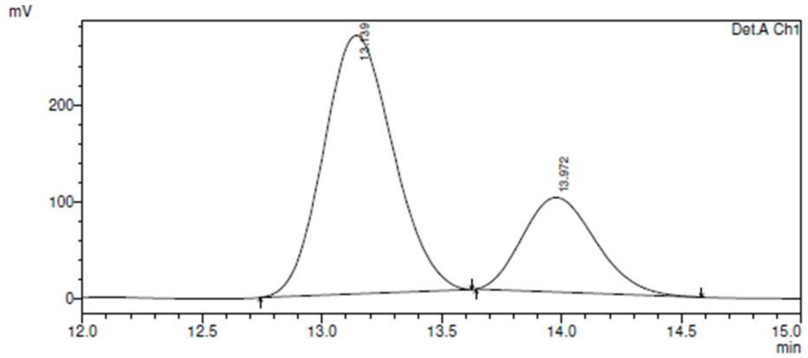

Peak#	Ret. Time	Area	Height	Area %	Height %
1	13.139	5378807	266167	72.245	73.247
2	13.972	2066388	97214	27.755	26.753
Total		7445195	363381	100.000	100.000

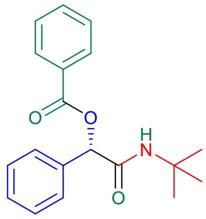

(9i)

Figure S38. HPLC traces of 9i – racemic

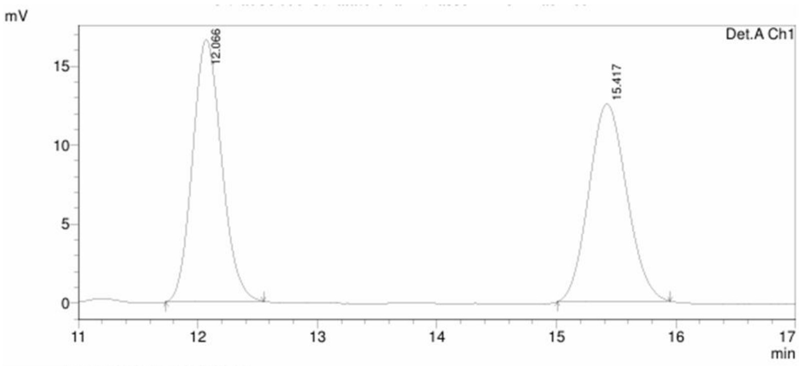

Peak#	Ret. Time	Area	Height	Area %	Height %
1	12.066	280213	16558	50.464	57.003
2	15.417	275059	12490	49.536	42.997
Total		555272	29049	100.000	100.000

Figure S39. HPLC traces of 9i – asymmetric ACDC

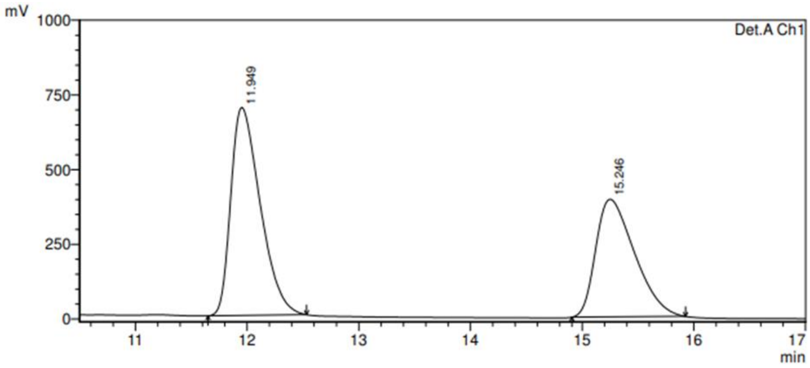

Peak#	Ret. Time	Area	Height	Area %	Height %
1	11.949	12753402	695806	57.267	63.863
2	15.246	9516491	393718	42.733	36.137
Total		22269893	1089523	100.000	100.000

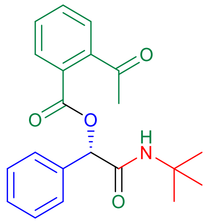

(9j)

Figure S40. HPLC traces of 9j – racemic

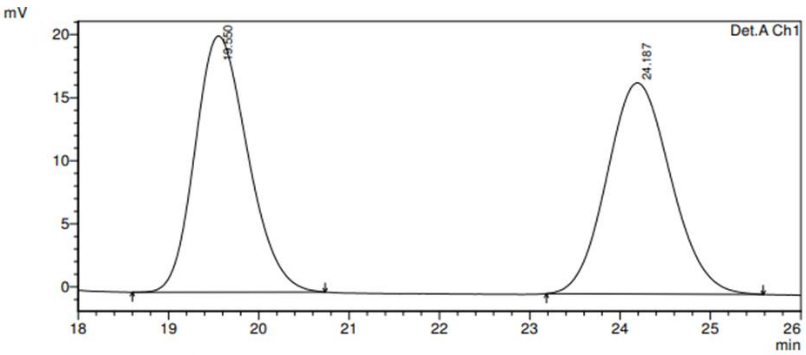

Detector A Ch1 254nm

Peak#	Ret. Time	Area	Height	Area %	Height %
1	19.550	823966	20310	49.946	54.824
2	24.187	825759	16736	50.054	45.176
Total		1649725	37046	100.000	100.000

Figure S41. HPLC traces of 9j – asymmetric ACDC

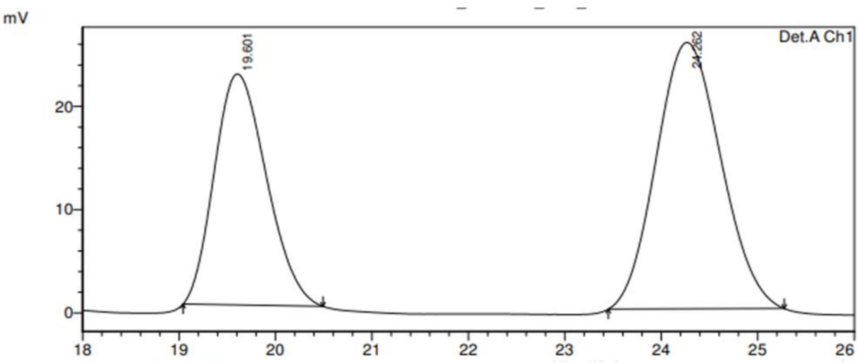

Detector A Ch1 254nm

Peak#	Ret. Time	Area	Height	Area %	Height %
1	19.601	840717	22365	41.102	46.434
2	24.262	1204748	25801	58.898	53.566
Total		2045464	48166	100.000	100.000

S5. Kinetic study database

Figure S42. ^{1}H NMR (600 MHz, C_6D_6) of the reaction between the carboxylic acid and the aldehyde in the presence of the catalyst. From top to bottom: reaction mixture, benzoic acid (pure) and benzaldehyde (pure).

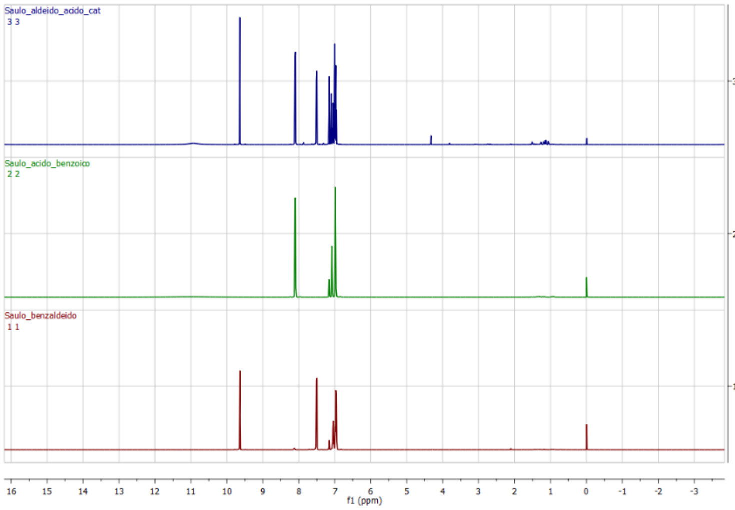

Figure S43. ^{1}H NMR (600 MHz, C_6D_6) of the reaction between the carboxylic acid and the isocyanide in the presence of the catalyst. From top to bottom: reaction mixture, tert-butyl isocyanide (pure) and benzoic acid (pure).

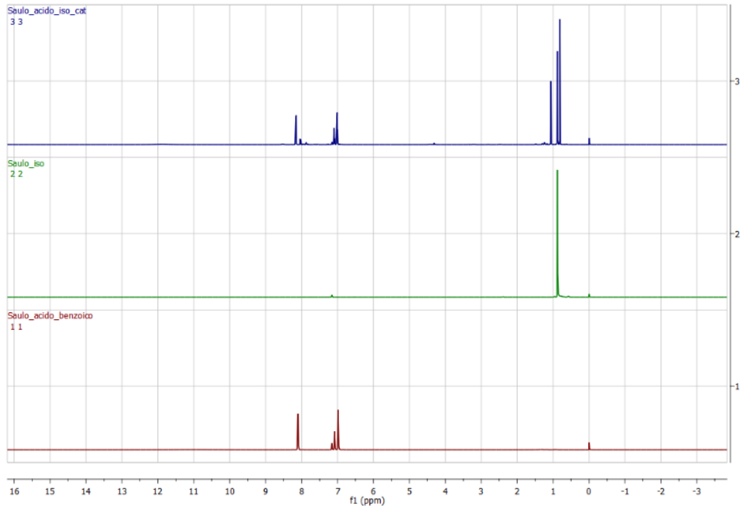

Figure S44. ^{1}H NMR (600 MHz, C_6D_6) of the reaction between the aldehyde and the isocyanide in the presence of the catalyst. From top to bottom: reaction mixture, tert-butyl isocyanide (pure) and benzaldehyde (pure).

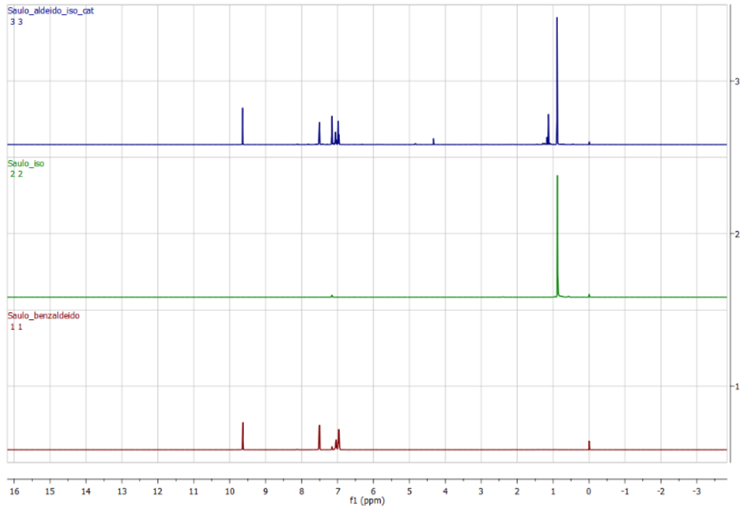

Figure S45. Differential model predictions based on a sequential optimization procedure. The continuous lines correspond to the model prediction of the mole fraction of all components in the reaction system.

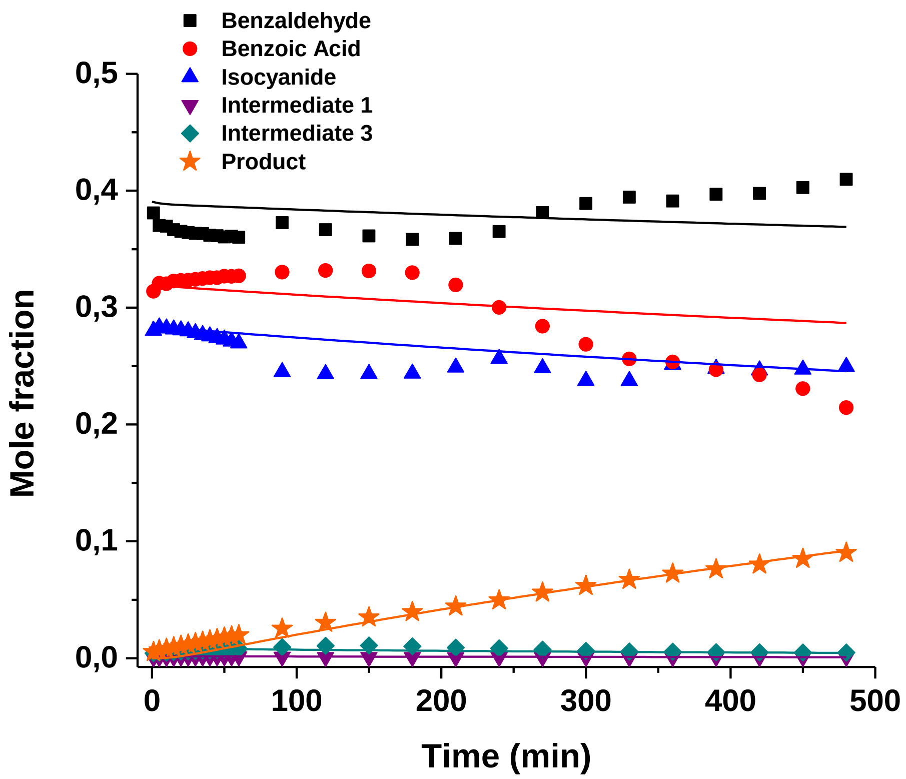

Figure S46. Carboxylic acid consumption as monitored by ^{1}H NMR.

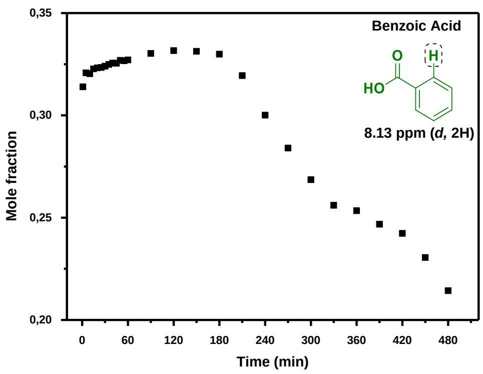

Figure S47. Isocyanide consumption as monitored by ^{1}H NMR.

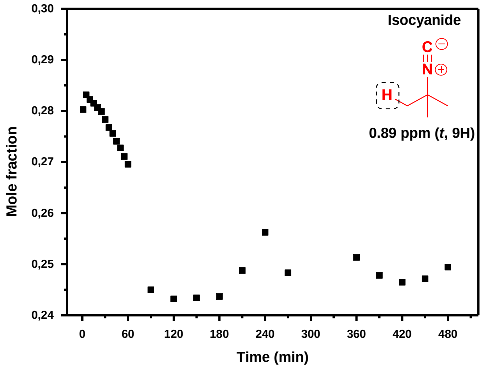

Figure S48. Aldehyde consumption as monitored by ^{1}H NMR.

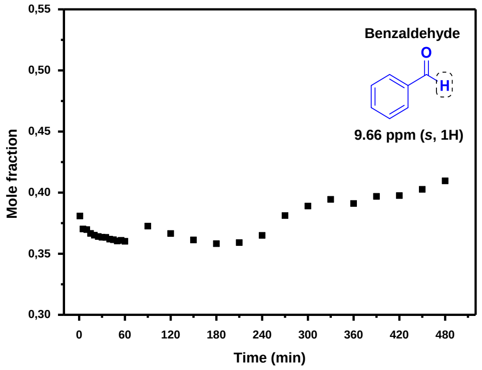

Figure S49. Int. 1 formation and consumption as monitored by ^{1}H NMR.

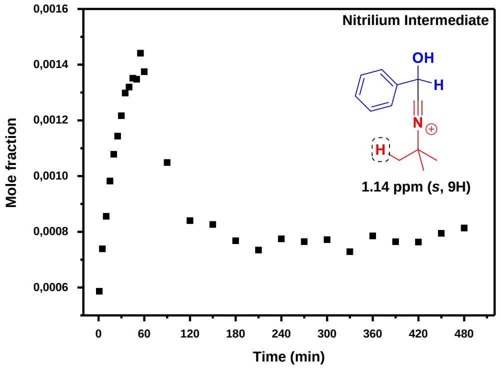

Figure S50. Int. 3 formation and consumption as monitored by ^{1}H NMR.

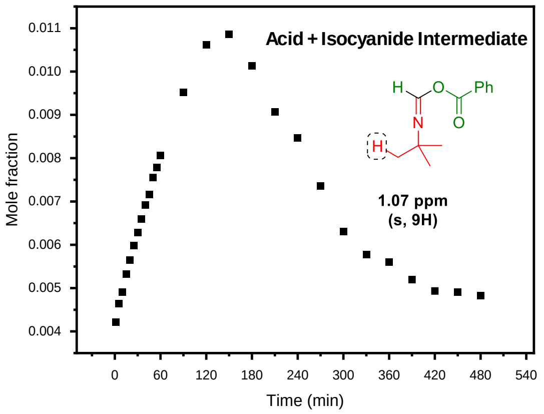

Figure S51. Product (**P**) formation as monitored by ^{1}H NMR. Note it could be monitored using two different chemical shifts with a similar behavior.

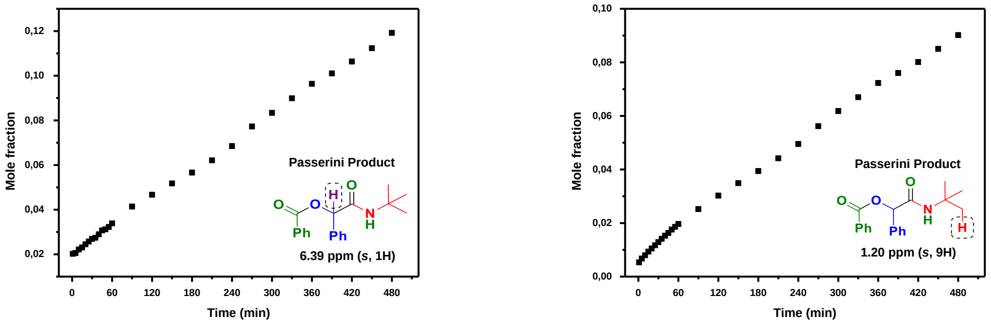

S6. Copies of IR-FT (ATR), ^{1}H and ^{13}C NMR spectra for all compounds

Figure S52. ^{1}H NMR (500 MHz, DMSO-*d6*) of catalyst (***S***)-(**1**)

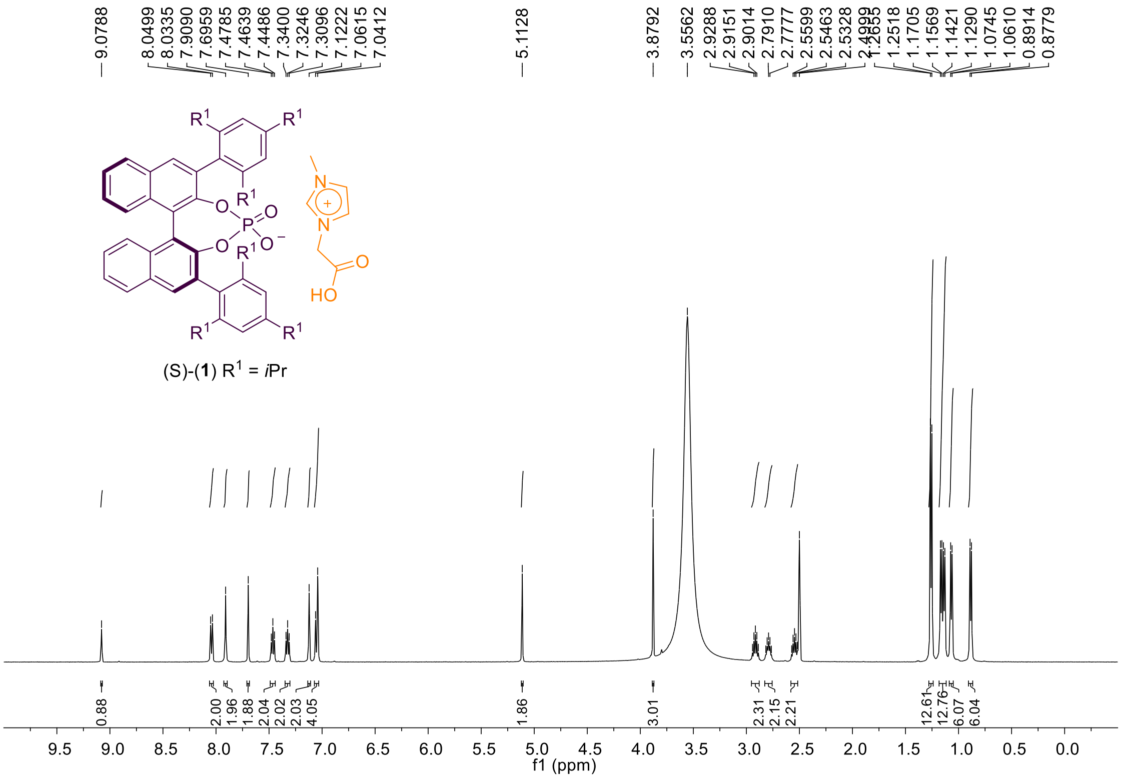

Figure S53. Expansion 1 of the ^{1}H NMR (500 MHz, DMSO-*d6*) of catalyst (***S***)-(**1**)

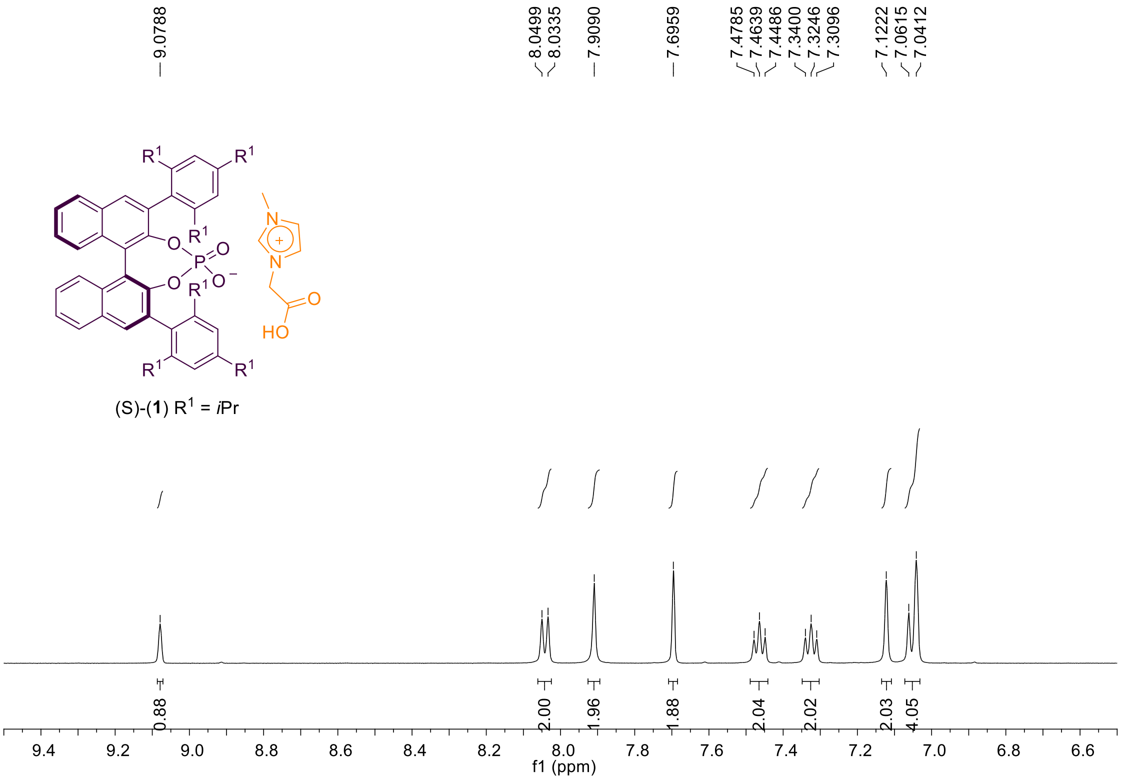

Figure S54. Expansion 2 of the ^{1}H NMR (500 MHz, DMSO-*d6*) of catalyst (***S***)-(**1**)

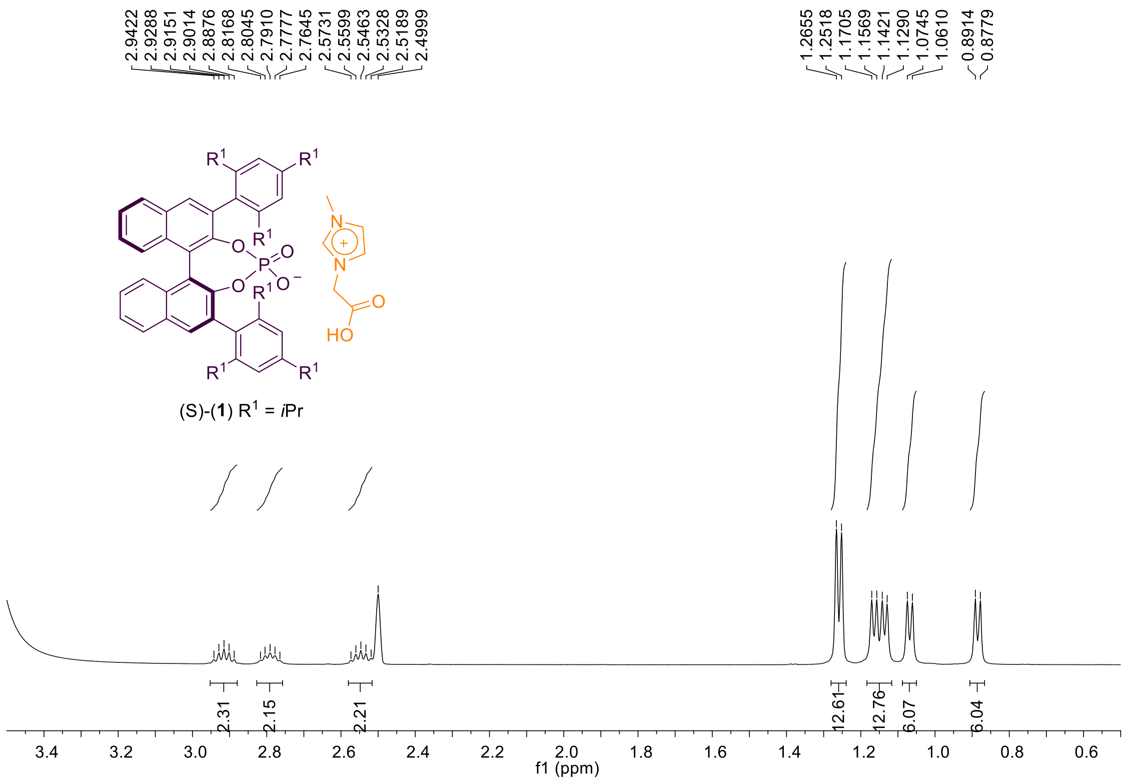

Figure S55. ^{13}C {1H} NMR (125 MHz, DMSO-*d6*) of catalyst (***S***)-(**1**)

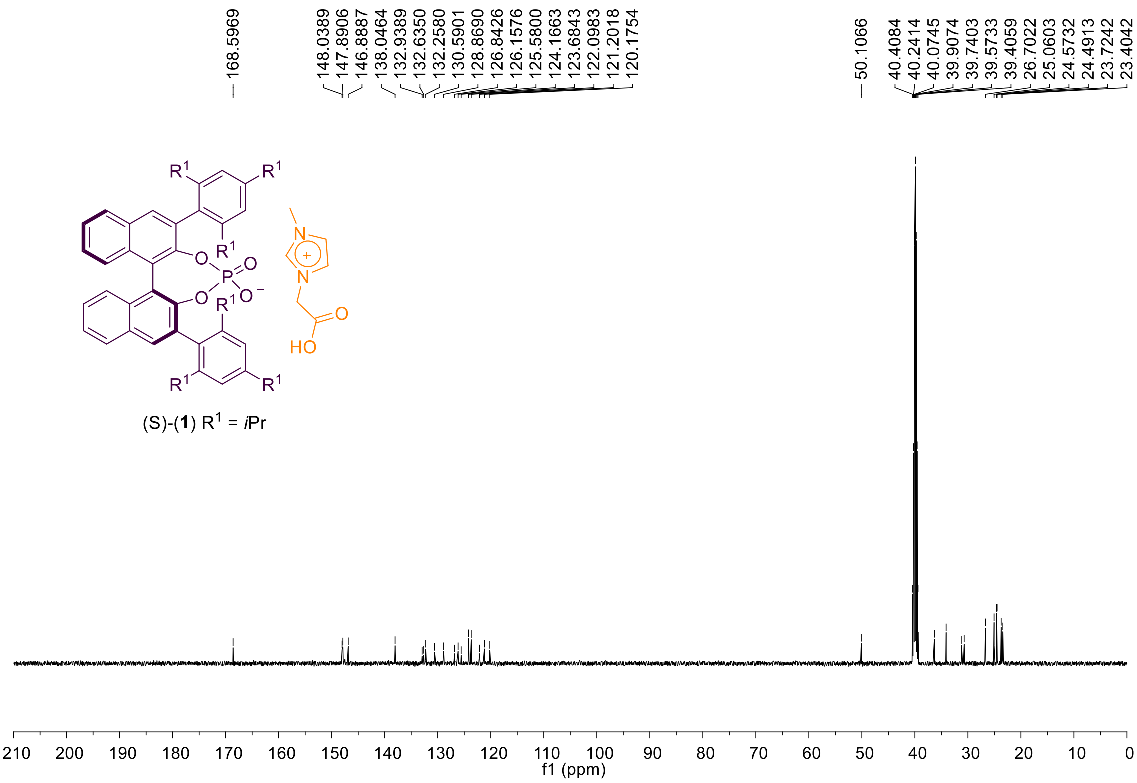

Figure S55a. ^{1}H NMR (500 MHz, DMSO-*d6*) of catalyst (*S*)-(**2**)

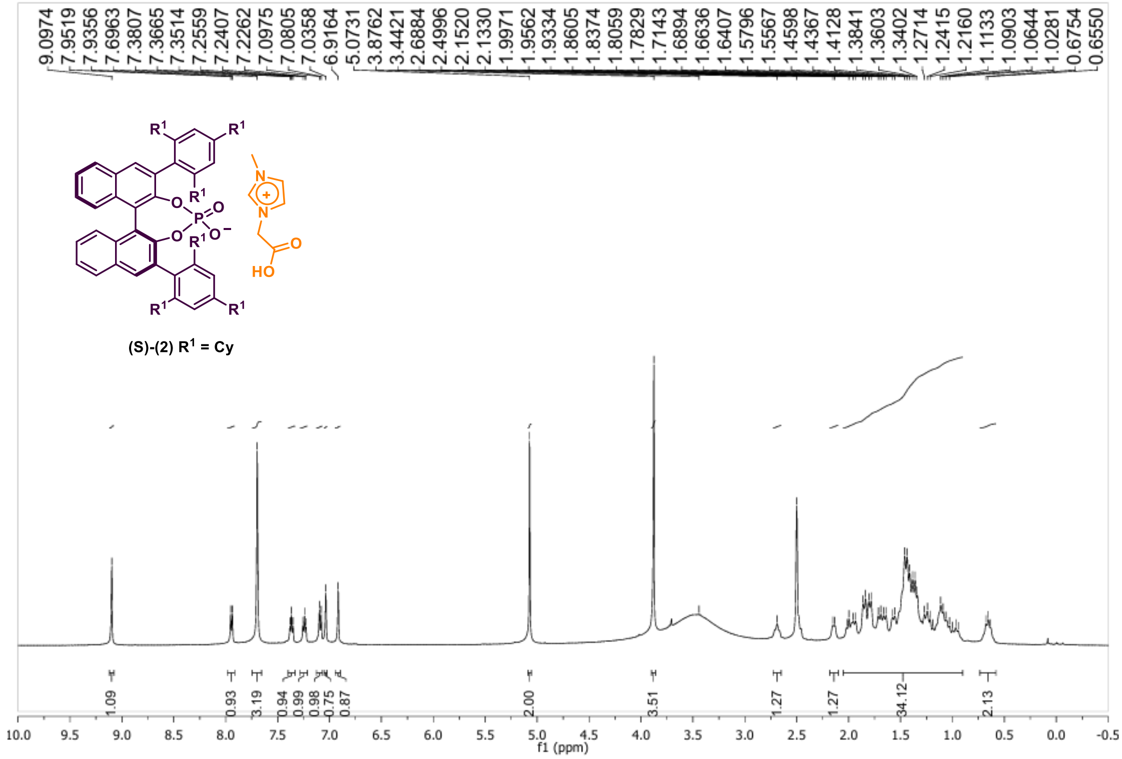

Figure S55b. ^{13}C {1H} NMR (125 MHz, DMSO-*d6*) of catalyst (*S*)-(**2**)

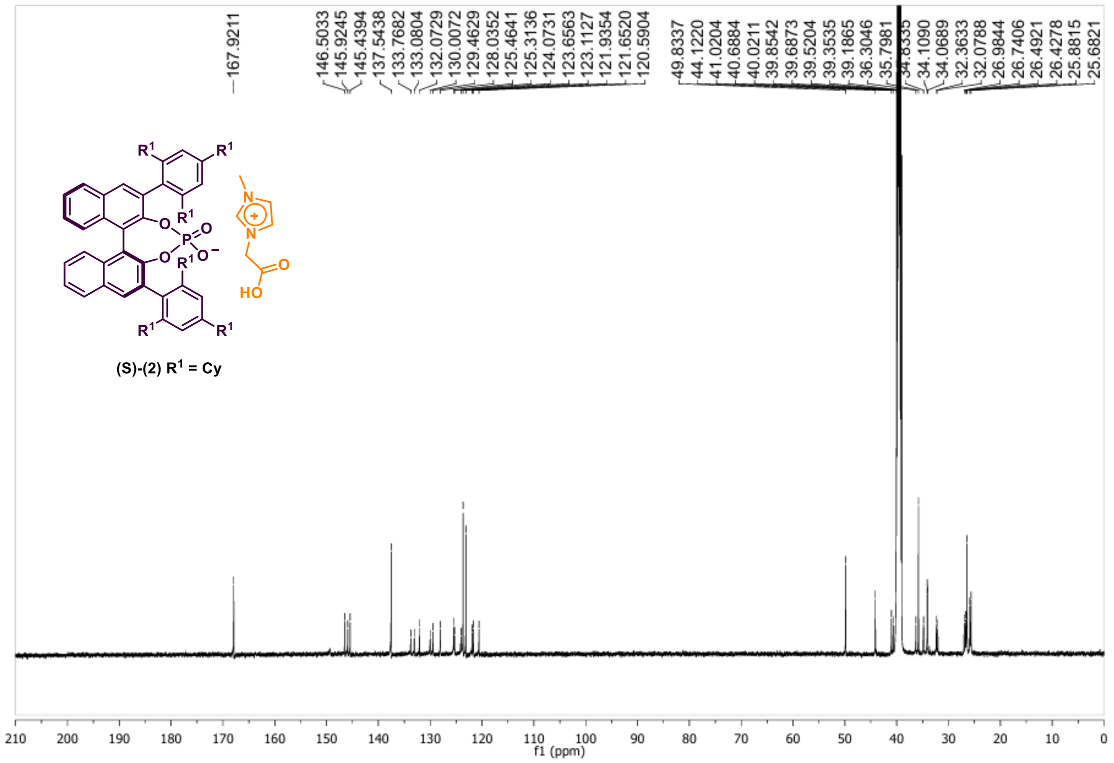

Figure S55c. ^{1}H NMR (500 MHz, $CDCl_3$ + 2 drops of DMSO-*d6*) of catalyst **(*R*)-(3).**

P.S.: Low solubility.

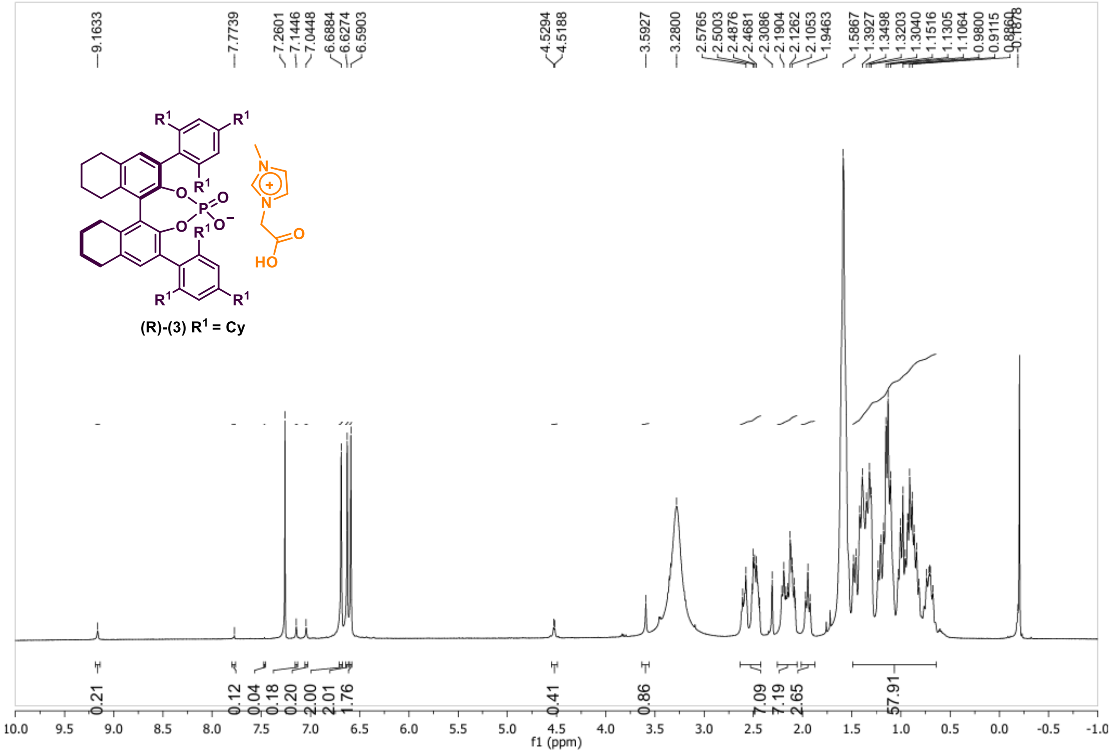

Figure S55d. $^{13}C\{1H\}$ NMR (125 MHz, $CDCl_3$ + 2 drops of DMSO-*d6*) of catalyst **(*R*)-(3)**

P.S.: Low solubility.

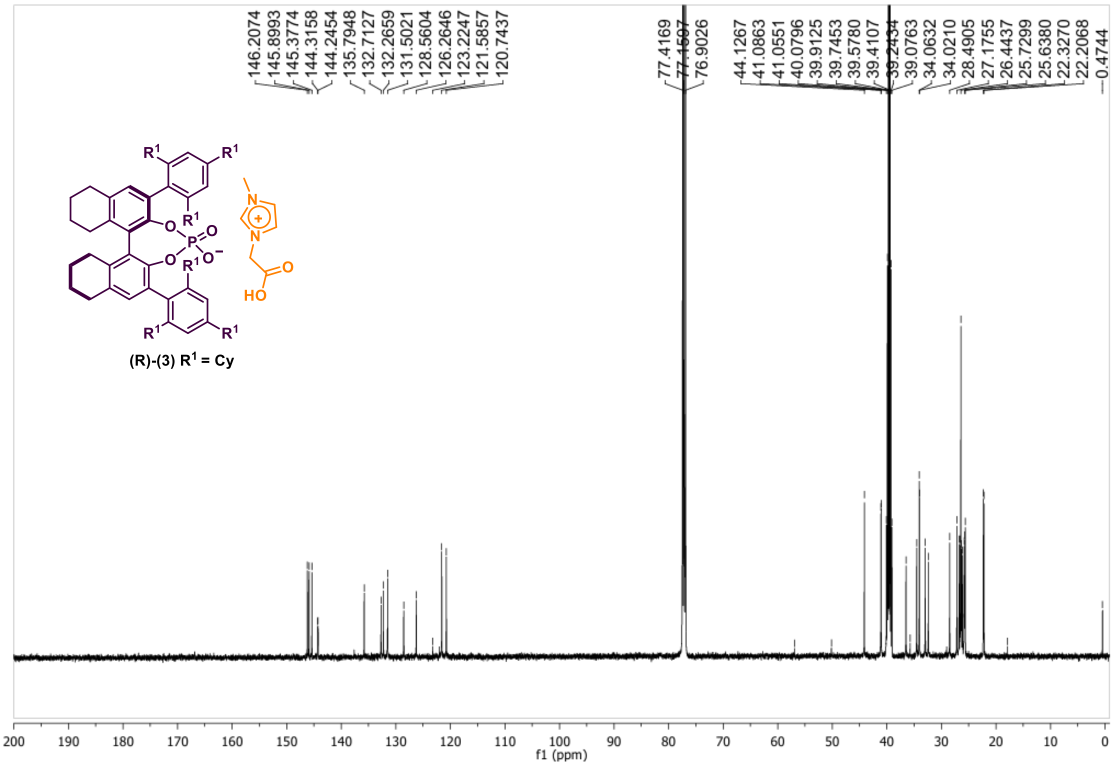

Figure S55e. ^{1}H NMR (500 MHz, DMSO-*d6*) of catalyst **(*S*)-(5)**

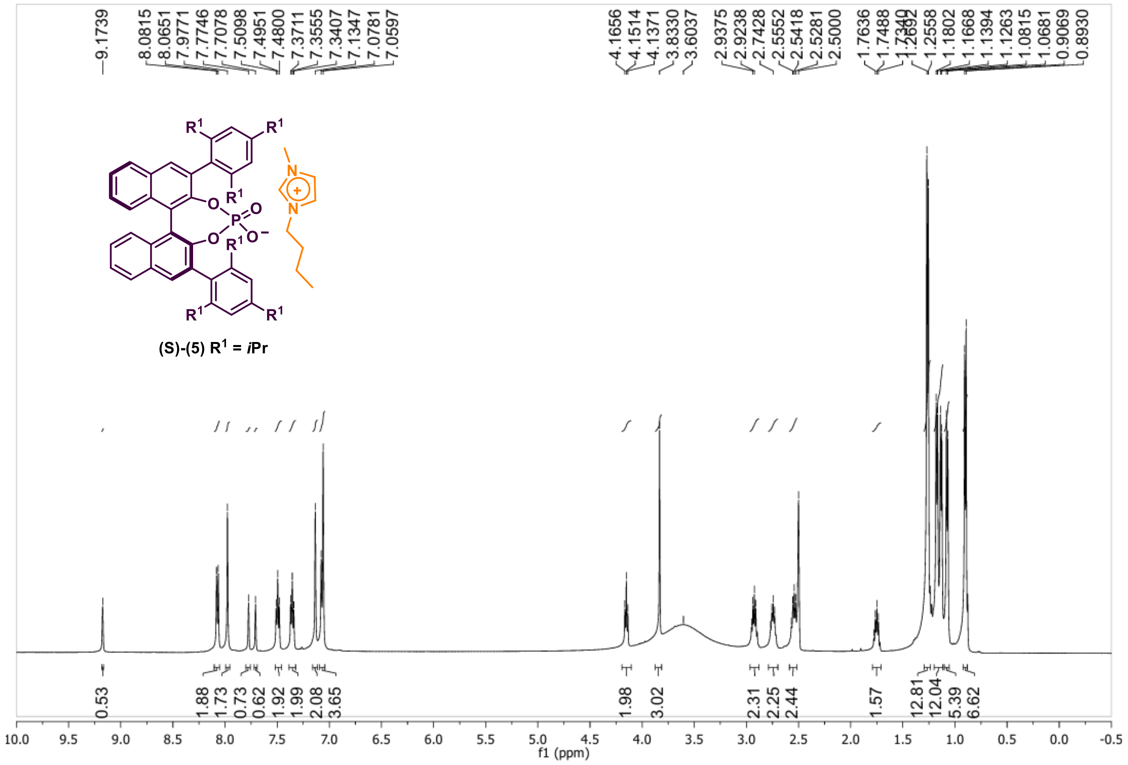

Figure S55f. ^{13}C {1H} NMR (125 MHz, DMSO-*d6*) of catalyst (***S***)-(**5**)

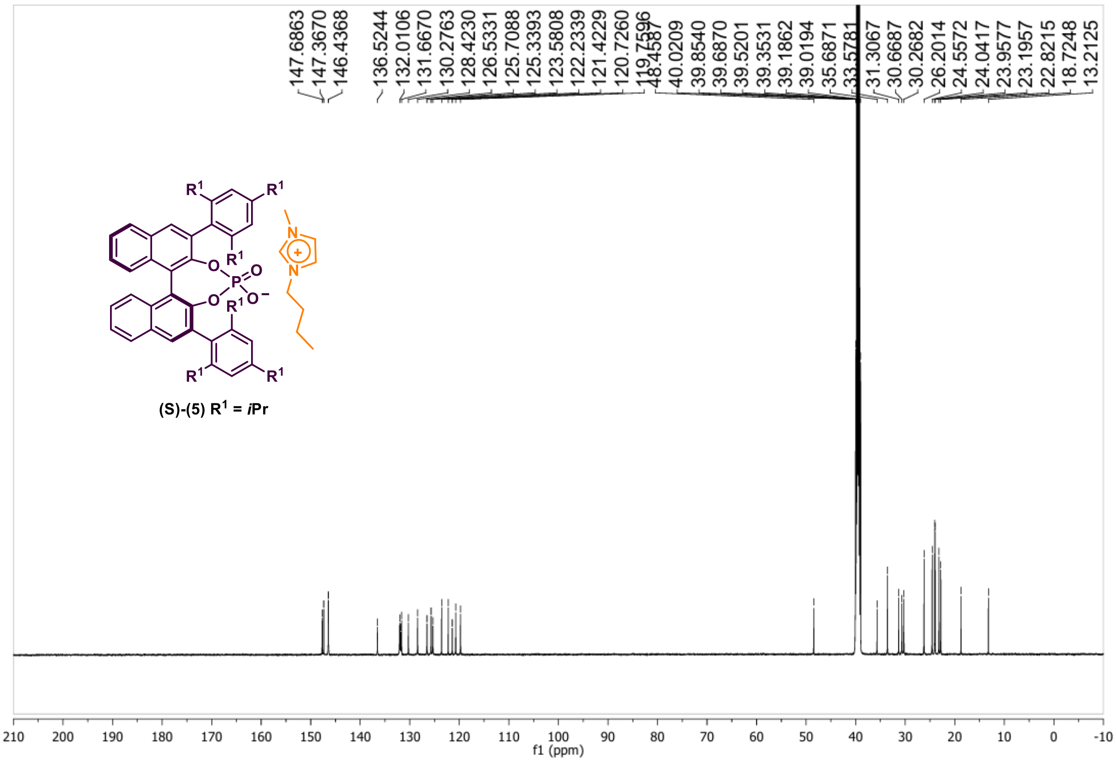

Figure S56. IR-FT of compound **9a**

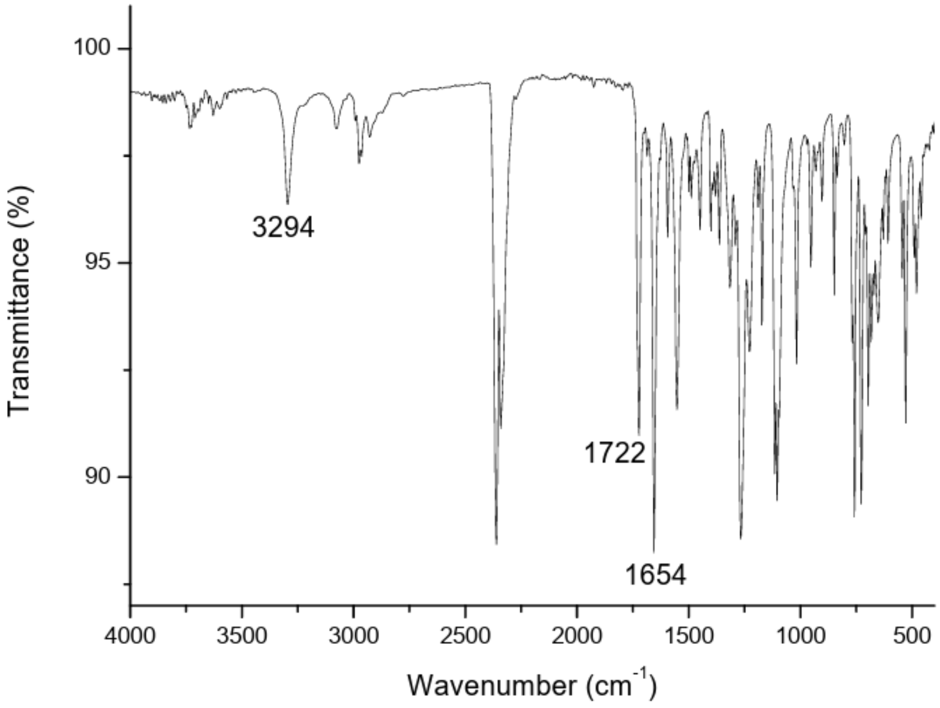

Figure S57. ^{1}H NMR (500 MHz, $CDCl_3$) of compound **9a**

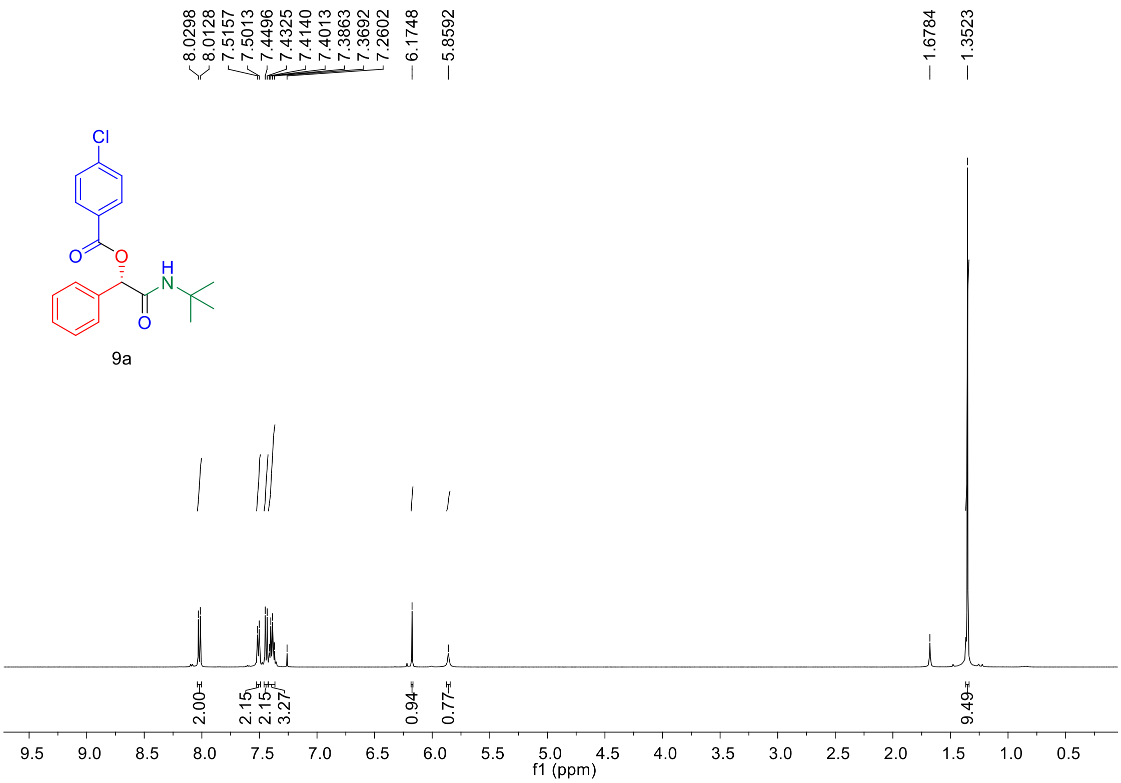

Figure S58. Expansion of the ^{1}H NMR (500 MHz, $CDCl_3$) of compound **9a**

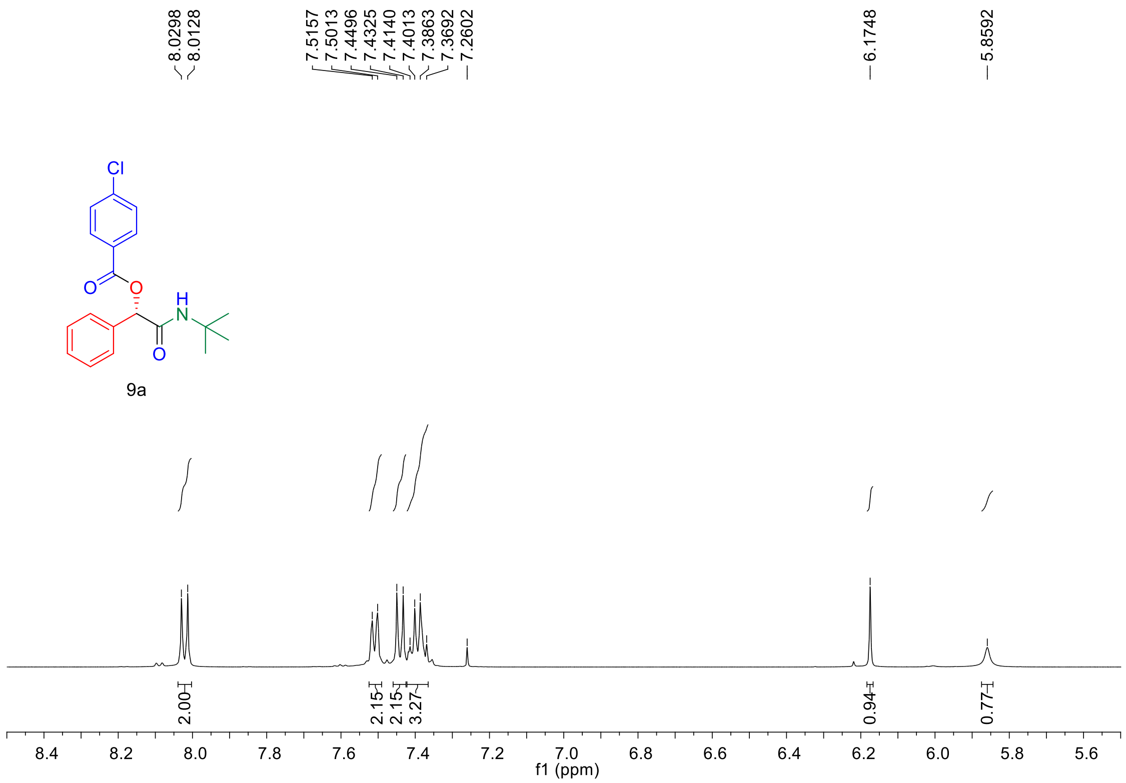

Figure S59. ^{13}C {1H} NMR (125 MHz, $CDCl_3$) of compound **9a**

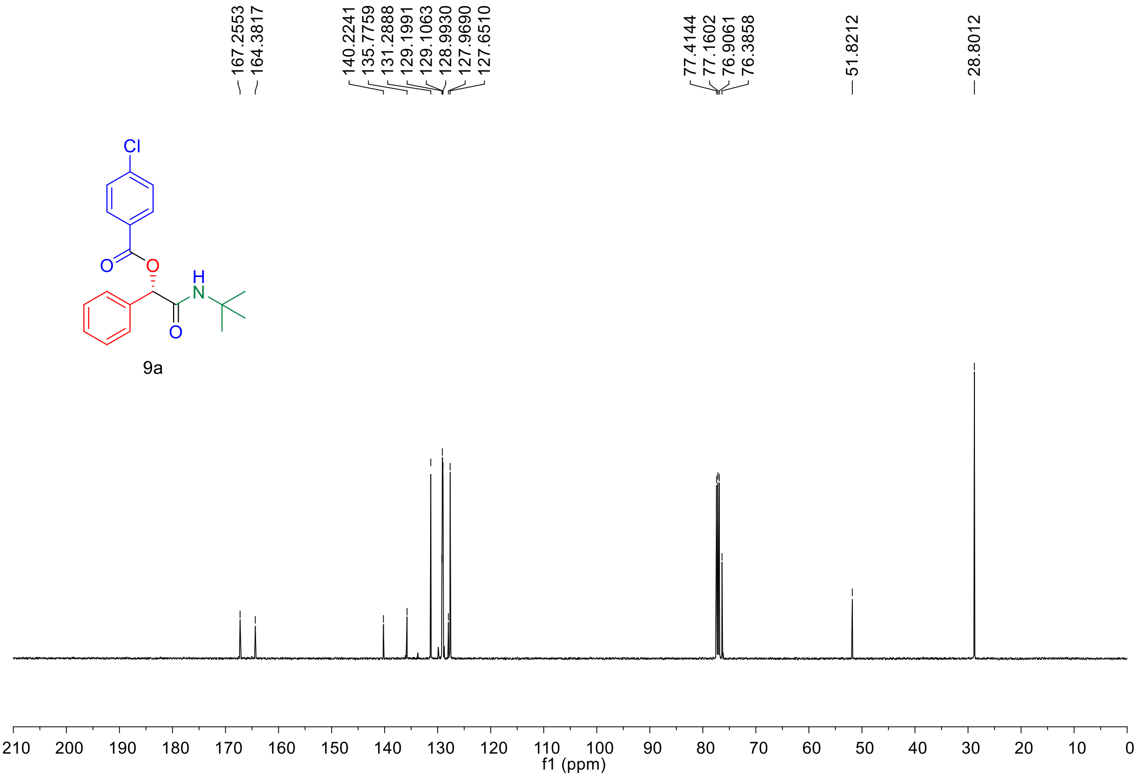

Figure S60. IR-FT of compound **9b**

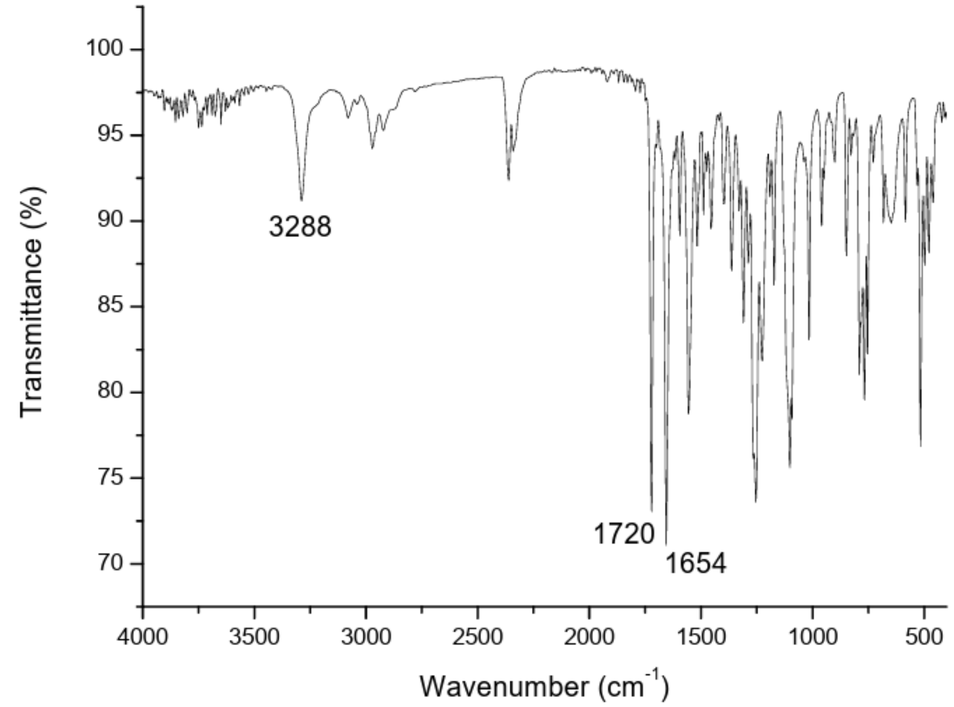

Figure S61. ^{1}H NMR (500 MHz, $CDCl_3$) of compound **9b**

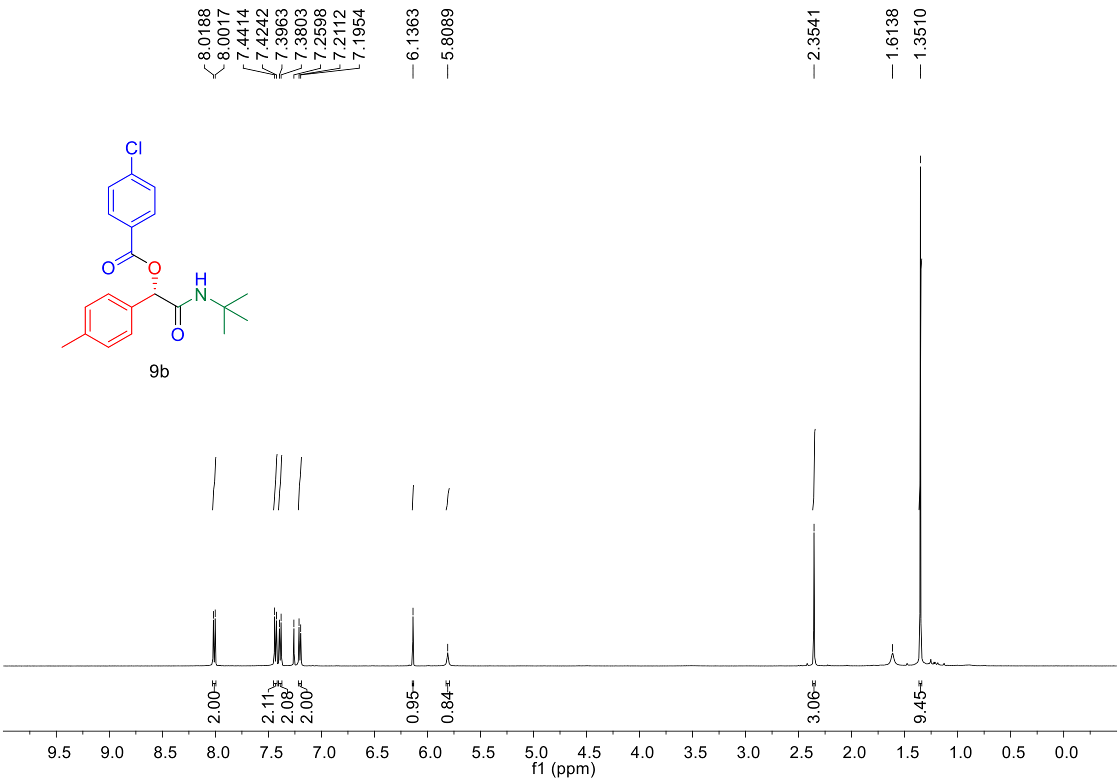

Figure S62. Expansion of the ^{1}H NMR (500 MHz, $CDCl_3$) of compound **9b**

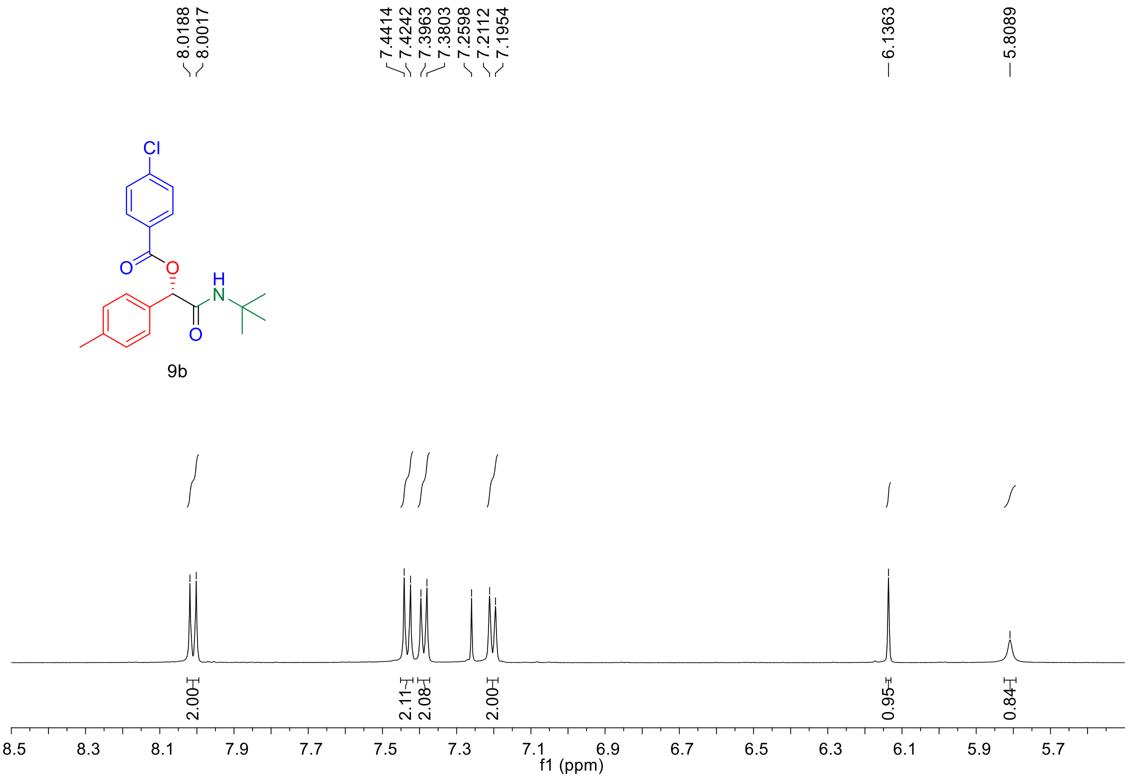

Figure S63. ^{13}C {1H} NMR (125 MHz, $CDCl_3$) of compound **9b**

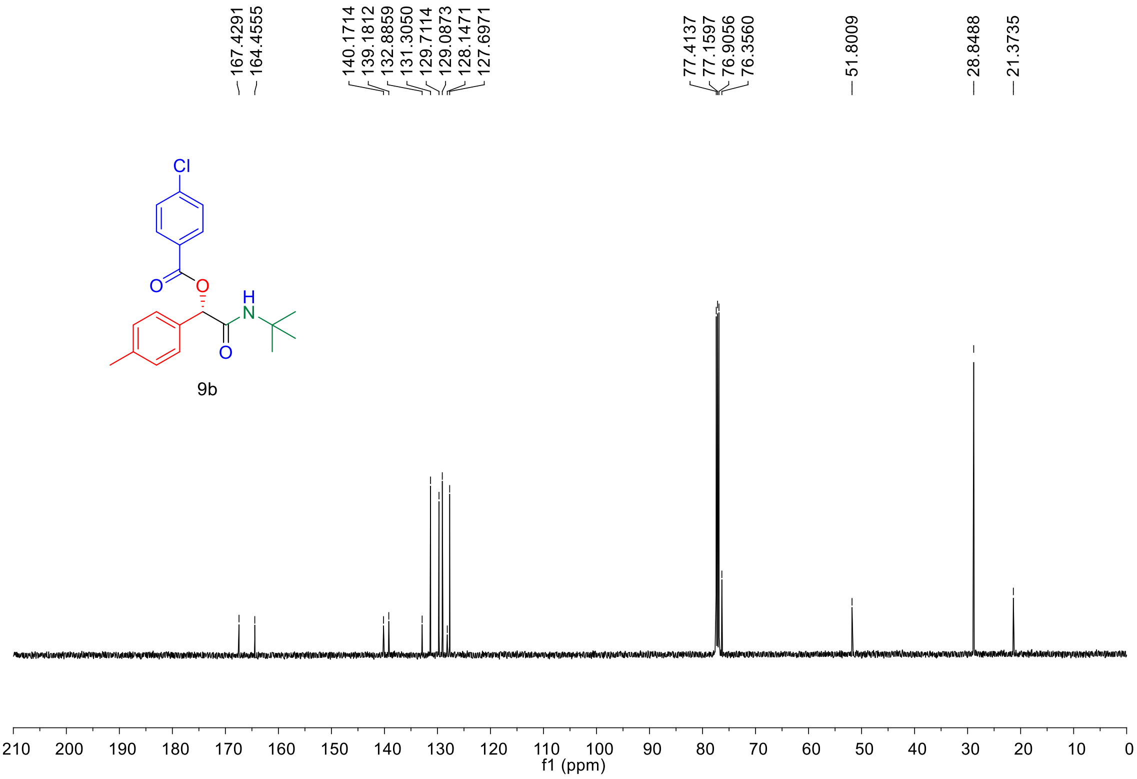

Figure S64. IR-FT of compound **9c**

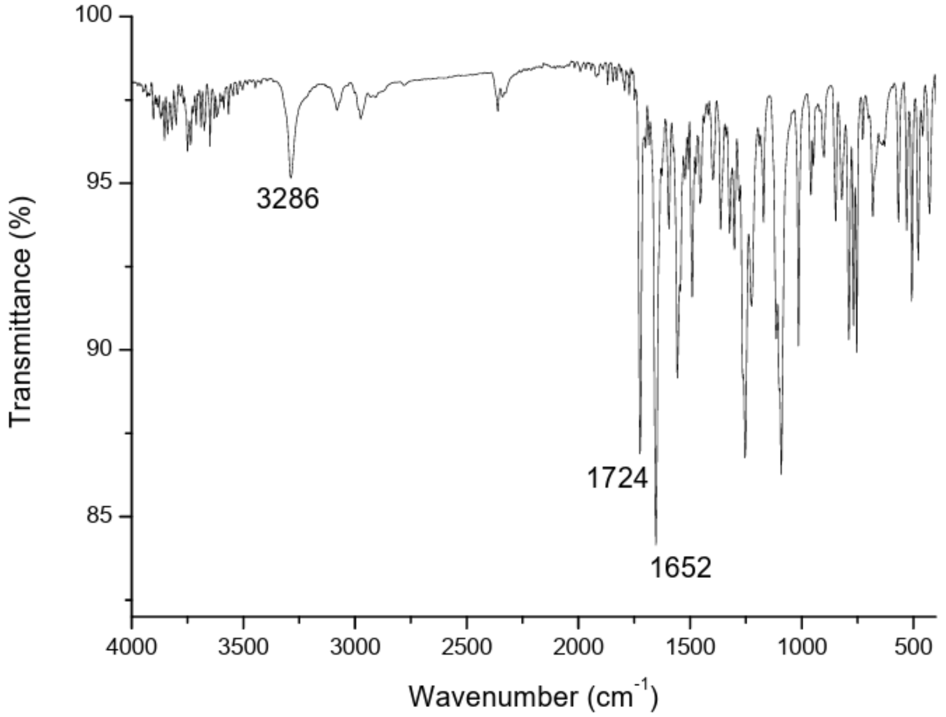

Figure S65. ^{1}H NMR (500 MHz, $CDCl_3$) of compound **9c**

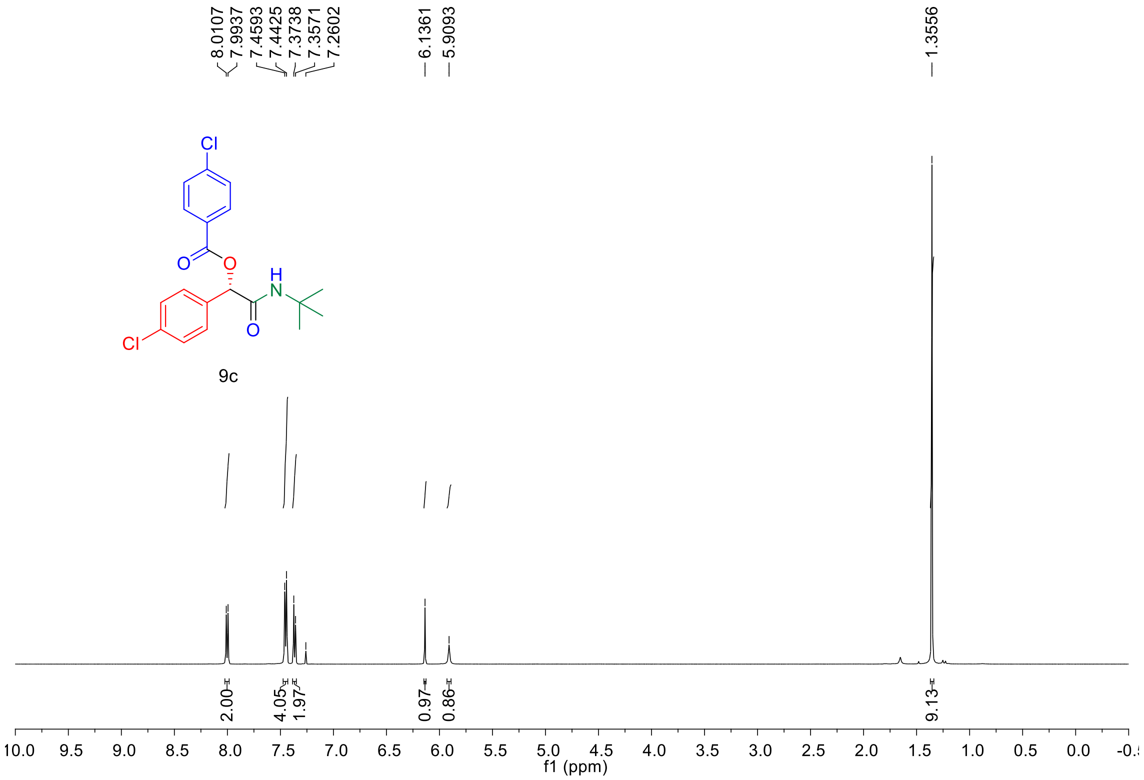

Figure S66. Expansion of the ^{1}H NMR (500 MHz, $CDCl_3$) of compound **9c**

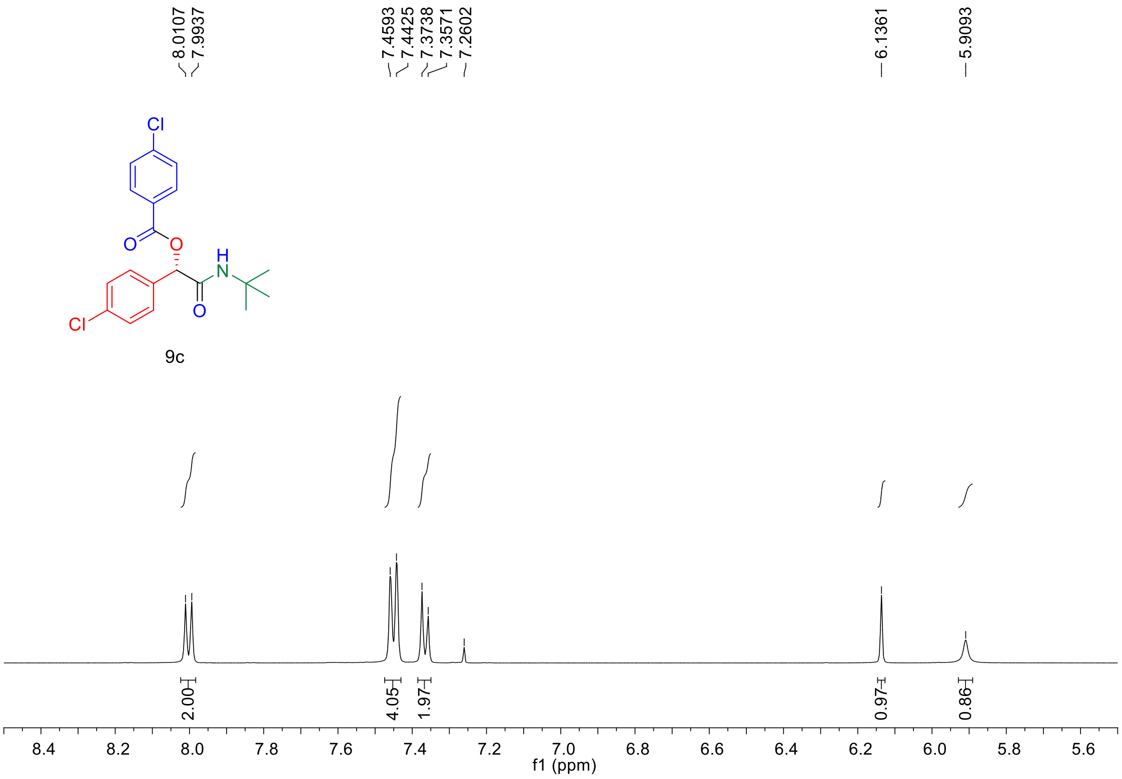

Figure S67. ^{13}C {1H} NMR (125 MHz, $CDCl_3$) of compound **9c**

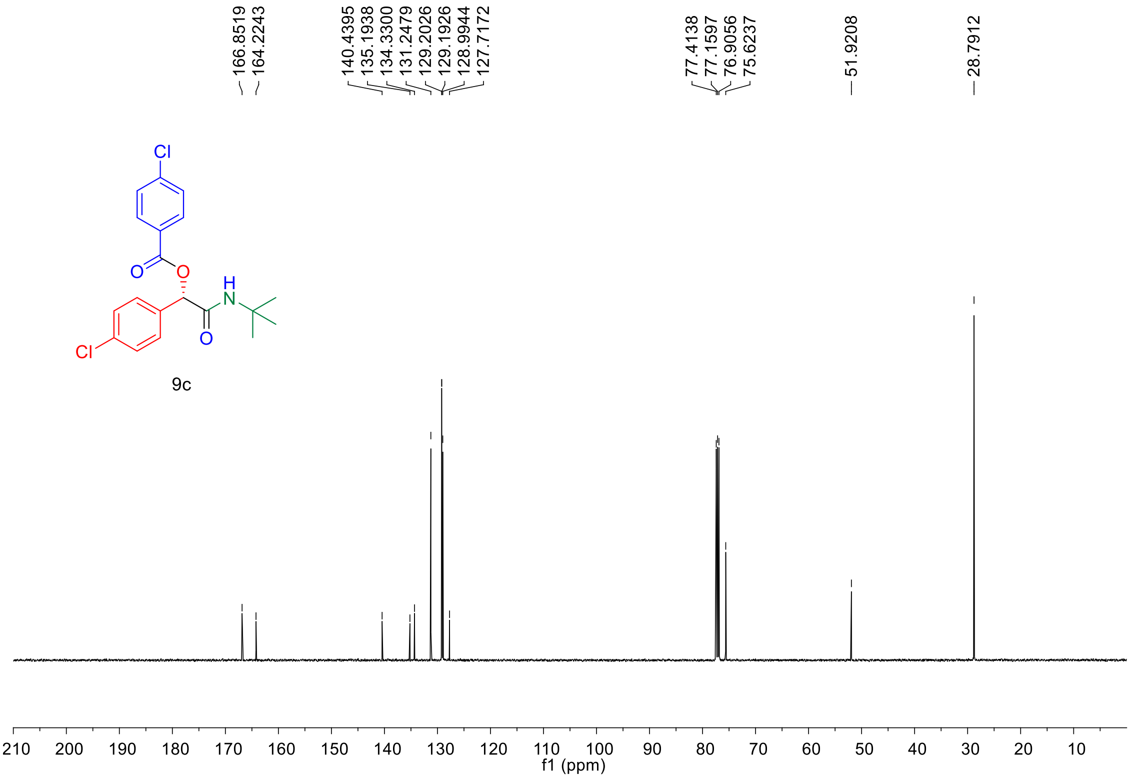

Figure S68. IR-FT of compound **9d**

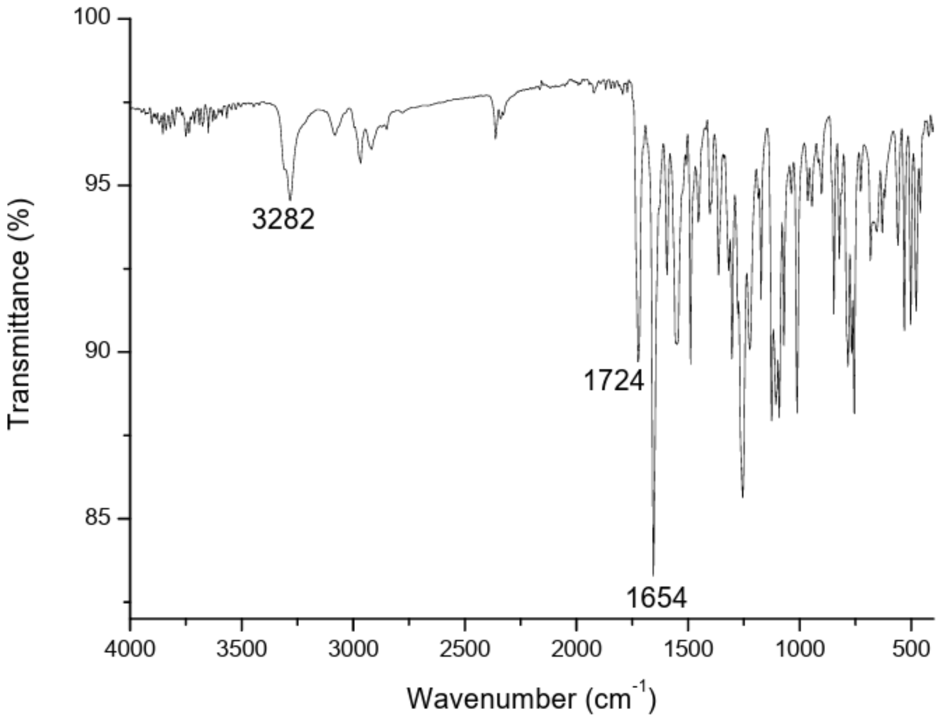

Figure S69. ^{1}H NMR (500 MHz, $CDCl_3$) of compound **9d**

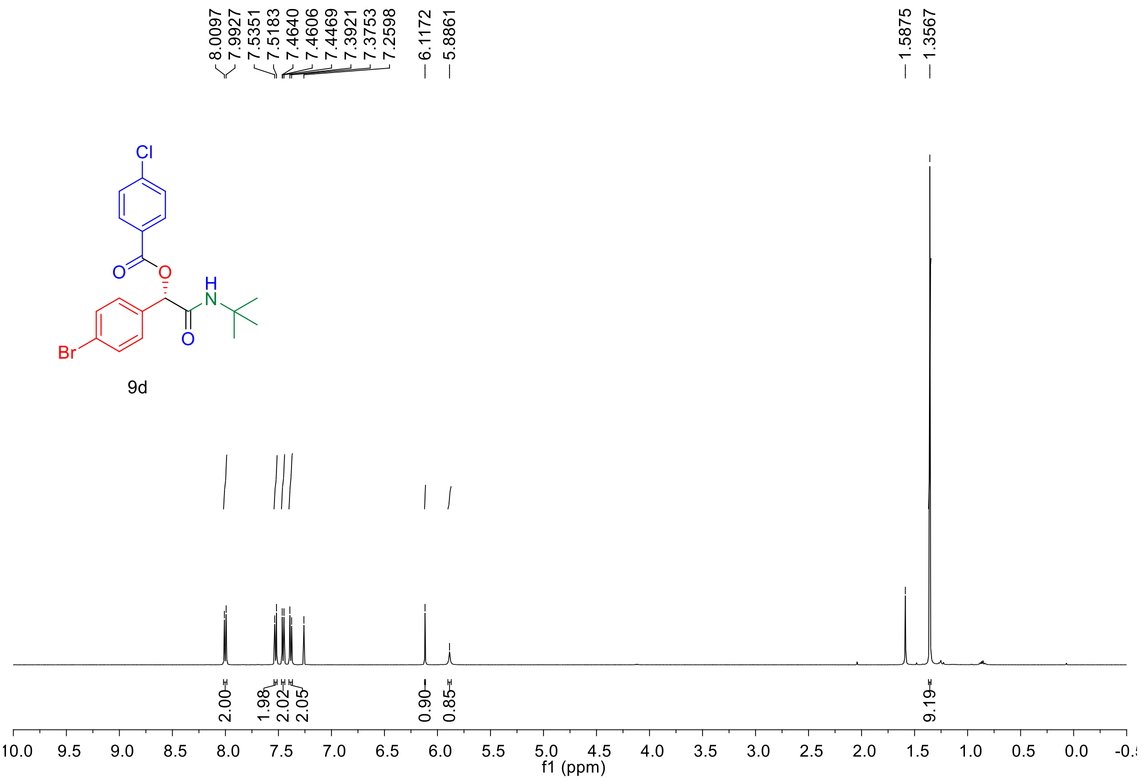

Figure S70. Expansion of the ^{1}H NMR (500 MHz, $CDCl_3$) of compound **9d**

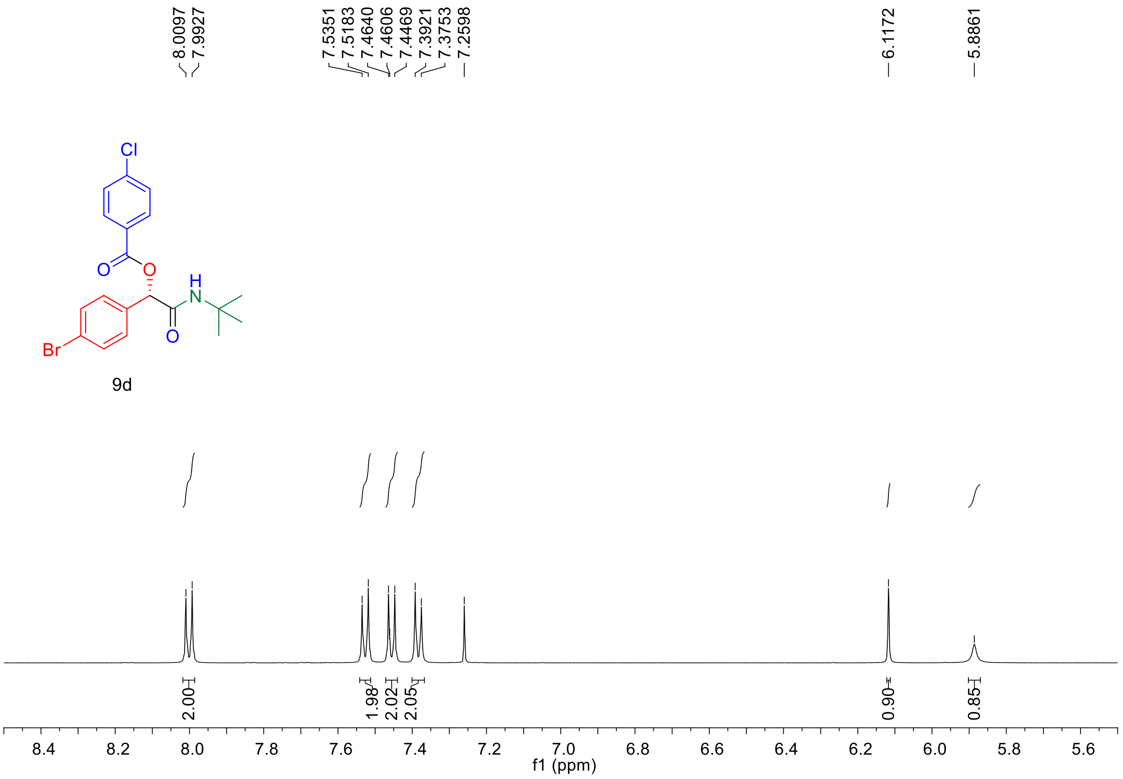

Figure S71. ^{13}C {1H} NMR (125 MHz, $CDCl_3$) of compound **9d**

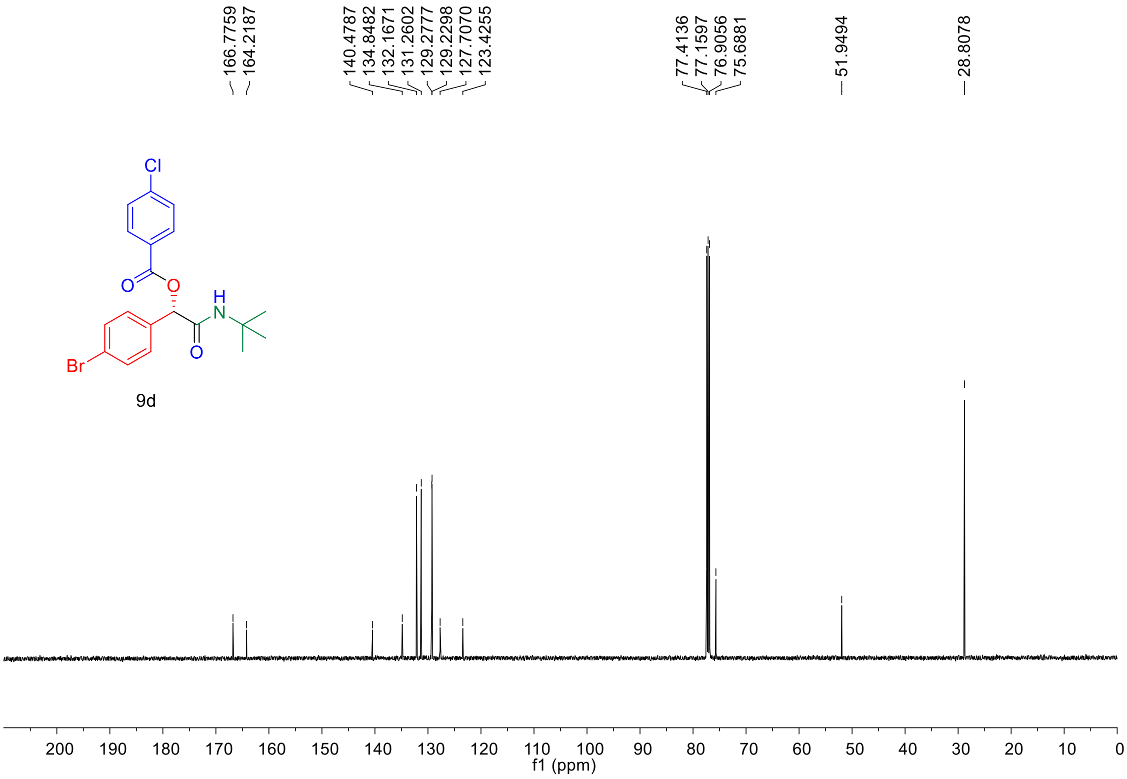

Figure S72. IR-FT of compound **9e**

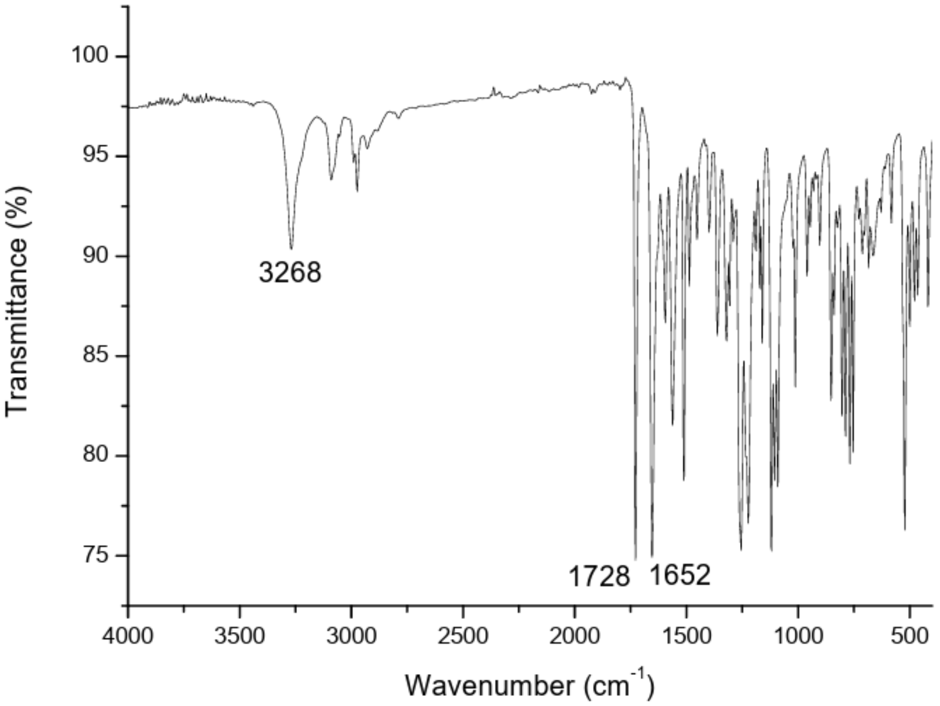

Figure S73. ^{1}H NMR (500 MHz, $CDCl_3$) of compound **9e**

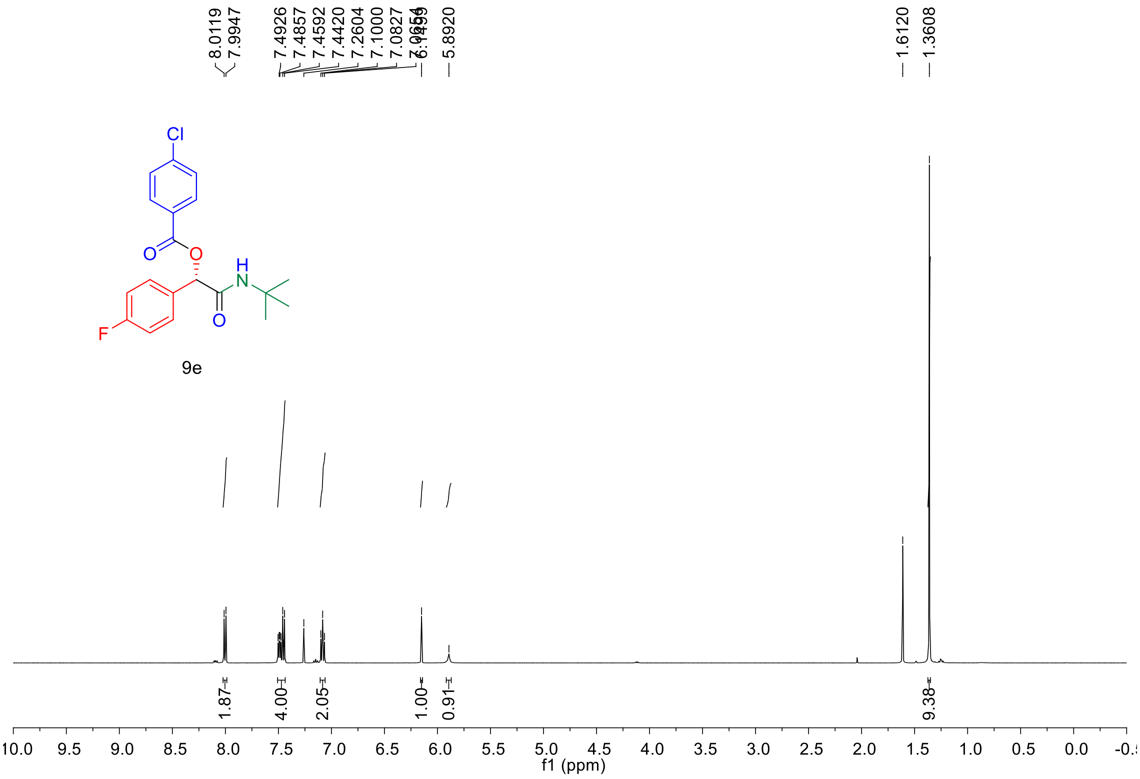

Figure S74. Expansion of the ¹H NMR (500 MHz, $CDCl_3$) of compound **9e**

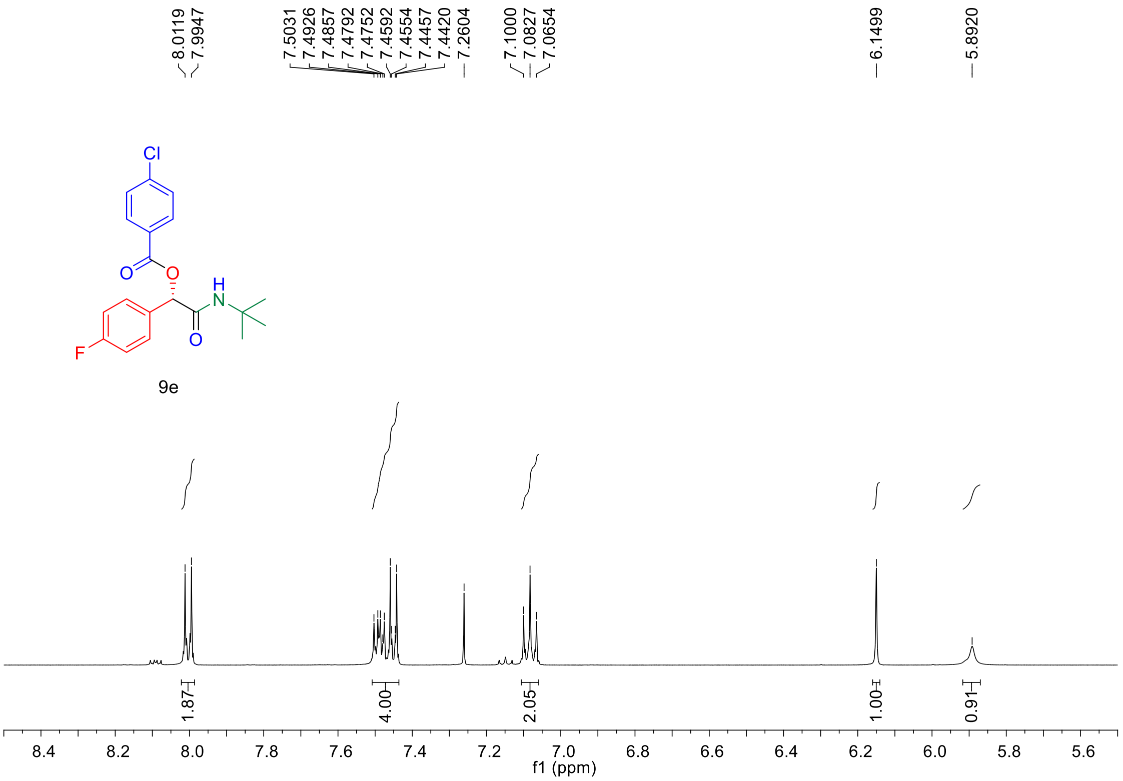

Figure S75. ¹³C {1H} NMR (125 MHz, $CDCl_3$) of compound **9e**

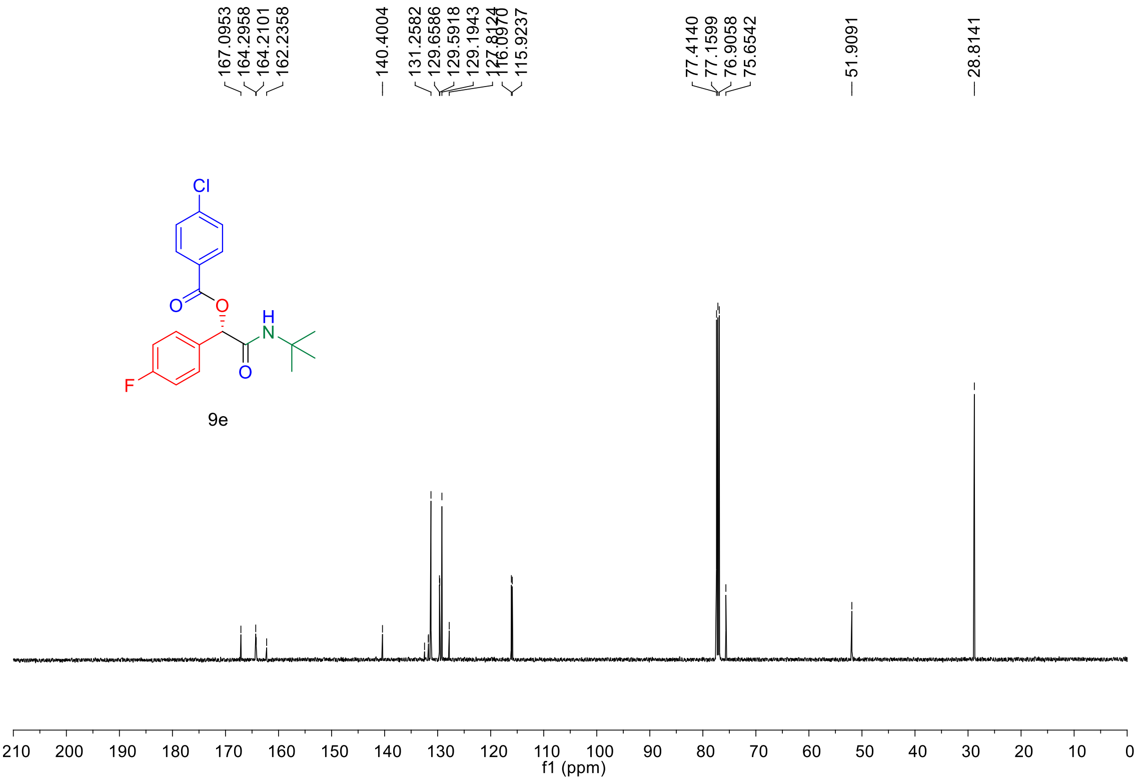

Figure S76. IR-FT of compound **9f**

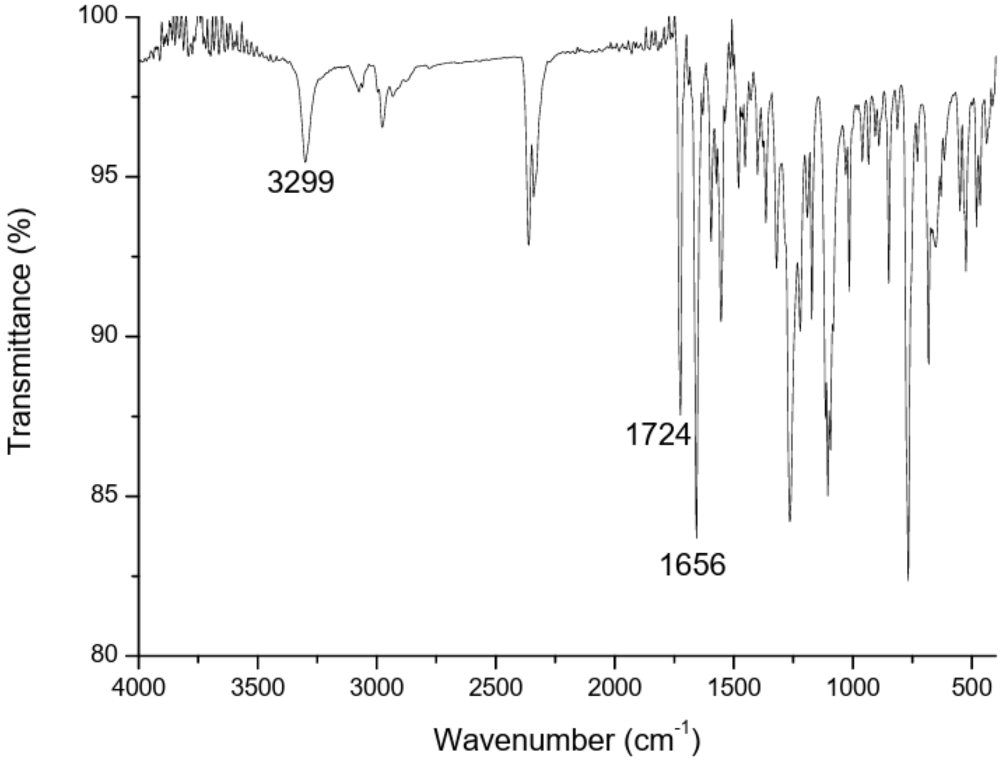

Figure S77. ^{1}H NMR (500 MHz, $CDCl_3$) of compound **9f**

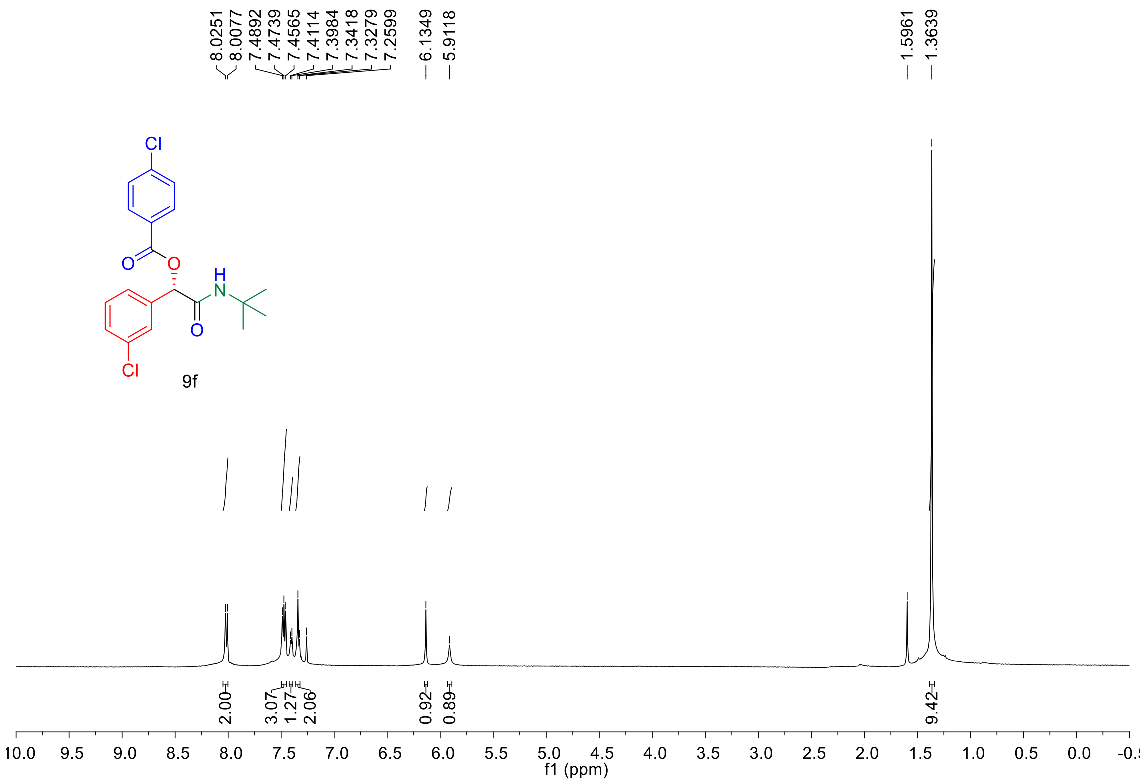

Figure S78. Expansion of the ^{1}H NMR (500 MHz, $CDCl_3$) of compound **9f**

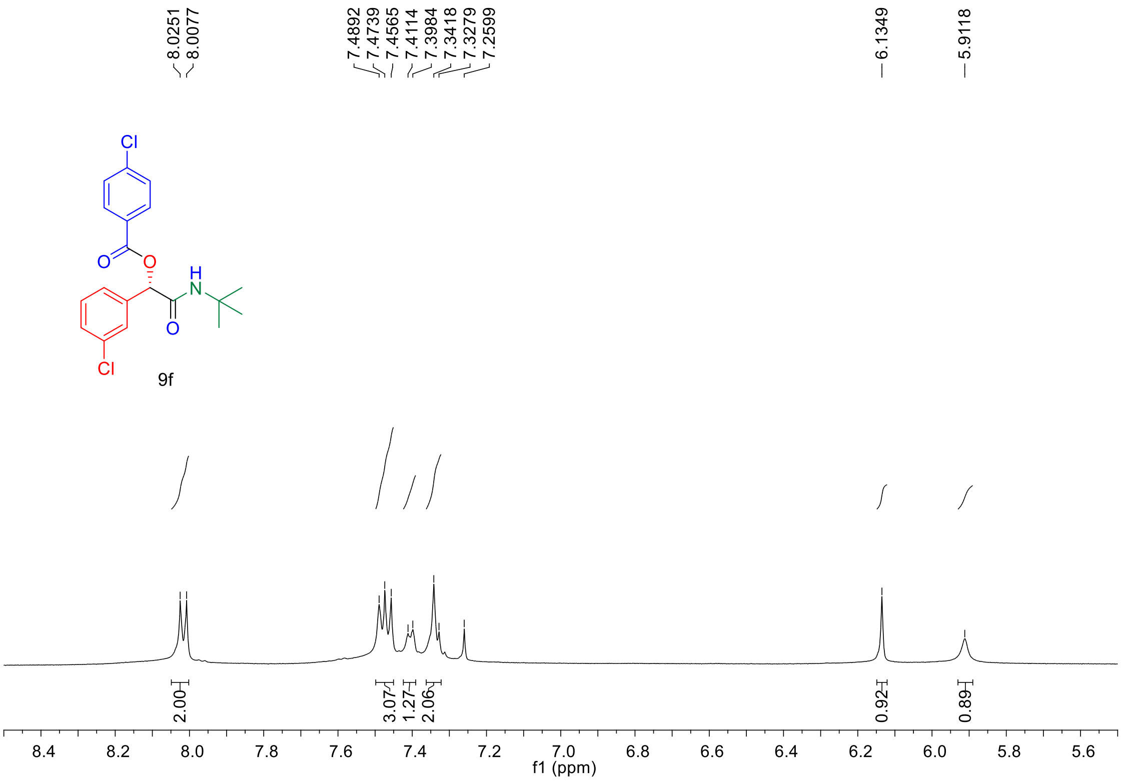

Figure S79. ^{13}C {1H} NMR (125 MHz, $CDCl_3$) of compound **9f**

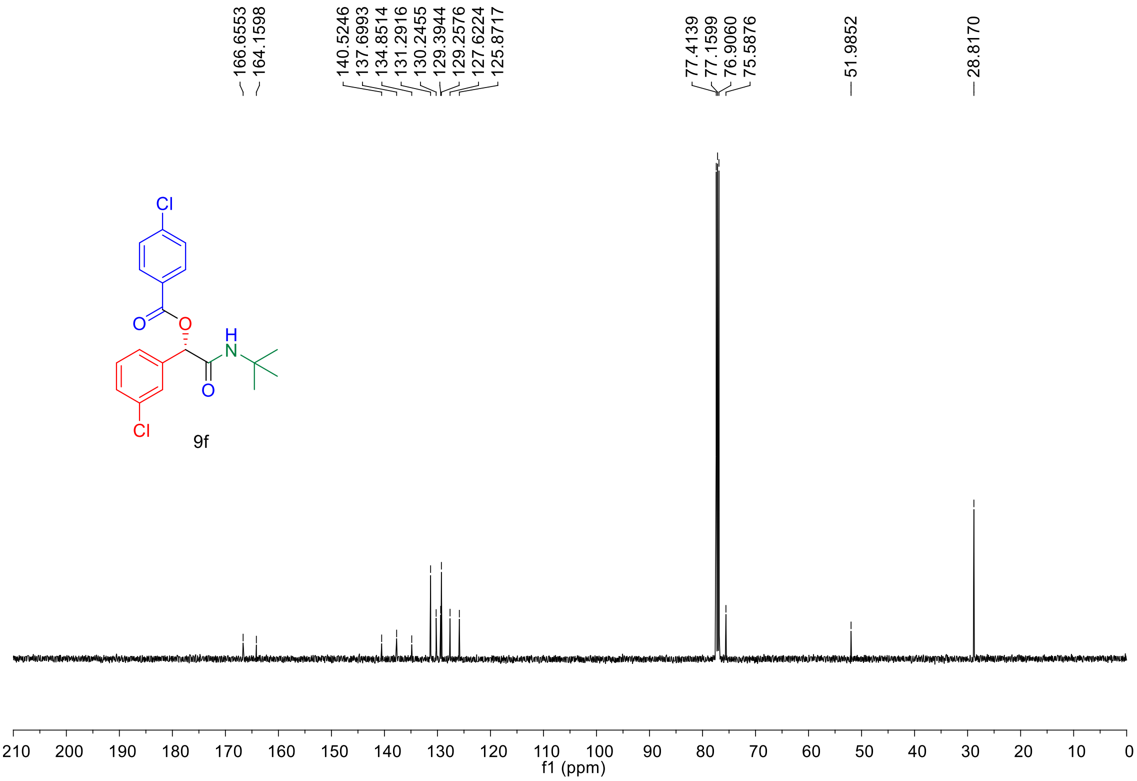

Figure S80. IR-FT of compound **9g**

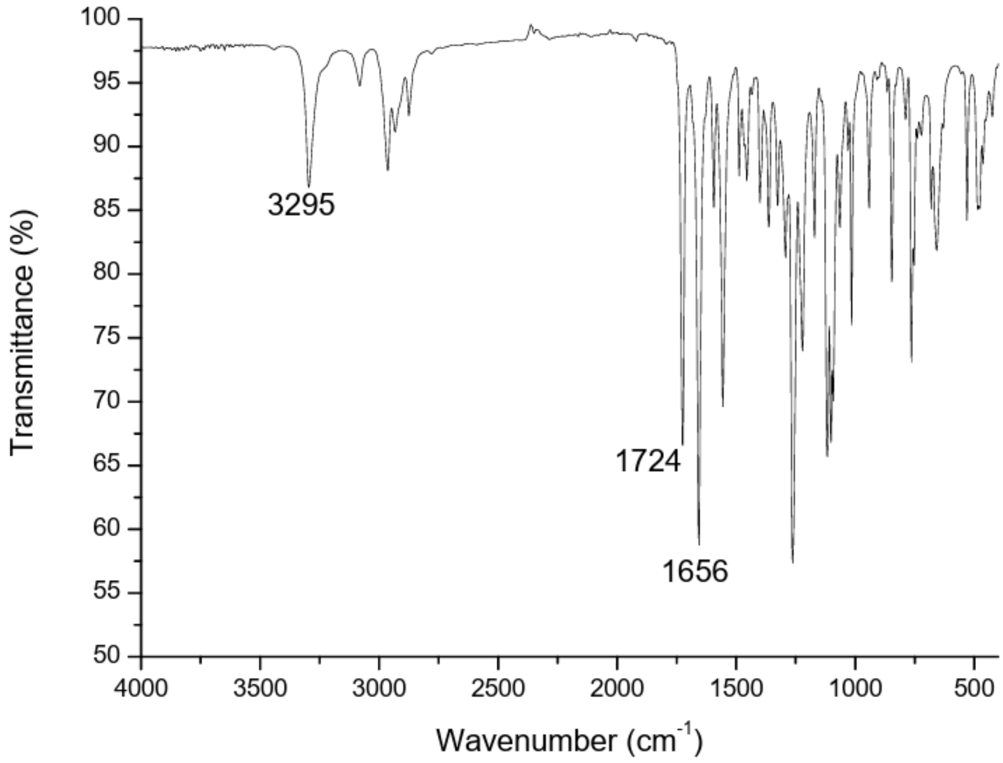

Figure S81. ^{1}H NMR (500 MHz, $CDCl_3$) of compound **9g**

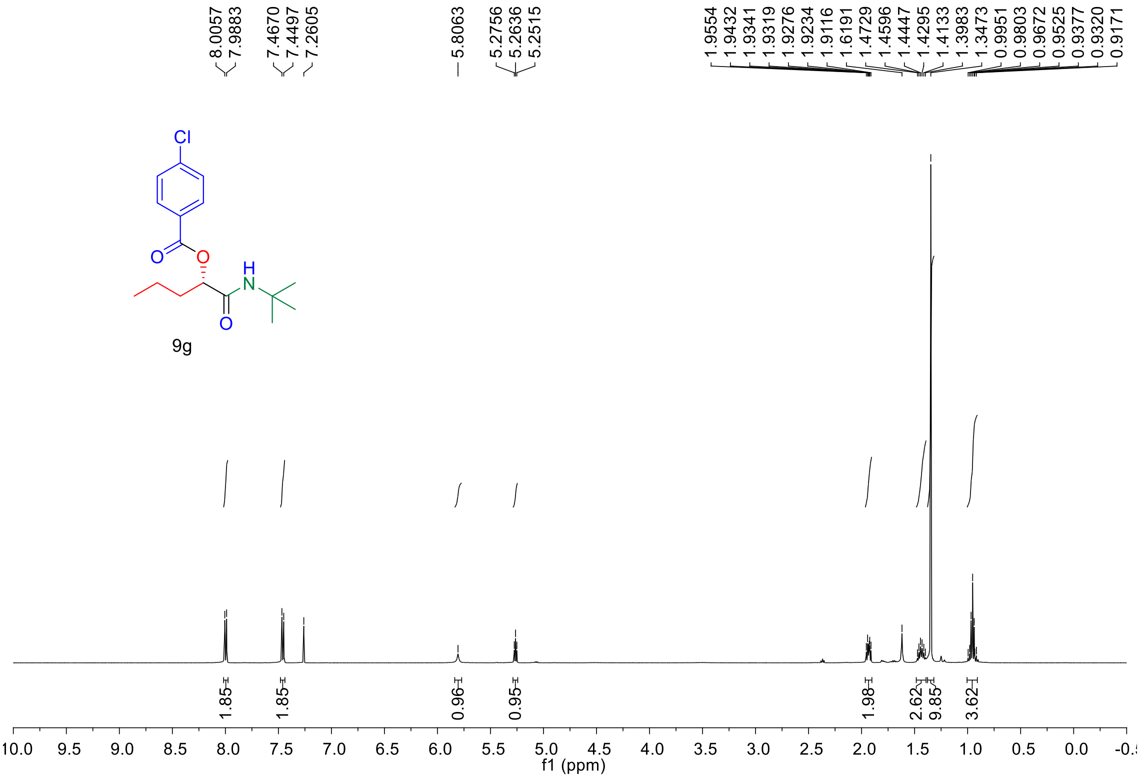

Figure S82. Expansion of the ^{1}H NMR (500 MHz, $CDCl_3$) of compound **9g**

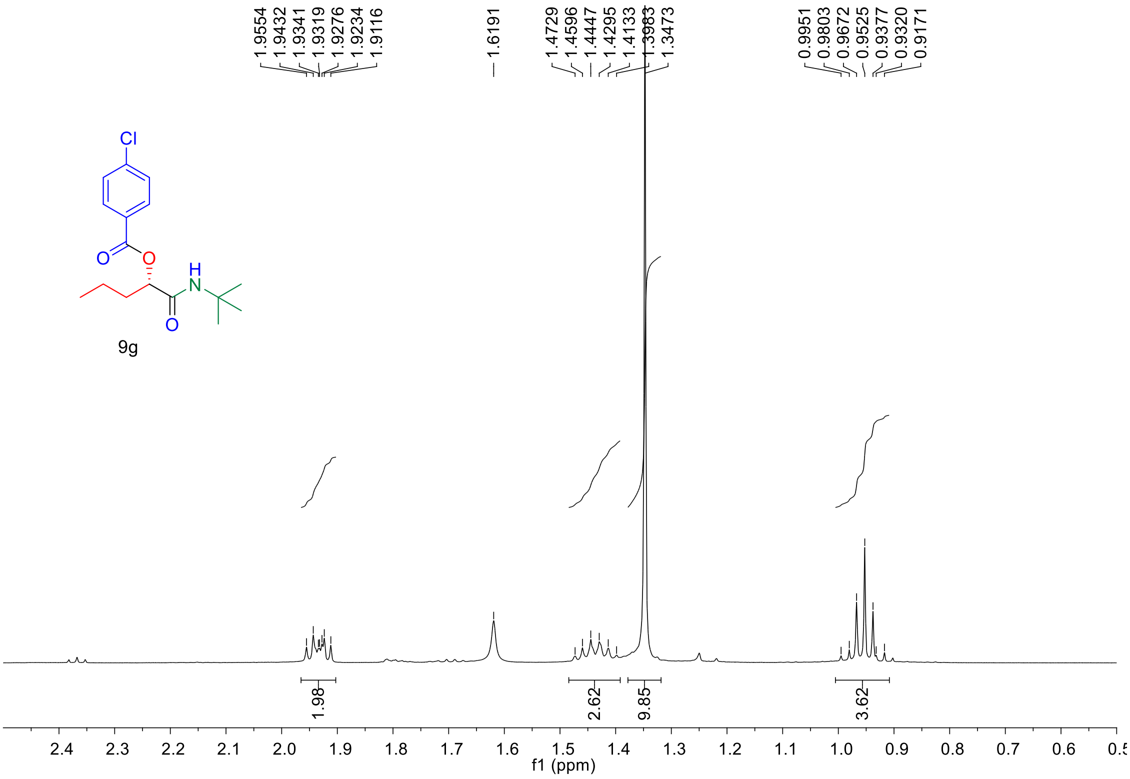

Figure S83. ^{13}C {1H} NMR (125 MHz, $CDCl_3$) of compound **9g**

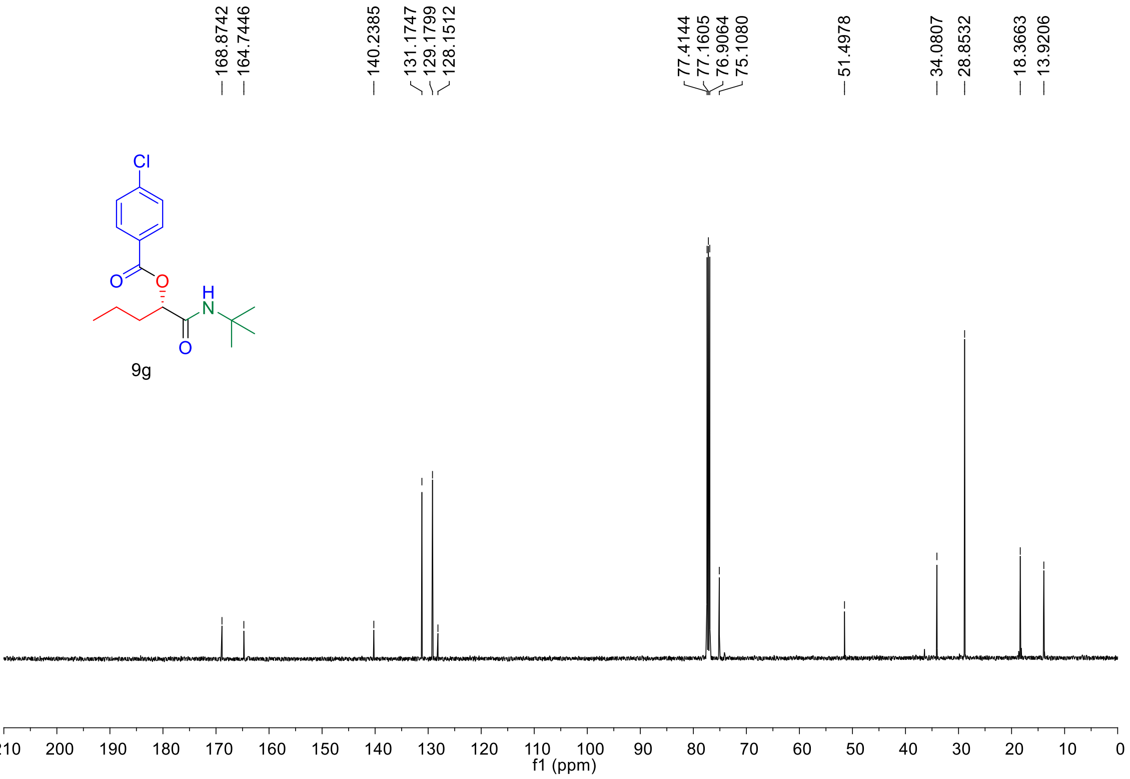

Figure S84. IR-FT of compound **9h**

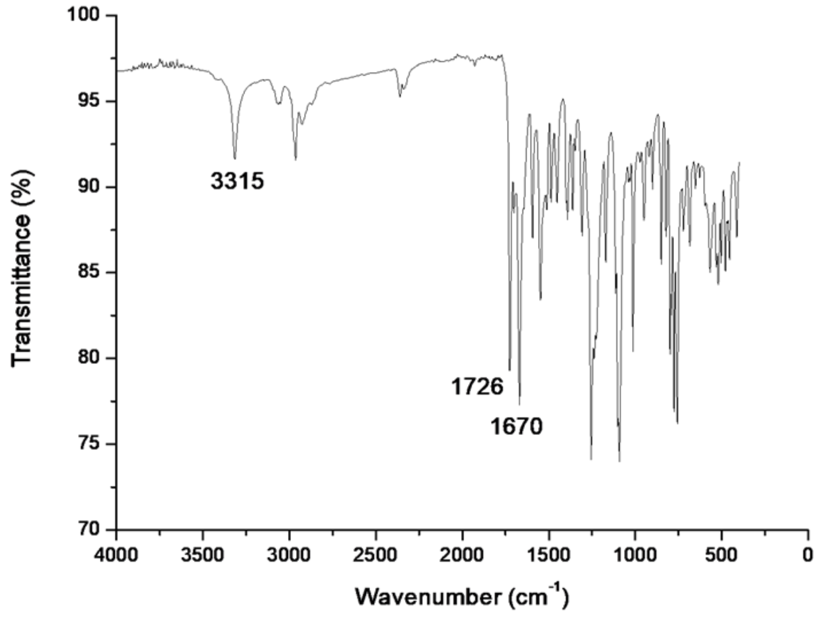

Figure S85. ^{1}H NMR (500 MHz, $CDCl_3$) of compound **9h**

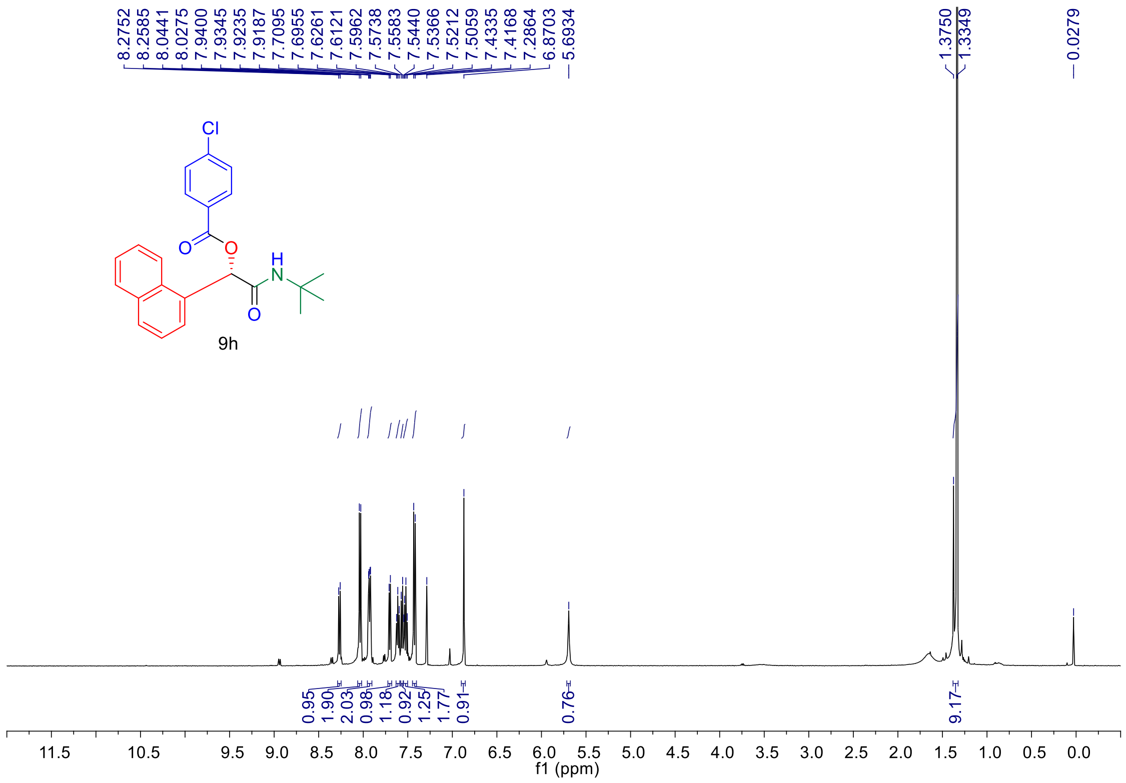

Figure S86. Expansion of the ^{1}H NMR (500 MHz, $CDCl_3$) of compound **9h**

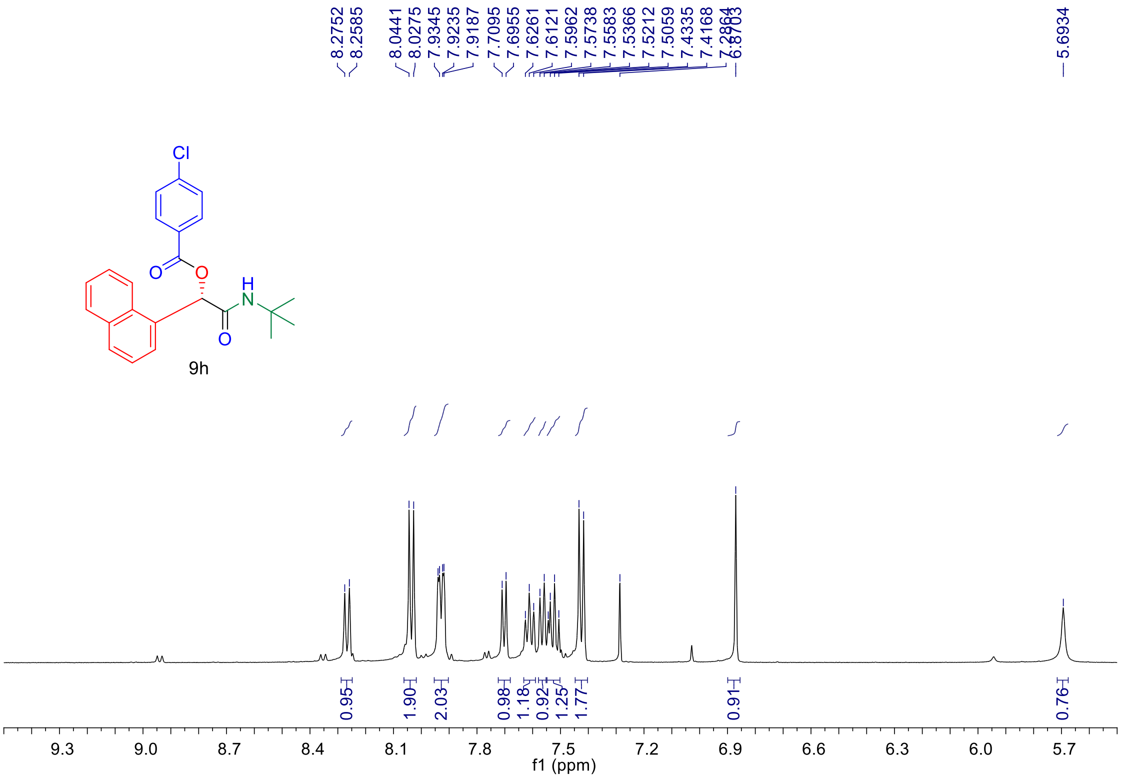

Figure S87. ^{13}C {1H} NMR (125 MHz, $CDCl_3$) of compound **9h**

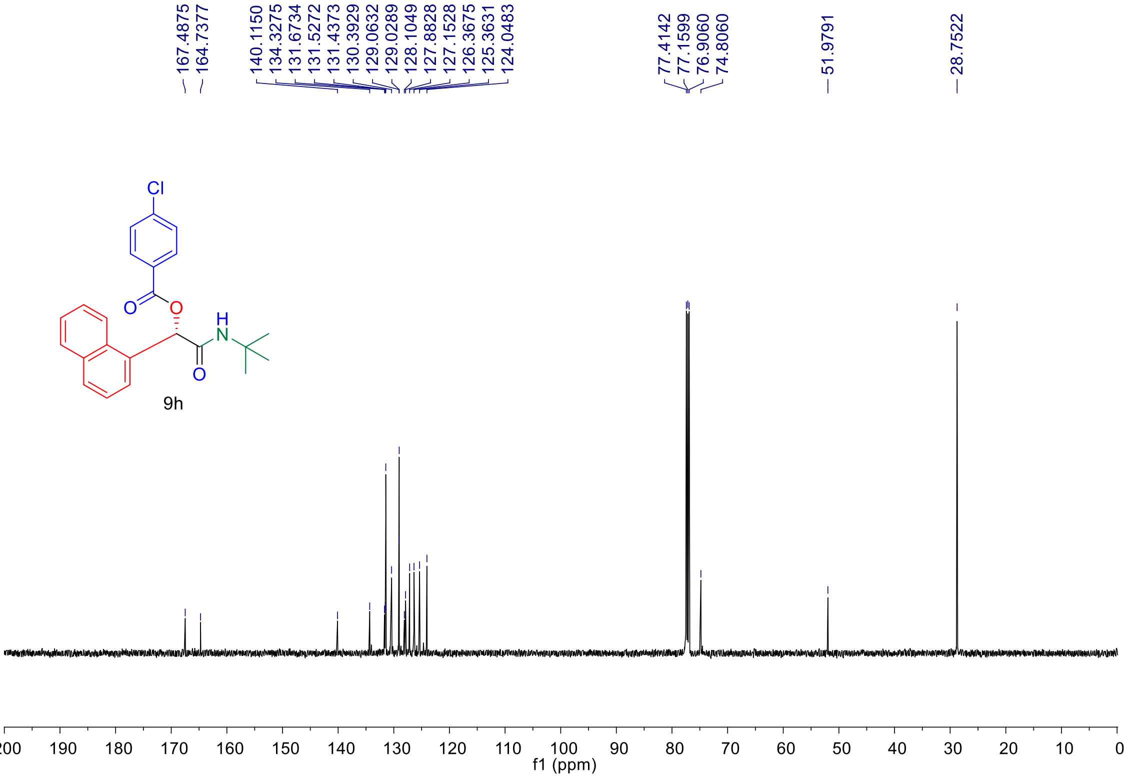

Figure S88. IR-FT of compound **9i**

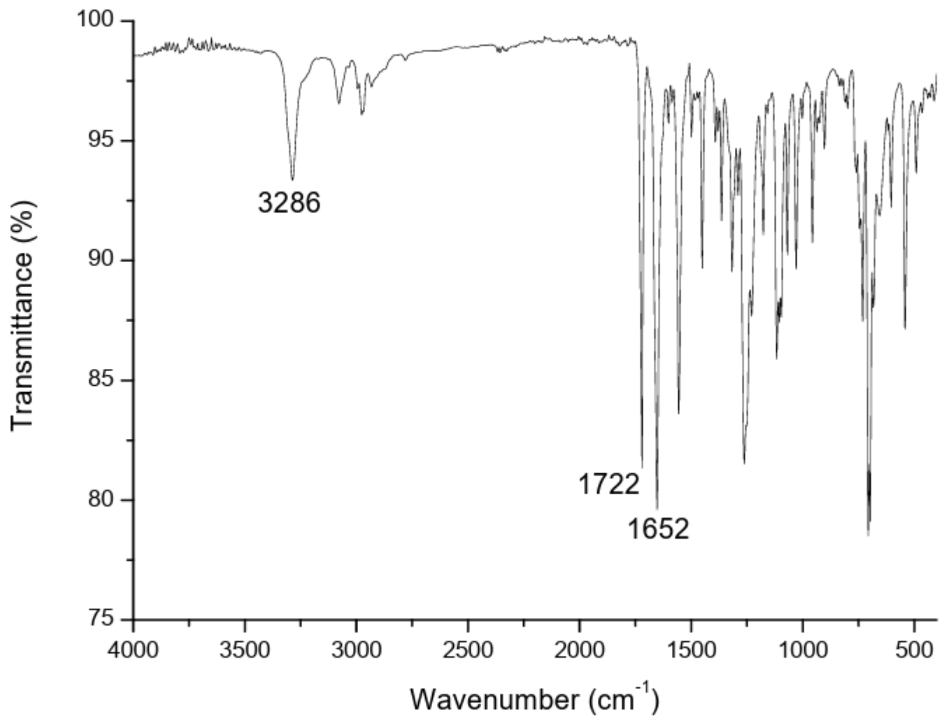

Figure S89. ^{1}H NMR (500 MHz, $CDCl_3$) of compound **9i**

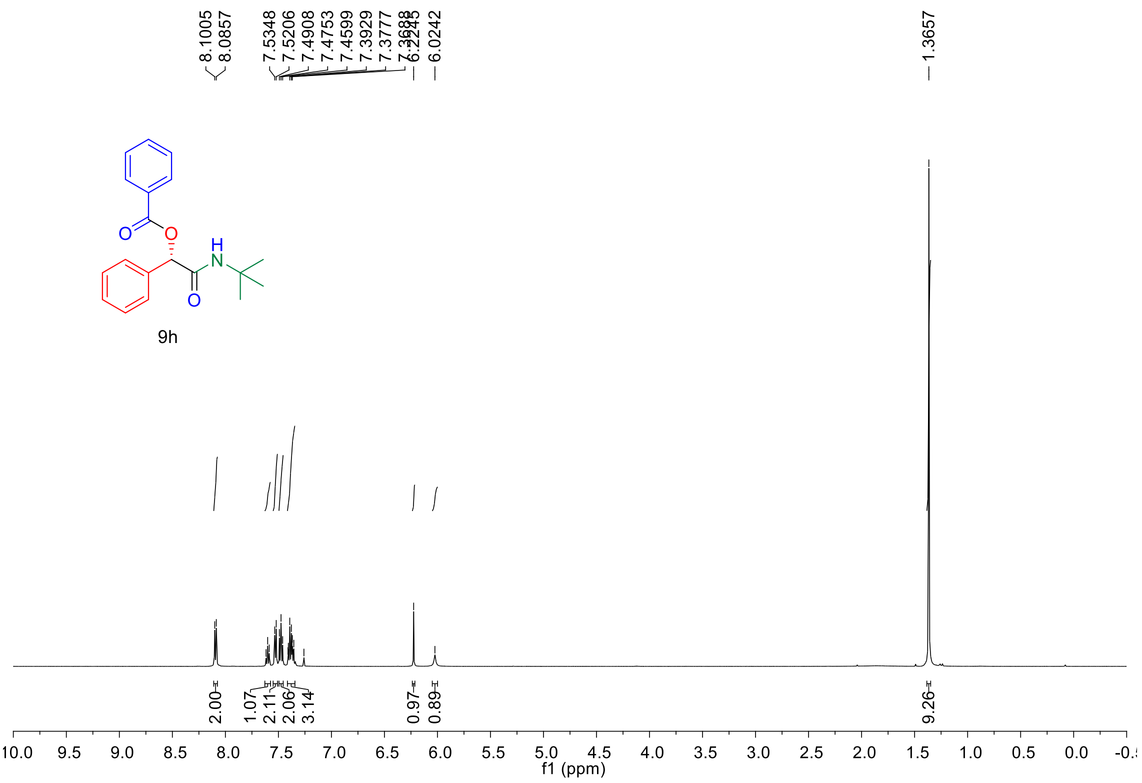

Figure S90. Expansion of the ^{1}H NMR (500 MHz, $CDCl_3$) of compound **9i**

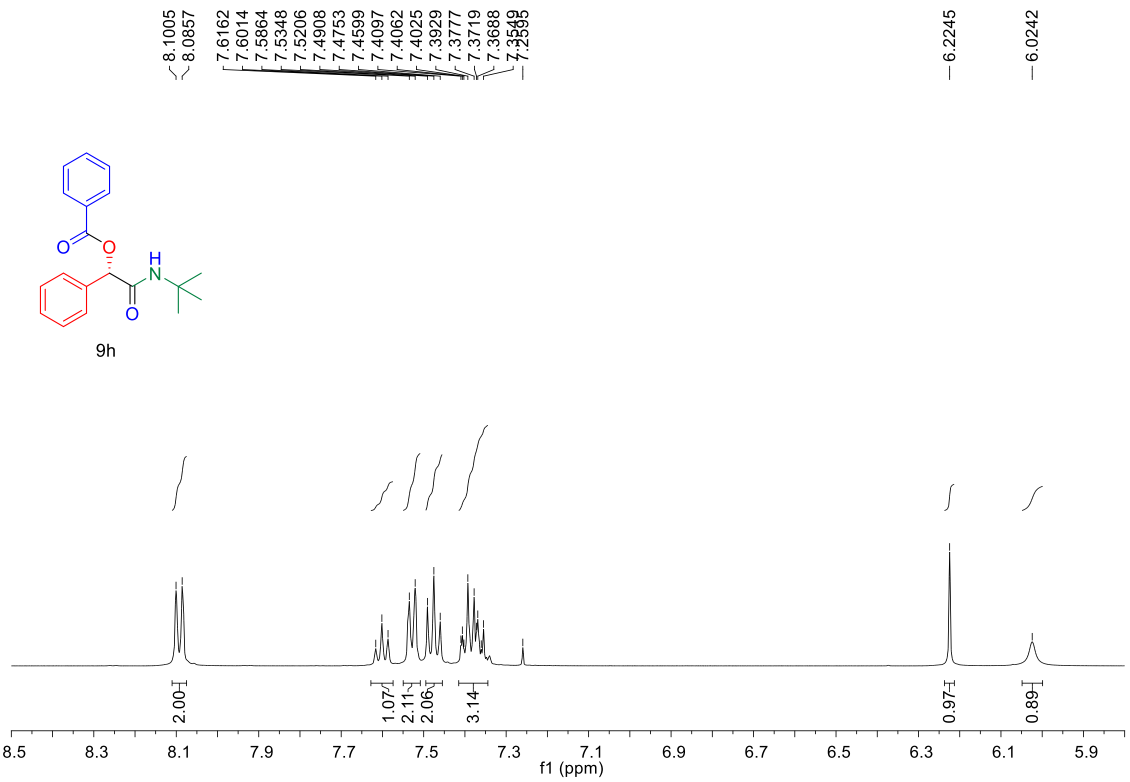

Figure S91. ^{13}C {1H} NMR (125 MHz, $CDCl_3$) of compound **9i**

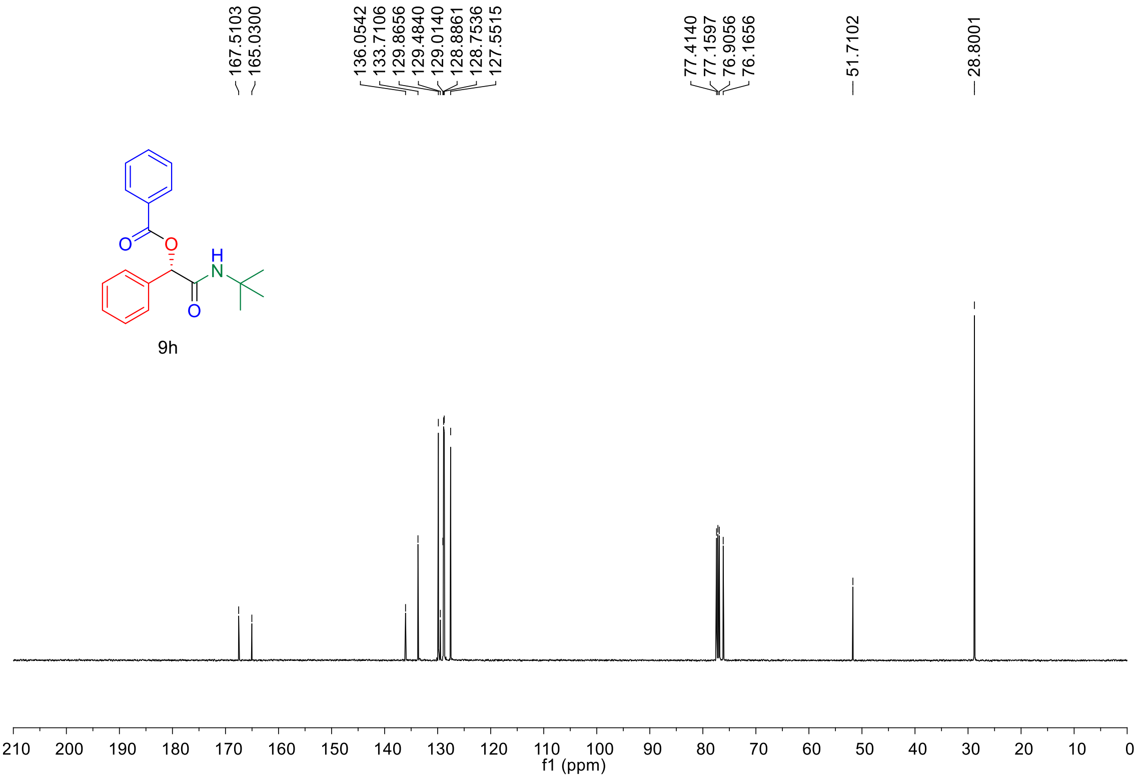

Figure S92. IR-FT of compound **9j**

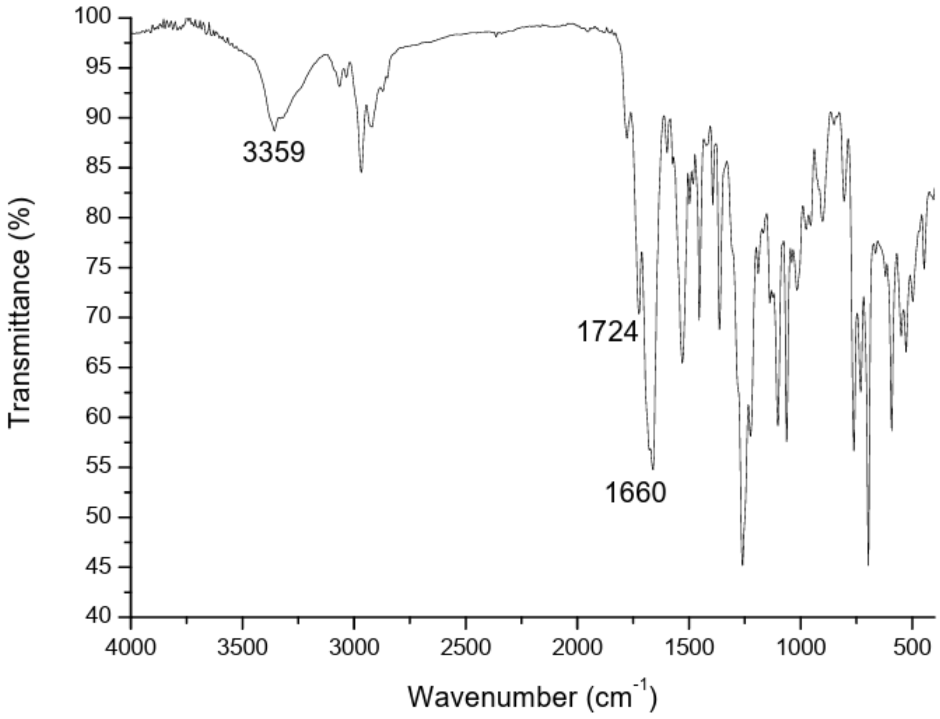

Figure S93. ^{1}H NMR (500 MHz, CDCl$_3$) of compound **9j**

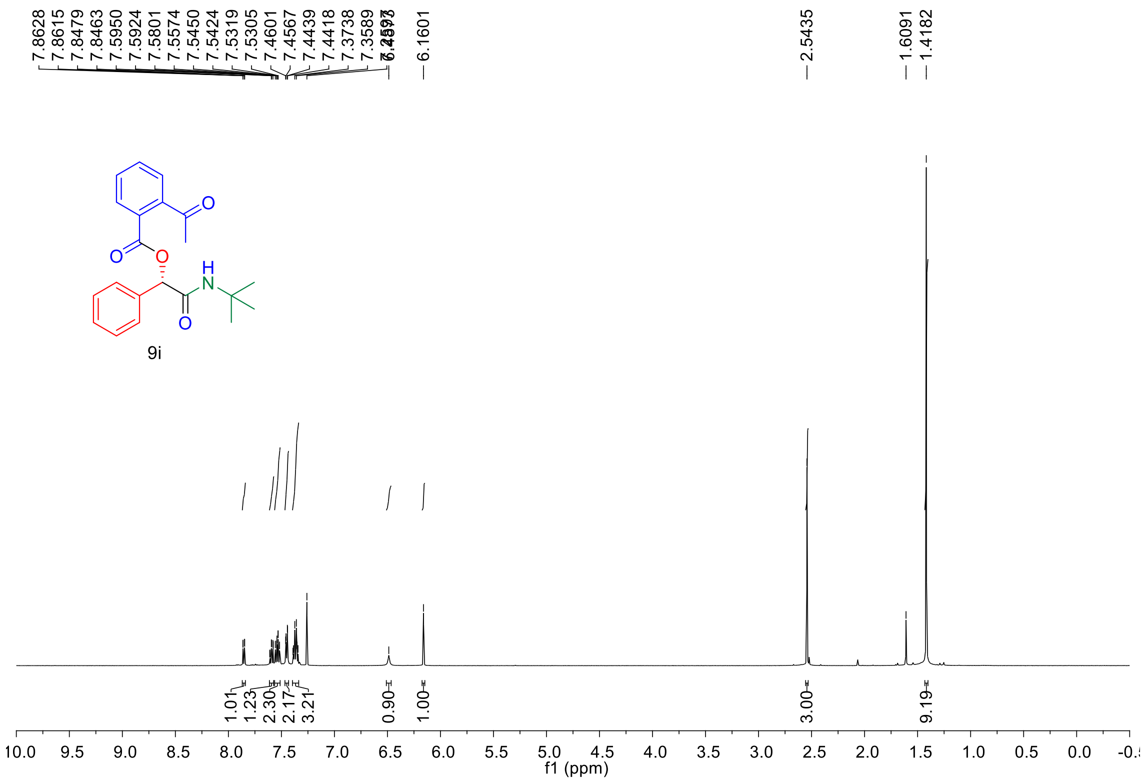

Figure S94. Expansion of the ^{1}H NMR (500 MHz, $CDCl_3$) of compound **9j**

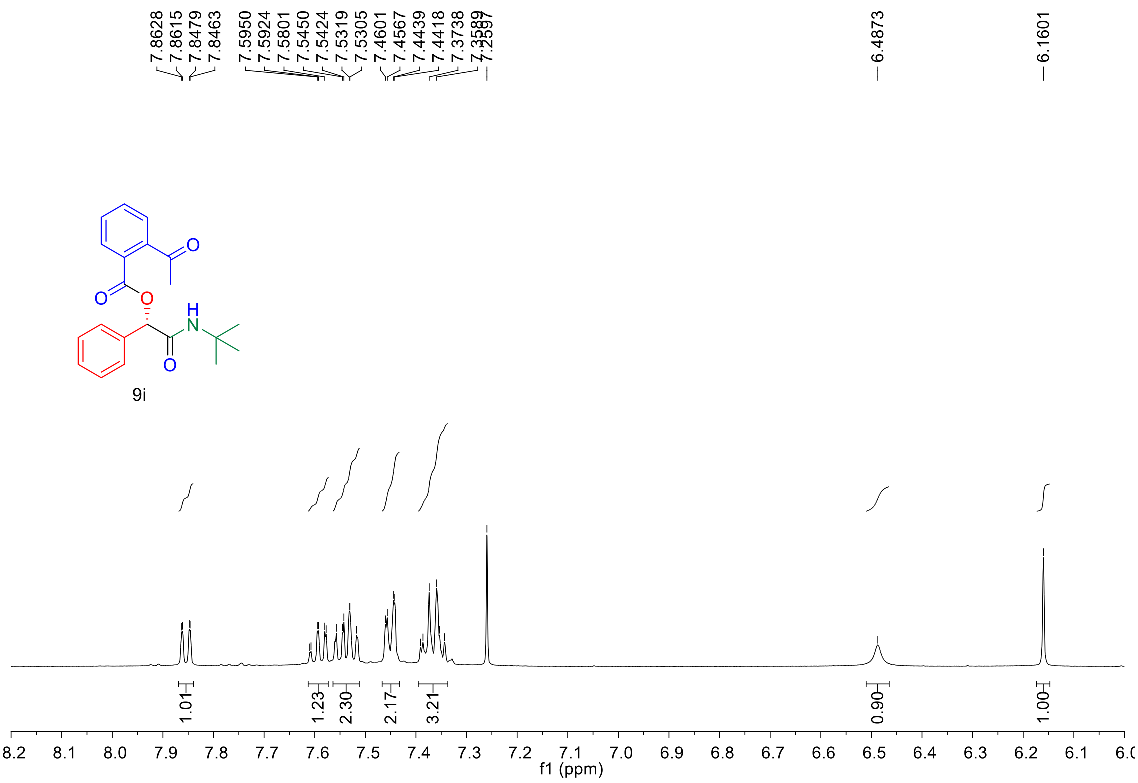

Figure S95. ^{13}C {1H} NMR (125 MHz, $CDCl_3$) of compound **9j**

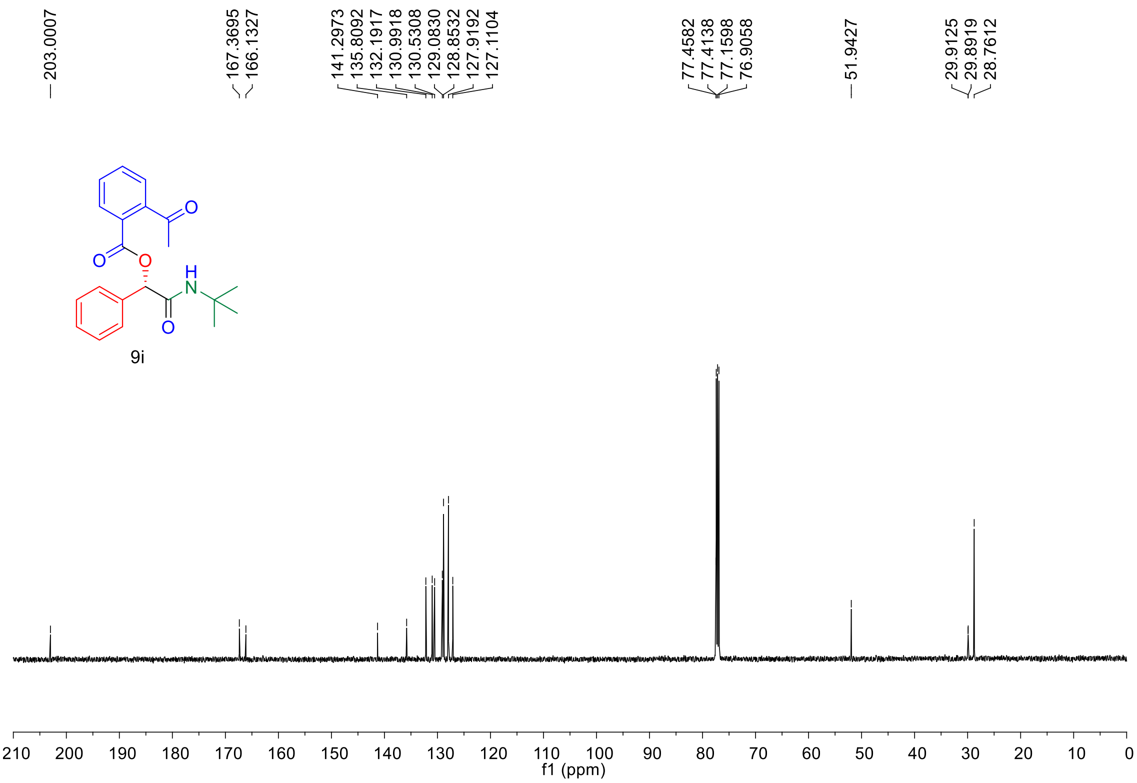

S7. Copies of HRMS (ESI-TOF) spectra for all novel compounds

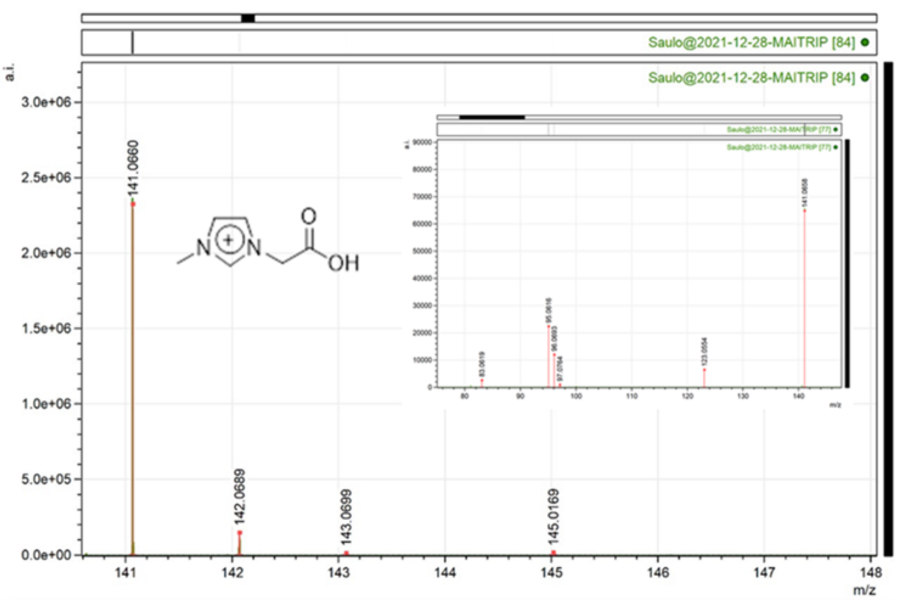

Saulo@2021-12-28-MAITRIP [84]
a.i.
3.0e+06
2.5e+06
2.0e+06
1.5e+06
1.0e+06
5.0e+05
0.0e+00
141.0660
142.0689
143.0699
145.0169
141
142
143
144
145
146
147
148
m/z
OH

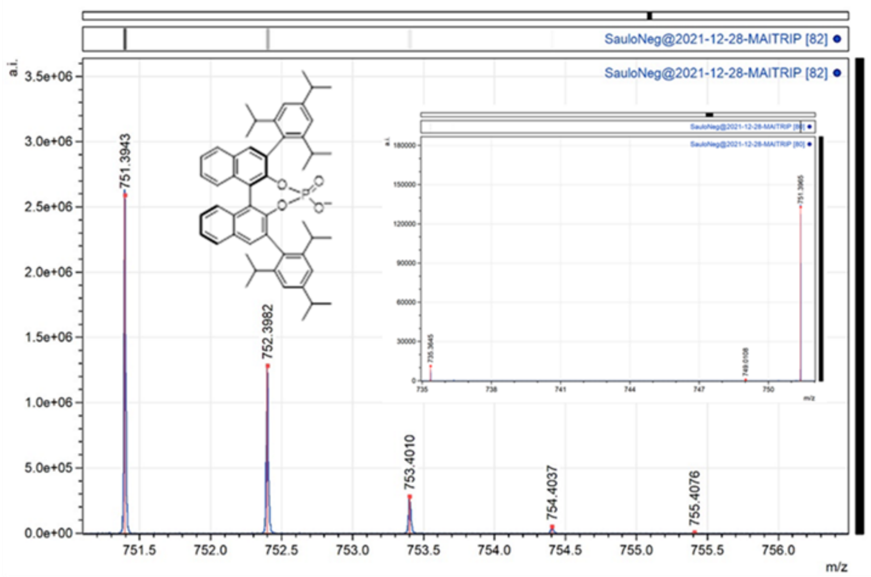

SauloNeg@2021-12-28-MAITRIP [82]
a.i.
3.5e+06
3.0e+06
2.5e+06
2.0e+06
1.5e+06
1.0e+06
5.0e+05
0.0e+00
751.3943
752.3982
753.4010
754.4037
755.4076
751.5
752.0
752.5
753.0
753.5
754.0
754.5
755.0
755.5
756.0
m/z

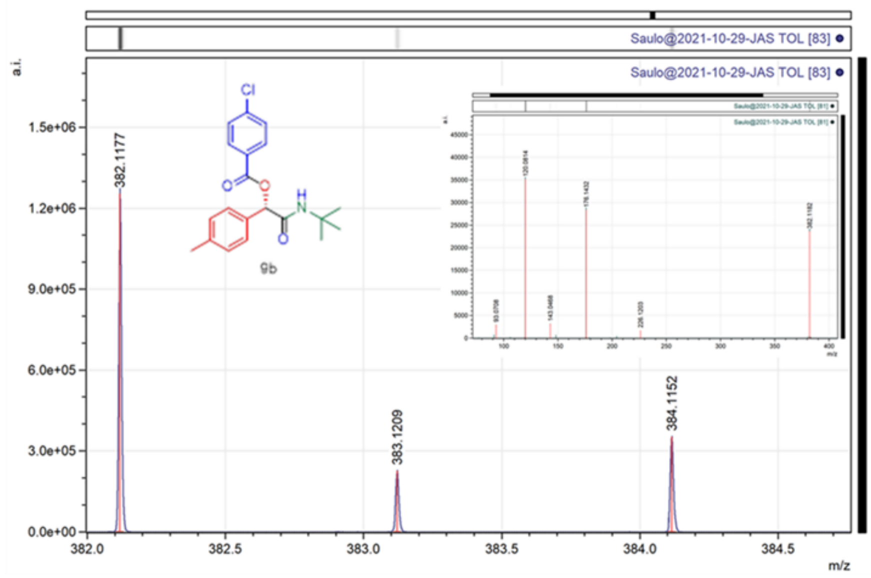

Saulo@2021-10-29-JAS TOL [83]
a.i.
1.5e+06
1.2e+06
9.0e+05
6.0e+05
3.0e+05
0.0e+00
382.1177
383.1209
384.1152
382.0
382.5
383.0
383.5
384.0
384.5
m/z
Cl
9b

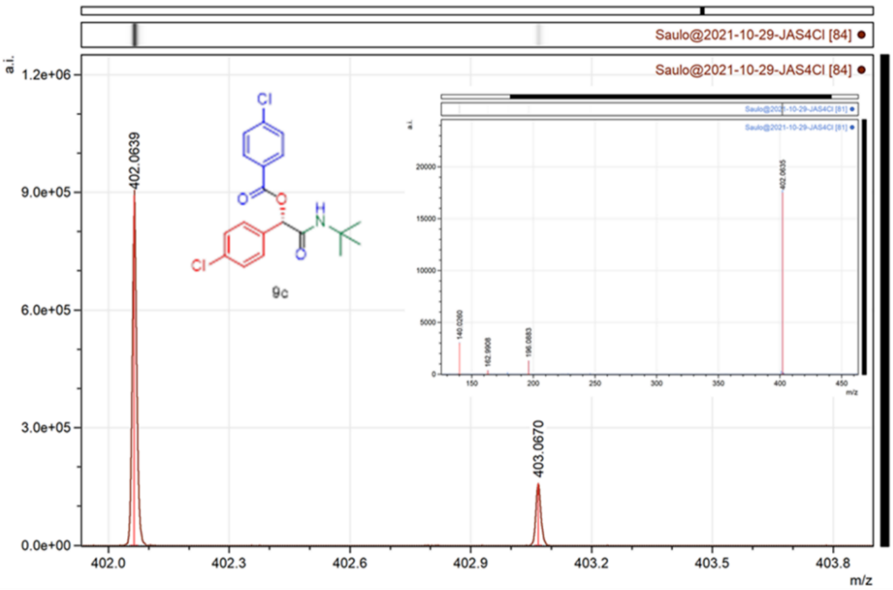
Saulo@2021-10-29-JAS4Cl [84]
a.i.
1.2e+06
9.0e+05
6.0e+05
3.0e+05
0.0e+00
402.0639
403.0670
9c
402.0
402.3
402.6
402.9
403.2
403.5
403.8
m/z

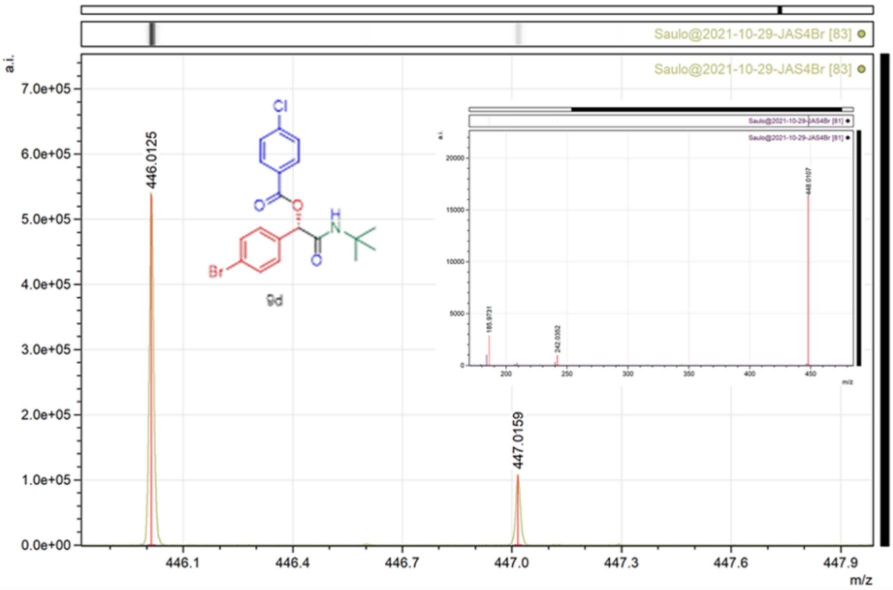
Saulo@2021-10-29-JAS4Br [83]
a.i.
7.0e+05
6.0e+05
5.0e+05
4.0e+05
3.0e+05
2.0e+05
1.0e+05
0.0e+00
446.0125
447.0159
9d
446.1
446.4
446.7
447.0
447.3
447.6
447.9
m/z

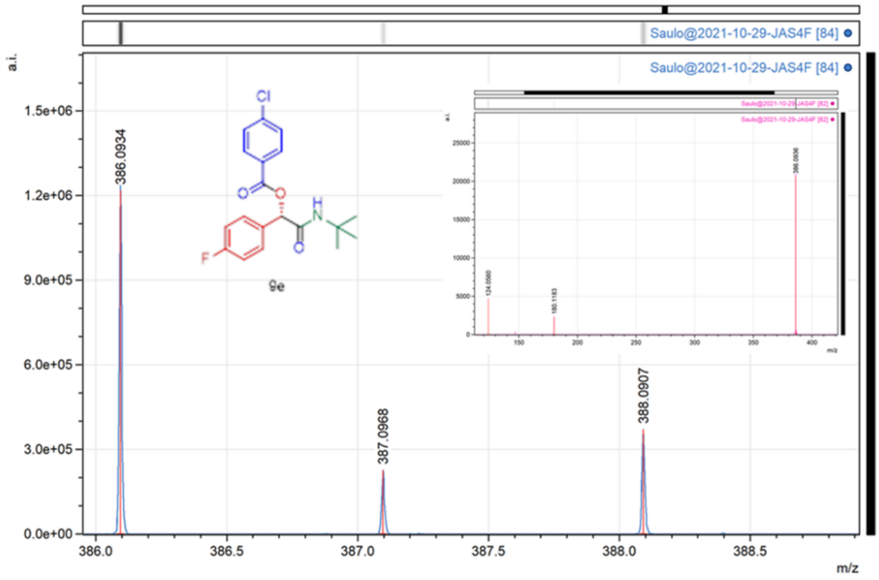
Saulo@2021-10-29-JAS4F [84]
a.i.
1.5e+06
1.2e+06
9.0e+05
6.0e+05
3.0e+05
0.0e+00
386.0934
387.0968
388.0907
9e
386.0
386.5
387.0
387.5
388.0
388.5
m/z

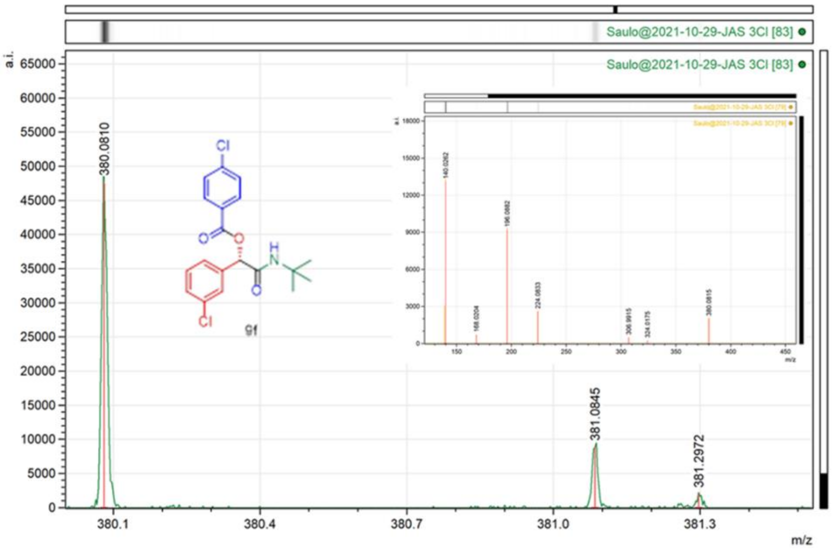
Saulo@2021-10-29-JAS 3Cl [83]
a.i.
65000
60000
55000
50000
45000
40000
35000
30000
25000
20000
15000
10000
5000
0
380.0810
381.0845
381.2972
380.1
380.4
380.7
381.0
381.3
m/z
Cl
O
O
H
N
O
Cl
9f
140.0262
168.0204
196.0682
224.0633
306.9915
324.0175
380.0815

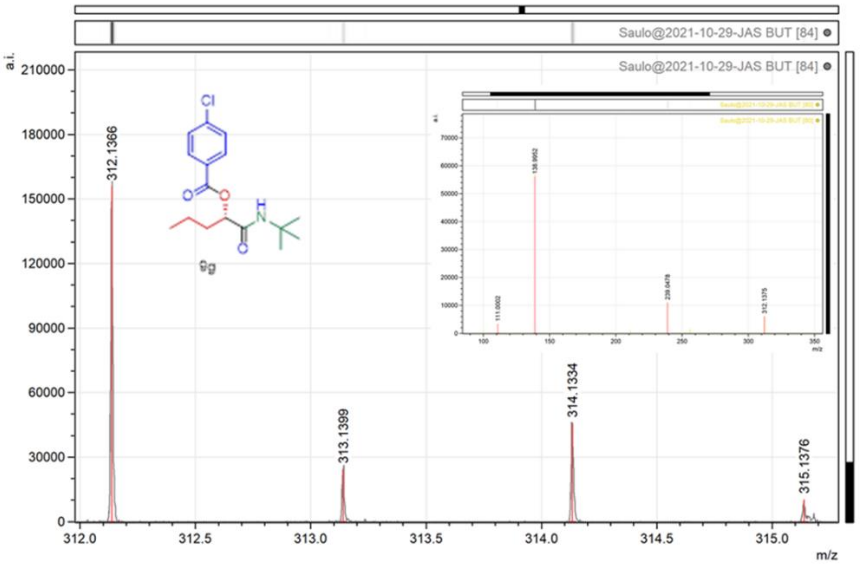
Saulo@2021-10-29-JAS BUT [84]
a.i.
210000
180000
150000
120000
90000
60000
30000
0
312.1366
313.1399
314.1334
315.1376
312.0
312.5
313.0
313.5
314.0
314.5
315.0
m/z
Cl
O
O
H
N
O
9g
138.9952
111.0002
239.0478
312.1375

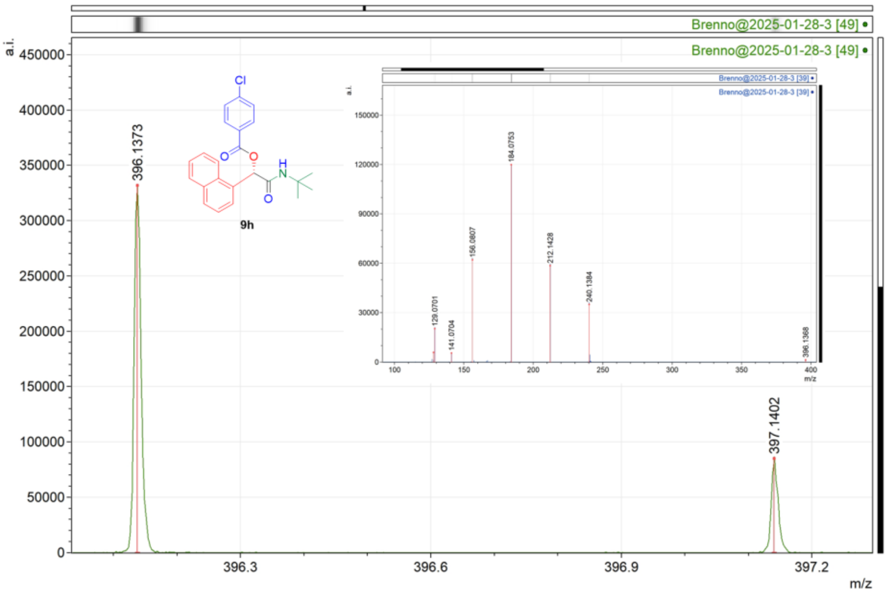
Brenno@2025-01-28-3 [49]
a.i.
450000
400000
350000
300000
250000
200000
150000
100000
50000
0
396.1373
397.1402
396.3
396.6
396.9
397.2
m/z
Cl
O
O
H
N
O
9h
128.0701
141.0704
156.0807
184.0753
212.1428
240.1384
396.1368

S8. Determination of the absolute configuration of major Passerini product

The absolute configuration of major Passerini products was determined by comparison of chromatograms (enantioenriched samples) corresponding to **9j** available in the literature.[1] The samples used in this determination were obtained using the general procedure for the catalyzed asymmetric Passerini reaction and 20 mol% of chiral catalyst **(*S*)-1**. Retention time (t_R) = 19.6 min (enantiomer *R*, minor); (t_R) = 24.3 min (enantiomer *S*, major); e.r. = 41:59 (Figure S91). Additionally, we carried out the experiment using the optimized conditions of the asymmetric protocol and 20 mol% of chiral catalyst **(*R*)-1**, obtaining as the major enantiomer, the *R*-product, e.r. = 61:39 (Figure S92).

Figure S96. HPLC traces of 9j – racemic

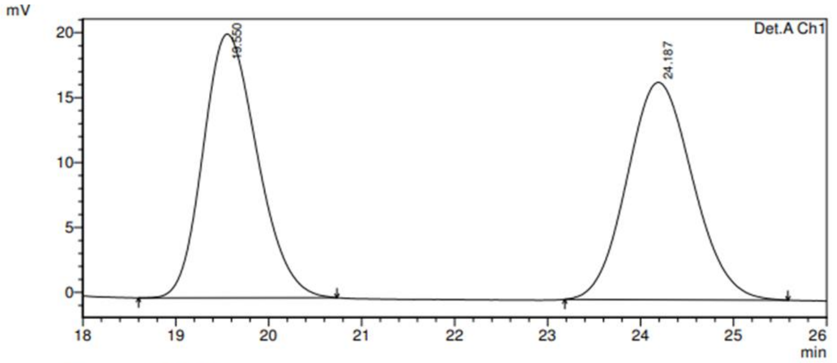

Detector A Ch1 254nm

Peak#	Ret. Time	Area	Height	Area %	Height %
1	19.550	823966	20310	49.946	54.824
2	24.187	825759	16736	50.054	45.176
Total		1649725	37046	100.000	100.000

Figure S97. HPLC traces of 9j – asymmetric ACDC (20 mol% of chiral catalyst (*S*)-1)

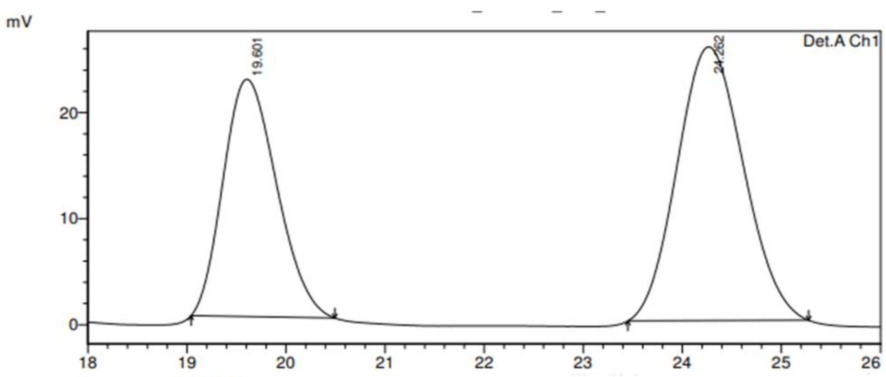

Detector A Ch1 254nm

Peak#	Ret. Time	Area	Height	Area %	Height %
1	19.601	840717	22365	41.102	46.434
2	24.262	1204748	25801	58.898	53.566
Total		2045464	48166	100.000	100.000

Figure S98. **HPLC traces of 9j – asymmetric ACDC (20 mol% of chiral catalyst (*R*)-1)**

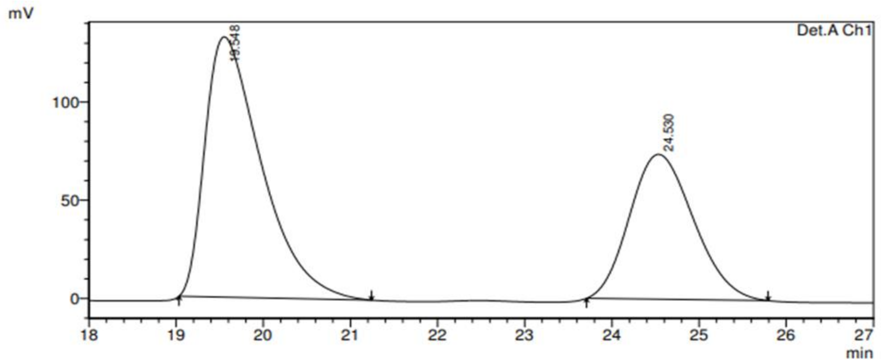

Detector A Ch1 254nm

Peak#	Ret. Time	Area	Height	Area %	Height %
1	19.548	5963889	132527	61.261	64.232
2	24.530	3771340	73798	38.739	35.768
Total		9735230	206325	100.000	100.000

S9. Tables with reaction and activation thermodynamic properties.

Table S2. Reaction and activation thermodynamic properties for the Passerine reaction catalysed by a counteranion.[a]

**Step 1**				
**Process**	**ΔEele**[b]	**ΔG$_T$**[c]	**ΔΔGsolv**[d]	**ΔG$_{sol}$**[e]
MS0 → TS1S	5.10	2.12	1.42	8.64
MS0 → MS1S	0.17	3.68	-1.09	2.76
MS0 → TS1R	2.31	2.95	3.93	9.19
MS0 → MS1R	-0.60	4.85	-1.37	2.88
**Step 2**				
**Process**	**ΔEele**[b]	**ΔG$_T$**[c]	**ΔΔGsolv**[d]	**ΔG$_{sol}$**[e]
MS0 → TS2S	2.57	5.68	0.26	8.51
MS0 → MS2S	-12.79	8.09	0.14	-4.56
MS0 → TS2R	2.63	4.83	0.22	7.67
MS0 → MS2R	-12.74	8.75	0.41	-3.58
**Step 3**				
**Process**	**ΔEele**[b]	**ΔG$_T$**[c]	**ΔΔGsolv**[d]	**ΔG$_{sol}$**[e]
MS0 → TS3S	1.27	6.80	1.16	9.22
MS0 → MS3S	-17.63	9.26	1.04	-7.33
MS0 → TS3R	2.14	5.35	0.73	8.23
MS0 → MS3R	-15.10	8.72	0.09	-6.29
**Step 4**				
**Process**	**ΔEele**[b]	**ΔG$_T$**[c]	**ΔΔGsolv**[d]	**ΔG$_{sol}$**[e]
MS0 → TS4S	-12.73	7.71	2.71	-2.31
MS0 → MS4S	-39.87	9.38	2.38	-28.11
MS0 → TS4R	-17.22	6.05	2.16	-9.01
MS0 → MS4R	-38.48	8.45	0.44	-29.60

[a] Units in kcal mol^{-1}. Standard state of 1 mol L^{-1} for all the species. [b] Electronic energy obtained at M06-2X/def2-TZVPP (ma-def2-TZVPP for O and N atoms) level of theory. [c] Nuclear contributions (translation, vibration and rotation) to the free energy. [d] Solvation free energy for pentane solvent obtained at SMD/X3LYP/def2-SVP (ma-def2-SVP for O and N atoms) level of theory. [e] Solution phase free energy.

S10. Coordinates of the optimized structures.

Coordinates of the optimized structures, obtained at the X3LYP/def2-SVP (ma-def2-SVP for O and N atoms) level of theory.

MS0

C	5.21010559518071	2.32562988440427	0.69251624952561
O	4.69029413534369	1.40291653604130	0.09667345866329
O	4.54467119379244	3.38957749821384	1.13482580194373
H	3.60000172066696	3.35389161178792	0.83904300159112
C	5.05653219152458	3.17723492063625	-2.88658779471776
O	4.20102241092133	3.70993737867264	-2.18958822379817
H	6.12811710199469	3.33887686060373	-2.63805866945224
C	9.20832310066748	2.98750016691301	-3.15933799204720
N	10.34706343584785	3.20654569086321	-2.99366020465939
C	11.75578763725979	3.47925554870059	-2.81702551407251
C	1.21179591191893	3.48448939348095	-0.55995110678773
O	1.91395743510420	3.50072154167127	0.43440679395097
O	1.62371849204521	3.56412365693915	-1.80157923765510
H	2.62638731232386	3.59860246827579	-1.89929148482507
C	6.67766328843305	2.38269202358292	0.98325574306508
C	7.27136803316674	3.49389749520352	1.59992844111586
C	7.47894064243456	1.29949743694137	0.59457646724079
C	8.64925821132075	3.53181092136961	1.81253878488267
H	6.64700021265674	4.33465039910935	1.90422111111342
C	8.85594233719775	1.32359620726869	0.80399784456983
H	7.00389334460017	0.44019089657845	0.11808537409875
C	9.43002988430836	2.44757339653655	1.40427381221602
H	9.12035736046826	4.39553012637954	2.28417344746922

H	9.48407653683920	0.48476263106050	0.50068268941267
C	4.81266930164631	2.32918641835089	-4.05951410561466
C	3.51312261936859	2.05012576369786	-4.52643941327748
C	5.92952493126952	1.79168382301220	-4.72580439175426
C	3.33989119176165	1.24142308900098	-5.64539900801554
H	2.64717882611293	2.46601793234737	-4.00861127003815
C	5.74832293844145	0.98118874486511	-5.84672264257370
H	6.93346784026573	2.01732325248518	-4.35451929710222
C	4.45619449478867	0.70645843198148	-6.30402597334746
H	2.33358555713992	1.02005890100956	-6.00801781131751
H	6.61409026652739	0.56173873404035	-6.36416019988713
H	4.31330569123084	0.06990977542663	-7.18111270166311
C	12.42342747417265	2.21612238125299	-2.24650131887377
H	12.28197033458436	1.35868927732385	-2.92135360561191
H	13.50340154316837	2.39294768943717	-2.13029262306688
H	12.00667067342756	1.96679206186680	-1.25995810010077
C	12.34260729414618	3.83438239889563	-4.19405738238918
H	11.85082861858866	4.72533713403162	-4.61252172365295
H	13.41815521621852	4.04420875811023	-4.09450736751044
H	12.21446723237831	3.00112497889292	-4.90121185653751
C	11.89979717680732	4.65749039692094	-1.83829355939977
H	12.96535558954539	4.90392442942982	-1.71581547635241
H	11.37744334640476	5.54846594279729	-2.21760801327083
H	11.49024433312960	4.39700017785308	-0.85140509037540
Cl	11.16567408693698	2.50499926322543	1.63894178369688
C	-0.30184146184158	3.34352521520873	-0.48690644952227
N	-0.80282421564778	3.50964143106551	0.86856235863956
H	-0.78577062057606	4.07247984126207	-1.15039786279424
H	-0.58930802104668	2.32574290135781	-0.81910786529583

C	-1.22688356668092	4.68038070518482	1.47079622655807
C	-0.91484837360806	2.50900847691692	1.74812849460536
C	-1.61347235714992	4.35415722844523	2.73811519996088
H	-1.22948744747270	5.63230741865178	0.94865690585010
N	-1.41117324354854	2.99322393374855	2.88762874505845
H	-0.69282208973961	1.46909679143675	1.51830575026791
H	-2.02484328530169	4.96725460414035	3.53422882562346
C	-1.73788490607836	2.17516343491289	4.05576134019928
H	-2.57826392330775	2.63948391216278	4.58629804041452
H	-2.04415597722489	1.18434475030371	3.69119773622799
H	-0.86762252715983	2.10369798630559	4.72404058154210
C	-6.96650320089878	-5.27040497648229	-0.64888132714918
C	-5.83565840531068	-4.50158522700134	-0.83063140094619
C	-5.87886800166798	-3.08190408965337	-0.71539195272696
C	-7.13115139751748	-2.47864881924654	-0.36354745068057
C	-8.28444932174317	-3.29709141106599	-0.20480313567720
C	-8.21034221466895	-4.66505886733559	-0.34775606167486
H	-6.89945485567003	-6.35807224933124	-0.73333672605558
H	-4.88620579844181	-4.98773153453580	-1.05324731721875
C	-4.72252737788583	-2.24113660019864	-0.89740556137698
C	-7.19209647869111	-1.07646852905247	-0.15008263169587
H	-9.23297959181524	-2.81625972458952	0.05015781008831
H	-9.10140307206841	-5.28370741379874	-0.21487893444403
C	-6.06904890993670	-0.27636045964585	-0.20782172776922
C	-4.82983049764708	-0.89049527055000	-0.57402058627300
H	-8.15631132385078	-0.62716291555499	0.10105467527848
C	-3.42670597964794	-2.79982781989417	-1.38686467810736
C	-3.33964555264951	-3.53701297565770	-2.62134649478776
C	-2.26316827785432	-2.61827494560559	-0.64310077357931

C	-4.41254765684861	-3.62080938358414	-3.55479422251946
C	-2.10913837862817	-4.19297090978476	-2.95491568344076
C	-1.03177570366328	-3.27830502106723	-0.95244761045909
C	-4.28748795495265	-4.33615702993856	-4.72780095572828
H	-5.34500708665660	-3.09836534177759	-3.34059185483833
C	-2.01887904824979	-4.93989074428600	-4.16267610350106
C	-0.99690437800715	-4.07309975716377	-2.08014351119194
C	-3.08389413176342	-5.01611884986960	-5.03319623176358
H	-5.12501988762615	-4.37495803010383	-5.42906830816834
H	-1.07624831207089	-5.44408386575237	-4.39325187172248
H	-0.07715925656855	-4.60966566923229	-2.32625437995492
H	-2.99922781563399	-5.58763480551184	-5.96091411807288
O	-3.72907727603440	-0.08221951777559	-0.67227942840445
O	-2.29949919746648	-1.84003460769669	0.48359621909765
P	-2.45534110675183	-0.18638108134854	0.39703792527545
O	-1.28628451030586	0.45771569964172	-0.31341091013911
O	-2.83122954750059	0.27042214826474	1.78210541210624
C	-6.15066323345790	1.19029548844847	0.09678346029336
C	-6.01736608214228	2.14376681972876	-0.94468512721348
C	-6.39069001689982	1.62998801764223	1.42184503850938
C	-6.12904743915499	3.50585495392702	-0.64131393622375
C	-6.49741609142868	3.00716812566212	1.66836044282617
C	-6.37061091247794	3.96366280919182	0.65927130542653
H	-6.03505964180419	4.23207956853286	-1.45351745403047
H	-6.68940964580173	3.34775378774962	2.69016177126408
C	0.16861907172478	-3.13981218305705	-0.06251868750807
C	0.18138559534442	-3.76513067399022	1.20809382234884
C	1.30349885662322	-2.40797052992227	-0.49611347733870
C	1.32405891176600	-3.64742138922192	2.01108456077489

C	2.41684116414875	-2.31815622697437	0.34950925063369
C	2.45237292687206	-2.92893283040261	1.60717523523761
H	1.33685784957790	-4.13678265190842	2.98952332260837
H	3.28230287424181	-1.74177218248215	0.01346047145611
C	-6.50650146731646	5.44854279081484	0.97106805446496
H	-6.68087296609825	5.53003082887094	2.05851663332518
C	-5.21963598725878	6.22884052822586	0.65600677606456
H	-4.99131511120618	6.20692887770135	-0.42248065796722
H	-5.31743397171297	7.28639189611860	0.95331319182481
H	-4.35516584919558	5.80049859087458	1.18854555211189
C	-7.72004876201895	6.07596774420715	0.26571868263169
H	-7.60905298421316	6.03959783501662	-0.83051063915106
H	-8.64829802651773	5.54299354834230	0.52375532487023
H	-7.84083744871969	7.13361152963878	0.55362662657311
C	-6.54509562505725	0.66979884473342	2.60132699324630
H	-6.33742442294816	-0.34537127997583	2.23694759417400
C	-5.52294515901798	0.94780685552032	3.71572518956516
H	-5.62578711239045	0.19922018005939	4.51910861837168
H	-4.50262273797652	0.88232542810186	3.31208166644866
H	-5.67243380298384	1.94140656842937	4.17245596216026
C	-7.98296128134871	0.67626895619249	3.14881657672546
H	-8.25693945266847	1.66542002588946	3.55247223364778
H	-8.71757506620949	0.42450612304742	2.36751735522527
H	-8.09158388808691	-0.05747337311816	3.96466229014416
C	-5.79886592541641	1.73579088625175	-2.40080273287266
H	-5.69280251188526	0.64297687030210	-2.43340967920917
C	-4.50420782188978	2.32609350785985	-2.98231671706741
H	-4.54240211464568	3.42755768047749	-3.02808722981334
H	-3.63654708830326	2.02943267209161	-2.37476153656008

H	-4.34121850155927	1.95978255622441	-4.00929711670678
C	-7.01925975025941	2.08859298357046	-3.26927574061165
H	-7.93725078819076	1.62358597310341	-2.87627394927379
H	-7.18843316276890	3.17747487370188	-3.30957533898416
H	-6.87532544280672	1.73533861194838	-4.30387057530840
C	1.36948344081040	-1.71435745409867	-1.85620879776102
H	0.37291157946463	-1.77827404187539	-2.31447259387134
C	-0.99243511548610	-4.59561219065270	1.72552028215780
H	-1.79796015412478	-4.54482411711093	0.97998876528105
C	-0.61375706955410	-6.08083944126501	1.86354747056018
H	-0.25330120474495	-6.49277913608400	0.90749946149395
H	0.18210851162760	-6.22989380446495	2.61209743476096
H	-1.48587497369955	-6.67571585866119	2.18248200869763
C	-1.56444870039451	-4.03759664706912	3.03898490114641
H	-2.44661375908558	-4.62028243744120	3.35266128585577
H	-0.82758947476528	-4.08842137293780	3.85790252980103
H	-1.87430282919407	-2.98948036029884	2.91553261850273
C	3.67410156447396	-2.81612553367608	2.50996950875959
H	3.45035839272450	-3.39639384312819	3.42259693552220
C	3.93736130950311	-1.36288482343792	2.93935252457598
H	4.16423714682168	-0.72157398690557	2.07187798319475
H	3.05930790831376	-0.93488303907191	3.44818008797219
H	4.79332280343307	-1.30829074057880	3.63296547028960
C	4.92485368078204	-3.43820377769748	1.86746202741152
H	5.78054204638001	-3.40494924192750	2.56248821216827
H	4.75122527558902	-4.48945599035164	1.58844631109226
H	5.21687225824124	-2.89376733914543	0.95447467444348
C	1.70278759850950	-0.21910358270211	-1.73262064957167
H	1.67315053775633	0.25574449004140	-2.72750931642710

H	0.95633358762874	0.27492168627175	-1.09563257835002
H	2.70453629208821	-0.04099107328813	-1.31053031761007
C	2.35751074415597	-2.42418724467365	-2.79922508460907
H	2.36272353399134	-1.94298024655660	-3.79168580371576
H	3.38627269436777	-2.38261925778545	-2.40466163354417
H	2.09866183692527	-3.48608209298018	-2.93784288926234

TS1S

C	26.31056500678675	31.91671245559772	34.63216133719498
O	25.27206619155545	31.74516674631772	35.23080066353797
O	27.44679458086768	32.20173384901484	35.22209684058922
H	28.24451184047377	32.38113916896938	34.60277989008882
C	26.33163970842038	31.76826669901587	33.09212546682142
N	27.17940645320062	30.64784667761760	32.67886094021686
H	25.30102913981659	31.59067506787803	32.76004145822539
H	26.71948350234508	32.67519667537712	32.60896005479199
C	27.10594463201513	29.35563296771225	33.17075214674231
C	28.21124060629065	30.73068979835183	31.83112302978060
C	28.12954672995115	28.65989291525995	32.59951053105197
H	26.34374799976797	29.05508698175315	33.88385614441217
N	28.80453321542009	29.53701703249922	31.76848509717739
H	28.52375163566855	31.64494064574829	31.31331184372770
H	28.43064171592651	27.62244919732008	32.70620858377544
C	30.02841500553852	29.24763558100809	31.02526114854630
H	30.89734842005626	29.33826345840521	31.69357208200420
H	29.95280367287672	28.23592077165376	30.60857633326810
H	30.10128388537164	29.95998350292424	30.19602081609533
C	33.16666646870563	39.59463820610155	32.27241348107381
C	32.78838865623253	38.36123438416207	32.76116750385329
C	31.41903346821650	37.97152223777723	32.80334591786924

C	30.44393266351140	38.89054696070464	32.29272796913086
C	30.86480578092189	40.16237036447921	31.81309201178123
C	32.19686073840638	40.51344726567511	31.80385300227911
H	34.22583727331463	39.86274360883532	32.24347164040663
H	33.55039253544968	37.66471597736403	33.11102502897720
C	30.98031904765722	36.69261267146543	33.30051566620144
C	29.07568241603857	38.51060288468508	32.26308257152255
H	30.10567703531672	40.85451238238956	31.43850440657765
H	32.50775019489340	41.49084728706315	31.42665521360382
C	28.65464741327560	37.25084003689931	32.63807190533353
C	29.64546206553223	36.34175265210767	33.12307160345300
H	28.33866409371444	39.23539178164945	31.90849097255373
C	31.91821884704477	35.75917573356300	33.99490268959751
C	32.66513580006169	36.17743819501582	35.15463882995660
C	32.07410060383015	34.45011311109672	33.54846614316023
C	32.45193988908012	37.42459595512275	35.80928353234503
C	33.64495604589556	35.28950122709224	35.70808525398672
C	33.05326815894571	33.55536878864019	34.07999614224732
C	33.18822896208248	37.78474558524283	36.91890070688965
H	31.68256516457609	38.09864638627557	35.43335488510001
C	34.40145194261781	35.69846014829060	36.84180727221258
C	33.83302737383103	34.00910472401009	35.12465380131449
C	34.18402908782492	36.92163750143767	37.43617144509079
H	32.99677313941947	38.74392694977701	37.40641791846384
H	35.15267900423632	35.01218850224964	37.24219757068106
H	34.60997680799424	33.35797818188017	35.53226894237070
H	34.76553165791019	37.22162326163197	38.31139391866862
O	29.22379866396881	35.09141792833218	33.50153286055814
O	31.30286109265201	33.99831040985791	32.50167246555302

P	29.68590066369824	33.71856846226710	32.70261304273409
O	29.44527632379704	32.60161751897716	33.70739456889261
O	29.08883337950807	33.58808258816751	31.32847234560705
C	27.20680135439022	36.86103561149378	32.57363184332395
C	26.45711877757264	36.73164998065381	33.77211058550351
C	26.57587665041391	36.65383809316799	31.32362583197414
C	25.09846087865320	36.40551673282439	33.69080029724181
C	25.20847763569567	36.33858526285908	31.30176324619272
C	24.44893512899036	36.20827479634518	32.46594867494681
H	24.52623740190452	36.31137656242441	34.61671498513464
H	24.71630672926987	36.18599751174028	30.33703452942736
C	33.28799885687271	32.18982489403383	33.49861396320919
C	33.98218035477606	32.05984270544356	32.26922694785513
C	32.89007812922364	31.02845978511622	34.20539173955626
C	34.28552280347832	30.77969868169313	31.78836871505645
C	33.22378742653166	29.77039603786864	33.67979979775451
C	33.92796563788973	29.61806401996119	32.48181482000447
H	34.83641199118818	30.69281293987319	30.84830135184147
H	32.93458115900547	28.87286362516955	34.23406649289939
C	22.97051861135154	35.84818637148781	32.39537362816725
H	22.69247600840400	35.86118536922252	31.32679632656222
C	22.70741210228720	34.42644893007105	32.92070109181213
H	21.64118973154177	34.16170566954356	32.82463548191878
H	23.29284109388044	33.68065085080694	32.35948647800372
H	22.98237853000010	34.33082841221665	33.98379104397615
C	22.08075498922807	36.87761438654930	33.11024201607527
H	22.25142681926151	37.89318133102642	32.71979035312839
H	21.01442746501326	36.63323107601612	32.97354770466341
H	22.27690858665845	36.89837143512889	34.19466843847231

C	27.31913781331588	36.76485135976656	29.99278166520844
H	28.38989011525852	36.87420123082927	30.21616959341922
C	27.17592398363208	35.49686462683997	29.13583912348565
H	27.54008925452585	34.61627679053035	29.68443987216617
H	26.13074652502275	35.32090225111174	28.83206930920237
H	27.77343350908011	35.59162246440012	28.21443912805305
C	26.88367672446626	38.01538433018238	29.20818611941465
H	27.46293363851506	38.10957717108260	28.27472010712568
H	25.81624434920664	37.96557877783538	28.93564323830472
H	27.03173722678980	38.93674089116091	29.79347500575666
C	27.06665403538664	37.00906449091396	35.14588429567336
H	28.15895332547888	37.03734114355004	35.03301096567876
C	26.75815883010782	35.91501459968562	36.17932667361652
H	27.29989233920881	36.11681632543398	37.11757457764917
H	25.68580845290024	35.86931361239766	36.42938150394065
H	27.06558939075739	34.92089380561228	35.82198313154175
C	26.63555627453655	38.39625968631410	35.65699598983981
H	27.11152690335137	38.62093297326366	36.62600905232661
H	26.91610829176506	39.19091305625192	34.94751189784505
H	25.54317758229910	38.44570397285139	35.79873233041239
C	32.13196492984130	31.09230822685190	35.53082951167470
H	31.78657139649691	32.12679791705560	35.66580897020683
C	34.44013582901874	33.27087936849504	31.45801280445484
H	34.13486065212633	34.17547685374507	32.00174383602230
C	35.97210228776214	33.32755624185042	31.33110052686015
H	36.36748577700709	32.47240458765575	30.75836076506246
H	36.28262088544867	34.24707046034056	30.80814304905340
H	36.45667912721353	33.31893385803748	32.32027386888239
C	33.75839215696540	33.32569788377438	30.08120579662127

H	32.66390695624471	33.33909800238717	30.18682312583234
H	34.05957319879765	34.23554585023373	29.53600220263776
H	34.03234689687415	32.46010583024750	29.45521476868208
C	34.33251522373174	28.23323093646933	31.98996650641455
H	33.78433918844951	27.50249837452059	32.61074311898071
C	35.83476101853271	27.98058345133505	32.20823956057176
H	36.11182003801873	26.95587795640569	31.90889888457135
H	36.44433776428338	28.68176124760668	31.61445602643446
H	36.11051268118794	28.11347452503147	33.26568287694536
C	33.94079267981628	27.97605496945411	30.52679439610209
H	34.18011175452130	26.94010883166230	30.23485819151283
H	32.86255285901329	28.13456818266977	30.36620830921394
H	34.48291307647974	28.64491290975888	29.83850898792964
C	30.87652977754441	30.20523133079048	35.53607319130619
H	30.30085299343681	30.37022018753073	36.46072479936912
H	30.22319391608490	30.45674665568377	34.68894072585034
H	31.12716019282503	29.13212002408015	35.49340978861875
C	33.04671606478901	30.74434784959952	36.71896210462418
H	32.49627266866369	30.83039185924714	37.67014991676334
H	33.42375357044924	29.71054311515552	36.64275618772098
H	33.92040311391820	31.41187166456742	36.77359704689203
H	32.04588096426354	26.17935345065686	25.10756626302398
C	31.59871460610347	27.17686174299864	25.22736526738291
H	31.15483318512511	27.47523849147977	24.26574684833421
H	32.39937928641908	27.88696627933567	25.48268797009909
H	29.78212776366905	25.14891707038367	25.86717903483513
H	31.57274225264721	25.73198988301021	27.62307502188978
C	29.38335512171626	26.16915359598159	25.96756270483497
C	30.53483127928168	27.12036253134920	26.33446400607333

C	31.14725551929067	26.74401788258429	27.69317458613057
H	28.92341326487433	26.45678894065816	25.01022163077407
H	31.95336023608620	27.43923708493470	27.97103318260952
H	28.61852198614761	26.17591549745142	26.75713557949937
H	30.37104344533923	26.75307135755429	28.47098592511800
N	29.96647301009767	28.45178887452168	26.46687052169214
C	29.47873047328909	29.49539568647390	26.59560121399389
H	30.95236812018120	34.70361784451320	28.53808937078604
H	29.64958350049035	32.67329546989830	29.07932363248652
C	30.61090300300899	34.05669056309125	27.72703506691096
C	29.88393617916962	32.90145077734202	28.03836240205807
O	28.02135171081843	27.22575167281114	29.04577459027344
C	30.88569291232093	34.38180460881060	26.39754929420861
C	29.43016907369583	32.06947199462706	27.01080233412751
C	28.59723051165325	30.84195882270348	27.34642321938770
O	28.43888680390280	30.51504921655951	28.58066109627347
H	31.45446181610270	35.28419909191632	26.15929228262845
H	26.95122812417113	25.01693990715732	29.58345098144979
C	29.69429962133119	32.40526836337420	25.67497769683724
C	30.42281441521830	33.55356847838155	25.36735488784592
C	26.89967293703196	27.68129268025574	29.25751187007568
C	25.94988611610150	25.41673019931166	29.75348946702683
H	27.40885248138978	29.58123185872426	28.91987545193835
O	26.59704383180024	28.95529370881067	29.19108012663158
H	27.70178654096612	30.77804031009696	26.68672473932447
C	25.74384237795824	26.79823113448436	29.62411688097717
C	24.89819455622710	24.56819587368205	30.09295911772791
H	29.32823790754060	31.76139335252503	24.86772346241424
H	30.62718815161540	33.80758221587191	24.32413478931991

H	25.05258564352158	23.49298481771583	30.19537331562439
C	24.45993506287171	27.32250070648726	29.84024303271309
C	23.62522435428513	25.10967274713677	30.30394888225356
C	23.39894691529312	26.48415795187170	30.17960560972062
H	24.29922155702134	28.39627107995532	29.73749585164424
Cl	22.30206613781398	24.05532494880635	30.72768320613967
H	22.39868874494183	26.88587739592690	30.34815013556234

MS1S

C	-6.52426059757825	0.02547553650539	1.19653862336355
O	-6.64387797112296	0.71083990290181	0.18446236169457
O	-5.98848141552337	0.46559010368602	2.31379194545543
H	-5.62703363610306	1.42631639350543	2.20398642757924
C	-5.83052902298951	3.61742942901781	1.19313187459785
O	-5.08292377727089	2.83411665922385	2.01592349691652
H	-6.90844614044878	3.34626078225166	1.24893612302318
C	-5.49389611398306	3.29058137422922	-0.23995256487643
N	-5.20485093219810	2.96436356081199	-1.30311575761955
C	-4.75918856228441	2.42305136832602	-2.57583914710072
C	-2.03218785207717	3.30287589253463	1.06515117965160
O	-2.55679575881250	3.58880729467356	-0.00160632864010
O	-2.63922288727867	3.00531855913834	2.16883534587822
H	-3.72881628001400	2.96157505066503	2.06316404603203
C	-6.98408540107718	-1.39717649378196	1.25514633144839
C	-6.80933054894181	-2.17859339960597	2.40773834456812
C	-7.59985905204476	-1.96349493856815	0.12914874927085
C	-7.24176527984172	-3.50384255253628	2.43691784219668
H	-6.32833986917491	-1.73922102447829	3.28235321307967
C	-8.03514138825239	-3.28676661264946	0.14576002489382
H	-7.73312961464790	-1.34787981054068	-0.76210540373154

C	-7.85238288696620	-4.05052272054549	1.30397348766373
H	-7.10851530150563	-4.11701744692565	3.32932321604816
H	-8.51432481861828	-3.73262925661303	-0.72703572837644
C	-5.71829939965044	5.13485020711246	1.40881301538317
C	-4.98817057417013	5.62837443965695	2.49311110784098
C	-6.38244644375976	6.02891642345794	0.55790484918390
C	-4.91572414512097	7.00725991922398	2.71671490234342
H	-4.48874940351931	4.92678116115311	3.16218107336013
C	-6.30557460110782	7.40486087870755	0.77976582833968
H	-6.97196976910113	5.65231442158568	-0.28470059655116
C	-5.56877415199058	7.89749657418431	1.86169128030726
H	-4.34582067345153	7.38582059265240	3.56899798265005
H	-6.82619272949648	8.09395578366063	0.11023914856498
H	-5.50906498646894	8.97409197635573	2.03926101311224
C	-3.91589043248827	1.17901685664129	-2.25136396587453
H	-4.51607620396877	0.43740242966824	-1.70627261711625
H	-3.56751878304514	0.73049516702129	-3.19271371680576
H	-3.04291308264820	1.44592448468695	-1.64056157450875
C	-6.01656871896760	2.05711598941707	-3.37965488361498
H	-6.62879603234165	2.94759562668627	-3.58822959761516
H	-5.70896009677441	1.61567730007921	-4.33869512768598
H	-6.62607573559993	1.32393714181244	-2.83208760307769
C	-3.92334257352905	3.50814187781105	-3.27110922650736
H	-3.54271418803014	3.10631100429226	-4.22151580783840
H	-4.53116292271693	4.39928333897705	-3.49005213872770
H	-3.07313940793944	3.80091636083701	-2.63869323219978
Cl	-8.39362055770304	-5.70852523870724	1.33303676700223
C	-0.50796179384472	3.24932018463077	1.22278047370915
N	0.18778427113459	3.74557718405714	0.04445402565381

H	-0.20226456574394	3.82679205749585	2.10656139977089
H	-0.20499439766865	2.20301592311996	1.38315070684940
C	0.33275742122289	5.06391335099871	-0.34279442158607
C	0.73552979868685	2.95386135644448	-0.88618390191998
C	1.00411935626180	5.05213385811023	-1.53105017656954
H	-0.04980977928396	5.88851286474627	0.25101380639951
N	1.24690605853959	3.72632059263182	-1.84895205069845
H	0.76211235365156	1.84492650195425	-0.80358873606478
H	1.33476477355720	5.86769660149717	-2.16712083087702
C	1.97285497923490	3.23056543384684	-3.02135717140037
H	2.93763732458320	3.75073842588846	-3.09020253709392
H	2.15725744280506	2.15198014748209	-2.88688196690557
H	1.37955822686668	3.41498024884303	-3.92839550986416
C	7.01765600569427	-4.97201793000970	0.41312506921004
C	5.80715667388033	-4.32166431029676	0.53371967387385
C	5.72140355759482	-2.90121067070218	0.46605232467945
C	6.93096266089678	-2.16705091582692	0.23442387511486
C	8.16631934233661	-2.86611605713939	0.13371135904643
C	8.21459578181448	-4.23980668144964	0.22387203571359
H	7.05177248357037	-6.06350910400486	0.45884945792883
H	4.89512745232619	-4.90264439737419	0.67026887782007
C	4.47957246837596	-2.18227205779917	0.58522026707612
C	6.87094613418761	-0.75578649928903	0.09098818229380
H	9.07994484610172	-2.28826635355592	-0.03144493960263
H	9.16861380860733	-4.76581818389301	0.13738840873212
C	5.67416355149392	-0.06764100318887	0.09686097455405
C	4.47540208988527	-0.81510872367849	0.32273309998971
H	7.80387407327583	-0.20499343551833	-0.05420468263043
C	3.21196713755414	-2.87129575119556	0.96931471949591

C	3.11198111421695	-3.62666232461778	2.19144934158695
C	2.08769363351767	-2.77836103520591	0.15216017681691
C	4.13042982025677	-3.63398556366244	3.18792190509873
C	1.91935209578574	-4.37894032350405	2.44806617874735
C	0.90042318283761	-3.54219087874660	0.38046834382885
C	3.98959879896748	-4.36366500438363	4.35012810389587
H	5.03092256572467	-3.03993627087923	3.03237834219926
C	1.81278045675370	-5.13689959450996	3.64758862600079
C	0.85949735994525	-4.34247003321953	1.50465078896708
C	2.82507782416189	-5.13484834789354	4.58175442916014
H	4.78346598034656	-4.34193683084046	5.10115056536852
H	0.89974986764941	-5.71356307181476	3.81974790419673
H	-0.02575877910811	-4.95587481150052	1.68940867259275
H	2.72765971578635	-5.71453920182001	5.50305587912650
O	3.29221167096092	-0.13009987889821	0.35417174841627
O	2.12934428044897	-1.97990654041236	-0.96276309646870
P	2.12699675084449	-0.32370099564038	-0.83301539888948
O	0.85425848605094	0.18961958617851	-0.19300547748866
O	2.58732766535238	0.23373164108328	-2.15056184515904
C	5.64160240920521	1.41991622063585	-0.09566462342105
C	5.33621898961159	2.26848583906522	0.99899700099068
C	5.95721781998077	1.98777226328235	-1.35514714919238
C	5.36359656007887	3.65668802335387	0.81596233857516
C	5.96666788039102	3.38562716929386	-1.48275084964409
C	5.68146087298277	4.24137876476267	-0.41521774569050
H	5.14779146716743	4.30019320834123	1.67268390762619
H	6.22352281410807	3.82568312521507	-2.45129284276070
C	-0.25124530528577	-3.53548327512374	-0.58258844841863
C	-0.13257193342342	-4.20231720910079	-1.82608092849295

C	-1.48414978862480	-2.93381371339843	-0.22251245431202
C	-1.25300585863887	-4.27788184128360	-2.66466801161784
C	-2.57386184103824	-3.04630545478403	-1.09648319734890
C	-2.48688662585333	-3.72205518215251	-2.31841870258171
H	-1.16581912089039	-4.80408681590850	-3.61988102860942
H	-3.52585982154782	-2.59507405350235	-0.80418536058028
C	5.78869420380173	5.75228261981179	-0.58250059520836
H	5.81957920178134	5.95126515651521	-1.66870555715325
C	4.58481623972208	6.51516086628723	-0.01019488942214
H	4.52267276323067	6.41337549618911	1.08559339498572
H	4.66406900079845	7.59192461730613	-0.23328887828079
H	3.63559611089225	6.14624650963344	-0.43066949818267
C	7.10520342852368	6.28085512885421	0.01496384456818
H	7.14064645094011	6.10805607480450	1.10335844582388
H	7.97532774702787	5.77372322185615	-0.42962378273038
H	7.21308595562148	7.36460666743268	-0.15902793068693
C	6.31173651952732	1.14373615260795	-2.57999604002659
H	6.18446292149588	0.08789372349276	-2.30617186723825
C	5.36897918861804	1.40059969052487	-3.76683491395097
H	5.65068129609146	0.76056299886721	-4.61929134719778
H	4.33345557128715	1.15753852090996	-3.48903585275020
H	5.41989060919780	2.44704032189234	-4.11395186602707
C	7.78374090938242	1.33866221884607	-2.98512769315283
H	7.97906914572377	2.37222537031992	-3.31697969635281
H	8.46811983155886	1.12678448832050	-2.14821535623823
H	8.04825371278945	0.66684913928467	-3.81823530621014
C	5.02424078399549	1.72552072152728	2.39302766219026
H	5.01197645464773	0.62914754401178	2.33498712902901
C	3.63427041227986	2.15785524870776	2.88792754418437

H	3.56862748816925	3.25137534652296	3.01848614116826
H	2.85582049327247	1.83917560894783	2.17851771148336
H	3.41213037050673	1.69565521712104	3.86377250962373
C	6.12369683303371	2.10359888835391	3.40131730898530
H	7.11133335773608	1.74876369655687	3.06682230148236
H	6.19096179640563	3.19529838901644	3.54101059588225
H	5.91730048411014	1.65460509489317	4.38690644600444
C	-1.67360879704291	-2.17444784514516	1.09097853286494
H	-0.68436924756420	-2.06403209413540	1.55640795725186
C	1.16314392031939	-4.87255034392722	-2.27994296502048
H	1.91537097157158	-4.72343649975701	-1.49314016692305
C	0.98972541498673	-6.39269008205590	-2.44259484187912
H	0.63659447945863	-6.85826572896135	-1.50858212942131
H	0.26127011686338	-6.63761963881124	-3.23303741891782
H	1.94772071677307	-6.86450542578134	-2.71724787851468
C	1.72248061831285	-4.22868474354605	-3.55970055838520
H	2.68650442006529	-4.68967365363008	-3.83168413067747
H	1.03849473656813	-4.36283956892054	-4.41439391128119
H	1.88840389312710	-3.15109847913132	-3.41497095508611
C	-3.68820633490137	-3.85754687407851	-3.24575350724738
H	-3.36602381231444	-4.49020722598487	-4.09120430581241
C	-4.12109246457069	-2.50329767256387	-3.83156641555524
H	-4.46959767101398	-1.82233212025292	-3.03803888112099
H	-3.28569162998092	-2.01072200546224	-4.35423258475448
H	-4.94837417940300	-2.63080682521150	-4.55008663211353
C	-4.87078022445947	-4.56635571284388	-2.56533745067895
H	-5.69720438966376	-4.72265456341321	-3.27872482580994
H	-4.57277761908624	-5.54891825759226	-2.16764451171835
H	-5.26632120501306	-3.97341470087201	-1.72442700265544

C	-2.21268102753757	-0.75181209778124	0.86866152882625
H	-2.27508188246511	-0.21261757236743	1.82852814374773
H	-1.54025320155083	-0.19326600904840	0.20120772025872
H	-3.22671645626967	-0.75920259108527	0.43587341123582
C	-2.56764893188966	-2.95381282797199	2.07198149552966
H	-2.65164492483869	-2.41699323586897	3.03144458067465
H	-3.58721669672013	-3.08188496418975	1.67165806367270
H	-2.16554769790579	-3.95781799245786	2.27958618671077

TS1R

C	27.91420838314445	28.63682725425477	33.26113970169905
O	26.79843285427154	28.86196598102929	33.68355218610225
O	28.97053096644088	28.30591026801638	33.94735101207865
H	28.87105413984730	28.47394218532878	34.98926573139502
C	28.16880208999042	28.70375322341672	31.74463667456517
N	29.56728910669515	28.89334255487994	31.37939193475859
H	27.80484705590839	27.77342177088378	31.28353509061832
H	27.57776358352437	29.53338703416921	31.33706219736792
C	30.53455752260018	27.90760389946784	31.30017274048830
C	30.15119481791115	30.08676516442199	31.20010191107053
C	31.71924817063062	28.53432324602213	31.04952262416307
H	30.30064388394485	26.85806724013015	31.44840967181013
N	31.45484138574230	29.89085172524157	30.98963792295115
H	29.69309145688661	31.08847050137339	31.25935963252790
H	32.72073984270544	28.13664740066577	30.91807603058036
C	32.43485551323753	30.95792893018372	30.77320871810038
H	33.18283488535852	30.93607134741985	31.57688169999434
H	32.92357051711386	30.81311745704794	29.79986464265099
H	31.90445397297141	31.91798958364310	30.79645575630524
C	32.04327605057738	39.55539934579904	34.15446944781464

C	31.91776813942469	38.20259052538377	34.39430967220353
C	30.72561925515316	37.49885239654157	34.05703188580535
C	29.66997422657751	38.23604240377956	33.42688925935904
C	29.82355143210907	39.63353068673109	33.20828062686275
C	30.98292217087911	40.28571723449883	33.56596980064726
H	32.97265791152397	40.06661882810418	34.41819520460900
H	32.74949250521201	37.65816089727514	34.84101020083823
C	30.54974167872293	36.08780914481324	34.28819872678647
C	28.49316312650798	37.55393599199866	33.02050825278901
H	29.00193167687998	40.17981368905281	32.73683487798240
H	31.09136776935711	41.35828965645308	33.38704656568603
C	28.35076074659212	36.18777563974567	33.15344178586243
C	29.41725891471384	35.46399851166758	33.77344737583557
H	27.68137063573865	38.13270778804713	32.57277420395160
C	31.57475825485852	35.29685991476282	35.03210282092858
C	31.97766969743695	35.64933789429013	36.36819293226373
C	32.17543469982319	34.19554002923670	34.42672506000475
C	31.29751335975787	36.62765971224560	37.14927616629471
C	33.08494158311550	34.96277884546014	36.96613382580467
C	33.32468061154852	33.54626437268490	34.97984893175266
C	31.70100505972719	36.92208000952457	38.43549266410663
H	30.43404050368287	37.13978302034953	36.72401724629702
C	33.48765422460168	35.30195314270179	38.28791150703216
C	33.75934503569736	33.95686993777136	36.22396953071908
C	32.81367376363353	36.26182857756427	39.01151228924708
H	31.15404603173431	37.66920423099506	39.01609461864363
H	34.33967140815186	34.77402399582915	38.72493505842086
H	34.64432085091845	33.48855197061511	36.66204229517960
H	33.12853419367608	36.50857663459267	40.02849087512013

O	29.26530505425428	34.11135985516446	33.93486537335534
O	31.70049177017682	33.75165169752671	33.22182289472728
P	30.20447623041682	32.99304778971860	33.13492661969420
O	30.17129812455117	31.74308663711543	33.97155824524136
O	29.89252008269416	32.93645207372524	31.65767818092491
C	27.08877930362233	35.50665795289313	32.71045571721777
C	26.11621143935625	35.14286061697564	33.67441978273762
C	26.83926466793653	35.27685268931730	31.33663873060668
C	24.91710398185500	34.56218657455070	33.24544654361232
C	25.61839480303360	34.69662786415297	30.96164122089724
C	24.64350894907995	34.33086238476641	31.89264581798273
H	24.16892370751410	34.29032914834781	33.99511189864872
H	25.41822633148097	34.52218914930019	29.90071381925328
C	34.10398589703028	32.52198552357876	34.20464269146526
C	34.93739605087084	32.94675560424913	33.13613639208487
C	34.08100034249844	31.15571832457841	34.57470740436121
C	35.73476246007612	32.00255028626839	32.47666231344642
C	34.90636394812145	30.25364776946767	33.88304087755653
C	35.74600398684773	30.65005586664246	32.83905917750440
H	36.38368344976569	32.34139305633971	31.66441809526026
H	34.90268118111942	29.20143681971645	34.18119297539425
C	23.32818996420794	33.70977012071658	31.44086393547423
H	23.37895033236223	33.62351529704920	30.34133692341722
C	23.13423875412692	32.29177068021635	32.00237146721440
H	22.21774177246915	31.83094207029791	31.59774793256663
H	23.98616539409153	31.64039895086463	31.75205271763205
H	23.04001220993753	32.30739292257972	33.10081670831082
C	22.12378233093128	34.60563683018029	31.77442025554695
H	22.23941018552663	35.60988339141801	31.33773580555619

H	21.18877254949708	34.16890333136590	31.38560672221484
H	22.00580316041953	34.72835978483719	32.86376203631842
C	27.83909645370143	35.66445464534485	30.24783159582179
H	28.78881385207933	35.91227881521877	30.74309191608398
C	28.13915282286593	34.51071871369239	29.27843708364711
H	28.51670899575344	33.63871153395017	29.83025557018076
H	27.25018873562393	34.21678707741854	28.69602553053718
H	28.91403916284191	34.81849818706485	28.55664674362641
C	27.37326148937642	36.91769735148094	29.48479632309623
H	28.12503945885632	37.21900156720250	28.73648034926865
H	26.42585965502842	36.73096448475204	28.95162045393283
H	27.20900700622411	37.77102334040494	30.16124292587837
C	26.31889396928856	35.39527112904378	35.16760713886866
H	27.31498746305209	35.83957517001393	35.30173124536951
C	26.29833428763451	34.09215886043450	35.98177921723124
H	26.50941550917767	34.29435052710338	37.04486024335110
H	25.31463010208286	33.59684366733454	35.92652134493582
H	27.05700634905541	33.38621230818337	35.61205708314142
C	25.30241578273977	36.41305291883196	35.71356122437081
H	25.50370212704484	36.63232603235877	36.77532141596072
H	25.34672353701861	37.36210526180638	35.15628643726917
H	24.26965036890584	36.03330207397480	35.64308677194755
C	33.21403278984946	30.62703269058478	35.71593972466576
H	32.53605844791532	31.43633526347725	36.01998428032920
C	35.02348574519677	34.40720796222620	32.69236187712893
H	34.37494930540388	34.99968996834249	33.35178102415871
C	36.44639256595601	34.97041906078796	32.85164329649669
H	37.16599304258779	34.46182108552144	32.18911209969303
H	36.46649480592740	36.04301116629452	32.59818094474839

H	36.80798141043184	34.85899924213134	33.88604132538941
C	34.49955351797199	34.60204575765685	31.25961567188602
H	33.45664588326387	34.26169355755053	31.17419549907078
H	34.53051715485980	35.66754773117600	30.97860231859682
H	35.10972834728652	34.04882980956782	30.52582091557746
C	36.65368940462742	29.64539037452420	32.13973361341083
H	36.47885150048041	28.66831197018239	32.62337519310854
C	38.14069688728318	29.98898735432286	32.32745207650078
H	38.78274357927639	29.21621145943680	31.87346345711424
H	38.39253444957241	30.95163612987894	31.85316179158748
H	38.39989265646439	30.06548576320727	33.39468573627823
C	36.31109472404968	29.48583345668501	30.64934894063291
H	36.94589145793774	28.71705104841583	30.17895251521852
H	35.25948391893152	29.18684623673332	30.50851533304585
H	36.46496368370482	30.42716223176029	30.09647528873010
C	32.32786215604449	29.44900798577448	35.28249887657679
H	31.68915213604776	29.12504679090544	36.11871145621526
H	31.66234420497710	29.74535607026514	34.45909297982606
H	32.92360864117376	28.57483301008805	34.97121076491572
C	34.06968808019965	30.25111419102311	36.93910015842987
H	33.42697770328856	29.92590590520007	37.77318274681906
H	34.76021333292006	29.42349993326622	36.70512701152528
H	34.67707910186009	31.10003964104571	37.29043680140428
H	29.38010906705317	30.47971559647020	41.12608897080885
C	29.49285688307505	30.94021522528994	40.14133317368951
H	30.42459284403159	32.73396371082778	40.91508274127445
C	30.07613985358665	32.20738845681082	40.02269933893130
H	28.57934668694298	29.28236784356697	39.09819939952122
C	29.05123373520897	30.26190349052323	39.00493679533601

H	29.46809779789424	24.52669622875806	38.52780435880255
C	30.21343204677224	32.80074388434520	38.76492610840948
H	29.30060577147097	22.31553207064047	39.68822014147088
C	29.19034473421568	30.85306575399272	37.74040870749187
C	28.58925811797725	24.28442730893953	39.12651145221010
O	28.86087217065436	28.87990602455168	36.36905278433043
O	28.72118715144384	26.74124795898630	37.85516476143410
C	28.49938567928021	23.05139153184420	39.77143436497109
C	29.76804837458371	32.12600444733082	37.62451444087892
H	30.67217664432860	33.78735378076527	38.66744539873392
H	28.72273123933451	27.63020870416827	37.37567975377240
C	28.76715730233968	30.15816756726266	36.48024329818523
H	28.96282882599771	30.75673822757796	35.57571837451661
C	27.61505797773196	26.55527131979794	38.55658743520098
C	27.55366605297548	25.22448501401931	39.23954445635955
C	27.36434176322330	22.75880726055444	40.53383997941175
Cl	27.24446138980287	21.21593102528605	41.34138475795402
H	29.87547360045602	32.57574545218178	36.63413815093917
O	26.72288823984485	27.38395844691047	38.64820156834154
C	26.42458268749587	24.91281401272736	40.01116203511064
C	26.32269844718031	23.68418380226617	40.65932683500655
C	26.84602973065919	30.46390356507473	36.51329287757594
H	25.62600732209557	25.65212204048644	40.09420078910901
H	25.44668831096843	23.43601383139225	41.26039888171027
N	25.73105235104360	30.17786486205071	36.65785452255359
C	24.36484791900363	29.72103307679323	36.80849981560247
H	24.93412104404606	27.62441129592100	36.66595792679863
C	24.27510910243322	28.33490044254180	36.14703686959077
C	23.45070135728027	30.73735665015165	36.10652860687079

H	23.54677885666692	31.73475509883642	36.56126502440588
H	24.76340726031154	28.94176609802915	38.80239465386011
C	24.06729618726695	29.63856163510294	38.31455930323396
H	24.15564808209867	30.62796805084451	38.78854825408099
H	22.40498388392703	30.41020158728451	36.20388530547284
H	23.03825600072381	29.27595755753851	38.45814126509309
H	24.57512612322959	28.38789774709858	35.09092929013574
H	23.69630813576342	30.81283379218740	35.03701758554222
H	23.23779140060376	27.97322914858483	36.21289624736368

MS1R

C	6.06881976604960	-0.06913959832414	-1.10801442600688
O	6.01985969502857	0.54717517280596	-0.05355348472615
O	5.79504737904898	0.48435183469732	-2.28151617777707
H	5.52087674538936	1.43899438372148	-2.16624992558687
C	5.46508787089608	3.99786960957999	-1.22419776588456
O	5.03222113229351	3.04265149800911	-2.11947788553974
H	4.85813441858427	4.92042704404219	-1.30106215490785
C	5.20541031810705	3.52641005403150	0.17765510580636
N	5.06303919430962	3.12997429412491	1.24601497049107
C	4.71175523716926	2.55456926516212	2.53482154581258
C	2.03664371295968	3.62693176447305	-1.28776460979618
O	2.59111511182938	3.95866075288425	-0.22664410752623
O	2.59745491155186	3.33551049304339	-2.38594291866119
H	3.97243329609586	3.16086799922167	-2.27865040850760
C	6.44612587167950	-1.51011988290919	-1.19436179507313
C	6.48784690575856	-2.19012189702025	-2.42143700090678
C	6.76181119429137	-2.20076515125788	-0.01439018723737
C	6.84274967207079	-3.53717564218583	-2.47143880440307
H	6.23699676857918	-1.65679399019201	-3.33892879331369

C 7.11481155580463 -3.54741227323754 -0.05165159617535

H 6.72519825002223 -1.66539680924929 0.93584820521635

C 7.15436762062891 -4.20845874899551 -1.28474889572324

H 6.87681222315258 -4.07229247818723 -3.42142291934402

H 7.35865103380206 -4.09052136212780 0.86251937262603

C 6.94397918580987 4.36265000229215 -1.35880505464224

C 7.45057206339410 5.45905554403487 -0.64663415420381

C 7.78987073300878 3.62652604948253 -2.19352476207853

C 8.79661339845611 5.80966122061596 -0.75860317094199

H 6.78857585282950 6.05141126253044 -0.00663852878249

C 9.13687441417295 3.98394646432763 -2.30764568174575

H 7.39087341863427 2.78632894539811 -2.76201517345153

C 9.64320938796055 5.06990602320178 -1.59040260928482

H 9.18349665107135 6.66744206649787 -0.20331020891413

H 9.79265871458693 3.40761192430582 -2.96453370586091

H 10.69654419562987 5.34528011814334 -1.68277647265224

C 3.62103933980877 1.50507387760557 2.26221753586315

H 4.00579449886918 0.71107337831139 1.60879913756477

H 3.31216704720264 1.06027697120192 3.21911950477469

H 2.74747863349158 1.96574703376456 1.78027086479688

C 5.98144459138126 1.91866129432788 3.11935324175660

H 6.75946664503019 2.67604298394309 3.29963085728351

H 5.72929830620916 1.44446403749883 4.07901402668428

H 6.37461527598952 1.15441310338205 2.43467931242285

C 4.18384917699825 3.70233156899171 3.40999702067825

H 3.88977527031936 3.29215846242024 4.38700472188778

H 4.95678056103818 4.46836201905113 3.57548334608411

H 3.30498712924239 4.17291279369591 2.94544228571452

Cl 7.59984107700656 -5.89255056067782 -1.34046565394244

C	0.49896969056424	3.52746909016976	-1.34283364569302
N	-0.14881874300719	3.96699193321304	-0.11234340164603
H	0.12247072894319	4.12209845493656	-2.18713379226856
H	0.21716909319059	2.47957125845697	-1.52059945990762
C	-0.25836112546882	5.26750131627411	0.33570133567888
C	-0.66709982005257	3.14296347071933	0.80682445892931
C	-0.87535893924929	5.21581274497077	1.55192976255565
H	0.10836679090295	6.11011330780686	-0.24200727132918
N	-1.12275822852435	3.88192473277048	1.82555152605877
H	-0.71661025560709	2.03732511396002	0.69769808636873
H	-1.16644484860727	6.00781241409012	2.23534767610944
C	-1.77655491797048	3.35004594465343	3.02345972877713
H	-2.65918601041447	3.96298403018743	3.24927652964031
H	-2.09878963134913	2.31538566374704	2.81693773689762
H	-1.07703722077243	3.37979761437181	3.87183707175236
C	-7.26995759821390	-4.58356778331486	-0.46045099498163
C	-6.01278567936315	-4.02002738715687	-0.53053401995006
C	-5.83794118680940	-2.60773613818662	-0.60189028695972
C	-7.01204761004028	-1.78612477843731	-0.55939950894113
C	-8.29570632552985	-2.39835213846652	-0.50784404161596
C	-8.42787988371464	-3.76869328452082	-0.46331094382412
H	-7.37213762485252	-5.66995121664394	-0.39719168604052
H	-5.13391793338896	-4.66439398414328	-0.51825728180464
C	-4.54552243839457	-1.97626294251003	-0.67925734291030
C	-6.87109357208385	-0.37381878362709	-0.54126770460323
H	-9.17937413003565	-1.75436587731037	-0.48861932798958
H	-9.41874725492349	-4.22718875151664	-0.41526014551593
C	-5.63596708801010	0.24114231356134	-0.49586150740776
C	-4.47380063547639	-0.59130420398651	-0.55183499235701

H	-7.77430903285314	0.24145158017275	-0.52789098196295
C	-3.29904202653897	-2.77391673441564	-0.87421644218165
C	-3.15533436843864	-3.67783706623238	-1.98709332947409
C	-2.23793051055528	-2.64964784480328	0.02028168066058
C	-4.08913190854152	-3.74332639186161	-3.06116254074417
C	-2.00565579960667	-4.53175792532423	-2.04810186738176
C	-1.09103557134158	-3.50332730691156	-0.02055569809181
C	-3.91466935017978	-4.62237576744624	-4.10983931556829
H	-4.95021681028626	-3.07526633356956	-3.05826680172955
C	-1.86470080619300	-5.44221990347055	-3.13265183244630
C	-1.01985456326580	-4.44097821179674	-1.03086802582519
C	-2.79923435872205	-5.49357966862241	-4.14336832945714
H	-4.64295384595523	-4.64253437399521	-4.92461027886175
H	-0.98697847589091	-6.09419375656166	-3.15460424819106
H	-0.16578747181153	-5.12180406788321	-1.06687805572640
H	-2.67590211262921	-6.19173686729863	-4.97509407058198
O	-3.24926536863245	0.01823569684578	-0.55094028060894
O	-2.30824336072769	-1.73409226305059	1.03703047462371
P	-2.21625146920910	-0.09579466434521	0.75989572086227
O	-0.86495802612663	0.31771271214241	0.21956995805024
O	-2.78603158514038	0.58650747600690	1.97188284538744
C	-5.53265345449273	1.73508067267516	-0.40072365735052
C	-5.09112656333315	2.49366635341020	-1.51454833274091
C	-5.92678649574601	2.39918527210360	0.78741817666369
C	-5.06895007267089	3.89109961515038	-1.42314661971082
C	-5.88815576753731	3.80159259744230	0.82233821698439
C	-5.47204252991893	4.57036763792533	-0.26730722795479
H	-4.74520482492341	4.46719649553340	-2.29439745164204
H	-6.20826328406412	4.31632398319112	1.73323598254354

C	-0.01329644683039	-3.42559289365238	1.02024668036105
C	-0.25712746688211	-3.91330536518684	2.32663566978783
C	1.26858701572817	-2.92120113415434	0.68442437708158
C	0.78890605310203	-3.90637821913080	3.25930834339179
C	2.27970442220074	-2.94397126112486	1.65440159593838
C	2.06648476266551	-3.43592931256802	2.94653865044697
H	0.60388397566073	-4.29107449383831	4.26660068627843
H	3.26944360223722	-2.56524876315867	1.38535446272864
C	-5.50696484436373	6.09242664120334	-0.20439441160946
H	-5.77120011534241	6.36102843831405	0.83375133080333
C	-4.14428573158026	6.73329387196890	-0.51029384071903
H	-3.82670106855668	6.53074420169788	-1.54652865860366
H	-4.19022633214260	7.82829664518659	-0.38806571598817
H	-3.35980787605367	6.34610137361865	0.15902676753047
C	-6.60293807383088	6.66919097297576	-1.11739734705580
H	-6.39974531065436	6.43921808914417	-2.17629892183640
H	-7.58950757448473	6.24819863572487	-0.86909177532951
H	-6.66355494190540	7.76593261625980	-1.01809192829110
C	-6.41579825651093	1.65446201519991	2.03032591964766
H	-6.28003150292676	0.57958752883101	1.85113984368062
C	-5.58891985735352	1.99126071797326	3.28163750151412
H	-5.95716548215454	1.41225792493358	4.14485031550999
H	-4.53458041419447	1.72618002072555	3.12031832479907
H	-5.66249252934236	3.05910896962937	3.55017701603814
C	-7.91592185873964	1.89725432308063	2.27347715225658
H	-8.12083710367602	2.95747351498616	2.49799229065506
H	-8.52111449819624	1.62366729174887	1.39473068343653
H	-8.27207258321873	1.30064348986502	3.12952248221047
C	-4.68285015743934	1.84224480943951	-2.83482772755614

H	-4.74360817655895	0.75302538812842	-2.70745145504271
C	-3.22862923241157	2.16435121024175	-3.21478471799862
H	-3.08500938393960	3.24240554040282	-3.39975363419979
H	-2.54154394893810	1.84758488268060	-2.41606325908866
H	-2.94367649928289	1.63112751112251	-4.13660521231965
C	-5.65556270873558	2.21056972225895	-3.96908034525068
H	-6.69091215539716	1.93542557475287	-3.71283149494340
H	-5.64121567085103	3.29207376272796	-4.18365190067717
H	-5.38449808706202	1.68393445447376	-4.89902673456798
C	1.59279650243849	-2.35961161707183	-0.70032206461047
H	0.64960941786320	-2.28346374532899	-1.25899007398458
C	-1.61139349486177	-4.47764687650025	2.75248857551338
H	-2.29727565789971	-4.39108697501879	1.89859442744456
C	-1.51800463399309	-5.97507889114620	3.09470628139293
H	-1.11972167548782	-6.55579455957976	2.24752674708155
H	-0.85942917527166	-6.15399973019863	3.96062865860526
H	-2.51318980759548	-6.37832744639371	3.34530896221551
C	-2.23282145019653	-3.67389187157754	3.90666865185522
H	-3.23471181205707	-4.06449043601211	4.15049860333624
H	-1.62159986632400	-3.73747826152059	4.82242602978647
H	-2.33788153894168	-2.61383183073276	3.63298861107650
C	3.18210240530000	-3.46690005190843	3.98352001659432
H	2.75257499487791	-3.90759005332411	4.90006102939210
C	3.67529217819770	-2.05614495433381	4.34512058267672
H	4.12818462318202	-1.55787521320768	3.47192493749012
H	2.84507718582128	-1.42421905109864	4.69874272570627
H	4.43851764396452	-2.09743865636102	5.14058232900060
C	4.35109677282146	-4.36576698973571	3.54772407751350
H	5.11995268524857	-4.42292744243055	4.33650905605429

H	4.00737280817266	-5.38912930974699	3.33132870875732
H	4.83435409440386	-3.97857941026904	2.63526006042937
C	2.17173184617000	-0.93731979060890	-0.63100541878379
H	2.33639005762588	-0.54299030526954	-1.64769516314571
H	1.46953605119279	-0.26767246830617	-0.11380278418796
H	3.14375995076617	-0.91517951826974	-0.11062951602102
C	2.52372018591933	-3.29905461953978	-1.48726714613635
H	2.71081624193762	-2.90224280360039	-2.49883392638970
H	3.50033225142757	-3.40928511927975	-0.98706794415127
H	2.09132403360733	-4.30639669364704	-1.59385229396222

TS2S

C	-6.33871329222252	-1.54976634342349	0.34742334890017
O	-5.35088157690960	-1.71549547170289	1.12073261150461
O	-6.58520111160039	-2.25735076942984	-0.67190347133957
H	-5.57702002515562	-3.18056661749931	-0.92091074332367
C	-4.48745469220869	-4.24305483314517	0.35433117453454
O	-4.75726088764877	-3.87701852047478	-0.95837016912469
H	-5.42753865762353	-4.36169824539369	0.92179671812219
C	-3.74736358448528	-3.12316833777458	1.04428190521698
N	-2.81354855493338	-2.61402210090000	1.51628942389119
C	-1.89806789047958	-1.63932994525071	2.09409258085596
C	-2.63153089178418	-1.98478844535405	-2.85711842578008
O	-3.15878591600446	-1.04589167564521	-2.31152399469298
O	-2.92188832656587	-3.25949858505701	-2.68693025419758
H	-3.65087229274720	-3.41429391844905	-1.99447898910225
C	-7.29591274597098	-0.43329378521256	0.67091364643356
C	-8.41272111771799	-0.19934633534692	-0.14396510668280
C	-7.08499404942517	0.38139347168768	1.79227722371134
C	-9.30568307802412	0.83019059303086	0.15176308430838

H	-8.56950325090899	-0.83873713003072	-1.01408497817564
C	-7.96842245735735	1.41497766634233	2.09928073496323
H	-6.21552302513616	0.19187003041237	2.42309395598735
C	-9.07625020096572	1.63183988871857	1.27423678175875
H	-10.17685369195914	1.01697961349993	-0.47812935780832
H	-7.80797266456609	2.05268899494446	2.97001620543735
C	-3.67181476454309	-5.52669858191685	0.48319218137866
C	-3.56463553181800	-6.14004736694306	1.73987595634805
C	-3.03501505882062	-6.10269782530620	-0.62110702068184
C	-2.81741673970139	-7.30796992019148	1.89496955533537
H	-4.07362454926202	-5.70452720718392	2.60531358166284
C	-2.28920865375365	-7.27547858793791	-0.46357814377648
H	-3.12887842150207	-5.64342772348125	-1.60488458136836
C	-2.17477633160694	-7.87780573953119	0.79101993630853
H	-2.74287155275381	-7.77927333379701	2.87790473039304
H	-1.79698381458979	-7.72047069051581	-1.33163163075999
H	-1.59162219104637	-8.79427043073047	0.90905057957800
Cl	-10.18980262171321	2.92475848823915	1.65277572876025
C	-2.72531468439105	-0.65401838564950	2.93420590098361
H	-2.04572267392846	0.09308942354440	3.36967859094003
H	-3.47532116819895	-0.14708093019504	2.31252837851595
H	-3.24579521021274	-1.17349603302244	3.75285883510710
C	-0.88897118638096	-2.40399051325782	2.96231503110199
H	-0.32806516034884	-3.13718348423980	2.36437977796349
H	-0.16939864985290	-1.69536460903772	3.39602260281939
H	-1.39393226348234	-2.93216100197817	3.78575764722201
C	-1.20675106457177	-0.93596045203812	0.91465800793212
H	-1.94294522256373	-0.44157928565987	0.26495810727048
H	-0.51107382260398	-0.17483668812840	1.29426619048589

H	-0.62076919433735	-1.64000232914663	0.30870237348434
C	-1.47480097770789	-1.83844765522704	-3.85220317842454
N	-1.26013649646278	-0.46116538083436	-4.26155195939321
H	-0.55978081911151	-2.21114156350883	-3.36908889075452
C	-1.97881868028989	0.22398636535510	-5.22443429810422
C	-0.36275981262210	0.37031866400772	-3.70946479134262
C	-1.48083330598819	1.49299555868590	-5.25522677969643
H	-2.77423085680705	-0.24435463236992	-5.79666365646594
N	-0.47485131715116	1.56013012330904	-4.30724911177568
H	-1.75258964163088	2.34798325478674	-5.86704303906717
C	0.33202724090586	2.74502948282542	-3.99382091374517
H	-0.28470126568309	3.48285716306627	-3.46250863420388
H	1.17954874293873	2.45036616637653	-3.35402067595009
H	0.70733161765638	3.17635026079345	-4.93154270027861
H	0.32605977403974	0.11100279311205	-2.87105601811476
O	1.23098942202847	-0.27274879250535	-1.40684241822122
P	2.16580215973593	0.85319224903003	-1.01887965951596
O	1.36416193284869	1.65444647786197	0.21727530158437
O	3.47765928660901	0.28900710097539	-0.16227301354770
O	2.68391868400689	1.81515168417840	-2.04589792711734
C	2.03487914592926	2.44735591650493	1.10944791524983
C	3.31855566667409	-0.36312334484908	1.03004092421148
C	2.82020398773922	1.84602732666503	2.09118039536389
C	1.84286426697759	3.86340666446521	1.03239520180589
C	2.92978331516797	0.35801888617664	2.15799387622516
C	3.64093356089377	-1.75787493075847	1.07457097646517
C	3.55707051332870	2.68776003151197	2.99899494361261
C	2.46876671918947	4.64735797690946	1.98125814266350
C	1.00399330729041	4.50900037509796	-0.03353417412139

C	2.65609294397003	-0.36742170691694	3.37125182772966
C	3.48865683531981	-2.41251783925650	2.28077211039070
C	4.15457305403209	-2.48131865123649	-0.13484328982331
C	4.51997642855351	2.18876615544664	3.92346174823656
C	3.34615102789217	4.10487918605156	2.95584674597181
H	2.31744821025585	5.72947825232842	1.96138553348343
C	-0.41099698800237	4.43882945669496	0.02696694073833
C	1.62576177268976	5.25759156630560	-1.06400291601415
C	2.05808404887863	0.23378878677531	4.51663433766631
C	2.96099817823861	-1.76723285395779	3.43035474717274
H	3.75437364938484	-3.47040257570854	2.35131429064094
C	5.43733118253055	-2.17426290943196	-0.65269484452917
C	3.38018407272543	-3.50206440187225	-0.74504447654623
C	5.19734991231754	3.03319737448192	4.77846127040294
H	4.73457769589780	1.12072314649398	3.94506501955978
C	4.04619376012644	4.94766273260035	3.86390317617740
C	-1.17093887406528	5.13541779591620	-0.92123888577197
C	-1.12868847631452	3.69332494988556	1.15045580293295
C	0.81551977398914	5.93808477883652	-1.98683731462363
C	3.14409187418076	5.38104600609821	-1.20035977165960
C	1.80627917607559	-0.49726728126031	5.66005454445146
H	1.78900825808740	1.28979768035985	4.48141843496071
C	2.71291248966596	-2.48714304270204	4.63278427159095
C	5.91091744881251	-2.88990283351470	-1.76027830047262
C	6.35342545641124	-1.12650863094541	-0.02254262244396
C	3.90498171764430	-4.18507317489642	-1.84994998099325
C	1.98247222143696	-3.88984013007950	-0.26043678408367
C	4.95061825921403	4.42725491866421	4.76294896889342
H	5.93639915237558	2.62113133705751	5.47022333861751

H	3.86152982502316	6.02464187979537	3.82197953103530
C	-0.57997069078247	5.90259070270604	-1.93330867113081
H	-2.26031314171187	5.09435923143411	-0.85206941327022
H	-0.41291948745740	2.98551065686041	1.59046909061235
C	-2.32459225658984	2.86478879169701	0.65979136340264
C	-1.55037225905495	4.66590787947124	2.26687463757032
H	1.29385314253449	6.52843556527800	-2.77386700996885
H	3.60140419867967	4.68731374089282	-0.48174951656585
C	3.65027993798855	4.96388683093110	-2.59086930837511
C	3.62252172409454	6.79989256578882	-0.84340764489118
C	2.14889588150730	-1.86979189703216	5.72820404668973
H	1.33975572197942	-0.01152507464752	6.52090811221697
H	2.96867868560141	-3.54988164016713	4.66571764238583
C	5.16731588057809	-3.89805936567097	-2.37803507492516
H	6.90317626713105	-2.65524820031216	-2.15573317687074
H	5.79822873719442	-0.62574484125837	0.78200228894513
C	7.57994176510197	-1.78616546805090	0.63296950222394
C	6.76802355142773	-0.03476443400319	-1.02197255309871
H	3.30007226162836	-4.96748052285545	-2.31613134706538
H	1.71964326176551	-3.22310651443592	0.57230297440552
C	0.91865145721866	-3.66803802901338	-1.34920323649442
C	1.94089642680856	-5.33086974325467	0.27643052930597
H	5.48812488420625	5.08523665035535	5.45020857681171
C	-1.41767551381320	6.71697420955425	-2.91288593311649
H	-3.14540545811411	3.49594122268605	0.28252998014634
H	-2.02963240208085	2.17316470489252	-0.14405410895208
H	-2.73878222499844	2.26567313961781	1.48646335727398
H	-0.68450217218917	5.21520925356238	2.66805791387263
H	-2.27547820531451	5.40839228315330	1.89401861794441

H	-2.02415128700878	4.12210195280880	3.10130729432968
H	4.74974613691859	5.03794153212606	-2.62718689330737
H	3.37534210539784	3.92093116778017	-2.80197722590665
H	3.25532280527270	5.61620360998119	-3.38792236171815
H	3.21475584037868	7.54910782369560	-1.54255298035211
H	3.31489323899905	7.09446557807732	0.17234013603669
H	4.72200105508122	6.86114650884008	-0.89515629390897
H	1.95786616080985	-2.43561897391222	6.64342159961691
C	5.71441645090457	-4.64322879880687	-3.58831709634029
H	7.27876535555140	-2.53390254053076	1.38398064455823
H	8.21159328690679	-2.29798414902084	-0.11183743517104
H	8.20457473951570	-1.02994882970727	1.13680045554927
H	7.36712001465703	0.73962738204335	-0.51513444013442
H	7.38383498110329	-0.44222859176217	-1.84070118287129
H	5.88474022885281	0.45093278454951	-1.46183020805199
H	-0.08436899088203	-3.92240722299712	-0.96585067426799
H	0.92369178310712	-2.61152313785481	-1.65731298397759
H	1.09902985338175	-4.30309436820501	-2.23255894776870
H	0.93715349547898	-5.57673370234488	0.66222075607872
H	2.17928520206883	-6.06341164821849	-0.51226177101376
H	2.66616336398844	-5.47962283506145	1.09232312914377
H	-0.72386572335827	7.10419252771921	-3.67980581252127
C	-2.48227310608324	5.88032350054015	-3.63944815684461
C	-2.05677385307191	7.93302142267468	-2.22002015854004
H	6.73641522562368	-4.26258056714904	-3.75973896984976
C	4.89777120206266	-4.34580817889147	-4.85702478080940
C	5.82490121168766	-6.15633036360933	-3.34106565728192
H	-3.22923370751100	5.47307072417286	-2.93893976359176
H	-3.02426429106615	6.49378698460441	-4.37761691152780

H	-2.03048175362555	5.02989824153527	-4.17538737291042
H	-2.76982604497778	7.61489909365613	-1.44171527593825
H	-1.29309840164421	8.56118655319650	-1.73589408733841
H	-2.60583084164692	8.55738286333279	-2.94441499742426
H	3.86231537423880	-4.71272122233843	-4.75999272926340
H	4.85055113520969	-3.26386718713388	-5.05612631822447
H	5.34486242701326	-4.83779246954322	-5.73691627032092
H	6.28990726546251	-6.66134092092679	-4.20388901843269
H	6.43427736163021	-6.37260209249215	-2.44968297603802
H	4.83317136355291	-6.61262139132867	-3.18641485882410
H	-1.66067638078792	-2.46272694177508	-4.73731655735922

MS2S

C	-5.77921923045082	-0.64489810031197	0.10853668903448
O	-5.29509907264063	-1.77723158775068	0.66090118664793
O	-5.42475467885178	-0.23621566413650	-0.98048929156215
H	-4.47154907067123	-1.30550211050519	-2.27110719698446
C	-4.90879944549580	-3.02418744109171	-1.46185267410069
O	-4.24607752946268	-2.23878997282761	-2.45612813610539
H	-5.98576281394936	-2.79663471071417	-1.50638130801818
C	-4.40486222985737	-2.62589712031098	-0.06331049378346
N	-3.33700995822572	-3.04603660843649	0.42197426992106
C	-2.71417143570473	-2.73613757866528	1.72148555228774
C	-1.26110805047233	-1.22704318051016	-3.65190210076272
O	-1.95984792601355	-0.27737894095427	-3.92798794335579
O	-1.68380654021508	-2.37146827648250	-3.14663953126330
H	-2.66061693203602	-2.33077155412198	-2.92587111888681
C	-6.77240379589259	0.04478357865007	0.96936706099961
C	-7.31235652398827	1.25929452495028	0.51473393537649
C	-7.19012952389271	-0.47973798621553	2.20366726639545

C	-8.25602201068346	1.94182325875777	1.27626048537507
H	-6.98056958474251	1.65861186962402	-0.44523900810351
C	-8.13480038778256	0.19638146488400	2.97213265378146
H	-6.77367056951154	-1.42197068489440	2.56064566692321
C	-8.66343021734190	1.40419443158065	2.50309683017516
H	-8.67967524278855	2.88563343037701	0.93037580679114
H	-8.46575655875501	-0.20397158878228	3.93130014945878
C	-4.74256620566065	-4.49880070140814	-1.76043185499697
C	-5.88246835608657	-5.30964688604756	-1.84172672031725
C	-3.47910345001966	-5.07835445845604	-1.95651385879931
C	-5.77038449138335	-6.67718566075920	-2.10850235997487
H	-6.87260253353625	-4.86687770443966	-1.69985340604207
C	-3.36863744857803	-6.44213121429022	-2.23192536027448
H	-2.57991716479001	-4.46644689306383	-1.88434304586919
C	-4.51102195561484	-7.24598444960239	-2.30540488023351
H	-6.66994123660498	-7.29473420463677	-2.16957947307346
H	-2.38010587597117	-6.88167854082440	-2.38640361068836
H	-4.41811946947592	-8.31407762162374	-2.51802610367526
Cl	-9.84720159830102	2.24976622343946	3.45863302233237
C	-3.60157531726945	-3.28206204031927	2.85638873411905
H	-3.09276867109960	-3.14327020420945	3.82257402613578
H	-4.56932621762732	-2.76206265262250	2.89874364078969
H	-3.78838506952249	-4.35856357070989	2.71973865359742
C	-1.36046239459432	-3.46387192575508	1.73134507556165
H	-0.71767852020752	-3.08848709284247	0.92112071844719
H	-0.83838315554981	-3.30677957222134	2.68752902987089
H	-1.50567861310505	-4.54464828682760	1.58092415010986
C	-2.47766803563019	-1.22324492262320	1.88283751804933
H	-3.42099270801781	-0.66575554776405	1.97050462775221

H	-1.88937342543007	-1.03676677134604	2.79404396932953
H	-1.90638264953447	-0.82491384422358	1.03076912298193
C	0.25063918042421	-1.24967027988633	-3.82447289043292
N	0.72754348629718	-0.12492030463488	-4.62309889843043
H	0.71193362812344	-1.18094864518384	-2.81565547661678
C	0.35840266371768	0.16414645882131	-5.92451369513248
C	1.59296369444566	0.80632954904098	-4.20074268001042
C	1.03030894139063	1.29326061391292	-6.28928398736024
H	-0.36080965206221	-0.44075751641250	-6.46768239850278
N	1.79181971949593	1.67558797428834	-5.19787805458643
H	1.02272419096829	1.85722020302765	-7.21728265288087
C	2.67651536693089	2.83445283202594	-5.11596357540537
H	2.10253771189064	3.75138946737295	-5.30699907290482
H	3.09624701033476	2.88164463806905	-4.10426636327635
H	3.48704940852538	2.73822195892966	-5.85228380063597
H	2.08674166921726	0.88615509653222	-3.20926716639113
O	1.51781783025825	-0.73524235008636	-1.11128350929913
P	2.24035301526290	0.50898402869166	-0.66578059048993
O	1.12051848451165	1.39280851270057	0.20237240468868
O	3.34147756893704	0.20683600521129	0.54406204540064
O	2.93781127969406	1.37930133107368	-1.68447684633954
C	1.45645348766021	2.27677277261892	1.18930547363944
C	2.90959695409539	-0.39857162822324	1.69745989013232
C	1.95363131464670	1.79845713782594	2.39962760046383
C	1.20724130978510	3.66583060864519	0.95093737435032
C	2.14782206256814	0.33240704826479	2.60574132813702
C	3.32007728817371	-1.74835345056086	1.93440279277526
C	2.32661949356473	2.74943291293459	3.41593611738388
C	1.46756383041448	4.55263048661890	1.97620882132645

C	0.70296014639822	4.15854477632179	-0.37354246199549
C	1.57887535086279	-0.36191002495741	3.73102765338019
C	2.85411333561675	-2.36607397468335	3.07721849867266
C	4.26449986601465	-2.45002560291041	1.00262501915978
C	3.00049197701381	2.38511258723305	4.61770062480560
C	2.04469827918245	4.13948414176096	3.20523308484055
H	1.26078052516528	5.61564632895254	1.82732064962942
C	-0.63862444929815	3.92360428350776	-0.76560941811780
C	1.56299503842112	4.89716294325820	-1.22325369443216
C	0.62545554650087	0.23156595182804	4.60781966554992
C	1.94952813937127	-1.72681690726266	3.96652380843136
H	3.16893158544070	-3.38895594119226	3.29705352072488
C	5.62165775668451	-2.04833972403197	0.94485128556739
C	3.81946562563002	-3.53683818941440	0.20705308441489
C	3.33484017846243	3.32879246490365	5.56674488783226
H	3.26720005309345	1.34173723915965	4.78320614145586
C	2.38474759130007	5.08609011612416	4.21190557779910
C	-1.09112099643658	4.43211898219422	-1.98894461574122
C	-1.62588995226889	3.17575033689978	0.12754592823416
C	1.05537841952045	5.38975339008566	-2.43541625738297
C	3.02306682930671	5.18907923278975	-0.87401613598147
C	0.09133065415862	-0.47273872091909	5.66779621460083
H	0.30623053111810	1.25830342097388	4.42665552229380
C	1.39693681840624	-2.41902630633404	5.08037663705336
C	6.49926742278884	-2.74146767630333	0.10033987389026
C	6.17921737067510	-0.90528799431361	1.79192028690075
C	4.74210990572824	-4.19620408229655	-0.61524862930478
C	2.37032931592306	-4.02235099428435	0.21231790424919
C	3.01266932272517	4.69378691599605	5.37335286931425

H	3.85967555216882	3.01916791271329	6.47408410266395
H	2.15134236741207	6.13983507133900	4.03565248416394
C	-0.26406738228689	5.17380307927019	-2.84054003484848
H	-2.12952665269123	4.25157792268069	-2.27896104206092
H	-1.07044894477991	2.79209192879177	0.99423159911331
C	-2.25034834793886	1.96265031990161	-0.57823801279151
C	-2.70648750992759	4.12500539668032	0.67597788479953
H	1.71348797370025	5.97401932389925	-3.08627864607431
H	3.25356742941737	4.67031750603619	0.06612358915530
C	3.99665497635807	4.63155113311827	-1.92521190200906
C	3.25672124783608	6.69057991581231	-0.63388912066061
C	0.48750427471589	-1.80935274225437	5.91758649650001
H	-0.64464139780096	0.00549107875426	6.31921730439527
H	1.70253523712473	-3.45467652372013	5.25342200647550
C	6.08617837014167	-3.81715253574097	-0.68896573611328
H	7.54807805709975	-2.43341692801843	0.05802272727513
H	5.34968769644721	-0.47403643520419	2.36889441674176
C	7.21028216614288	-1.41218154511205	2.81562555465972
C	6.75577004997025	0.22741895525493	0.92652528685125
H	4.39273999334890	-5.03326289454434	-1.22511084042839
H	1.77254279507127	-3.27951360473864	0.75773092937177
C	1.77427194749611	-4.10663979797259	-1.20195963493279
C	2.22802059761656	-5.36723735939919	0.94707030895065
H	3.27581610624318	5.43102344280640	6.13590644998144
C	-0.78618814682765	5.75783408739233	-4.14746332212688
H	-2.86353940005547	2.25438227079262	-1.44598368696917
H	-1.47178054215426	1.26883683039859	-0.92804993207128
H	-2.90801023204464	1.41404488413447	0.11474663187506
H	-2.25893696152765	4.96749054376090	1.22676942754178

H	-3.32301014888697	4.54642820709596	-0.13529800951820
H	-3.38162427361609	3.58946609123093	1.36407641711340
H	5.03877740645348	4.81676100920298	-1.61667322933963
H	3.85875376453506	3.54465439094802	-2.03058313994064
H	3.85814689333608	5.11560786814860	-2.90745006328434
H	3.06687207405687	7.28164221431428	-1.54550420532178
H	2.59615421721962	7.08183891806203	0.15611045871459
H	4.29914391487733	6.87766901877196	-0.32744604174267
H	0.06686436839687	-2.35448051450740	6.76628632336612
C	7.06806657939222	-4.54127182638441	-1.60074165493208
H	6.78168765093669	-2.19195062522977	3.46508478946097
H	8.09792726248659	-1.84082996643931	2.32184264173106
H	7.55498432876209	-0.58562800605745	3.45889575276240
H	7.08664660332554	1.06584144232769	1.56176291117964
H	7.62881161759399	-0.10986126546467	0.34364223146821
H	5.99825864138580	0.60691911517878	0.22541770616186
H	0.70066770753274	-4.35165672569807	-1.14748047343342
H	1.87406636374610	-3.13920759125843	-1.71288942882515
H	2.25993305119647	-4.88719878382047	-1.81096290997537
H	1.17133138315113	-5.68064303347072	0.98387040217635
H	2.79644740890795	-6.16317452261981	0.43725570955926
H	2.59939581267213	-5.30979803838621	1.98220011088966
H	0.07618716198125	6.22791615432036	-4.65369454141812
C	-1.34730662137721	4.68332274176238	-5.09280358888651
C	-1.82444860040661	6.86495192432905	-3.89645358212878
H	8.04839653518080	-4.05152366492054	-1.46569525739616
C	6.68568127077002	-4.39726830175242	-3.08323995795672
C	7.23701519753427	-6.01925235068471	-1.21265516768273
H	-2.23025656333300	4.18516936238662	-4.66136865996100

H	-1.65398233154832	5.12766430729964	-6.05420754639394
H	-0.60196106305492	3.89858639773885	-5.30033701528669
H	-2.71899707631980	6.46237701960487	-3.39349004689523
H	-1.41147218007667	7.65955458055742	-3.25579887783634
H	-2.15089438592459	7.32329698405131	-4.84482242169701
H	5.71456244183971	-4.87497718544174	-3.29405463598849
H	6.60478350044257	-3.33778393334207	-3.37252562025811
H	7.43930686691656	-4.87411843683385	-3.73198403044305
H	7.99808370542234	-6.50675150721358	-1.84449376672082
H	7.54778754555562	-6.12331749569450	-0.16130930010393
H	6.29434133335156	-6.57708589524865	-1.33802738820253
H	0.56507008156772	-2.19751265733494	-4.28518026326276

TS2R

C	-6.47363519754254	-0.35678600692015	-0.29928198806869
O	-5.64449618290408	-0.75148918723407	0.56142447903311
O	-6.61386436155273	-0.87577069983990	-1.45024223519576
H	-5.73058533010145	-1.85004679965854	-1.61852977105716
C	-4.51857119657097	-3.16597211737225	-0.53393206050084
O	-4.97149427897260	-2.65157850802993	-1.73210791746237
C	-3.93822898013195	-2.09791711855499	0.36122644910835
N	-3.08805307697215	-1.62673486615632	0.99813140608170
C	-2.25825227531576	-0.76652799322132	1.82814170703596
C	-2.50942581522881	-1.23336803181561	-3.47338938281488
O	-2.80842766785547	-0.25379190611021	-2.83114471322223
O	-3.12239729901255	-2.39566029702362	-3.47465241855271
H	-3.90039551824881	-2.43104882301522	-2.80243855432552
C	-7.36792148905415	0.80154517339431	0.04904400471731
C	-8.25458681666578	1.32338574910468	-0.90373123475521
C	-7.32431522268506	1.37270997705569	1.32871772596482

C	-9.08134932469654	2.40148797325605	-0.59000018058539
H	-8.28338106548509	0.87202596323651	-1.89656227211879
C	-8.14793699386698	2.44851133393877	1.65728227774845
H	-6.63542291361147	0.95782572601353	2.06610005234570
C	-9.02167973005213	2.95754513666309	0.69146145186593
H	-9.77096979309684	2.81438807660002	-1.32791149530118
H	-8.12037203475102	2.89583368867378	2.65212369205376
Cl	-10.05401798195395	4.30945563115285	1.09214656612440
C	-3.18916629599017	0.13336439758847	2.65634353656592
H	-2.57314215754449	0.78871149147167	3.28990025180789
H	-3.81990622373561	0.75063689230473	2.00266720581221
H	-3.84610117446124	-0.46595136846451	3.30406183785497
C	-1.41092533763019	-1.67593870511037	2.73123964559085
H	-0.76455462369749	-2.33518941148174	2.13411201144035
H	-0.76212652971065	-1.05611429944045	3.36681048134695
H	-2.04673444213810	-2.29349243428923	3.38420716779318
C	-1.37573185348145	0.05336870643145	0.87404079361639
H	-1.99418142651701	0.66840475879483	0.20441078501059
H	-0.73116285872694	0.71835144246509	1.46541318468702
H	-0.71751240504792	-0.57707751683398	0.25938677185587
C	-1.28669268443419	-1.27499249111237	-4.40040752296122
N	-0.71809923256284	0.04361088837408	-4.62180921700047
H	-0.52773273106253	-1.92096876840948	-3.93557601586546
C	-1.07714874033442	0.92417882922237	-5.62568340839316
C	0.17220992577130	0.63935223969313	-3.81340489302745
C	-0.36414666791796	2.06768133198732	-5.41736076447051
H	-1.79728465634785	0.66174450154166	-6.39496770946449
N	0.40863164435332	1.86662061600315	-4.28793280357960
H	-0.33498131792928	2.99854696220775	-5.97569877665036

C	1.32493581826022	2.84406054081049	-3.68906987481850
H	0.76189397279831	3.55646277362816	-3.06997612969125
H	2.06075383956691	2.31074688167468	-3.06646074625176
H	1.84125134180690	3.38122840314461	-4.49483839236554
H	0.60949030502082	0.19234234437021	-2.89288777030022
O	1.18707726361157	-0.45814475126574	-1.30774005259959
P	2.43086605252670	0.29598139367725	-0.88602373776946
O	1.92544467504281	1.28331505511296	0.37193456333486
O	3.48553975081135	-0.69164744655891	-0.05712074154222
O	3.23900763472477	1.07092509934330	-1.88378761742786
C	2.81542844417297	1.79906259442099	1.27668435870715
C	3.13057998329287	-1.27836462141605	1.12675095640735
C	3.37493658025543	0.95137412305902	2.23107868037823
C	3.08312687148148	3.20490697905641	1.24371562730363
C	2.99617181150853	-0.49224839282535	2.26926012235770
C	2.99036650603273	-2.70280722029437	1.14781077514753
C	4.35531501646471	1.48285785865786	3.14238792848854
C	3.94318622628526	3.71586541033807	2.19644559077972
C	2.47726779341651	4.12965832363785	0.22584101646102
C	2.50295268796678	-1.11150281002061	3.47174966593429
C	2.62590271340400	-3.29506932870747	2.34067081516340
C	3.26606581809436	-3.53077993622283	-0.07239607404069
C	5.11689630937524	0.67192696198447	4.03320002901703
C	4.61511373122447	2.89205247537718	3.13579929569283
H	4.14683074327511	4.78957206513053	2.20671091283521
C	1.11696183845197	4.52175215988687	0.33202190562145
C	3.28952700443434	4.68482878384276	-0.79325106287573
C	2.13883857590724	-0.36966178198310	4.63282651241662
C	2.33028969671737	-2.53439806248128	3.50299579764250

H	2.53692016404112	-4.38329659758592	2.39074863667975
C	4.59316591551713	-3.67020239214953	-0.54705887917638
C	2.21055392295759	-4.20769247961872	-0.73447149888232
C	6.04490719600129	1.22600659004287	4.89019720691753
H	4.97234189265543	-0.40812954165307	4.02636229104998
C	5.56452954978904	3.43609175212911	4.04573656338185
C	0.60840909777940	5.46898789125186	-0.56501827791200
C	0.22555585633075	3.99673974873633	1.45708990985225
C	2.72792299093458	5.63417618626310	-1.66297572315572
C	4.76476038355585	4.32193884409229	-0.97090934989015
C	1.65849216470779	-0.99916666700738	5.76323721698924
H	2.23429792750814	0.71635311680401	4.61958758704049
C	1.85399995249930	-3.15559304568791	4.69168470301198
C	4.83093390989719	-4.47854824047014	-1.66674968836169
C	5.78630383822114	-3.00836759714465	0.14053952719054
C	2.50625692808755	-5.00748941542776	-1.84573991447998
C	0.75332801292720	-4.09931449504528	-0.28582793992740
C	6.26453623330181	2.62466007180834	4.91076776153491
H	6.62059051234488	0.57709612216492	5.55504212818274
H	5.73977578546573	4.51535989823864	4.03250236597373
C	1.39907422328689	6.05045191244051	-1.56529439085600
H	-0.43308824512463	5.78094414333558	-0.46534422635410
H	0.60201414139237	3.00200642945086	1.73495064631648
C	-1.24323611040703	3.81829440643454	1.04837203045783
C	0.33210300805290	4.89583640880112	2.70330211504395
H	3.35897764495329	6.07498834159113	-2.44000366562748
H	4.98792394462323	3.48898089133738	-0.29043947514250
C	5.08804615308348	3.82532461392793	-2.38934807292616
C	5.67692974360810	5.49942817095463	-0.57958126528107

C	1.52490159044984	-2.40855405102077	5.80195272011242
H	1.37974500325220	-0.40422718368777	6.63671548607280
H	1.74289067701771	-4.24345553178650	4.70228975806487
C	3.80841873744043	-5.15756094973880	-2.33371681596333
H	5.85602771206449	-4.58958906958281	-2.03159788161262
H	5.40411534351733	-2.39143466023296	0.96517072668594
C	6.71742705108281	-4.05925632397519	0.77126626241506
C	6.55332545646567	-2.06693480454285	-0.80183001673732
H	1.68712561049056	-5.52849620061367	-2.34880379666976
H	0.71089454513671	-3.35627700063762	0.52270537198099
C	-0.15896474490864	-3.57390712094142	-1.40505445104254
C	0.23166269801192	-5.43044550109423	0.28193421247518
H	6.99726301017766	3.05279961769991	5.59919076299875
C	0.84976650611277	7.13106383656273	-2.48945406606777
H	-1.74924355918478	4.78312593030646	0.88188125334027
H	-1.33935186974584	3.22061321516065	0.12930583648678
H	-1.79954083467661	3.30233958285359	1.84732863191190
H	1.37150630207762	4.97756604241840	3.05506294779638
H	-0.03090267142965	5.91363500118085	2.48361474523828
H	-0.27487475657889	4.49140382281674	3.53060828643981
H	6.15431027055394	3.55350940271013	-2.45717470776345
H	4.49883602600761	2.92862968555417	-2.62743172520774
H	4.90129338323632	4.60035065482662	-3.15180161081167
H	5.52052121169096	6.36658723787290	-1.24286473133232
H	5.49197932130466	5.83577254144305	0.45264029583905
H	6.73773998654708	5.20978977967507	-0.65574321165581
H	1.15484377018999	-2.89719656812867	6.70665137448996
C	4.11209202483877	-6.03532675351061	-3.54097830891541
H	6.17195616069563	-4.70613250797077	1.47690496711754

H	7.17238810404746	-4.70945097907005	0.00597390042087
H	7.53797425365763	-3.57085413423468	1.32248531211969
H	7.36092341615898	-1.55221466824989	-0.25551915315447
H	7.01903932339347	-2.61590212068821	-1.63692927557869
H	5.88410943524265	-1.30157610184646	-1.22142438233745
H	-1.19120215772423	-3.45541624498823	-1.03277306348403
H	0.19721892377516	-2.58915457291182	-1.74195119313762
H	-0.20221545207414	-4.26316509608339	-2.26426517383737
H	-0.80404946313340	-5.32179265102224	0.64751742090626
H	0.23146904019301	-6.22333293718971	-0.48404086990207
H	0.85052071060369	-5.78350396400007	1.12179511252769
H	1.64181724953194	7.35033590551575	-3.22683051907918
C	-0.39198136220906	6.67543134351448	-3.27198900422398
C	0.56678273806112	8.43493425710735	-1.72341778413797
H	5.19343616233176	-5.93724742048290	-3.74056375694328
C	3.37049554775340	-5.56600211991764	-4.80309144121681
C	3.83176229412767	-7.51973657274085	-3.25349320737341
H	-1.23517145142351	6.44877835792913	-2.59935269164820
H	-0.72697139600751	7.46317603184572	-3.96643130588235
H	-0.18493835784385	5.76960717426920	-3.86423830132961
H	-0.22632054333965	8.29203920441920	-0.97105207425245
H	1.46583907546152	8.78976797582908	-1.19629484306285
H	0.23638431898651	9.23141401214321	-2.41064554606442
H	2.27832307791197	-5.66421464855568	-4.68736516736055
H	3.59051398049009	-4.51036316076348	-5.02655981542771
H	3.66537070996257	-6.16996543405600	-5.67712995947361
H	4.10675284846689	-8.14655276919695	-4.11797765587784
H	4.40258927190949	-7.87223597267753	-2.38023111404674
H	2.76280848607313	-7.69089525272859	-3.04420720349778

H	-1.55843654955810	-1.72353783916987	-5.36542070733019
C	-5.50729819906974	-4.03115077929158	0.24577458297385
C	-6.88389898029653	-3.94802756999840	0.01304307824915
C	-5.02025768024732	-4.93472789310443	1.20074115008654
C	-7.76262559614096	-4.75718595024396	0.73845521911627
H	-7.26707468894294	-3.25540695652723	-0.73708924901634
C	-5.90014084337946	-5.73828985344566	1.92771694760691
H	-3.94267139598277	-5.01868121596145	1.37486611669808
C	-7.27606565263205	-5.64947139624178	1.69710942455355
H	-8.83681854254136	-4.68768612125563	0.55079473432961
H	-5.50996973237930	-6.44127025744843	2.66766921706568
H	-7.96782098283170	-6.28006448987283	2.26111010184826
H	-3.62552931066964	-3.78893032269745	-0.73875900125631

MS2R

C	-6.74130057182166	-0.78617988841023	0.34862121115344
O	-5.63800933696413	-1.28850983656274	0.92764963678167
O	-6.86856650443085	-0.67544957117929	-0.85865094196484
H	-5.86615436843676	-1.65137321569206	-1.95287886748644
C	-4.89588456194111	-2.92626833871697	-0.82341008536789
O	-5.22808428948000	-2.38443773167958	-2.09916670062266
C	-4.55005706587025	-1.80220074839829	0.16378776444294
N	-3.36879547642501	-1.42919989271939	0.32026321271910
C	-2.77419368331422	-0.37040174078407	1.14637253082889
C	-2.56087306327865	-0.97544925261514	-3.76100160136800
O	-3.08086829830499	0.08446545340191	-3.50537581514302
O	-3.08703516016071	-2.17130502957884	-3.58364279435048
H	-3.95251767207586	-2.13664925505821	-3.06869013856520
C	-7.77555357358268	-0.36939592072997	1.32536477575008
C	-8.95139920978605	0.22204597847504	0.83522658337197

C	-7.61979629933742	-0.55958391528472	2.70810371579195
C	-9.95823559872336	0.62052369351444	1.70877208410582
H	-9.06273819372378	0.36409236264103	-0.24097301952724
C	-8.62377077927669	-0.16627180037481	3.58968211318043
H	-6.71029471142242	-1.02268090847897	3.09125772706534
C	-9.78873538770079	0.42151717149540	3.08408554511828
H	-10.87408136020247	1.08158737819850	1.33669395221424
H	-8.51309231387277	-0.31238086459877	4.66495284620646
Cl	-11.04734402935706	0.91265287454115	4.18100279520819
C	-3.74331527352635	0.71877963247538	1.64316898875321
H	-3.16142221966085	1.51901141092291	2.12488772783229
H	-4.29599135838826	1.17221888601873	0.80578046993896
H	-4.46682609165495	0.33934161406380	2.37637329637883
C	-2.11456144120450	-1.08392613567029	2.34204673319318
H	-1.40501561921353	-1.84946490787311	1.99539179529148
H	-1.55940510598867	-0.35650331979791	2.95343210157094
H	-2.86945096484037	-1.56872944998097	2.98190495049852
C	-1.68961967522063	0.27854079721659	0.26718715862769
H	-2.14478417410890	0.76009479692028	-0.61308108084892
H	-1.14479659946194	1.04657671537574	0.83512382651274
H	-0.95441082807041	-0.45667653716899	-0.08643538035871
C	-1.15351341952551	-1.11081836944470	-4.34810635442973
N	-0.53086132359717	0.17263947247792	-4.62183888169064
H	-0.53010141320988	-1.65931451063955	-3.62809097710127
C	-0.82554188944954	1.01696661259268	-5.67621836883109
C	0.40976296152398	0.74227104471770	-3.85122718487669
C	-0.02660525140722	2.11291795248519	-5.53417237027422
H	-1.57255434941600	0.77021836572946	-6.42442307161007
N	0.73499567457410	1.91901245976554	-4.39510599021544

H	0.06753413610405	3.00709244714083	-6.14301650134803
C	1.73111867862406	2.85173421390149	-3.85458273028923
H	1.23145564055607	3.64984199316463	-3.28793804238528
H	2.41657826637510	2.29894361447417	-3.19192751769392
H	2.29445536937208	3.28707381419561	-4.68996340760747
H	0.82181221122850	0.31047325318028	-2.90775534050200
O	1.35082694409357	-0.30907996271270	-1.33271073901105
P	2.62819547706706	0.38374881677058	-0.90834387611251
O	2.15460923711210	1.46080484971082	0.28689087254450
O	3.59994633883887	-0.62222428488545	-0.00402801183009
O	3.50361968353611	1.05832822954147	-1.92376760007050
C	3.04624449241858	1.95455352136736	1.20054611891978
C	3.17101741703198	-1.13733784352806	1.19050916262475
C	3.52333356810729	1.11656971738036	2.20668655842993
C	3.40059085354594	3.33883811407463	1.12083412530756
C	3.05130804313972	-0.29664138784702	2.29442209488616
C	2.94092317202560	-2.54803109811882	1.26477837787375
C	4.51077215263022	1.62582246297869	3.12304102318094
C	4.26895812815542	3.83668213990392	2.07258161326270
C	2.86517076117615	4.25377088574990	0.05684303240850
C	2.48342166357615	-0.83095498486010	3.50437126116340
C	2.49878593906279	-3.06383979728151	2.46656210121474
C	3.20801831157114	-3.44040906697857	0.08900226101084
C	5.19175392041739	0.80896699037668	4.07200093884073
C	4.86392076606044	3.01340066294939	3.06290396003362
H	4.53832447569386	4.89558795852126	2.04501464455109
C	1.52217397611022	4.71006697854525	0.11644790468003
C	3.72490755531440	4.73260934713868	-0.96163239779105
C	2.13776512678065	-0.01984184962511	4.62362236138991

C	2.21693380786487	-2.23723170022037	3.58650336574954
H	2.33800601430277	-4.14110368890958	2.55795676132718
C	4.54071335949387	-3.68548661526805	-0.32250751308455
C	2.13598171696198	-4.06647653399197	-0.59557909945509
C	6.13141124505243	1.33987923633926	4.93094897644220
H	4.97498463502013	-0.25829244290717	4.10878003283280
C	5.82391621588676	3.53509792007620	3.97476275718662
C	1.07825593661769	5.64578767056181	-0.82537974257294
C	0.58350391326033	4.26790687794502	1.23880384322437
C	3.22784895537582	5.67483419420987	-1.87742740719167
C	5.18224116390726	4.28804529896451	-1.09478058909946
C	1.58683329927097	-0.56960800836425	5.76292205419242
H	2.30277587618586	1.05652196067355	4.56950965747056
C	1.66692690298904	-2.77619711177115	4.78322866458370
C	4.76832702148819	-4.53704247931447	-1.41169458390530
C	5.74659550279060	-3.09328411990326	0.40450328511694
C	2.42169516582105	-4.91524367290065	-1.67296248071755
C	0.67426230248779	-3.85242254074685	-0.20434653209732
C	6.44457655747265	2.72032101492269	4.89546860845434
H	6.64331698830403	0.68603793576244	5.64161398904027
H	6.07145181308321	4.59876833477273	3.91838309543150
C	1.91752378542028	6.15355323852142	-1.82638781436875
H	0.05028369200553	6.00834114616755	-0.76219855505609
H	0.90838369625294	3.27088109035644	1.56690996821366
C	-0.88105243622249	4.13063439845381	0.80054598831764
C	0.69903862429923	5.21290895240577	2.44972066834929
H	3.89498856648865	6.05866719393166	-2.65488497804831
H	5.35185310459219	3.47911887666213	-0.37124057795730
C	5.49722948618593	3.70404137603505	-2.48177033090893

C	6.14929067070918	5.43404190931671	-0.74497034076026
C	1.35846004572216	-1.96422661004051	5.85284404425046
H	1.32339138342315	0.07813280199953	6.60301893779628
H	1.48295487935841	-3.85307848618891	4.83283736733910
C	3.72882627772166	-5.16137713098424	-2.10525958805758
H	5.79733648254920	-4.72561273458790	-1.73106603619965
H	5.37303902895546	-2.42724431863089	1.19442173381092
C	6.56600585554932	-4.19217293449532	1.10504177771599
C	6.62595608371353	-2.23706129737240	-0.52119756716179
H	1.58979644504630	-5.39453049062730	-2.19607364171357
H	0.64629980332720	-3.06250493034972	0.55886031491232
C	-0.17486689511838	-3.34508078806339	-1.37964515000908
C	0.06409295793036	-5.12011225480211	0.41831701755677
H	7.18609294817829	3.13062763497615	5.58542945613197
C	1.43704949366108	7.22163201819447	-2.80180801698719
H	-1.34139068007791	5.10541534499253	0.56997705463992
H	-0.97990874929489	3.48738101811359	-0.08701893049657
H	-1.47747969376144	3.68168471884909	1.61117654434726
H	1.73394154216817	5.26995246912999	2.81937775428792
H	0.37939450890934	6.23412328628363	2.18266549308067
H	0.06197430849747	4.86392172695799	3.27953425385164
H	6.54798630442261	3.37223961325413	-2.51936936350325
H	4.86304989359022	2.82908442329279	-2.68255813454531
H	5.36189629981077	4.44836579892804	-3.28478161821908
H	6.04671417418934	6.27634738174904	-1.44953259771648
H	5.96905622851524	5.82675786553185	0.26806210111920
H	7.19444206248325	5.08669934571267	-0.79140399664094
H	0.93057969731875	-2.38983169250090	6.76399416031268
C	4.01883073830222	-6.07345604001103	-3.29018833279737

H	5.94293808676311	-4.77405589332849	1.80298701801930
H	7.00092505467381	-4.89870455995892	0.37885887578963
H	7.39815034442110	-3.75108946706700	1.67843002166552
H	7.44358491813595	-1.76936915078915	0.05194328306216
H	7.08717136089736	-2.84145544399475	-1.31976441697423
H	6.03568064968195	-1.43636462155213	-0.99019966315226
H	-1.19698852070121	-3.10855145509853	-1.04067967070783
H	0.27289242210263	-2.42140646309115	-1.77542764431046
H	-0.25445610499157	-4.09018418689861	-2.18863383388839
H	-0.97628997454480	-4.93721560038430	0.73580725889128
H	0.05211876095304	-5.95528486379379	-0.30168397328715
H	0.63433894333344	-5.45336290382128	1.29976518166797
H	2.25437690576332	7.38206631344568	-3.52680535918462
C	0.19619354598347	6.78895395809434	-3.59882743520385
C	1.18999787883059	8.56291434009849	-2.08986876453021
H	5.11549731230968	-6.09046766925044	-3.41673513183217
C	3.41793699985148	-5.52773558744001	-4.59622269040288
C	3.56500951431010	-7.51945691028699	-3.03269452712751
H	-0.67046959892461	6.61859832701654	-2.93946445452640
H	-0.09093304479506	7.56492568670665	-4.32731210764762
H	0.37999451673097	5.85586501783903	-4.15551075275291
H	0.37424452131654	8.47885755906518	-1.35294329464410
H	2.09019023684935	8.89978339575059	-1.55298415554279
H	0.90901243981937	9.34696168342385	-2.81258236456673
H	2.31647436783332	-5.50370364179406	-4.54985676761884
H	3.76670622824873	-4.50275026638575	-4.79718331881966
H	3.70064982737700	-6.16138498018081	-5.45333602974571
H	3.83757244873732	-8.17323410026373	-3.87788280202236
H	4.02945884877264	-7.92739506907917	-2.12129046685965

H	2.47142681271501	-7.58024424333961	-2.90545366137163
H	-1.19513004971268	-1.70968487556801	-5.26997202748476
C	-5.93426667905476	-3.90939851636309	-0.29715386615376
C	-7.10353491476971	-4.20152697554792	-1.00704551252611
C	-5.69401326364342	-4.56622195769359	0.92018339150262
C	-8.02298172557026	-5.12855030901086	-0.50393642381740
H	-7.28922994981153	-3.70979616193165	-1.96285117545257
C	-6.61009592752882	-5.49142541497417	1.42094011669262
H	-4.77824502608653	-4.35459719525801	1.48039322363825
C	-7.78080999356408	-5.77445051177184	0.70914595985589
H	-8.93190713084856	-5.34804665024240	-1.06966397044275
H	-6.40829208966923	-5.99692805507850	2.36862448946320
H	-8.49874346202687	-6.50010676117057	1.09912589284860
H	-3.94636693301651	-3.46074453694330	-0.96804082390021

TS3S

C	-6.18399066336825	0.31073094320361	-0.80094920512738
O	-5.79371970130370	1.23547073244178	0.15264013250028
O	-5.70928086552888	0.47276987554297	-1.97407074498878
H	-4.42936194831296	-1.27676736534156	-0.67947187157055
C	-5.06898856561197	-0.73765901897024	1.30482440362112
O	-5.30046818613532	-1.07560068443895	-0.07061786381712
H	-5.85717417196400	-1.23904101650096	1.88877120912029
C	-5.33222458124291	0.77023154047034	1.37970484020345
N	-5.16215251404496	1.46364774505558	2.40345768510369
C	-5.43243080517039	2.90336682511036	2.56510462919295
C	-2.83180529223405	-0.61344305230632	-2.19917303240197
O	-3.35360673008628	-1.53537769386595	-1.53342975030661
O	-3.33253876919730	0.53671765400376	-2.39061065680751
H	-4.58691645727089	0.59704555668900	-2.09616764907184

C	-7.60848593223287	-0.15355511482145	-0.71821683535112
C	-8.42553803513784	0.14585464407013	0.37973194892162
C	-8.13010785014443	-0.91343197965961	-1.77571952694764
C	-9.74368039902412	-0.31007015904225	0.42710203881317
H	-8.04877410585759	0.75522232310750	1.20123365545784
C	-9.44321173421753	-1.37360302058217	-1.73787950911022
H	-7.49475493551317	-1.13935914271926	-2.63291628001607
C	-10.24569586152437	-1.06940424020267	-0.63243928614291
H	-10.38461732571077	-0.07341388325890	1.27739079091698
H	-9.85208576837467	-1.96516965950300	-2.55816881605639
C	-3.72073406880527	-1.18271361285439	1.82344180407016
C	-2.53531639032977	-0.54039886607530	1.43615789261832
C	-3.65164273972750	-2.28115829956313	2.68936019399438
C	-1.29887233272596	-0.99932813528208	1.89040377194877
H	-2.57052548963389	0.32566951009462	0.77023020181630
C	-2.41632221822349	-2.72957204374652	3.16493380841682
H	-4.57127477232412	-2.78588380460383	2.99803838517038
C	-1.24036928441258	-2.09197075067910	2.76299157180909
H	-0.38204342727198	-0.51931330891764	1.54353417439589
H	-2.37411893954611	-3.58112652999205	3.84853147881641
H	-0.27207280346070	-2.44486968650791	3.12650224333481
C	-4.59967279358020	3.72760744114305	1.56612521542646
H	-4.74540430873644	4.80252462424625	1.75576200378240
H	-3.52860278299119	3.50476164338666	1.68151320836350
H	-4.88988065026551	3.51987395436025	0.52661229454168
C	-5.01336082402470	3.26026879769539	4.00023367290108
H	-3.94790954085989	3.03337315812352	4.15494635399817
H	-5.17783443521151	4.32996202648211	4.20359362060806
H	-5.59149926897504	2.67135705600208	4.72859693261847

C	-6.93375381216283	3.19276524874534	2.38084472447736
H	-7.25801932316953	3.00727375923821	1.34676453659002
H	-7.53624863853424	2.57116080371957	3.06256167000722
H	-7.14471113566940	4.24765371008770	2.61517151608866
Cl	-11.89195533241741	-1.63863816973987	-0.58133185281891
C	-1.47275408804525	-0.92602754519036	-2.80998520073342
N	-1.02694431431306	0.05354308991567	-3.80170247179204
H	-0.71411677960594	-0.95762523394143	-2.00002564297805
H	-1.51540440797561	-1.92560918559533	-3.26678861746126
C	-1.73161590455386	0.47658810685053	-4.91486510542698
C	0.15952180855866	0.67519864192461	-3.77984507229991
C	-0.94246783028539	1.37417737951794	-5.57125506941200
H	-2.73585079697338	0.12630181501011	-5.12958883227428
N	0.23106099379935	1.48229123111339	-4.84424362447580
H	-1.11830375210986	1.94684891075772	-6.47705168627843
C	1.37873700000165	2.32942903853681	-5.15872335652241
H	2.14040011973214	2.18691345157996	-4.38270020700880
H	1.79099995790439	2.04940997955417	-6.13837022684262
H	1.06927890319269	3.38388314609034	-5.17151941770647
C	0.84331973038288	-4.45350943137380	0.21439408072855
C	2.16834748905795	-4.57905695639339	-0.53657611610923
H	0.93513192959478	-3.61364645005545	0.91668418517034
C	-0.32444125620203	-4.10718413101022	-0.72220406575396
C	0.53017225848287	-5.71614137906504	1.03636860508399
C	3.32078473989398	-3.83189729496136	-0.18299664997214
C	2.24012427762063	-5.46395900203954	-1.62047850994883
H	-1.24081757151667	-3.92124279044925	-0.13974102908628
H	-0.09807798488343	-3.19341273223060	-1.28844550018309
H	-0.54148516556154	-4.92182158294305	-1.43315420879337

H	-0.40850311580251	-5.58725791878118	1.60046128005234
H	0.40993927439473	-6.59886873094459	0.38621232209359
H	1.33061967875685	-5.94511111382263	1.75769665954147
C	3.31348291331342	-2.94597467442435	1.02836890650267
C	4.50915813273439	-3.97494849530293	-0.94152330702648
C	3.40452389848682	-5.62881079139396	-2.37711823788262
H	1.34993431488126	-6.04183859842102	-1.88235251219007
C	3.42273637670165	-1.52350481531276	0.92169493365991
C	3.26907918538861	-3.49119525537725	2.29616649732320
C	4.52077932041713	-4.86833444554828	-2.02091338296039
C	5.79561201354921	-3.21858438105587	-0.61277000084291
C	3.46730681378993	-6.60737632465184	-3.54252813919286
C	3.59843705373817	-0.69738129084998	2.03003671305589
O	3.42626857491215	-0.97604621191734	-0.33712207737800
C	3.27207781171803	-2.68746075329527	3.46697246645365
H	3.21186270371396	-4.57675283418295	2.40667796138252
H	5.43939987420584	-4.98295916571896	-2.60364841565493
H	5.59586700418216	-2.56608974589397	0.24775498308682
C	6.92255191209457	-4.17531853363112	-0.18554163104417
C	6.24050733623165	-2.30804049095904	-1.76918240141923
H	4.47013813543021	-6.50175993567708	-3.99233318517186
C	2.43917304416412	-6.27773494136642	-4.63683090779678
C	3.32908997014828	-8.06473098165132	-3.07117398703391
C	3.97812979137005	0.73358614900063	1.84060528034383
C	3.43292772303669	-1.26864622335445	3.34158676299624
P	2.14503522166051	-0.04087878541208	-0.84116757295890
C	3.11972659450372	-3.26270952711267	4.75992525972355
H	6.61783181294947	-4.79836291598262	0.67039821384445
H	7.21348069578472	-4.85375902124212	-1.00431828399107

H	7.82055442064888	-3.60762063637905	0.10974380926601
H	7.12891761055335	-1.72349684574539	-1.47759335109951
H	6.50884744739963	-2.89099201229923	-2.66594767601291
H	5.44195438673703	-1.60247450214094	-2.04103532681492
H	1.40682741656208	-6.38520157478729	-4.26505691022556
H	2.55790779409677	-5.24369147079035	-4.99657013654427
H	2.55295521568408	-6.95602430971611	-5.49874428276895
H	3.43791838917942	-8.76435631551785	-3.91666908117755
H	4.09435128994968	-8.31505386320950	-2.31988442100504
H	2.34210568652766	-8.24469046634904	-2.61381502242639
C	5.16481674775556	1.27437028002338	2.45565961714195
C	3.21509453390515	1.56351897194196	1.02404521921205
C	3.38416376117101	-0.48360154949608	4.52925140003396
O	2.09950402124632	1.06462891919577	0.40760894167382
O	0.82846661573104	-0.77199317397942	-0.77281944421503
O	2.56978290588153	0.60913624156307	-2.13722909720608
C	3.09263271213829	-2.47409981518495	5.88948575222050
H	3.01322974287509	-4.34828216707332	4.83823562364757
C	6.10924292586093	0.47792228245031	3.16610353701409
C	5.43827838293061	2.67599509260531	2.32773221930208
C	3.49719421816036	2.95639865633279	0.85998056237371
C	3.21448777804898	-1.06875575153069	5.76691828499964
H	3.46874613944283	0.60061474615195	4.45207849584108
H	2.96743586292007	-2.92728920612486	6.87602294190393
C	7.22545196587495	1.03978168860592	3.75034350967834
H	5.95095426474314	-0.59746344240807	3.23959213999293
C	6.58809672687410	3.22875168374849	2.95808779273619
C	4.57314102762808	3.48346612959184	1.54461472172675
C	2.65914417097791	3.82225912349861	-0.03207533274334

H	3.16974337128463	-0.44105775752327	6.66048773600319
C	7.46398421787142	2.43236442166431	3.66198635048549
H	7.93512487102321	0.40133265271251	4.28265134084405
H	6.77017198212496	4.30250294888781	2.85944698748527
H	4.79042596422984	4.55112559459605	1.45673040006445
C	1.35325757652114	4.20208281872187	0.36436591242193
C	3.18434617940945	4.28790636375577	-1.26204500210329
H	8.34706432695265	2.86705410308029	4.13685145905867
C	0.60230923617948	5.03534372997963	-0.47291435610528
C	0.74999533857757	3.75893673617272	1.69575343372777
C	2.38879378796199	5.12195232448780	-2.06240271907889
C	4.58852816578026	3.92828904541644	-1.74834128722204
C	1.09822045657295	5.50897352425291	-1.69327916935208
H	-0.40147536353656	5.33028716608910	-0.15468660528338
H	1.47040722949817	3.09000162950900	2.18621874919211
C	-0.54422431987095	2.95486446387931	1.50002948328713
C	0.53622440105265	4.95154038478115	2.64374940736946
H	2.79523765056268	5.49168980371834	-3.00896309933738
H	5.01726054448429	3.21549507904816	-1.03125882952108
C	4.57452678762559	3.21427831297372	-3.10982501827879
C	5.50864974656553	5.16115100733272	-1.77077894362473
C	0.26972820339607	6.42080429762303	-2.58926135742612
H	-1.33185150120040	3.56495326812075	1.02835434787551
H	-0.36178279595261	2.07744462577031	0.86270763948404
H	-0.93192358708196	2.59805120220847	2.46841188041084
H	1.47646982999329	5.49873438926879	2.81630014611556
H	-0.19686989184680	5.66847460489339	2.23817430578848
H	0.15914016762786	4.60695455763630	3.62089696350033
H	5.59937299206580	2.93072135567276	-3.40130809415693

H	3.97058914871591	2.29612721084539	-3.04948107808941
H	4.17683993038760	3.86343810185263	-3.90909087711630
H	5.15369443790940	5.91816337550767	-2.48994495428910
H	5.56200498928698	5.64440687549917	-0.78235183378614
H	6.53248843418321	4.87678601609492	-2.06472680460939
H	0.89213036896228	6.65005209271389	-3.47257593058137
C	-1.00738989040423	5.72944402797230	-3.09534054244407
C	-0.06156845545161	7.75776678685254	-1.90604801127976
H	-1.69014074170697	5.48546707765805	-2.26509519647204
H	-1.55617927640936	6.37983606967720	-3.79655909815377
H	-0.77492459123380	4.78517622943785	-3.61463635298844
H	-0.69455894251536	7.60593457790940	-1.01650092658927
H	0.85344689049496	8.27587531630110	-1.57970922691076
H	-0.60743573855723	8.42599572622874	-2.59261168425328
H	0.98502679657734	0.55618049257798	-3.04931421713621

MS3S

C	-6.05129086554950	-0.10255393318369	-0.63956252161735
O	-5.99458003389165	1.01399968244494	0.25627935595979
O	-5.69100079528336	0.25046705907219	-1.89405263986092
H	-3.72767345673153	-1.49631068592599	-1.06957564115962
C	-5.01145220459089	-0.81021778626768	1.33381331918084
O	-5.07916432266506	-1.01571248281584	-0.09541587251095
H	-5.80149275257291	-1.41754160625266	1.81071159768916
C	-5.40776663635963	0.66023950946148	1.44896996164185
N	-5.24477429936332	1.37989911182781	2.46116919459879
C	-5.65605343638016	2.78912183139477	2.59057609491861
C	-2.46957572650099	-0.68621041312369	-2.26786016455972
O	-2.94982540954394	-1.73795127881254	-1.64296681510491
O	-2.98198202431569	0.42107097664302	-2.27303074149423

H	-4.74159878809050	0.51491468244086	-1.92151293034853
C	-7.43892991825287	-0.73197124829479	-0.65885786758478
C	-8.49970398876024	-0.22459683930092	0.09657817216635
C	-7.64610600523535	-1.86681327344678	-1.45629467409161
C	-9.75452855698962	-0.83997684722123	0.06241913547183
H	-8.35470298097204	0.66429873383670	0.71043438206416
C	-8.89118419927859	-2.48897466060584	-1.49830354625551
H	-6.82290496711376	-2.26515798579578	-2.05235691757420
C	-9.94233595787316	-1.96939162584196	-0.73441374606421
H	-10.58476835585703	-0.44431289280277	0.64913299124354
H	-9.05569903692326	-3.37212898237551	-2.11735784212103
C	-3.67049055395470	-1.20814786928893	1.88923880951185
C	-2.49726102380355	-0.54807497651161	1.49358406751838
C	-3.58389761972257	-2.26852409180336	2.79952088563380
C	-1.25570394420107	-0.95311887784394	1.98417958670477
H	-2.54988458837419	0.29039712316622	0.79464218604578
C	-2.34385115272717	-2.66219888747216	3.31082067299362
H	-4.49375482698282	-2.78765695119951	3.11314731593265
C	-1.17960769057110	-2.00828102749460	2.90100513712168
H	-0.34797696865496	-0.45587298673275	1.63757027861308
H	-2.28832531298363	-3.48524947676672	4.02760333478775
H	-0.20789083082992	-2.31884526648917	3.29310075972608
C	-4.94149052236799	3.66027699531817	1.54007751685940
H	-5.18508839954454	4.72203187891538	1.70305349217722
H	-3.85003578941442	3.54534188933295	1.62239358576170
H	-5.24560480188635	3.39124450127426	0.51847950064243
C	-5.23058194622787	3.23351063898558	3.99915962382395
H	-4.14338011667630	3.11780778522047	4.12381877618148
H	-5.49482802095598	4.28783919430116	4.17697826196852

H	-5.72384611780928	2.61412123927392	4.76385050768357
C	-7.18379460231639	2.92674596223258	2.44939595112251
H	-7.51567878063198	2.69016260128390	1.42815128237746
H	-7.70063625401980	2.25871286961374	3.15651853259401
H	-7.49281994194473	3.95988084933083	2.67276401714001
Cl	-11.50600920357206	-2.74483906020908	-0.78294884363211
C	-1.17057745924050	-1.00534779233480	-2.97792948407100
N	-0.79256543364628	0.03556628435294	-3.92718413328088
H	-0.37615598213353	-1.07105013618771	-2.19721086655340
H	-1.24481533246871	-1.97838929488109	-3.48464331906107
C	-1.54279966318050	0.47288827242165	-5.00512653097068
C	0.35123214603967	0.73392414647371	-3.88088424856011
C	-0.82445981001437	1.45565228217080	-5.61808556146988
H	-2.52263787304507	0.06443246377619	-5.23230719585593
N	0.35134068611386	1.59985811160726	-4.90030672741910
H	-1.04999312242971	2.06298818423566	-6.48969404925383
C	1.42367925442832	2.55242443347850	-5.17785614382836
H	2.25959843242009	2.35382151129929	-4.49700574410961
H	1.75827880487640	2.43847641915300	-6.21815605304760
H	1.06521327318844	3.57748154234789	-5.00841062551349
C	0.93133575036236	-4.44744315605617	0.29016278176236
C	2.26722615412362	-4.51903115499388	-0.44926405034945
H	0.98418244379275	-3.60949821082784	0.99845504109134
C	-0.24171394291057	-4.14050372292991	-0.65293099814209
C	0.65913623791134	-5.72643159200096	1.10139654881185
C	3.39080126850164	-3.73586437592362	-0.07879125966161
C	2.37986329041901	-5.39361985646017	-1.53795234242359
H	-1.16977536319352	-4.00216575913897	-0.07573346536773
H	-0.05086760956024	-3.21071613142776	-1.20549872896336

H	-0.41822097129042	-4.95493901319697	-1.37527119108956
H	-0.28397676212167	-5.63295561237493	1.66500137718112
H	0.56964066841492	-6.60746391768453	0.44399450864138
H	1.46544282344001	-5.93476622308680	1.82231280993756
C	3.33256796393268	-2.84638331921822	1.12835704767549
C	4.59287277826777	-3.83898088179923	-0.82215097536855
C	3.55741800584702	-5.51608703279917	-2.28223354157479
H	1.51208616391771	-5.99848844133410	-1.81383532522006
C	3.40232228065101	-1.42207878307650	1.01667432239819
C	3.26097072169423	-3.38459719840316	2.39799617081576
C	4.64560273213588	-4.72521200453444	-1.90625134876532
C	5.85308538301167	-3.05009044167648	-0.46926029359881
C	3.66213549689930	-6.48006845795704	-3.45670964783618
C	3.49649356796378	-0.58159574866588	2.12368678389675
O	3.44529859733001	-0.88171422892446	-0.24596388342841
C	3.19212697870810	-2.57396778439905	3.56181338306680
H	3.23392801212888	-4.47055835970847	2.51492033006362
H	5.57489694849716	-4.80942777721706	-2.47712050471330
H	5.61765470437126	-2.39532484527134	0.38050096126607
C	6.98903388741588	-3.98003505169029	-0.00752487148027
C	6.30701360638144	-2.13911463874839	-1.62172989764811
H	4.67276026712785	-6.35352694887191	-3.88305315617272
C	2.65320215769260	-6.15143816479623	-4.56926747142201
C	3.53609526863151	-7.94536044688711	-3.00744472600996
C	3.81953012206214	0.86371284568534	1.93671370118268
C	3.30093773928800	-1.15057399807553	3.43203274644105
P	2.15202403076876	-0.00800137654963	-0.81456676182185
C	3.01204371614994	-3.14699237751911	4.85228987290404
H	6.67707858746397	-4.60180824031361	0.84667644752120

H	7.31068334594048	-4.65919314567312	-0.81403338468019
H	7.86894959750885	-3.39246146264456	0.30282803703099
H	7.17344206510791	-1.53137419211819	-1.31210515439197
H	6.61314451785694	-2.72240483853505	-2.50602458663801
H	5.49998150799015	-1.45459665749332	-1.92081276916579
H	1.61490233805801	-6.27697341186020	-4.22020803434175
H	2.76582173494107	-5.11170809035072	-4.91447867663210
H	2.79431186797810	-6.81784605431493	-5.43637894271809
H	3.67725018208524	-8.63155246843431	-3.85907003245887
H	4.28664739279062	-8.19334226184424	-2.24074087010210
H	2.54123490071121	-8.14789910860896	-2.57747460379708
C	4.95473432700975	1.46159178521992	2.59394346058204
C	3.05533189315002	1.65147472085606	1.08013910557556
C	3.17282843385444	-0.36070551239274	4.61051229855091
O	1.98855908696958	1.09365163175293	0.42470812653523
O	0.87043258603057	-0.80408848277376	-0.81407304027602
O	2.61062883086737	0.66127629632611	-2.09013970898558
C	2.90892156971826	-2.35313060940008	5.97369813041162
H	2.94520564141293	-4.23538996728823	4.93463710434458
C	5.90329308455292	0.71401558400496	3.35049565442621
C	5.17150167545013	2.87249749560070	2.46142338958086
C	3.28672154778712	3.05228640826183	0.90535941639817
C	2.97816396784512	-0.94467390739926	5.84502469517373
H	3.21532952544526	0.72556806811405	4.52833507190145
H	2.76309459077234	-2.80473329256420	6.95807945964442
C	6.96894256485075	1.33002322807015	3.97321999109929
H	5.79004353968364	-0.36673091009553	3.42852583313648
C	6.26923532946010	3.48150794874306	3.13194127750679
C	4.30793288061945	3.63233087734826	1.63063869001277

C	2.47055550066101	3.87575964794223	-0.04631831624825
H	2.87187577882724	-0.31386827528928	6.73110865950421
C	7.14922536440924	2.73105478494629	3.87966360209740
H	7.68385616854674	0.72859743484371	4.54042671645557
H	6.40836579872946	4.56121179943057	3.02875985025329
H	4.48586930935039	4.70654685759405	1.53499595422604
C	1.13239352131965	4.21976153738730	0.26653972328655
C	3.05496930955699	4.34837396532888	-1.24720082167824
H	7.99214652680486	3.20874627058041	4.38516445531849
C	0.41170571179584	5.03060943057694	-0.61856947069022
C	0.46216614288492	3.76796632503103	1.56193303611195
C	2.28805902624844	5.15991234227380	-2.09733065506041
C	4.49250953626884	4.02007695790848	-1.65145609318019
C	0.96864548821164	5.51687949841511	-1.80721999158977
H	-0.61653386550743	5.29999758701449	-0.36243724248066
H	1.15440458749679	3.09168505942062	2.08192128661394
C	-0.82391511811388	2.97156832040164	1.29704993457764
C	0.20657924973143	4.95573212677183	2.50588975514706
H	2.74190022069589	5.53731566907979	-3.01906086844919
H	4.89690461186789	3.32075646180398	-0.90763837818313
C	4.57021983756122	3.29847094780234	-3.00675845056179
C	5.38424818448056	5.27362654756598	-1.63138808131863
C	0.18245390255595	6.42817948137384	-2.74171113614424
H	-1.59666110805840	3.59157519869808	0.81359147094432
H	-0.61865571395120	2.11036838252303	0.64419739698916
H	-1.24621911355054	2.58993930880139	2.24078279442049
H	1.14065415117640	5.49302444474534	2.73412560487262
H	-0.49610387660916	5.68120547994567	2.06347875631431
H	-0.22771043347469	4.60830679561216	3.45785583819064

H	5.61498412983822	3.03221811984923	-3.23713485283347
H	3.98058986420331	2.36944792751007	-2.97392114947024
H	4.20853759999124	3.93624520785819	-3.83203188478719
H	5.05224901625997	6.01861732807564	-2.37367837859115
H	5.37222216458719	5.76295898603288	-0.64467119594460
H	6.42878429379897	5.01110785163277	-1.86692255581070
H	0.82601405581917	6.61449574802758	-3.62017479722793
C	-1.11245190390564	5.77436681132485	-3.25102339260812
C	-0.10918541252456	7.79255297436915	-2.09413739194753
H	-1.81872925452000	5.57925384824799	-2.42789952253505
H	-1.62324839831652	6.42837920567027	-3.97687965438925
H	-0.91166204939851	4.80845699357698	-3.74326627102569
H	-0.75606607497438	7.68152194913901	-1.20855907473378
H	0.82000182913961	8.28526287225555	-1.76864908563060
H	-0.62317896685013	8.46350659733478	-2.80230022646083
H	1.18420198262013	0.63424041950762	-3.14627306794795

TS3R

C	-6.25485554780682	-0.46088232423069	-1.31610517627966
O	-5.99061651086743	0.78294074686431	-0.80989279620722
O	-5.79939532872914	-0.71546807971585	-2.48786929756318
H	-4.28377054971133	-1.71305148752054	-0.73083098007468
C	-4.68739853483864	-0.41917871588568	0.81884410493956
O	-5.12158092630587	-1.35055446170327	-0.15542011124043
H	-3.59383686463912	-0.29251274500968	0.73342201264439
C	-5.30702587094444	0.92469374375009	0.40029450858823
N	-5.16135575354211	1.99563043543605	1.01815924115555
C	-5.67741299980309	3.32675601296217	0.65055913033509
C	-2.74049407710503	-1.33967678474432	-2.37070996545815
O	-3.12580170958327	-2.06011932557186	-1.41670855846160

O	-3.40280960686856	-0.42016099499070	-2.92334123744582
H	-4.76316809399399	-0.46143387673666	-2.66199341732350
C	-7.57773136172137	-1.05604784647345	-0.97400125848967
C	-8.40071641650663	-0.49575133060137	0.01286057104446
C	-7.99730516574801	-2.21212024882803	-1.65028640420947
C	-9.62591828054117	-1.08280026118107	0.32598247929120
H	-8.09626442713340	0.41013158513303	0.53736401213975
C	-9.21802782130730	-2.80581386364291	-1.34304777474989
H	-7.35772843845569	-2.64356906597125	-2.42115393595635
C	-10.02709150942664	-2.23774500923934	-0.35212790822062
H	-10.27182344347156	-0.65022285814118	1.09093666446265
H	-9.54939749368092	-3.70498228157964	-1.86411568948344
C	-5.03579445975196	-0.84265114959548	2.23651708243039
C	-5.95392209185289	-1.86914078292291	2.48554438720664
C	-4.41730699079877	-0.19229875341942	3.31374516953536
C	-6.25737934579963	-2.23449205239912	3.80075579528342
H	-6.41935761240443	-2.39236009225035	1.64940824268925
C	-4.72434959590627	-0.55845292251758	4.62493337335113
H	-3.69597430209186	0.60465156500769	3.12271439525355
C	-5.64662722063522	-1.57992806364811	4.87229565627054
H	-6.97152851599524	-3.04078601500187	3.98575050743194
H	-4.23380860495304	-0.04835865858633	5.45745830376977
H	-5.88143908608084	-1.86983591128063	5.89943724322219
C	-5.04356646986840	3.78298443113844	-0.67733386047136
H	-5.35676243102399	4.81265467671825	-0.90908840145914
H	-3.94497881270269	3.77052212420966	-0.60531402179632
H	-5.34434507460168	3.13659033402917	-1.51415318644201
C	-5.25822676377231	4.28153911236791	1.77928244395443
H	-4.16372550263707	4.28803608301202	1.89231756785966

H	-5.59484036647915	5.30845737035670	1.56892189578462
H	-5.69349139201097	3.96051483669437	2.73789154450097
C	-7.21334245745480	3.30884536550824	0.53815378330645
H	-7.55382052510361	2.69063994679654	-0.30434486261008
H	-7.66535384201101	2.92749321052874	1.46771731458746
H	-7.58771393076793	4.33156261910289	0.37766019833157
Cl	-11.55412443058676	-2.97728684854905	0.03641113494049
C	-1.35102580037140	-1.67515382564032	-2.91375824870630
N	-0.78776562914653	-0.66406299785454	-3.80260529728643
H	-0.65311317089318	-1.81753304492380	-2.07669697039171
H	-1.42614042638247	-2.63756759765628	-3.44539754573953
C	-1.31527793710418	-0.17601884435015	-4.98530299099012
C	0.38362117318352	-0.05599663173941	-3.56263389330008
C	-0.42765107583629	0.74764499900331	-5.45563056110568
H	-2.27586797138028	-0.50134155279947	-5.36933798849411
N	0.62153996350123	0.80700374483945	-4.55315019686892
H	-0.45762658030613	1.36975820204704	-6.34530250543523
C	1.80118644615692	1.67244972469885	-4.64238411467970
H	2.46029626822810	1.44790935133608	-3.78781622330329
H	2.32184355773741	1.47917619130565	-5.59055939944881
H	1.48541422091713	2.72394470475331	-4.59338068186337
C	2.05617060235764	-4.39297519845214	0.18751828409192
C	3.46244958025210	-4.31574036285316	-0.40734370989959
H	1.94517894766949	-3.55449081416053	0.88917358481265
C	0.96411150742281	-4.21645763457301	-0.88044521551150
C	1.84538694100433	-5.69466968442502	0.97991941311674
C	4.45088591983078	-3.42752248124795	0.08725892877620
C	3.78145146534530	-5.15225907898160	-1.48493099669659
H	-0.03058893553466	-4.17908974171114	-0.40510530684752

H	1.12456741218006	-3.27399878355596	-1.42297007890579
H	0.95327262049464	-5.05136459847477	-1.60081693293684
H	0.83944842301775	-5.71809471596296	1.43127989561184
H	1.94060717978908	-6.57915759266160	0.32786360812851
H	2.58193733523722	-5.80257559991097	1.79153435776703
C	4.16574719683995	-2.56232165074475	1.27973492438315
C	5.72698558257406	-3.38690847010502	-0.52468913329494
C	5.03725686491116	-5.13485412207249	-2.10037943276680
H	3.01557929127158	-5.83610314584432	-1.86003529905152
C	4.01102263072941	-1.14627879882609	1.14891537601745
C	4.08375377579555	-3.10466596061412	2.54584523192299
C	5.98941138185352	-4.24174324879232	-1.60364535660956
C	6.84210813821301	-2.46248460484892	-0.03919882331747
C	5.35843156688480	-6.03871570918930	-3.28351739606255
C	3.85156267164329	-0.29921128944949	2.24444285871855
O	4.10500961199051	-0.62061559184038	-0.11277246199781
C	3.79191151977618	-2.31334871524028	3.68817742004669
H	4.22378871000752	-4.18073341046790	2.67521760111613
H	6.97632111766664	-4.21359499575297	-2.07464429939204
H	6.44565219904320	-1.85962391334717	0.78931928784178
C	8.03057905213721	-3.26059623653945	0.52499040022062
C	7.29225351284311	-1.47960274080796	-1.13252239575905
H	6.41714069786820	-5.85998328134254	-3.54101645872442
C	4.52035102794756	-5.67663138106500	-4.52130626792898
C	5.21717064418061	-7.52922362198514	-2.93588453538361
C	3.87180627091400	1.18307566938605	2.06033949573105
C	3.65485730264436	-0.89361069879930	3.54249357983492
P	2.79856280595881	0.05888721805429	-0.88240561923749
C	3.61792889753864	-2.90682317049411	4.96965399057755

H	7.71243745307222	-3.93468520313747	1.33613756570485
H	8.51103626603056	-3.87816466700907	-0.25182674709031
H	8.79812680326915	-2.58019284270634	0.92980419909849
H	8.04889411776028	-0.78223189183720	-0.73625436417159
H	7.74508066838610	-2.00484705576280	-1.98977101851109
H	6.44206909737307	-0.88697771303324	-1.50061373782525
H	3.44593719237294	-5.84351466822051	-4.33776490565887
H	4.65092448989159	-4.61807544404445	-4.79510400368460
H	4.81099867767108	-6.29397533715402	-5.38780606428556
H	5.51932447088451	-8.16087556294661	-3.78778993869851
H	5.84332467594471	-7.79939463665608	-2.07118269987069
H	4.17468115585927	-7.78700135749498	-2.68656762235672
C	4.76683257103277	2.02926791672638	2.80818210390498
C	3.02402351592861	1.77823736832033	1.13041875768326
C	3.29018741081572	-0.13996991224938	4.69541901688960
O	2.20642958586537	0.98990529963158	0.36985728359787
O	1.69705774237031	-0.94326971966665	-1.15469035448662
O	3.33879216201042	0.89923150788841	-2.00522227141220
C	3.28688472631748	-2.14368358259206	6.06777264887644
H	3.73981069758508	-3.98959485382399	5.06238981042930
C	5.79872075907258	1.52245045756307	3.64949272316383
C	4.64559018290229	3.45298496168123	2.68320446433321
C	2.90666002011302	3.19576030596801	0.97721012809937
C	3.10849683836669	-0.74706394210837	5.92079909859274
H	3.14020587778810	0.93544108210293	4.60144180543344
H	3.14893958419683	-2.61243235298839	7.04530611700570
C	6.63062914225063	2.36889758816783	4.35284124009877
H	5.93860842696631	0.44453883364704	3.72774389770824
C	5.50584280142388	4.29907147829079	3.43754530443240

C	3.68940507746173	3.99559239712083	1.78461762654911
C	1.96864858273425	3.78624229763271	-0.03307682306962
H	2.82035220313817	-0.14353804425329	6.78532905407482
C	6.47739222017871	3.77329915874508	4.26037813818342
H	7.41909678362884	1.95027584096808	4.98344744296183
H	5.38897955397333	5.38179129558951	3.33720286772784
H	3.60131127784129	5.08245311699229	1.70897206536619
C	0.56535495599958	3.67410581255648	0.14282908539365
C	2.48033091365534	4.46852279348679	-1.16504720211562
H	7.13750484727369	4.43377305544802	4.82820842309344
C	-0.28970806143872	4.23979603138006	-0.81014795414569
C	-0.05140259679477	2.99452941756196	1.36482421561014
C	1.57621981020836	5.02023435574811	-2.08648428250492
C	3.97693553594325	4.63660100005883	-1.43204831326849
C	0.19146235223010	4.91849714811526	-1.93618919373723
H	-1.36926821217393	4.15620102782965	-0.66028496877613
H	0.76611994138208	2.57480386064507	1.96583772181177
C	-0.96894757068326	1.82106562460609	0.98180639403852
C	-0.78034826317020	4.01176596496156	2.25999809461518
H	1.97003898526874	5.55411160771808	-2.95675585069053
H	4.52090784826967	4.07618303492203	-0.66050740093381
C	4.40340795784803	4.03720455761169	-2.78226511956144
C	4.40582198465153	6.11007056924330	-1.31463474838874
C	-0.75259045923436	5.54133090111011	-2.95706822387400
H	-1.85232132694942	2.16233003584385	0.41629805088431
H	-0.42143719803125	1.08319065780237	0.37745682107606
H	-1.33469102258404	1.31167493560150	1.88932156945088
H	-0.10640680421064	4.82473710207490	2.57292813786282
H	-1.63596183637302	4.47247978772870	1.73862081405431

H -1.16760961954775 3.52293063085895 3.16935815062773

H 5.49165892691620 4.15029369872166 -2.91919781762579

H 4.16408531754113 2.96446273631451 -2.81374090238863

H 3.91350844105680 4.54394297296874 -3.63142862129952

H 3.91705096303574 6.73598726526078 -2.08000524459225

H 4.14793778155547 6.53166759685368 -0.33003602277237

H 5.49534668064255 6.20875347514798 -1.45109030837496

H -0.11932793008791 5.97746749080212 -3.74966695515711

C -1.66524309437758 4.49818712777295 -3.62246246172085

C -1.57928963418970 6.68814291579320 -2.35240623296891

H -2.34915821323476 4.03430119151901 -2.89294000809210

H -2.28411857699850 4.96158006782239 -4.40870759310867

H -1.07919838841736 3.68717027421056 -4.08412892616342

H -2.25017525309364 6.32183046004659 -1.55774932097810

H -0.92868937813405 7.45817168163111 -1.90983308952818

H -2.20585693321305 7.17006448206614 -3.12145946159411

H 1.01832636639996 -0.28668112219435 -2.68123588409751

MS3R

C 5.77911011696488 0.94585956948132 -0.93880087874758

O 6.02855737188230 -0.39960356613667 -0.54611196412257

O 5.43190953414199 1.02994893361711 -2.24624502471100

H 3.27319204235211 1.97489256177720 -0.88714863628000

C 4.37833528965102 0.39124789995072 0.91394960880287

O 4.64675177514863 1.34523390512990 -0.11794707651848

H 3.32440324911747 0.07812201897031 0.82695273504892

C 5.25527477340192 -0.79388728438500 0.51614063883993

N 5.23509096729507 -1.91987041094996 1.06415265812542

C 6.05791541254896 -3.08017871161642 0.67729123891337

C 2.05882078317566 1.35943707762594 -2.23334333984321

O	2.39366565216262	2.21628529393514	-1.29282147123568
O	2.77333268276399	0.45642685336451	-2.63908356622085
H	4.54963850717178	0.61722862683158	-2.39621525962287
C	6.98450975593981	1.83252935031425	-0.66850341763532
C	8.02660250317507	1.41948805762473	0.16786742527184
C	7.02637630730838	3.11468641005828	-1.23293123443963
C	9.09607888168507	2.27334528119035	0.44549713160603
H	8.01550492099543	0.42014000289195	0.60320074340684
C	8.08671706315200	3.97739404956705	-0.96005118443075
H	6.22421712529266	3.43654862267798	-1.89884629152728
C	9.11804664118373	3.55065472514365	-0.11737176400849
H	9.91103688856932	1.95087608202997	1.09499252673969
H	8.12206082962116	4.97592746584376	-1.39809591288923
C	4.62760049637649	0.93581206740982	2.31137678216372
C	5.20278069710952	2.19442834131737	2.51821353790474
C	4.25037632602193	0.15886225645255	3.41737983325625
C	5.40567801926754	2.66888692057259	3.81826398968589
H	5.48471134494911	2.80771848180586	1.66213897297133
C	4.45657712517171	0.63534990394990	4.71252605983782
H	3.80182636675288	-0.82367201295487	3.25788126615658
C	5.03562003774352	1.89206653268457	4.91720129898993
H	5.85429940694693	3.65404103288256	3.96832649056027
H	4.15763220240326	0.02379734823538	5.56736637144888
H	5.19221965070640	2.26574163312165	5.93215220343299
C	5.69770282071056	-3.52855685447309	-0.75175072500236
H	6.24241357964040	-4.45037538800355	-1.00937528164104
H	4.61932997148884	-3.73895243688319	-0.82975861953383
H	5.95646665397459	-2.75901377242281	-1.49314956458104
C	5.71566923615572	-4.20169988598136	1.67093917560723

H	4.63974651598848	-4.43166143833448	1.63648697077403
H	6.27827384082120	-5.11960727338282	1.43940381558935
H	5.95935871553949	-3.89281701141215	2.69899383493712
C	7.55944592842269	-2.74968603677010	0.77635447159782
H	7.85895807143631	-2.00288288925404	0.02759133128458
H	7.80373894150913	-2.36481048017010	1.77916139910095
H	8.15837336337167	-3.65809630830796	0.60703191911143
Cl	10.44926426919170	4.62579495678995	0.22808481080856
C	0.66380684608007	1.62430056571015	-2.75584877356626
N	0.33973125807132	0.78806422335071	-3.90499516626535
H	-0.07230264659087	1.40892808898537	-1.94301730220029
H	0.56180053563008	2.68815561450479	-3.01746932307386
C	0.98985314892060	0.79026733484696	-5.12713795388089
C	-0.63991773558540	-0.12770134847643	-3.93393117712461
C	0.37723138211132	-0.14481325221988	-5.90628100558604
H	1.83605466912460	1.43897959304689	-5.33081026256669
N	-0.63409673150589	-0.70266540051884	-5.14177676586950
H	0.57583247076953	-0.46138690079408	-6.92597399722655
C	-1.56121810982467	-1.74934942657208	-5.56364342155334
H	-2.24927482961616	-1.96360388223146	-4.73752422152597
H	-2.13095893549454	-1.40989315994653	-6.43997592491012
H	-1.00191098724247	-2.66174228123736	-5.81330678003707
C	-2.16305839541880	4.23042476971575	0.89467014670466
C	-3.43153864595997	4.11081183646458	0.04877576446534
H	-2.09778408795068	3.33432261640562	1.52695377670457
C	-0.88518764141093	4.25317815251273	0.04080190671485
C	-2.22194225805225	5.45543470171859	1.82470600570269
C	-4.41296060048002	3.11197428637730	0.27723012555808
C	-3.63333840678122	5.03356507464958	-0.98609556209072

H	0.00569692163590	4.26610156657473	0.68992677893480
H	-0.83352602365228	3.35203369128703	-0.58512115144890
H	-0.83273377261786	5.14619271488765	-0.60422929931675
H	-1.32256956938292	5.50466513306543	2.46079816889809
H	-2.27506763030342	6.39345180982460	1.24690438354842
H	-3.10212018706108	5.42639867791211	2.48613761298990
C	-4.25317977921048	2.13782684234059	1.40773428919414
C	-5.56588009920568	3.06128187437701	-0.54324535704309
C	-4.76419007384808	5.00254393618763	-1.80871638691591
H	-2.87493595686677	5.80276706062546	-1.15416591830301
C	-3.98999708083170	0.75255447902343	1.16770794847032
C	-4.38153574365074	2.54747106466812	2.71933299997656
C	-5.71354609017259	4.00694461317018	-1.56675644450166
C	-6.67956626545882	2.03789860794696	-0.33029146261343
C	-4.95595170433959	6.00356464660725	-2.94052806271055
C	-3.91287303958342	-0.19249024830964	2.18907927540029
O	-3.87971949284851	0.34871858663785	-0.14064658394215
C	-4.18651921411550	1.66070734229400	3.81065734714482
H	-4.61147351764688	3.59445377530124	2.93096189738975
H	-6.60700480674234	3.97145848576456	-2.19709329573931
H	-6.36799221715107	1.36231850631287	0.47787609864156
C	-7.97900622846230	2.71636392146662	0.13768544857491
C	-6.91531136781267	1.16776487115012	-1.57523580019000
H	-5.95063248280331	5.80416065465012	-3.37655756799982
C	-3.91903056090626	5.80122916050147	-4.05864646721948
C	-4.95927911740020	7.45775694207977	-2.44338603991037
C	-3.81689061687223	-1.64698723510155	1.86234447268205
C	-3.92601004875133	0.27503829178625	3.55222090303219
P	-2.41923408171801	-0.13836015230726	-0.75872096246609

C	-4.22320761100365	2.12911696933546	5.15377477032606
H	-7.81629080684452	3.29823136432590	1.05874049917688
H	-8.37424900915526	3.40569373175303	-0.62664478359552
H	-8.75858824498354	1.96409020402301	0.34317238804129
H	-7.66954567072263	0.39154179263436	-1.36432544972057
H	-7.28560213457265	1.76403687868848	-2.42581210835306
H	-5.98614265274658	0.66551355699528	-1.88214917306640
H	-2.89633646632320	5.98886654531134	-3.69118817213093
H	-3.94987834747943	4.77121454618104	-4.44739829228667
H	-4.10463990523463	6.49097010744878	-4.89902174179134
H	-5.17291327111456	8.15443227723529	-3.27094995704476
H	-5.72029804535559	7.61151143804529	-1.66244407689498
H	-3.98274646714903	7.74071855422795	-2.01718042233753
C	-4.74692382045307	-2.60483551959129	2.40629382859127
C	-2.83366439364195	-2.10577607689363	0.98999902563075
C	-3.65413273989935	-0.56903644481027	4.66727052398484
O	-1.96394169463134	-1.21217234312420	0.42511036736274
O	-1.37945091147281	0.95338158603875	-0.73305669663394
O	-2.71966056668120	-0.84197820121973	-2.06317605866243
C	-3.97670854566981	1.27947126693308	6.20956576655194
H	-4.43684305955129	3.18692602720643	5.33033216770057
C	-5.89982282885330	-2.23360903093397	3.15667621760603
C	-4.53322607410324	-4.00132867437202	2.15893268589815
C	-2.63133760324792	-3.49261595048894	0.70182718071182
C	-3.67510678650043	-0.08065767903883	5.95732131587233
H	-3.41155532692851	-1.61737107887112	4.49354653731391
H	-3.99829699977014	1.65314764650320	7.23628170912671
C	-6.76047309584989	-3.18359514411765	3.66647333888239
H	-6.10979863341017	-1.17698096487718	3.32006472705806

C	-5.42844645934652	-4.95770379731276	2.71508655922240
C	-3.45544896990350	-4.40548069405755	1.32775969823615
C	-1.57973222560763	-3.92401138909737	-0.27631786662355
H	-3.45162101422439	-0.74975201106695	6.79213676898376
C	-6.51821530192458	-4.56284306900538	3.45917344269271
H	-7.64070929779800	-2.86682951536868	4.23157837371285
H	-5.24096179227788	-6.01812426367075	2.52475387622478
H	-3.30548822878290	-5.47317871898383	1.14849066924272
C	-0.20364208263658	-3.79273084426423	0.04160410667034
C	-1.96160592364224	-4.45558897340925	-1.53325917540657
H	-7.20277343064782	-5.30662204799613	3.87440599836336
C	0.75472327005567	-4.16963865311445	-0.90643075840980
C	0.26913876715249	-3.29498760896607	1.40664351178132
C	-0.95858005981562	-4.82442810983163	-2.44257307973874
C	-3.42163463451200	-4.65010381503814	-1.94606911544683
C	0.40203810290472	-4.68284626950516	-2.16042972660179
H	1.81253710232513	-4.06258872876265	-0.65205391730931
H	-0.62019866752414	-3.01253826845103	1.98661725478318
C	1.15432389535997	-2.04196417852984	1.30378947371179
C	0.97596501048254	-4.41415737984422	2.19230076364223
H	-1.24997814744611	-5.23917825479545	-3.41258087695584
H	-4.05352369638639	-4.23853396426708	-1.14835000396938
C	-3.77979409606067	-3.87170429680389	-3.22250735833209
C	-3.77432963693314	-6.14142855886974	-2.08321820603591
C	1.45488430914357	-5.07668089797002	-3.18822791536417
H	2.10797468621338	-2.25939940857126	0.79492249738927
H	0.63163068891203	-1.24055337043469	0.76157796163381
H	1.39947166399415	-1.66612401294944	2.31107955625685
H	0.32806316326225	-5.29802663009810	2.30293421206356

H	1.90174544465894	-4.73874378585311	1.68888415526608
H	1.25197950395648	-4.06510543885327	3.20102536745621
H	-4.85295007396087	-3.98441990744122	-3.44903450449806
H	-3.56648099420310	-2.80111130967569	-3.08198912431442
H	-3.22238694533784	-4.24625019615588	-4.09867506246265
H	-3.19053947510689	-6.62368036105010	-2.88497624278740
H	-3.57232398967693	-6.69354660314471	-1.15158047757206
H	-4.84223401141594	-6.26709253186056	-2.32713334229177
H	0.91059797579447	-5.47593047125770	-4.06260380353668
C	2.26868431408166	-3.86342002576716	-3.66989424903509
C	2.37893260488491	-6.19480669204903	-2.67946945157072
H	2.85291200631128	-3.41831364702679	-2.84838520838725
H	2.97781229265793	-4.15352981804844	-4.46293116826829
H	1.61367798353830	-3.07150291367477	-4.06872973133995
H	2.97510939341746	-5.86135560892014	-1.81428833574413
H	1.80050076260652	-7.07772766557177	-2.36619006831273
H	3.08446144920656	-6.50896919995746	-3.46637019399327
H	-1.37433662981350	-0.37313480603051	-3.13067634225591

TS4S

C	7.35608897195647	0.34936145804002	0.00569816064644
O	7.17277722722774	-0.29360337974700	1.37605139827166
O	6.62503278080461	-0.36684914200145	-0.87112408573172
H	5.67519310406059	-0.44770939923398	-0.59198104176833
C	5.98831406874152	1.68538907187041	1.34308339429364
O	6.87743726454750	1.64582579970433	0.23671067782222
H	6.46386595175705	2.29902618315317	2.13485572749760
C	6.10635979285154	0.26288052594948	1.90152935663786
N	5.34312575682000	-0.27693496361580	2.78613917147155
C	5.62810000891112	-1.56762244949692	3.49338454649624

C	3.03275120392125	-0.55941713055717	0.68599714049694
O	4.05053550238260	-0.67215335178481	-0.02638482437738
O	2.99726004899177	-0.30977608251661	1.93051826183427
H	4.21572017258574	-0.13088260317557	2.52169144366693
C	4.64812031545966	2.31268424765572	1.02393389568782
C	4.22536280439184	2.47587804369799	-0.30025436085460
C	3.84307696286683	2.78400668874349	2.07020946109111
C	2.99256034532218	3.07569688759395	-0.56907019291259
H	4.86503570514632	2.13839093211425	-1.11594815120698
C	2.60534919351631	3.36843341528815	1.79846404039198
H	4.17838919543870	2.68495698005223	3.10641349771989
C	2.17397296435404	3.51165403129982	0.47671611019103
H	2.67072129969877	3.20678885810801	-1.60523133510709
H	1.97212841690995	3.71293864167576	2.61875057462144
H	1.20233921667825	3.96271872972918	0.26610650146417
C	8.82193525081341	0.34076320503570	-0.34252269716333
C	9.34222556016496	-0.65285202072082	-1.17980203919358
C	9.67852847417711	1.30904552427415	0.19545229345927
C	10.70507332883268	-0.68427207040858	-1.47543370760751
H	8.67268735394012	-1.40053929943388	-1.60566912732103
C	11.04211532099615	1.28599192478490	-0.09219734469972
H	9.27484019303815	2.09664768391170	0.83345374007507
C	11.54874855500565	0.28570579089257	-0.92764988616400
H	11.11655534339432	-1.45426983087990	-2.12952145927960
H	11.71365276336071	2.04007849762119	0.32057636060407
Cl	13.25315645537444	0.25252580906495	-1.29429072396142
C	7.00879781985373	-1.49111501170522	4.16358560836072
H	7.82277426288490	-1.43460659106108	3.42788253694349
H	7.07173288854853	-0.61719242672947	4.83102983547266

H	7.16771177039314	-2.39308810873839	4.77319549782116
C	4.53855543108848	-1.70064581266716	4.56810545715345
H	3.53612987476230	-1.72692088236286	4.11638942129517
H	4.68766281813071	-2.63054209523110	5.13637765360671
H	4.57723251966176	-0.85599803570232	5.27332189020169
C	5.55530387672364	-2.75298100777505	2.51529253523169
H	4.57079025724322	-2.80487058993125	2.03008128076959
H	6.32481093376377	-2.68083590708244	1.73497177051017
H	5.71357849441892	-3.69135981678069	3.06845518929716
C	1.64806982074978	-0.78363450757686	0.06690112940998
N	1.59738746735266	-0.58174245973804	-1.37913921858998
H	1.32784072036520	-1.81471092799761	0.29076474636710
H	0.91290855625253	-0.11127051963412	0.52700317119799
C	2.31022557776461	-1.25416437408892	-2.35481405383498
C	0.74296617055946	0.26325791016971	-1.96900527347671
C	1.86024052969023	-0.79135917132151	-3.55713729728183
H	3.07178466803446	-1.98401176044456	-2.10862464482563
N	0.88175965677361	0.15127843103857	-3.29307031066827
H	2.14509597696984	-1.05683785812361	-4.57059358116121
C	0.04633439375287	0.85055051512333	-4.26923358447859
H	0.12340651398705	0.32977965847594	-5.23131742590857
H	-0.99340176555891	0.81268128074485	-3.90918707189236
H	0.38508426619947	1.88995121940691	-4.38718707543509
H	-0.00136175477903	0.86904471330743	-1.42406824311349
O	-1.46908768508614	1.13765042902504	-0.28305395010987
P	-2.49219442715105	0.25475963112215	-0.95954424136310
O	-2.70102678937386	-1.03679900337846	0.08753537974266
O	-4.02255751895986	0.90434586458587	-0.91001664601583
O	-2.27105744990889	-0.20604292534392	-2.37516508410962

C	-3.73861384395443	-1.91173160250574	-0.08452661084595
C	-4.77102116614702	0.99406737351455	0.23204301704863
C	-5.03882435888673	-1.49841077539562	0.19480024746962
C	-3.42183048870465	-3.25011660719201	-0.47751094903330
C	-5.27959812519910	-0.16304839796287	0.81793982527064
C	-5.06727684339784	2.30409854967174	0.72538592371687
C	-6.12264029983580	-2.39312997202933	-0.11919375578143
C	-4.46718574185999	-4.13031330758149	-0.67095213003986
C	-1.99857917194266	-3.70798957807358	-0.61711208922151
C	-6.00912934214897	-0.04311053144743	2.05519649099213
C	-5.86146229051823	2.40552833889690	1.84940953393083
C	-4.54484573348338	3.53589715653488	0.04780216837679
C	-7.49138118036209	-2.00308509761207	-0.06046406630149
C	-5.82359533560134	-3.72845763381930	-0.54666209172539
H	-4.25088177590341	-5.16503445879052	-0.94879364648566
C	-1.20577463699177	-3.90759182897903	0.54124252727683
C	-1.45652157081215	-3.99760767841564	-1.89420546175407
C	-6.41131287380752	-1.16541298336789	2.83608890773371
C	-6.32015811481563	1.26351638784946	2.55637572134503
H	-6.12251078723761	3.39646884115928	2.22970023713730
C	-5.08523292077275	3.95121717394627	-1.19252481165800
C	-3.53539877320995	4.31324492883351	0.67067767179424
C	-8.50159754448599	-2.88698485975405	-0.37797452214235
H	-7.73911236584570	-0.98141201325140	0.22758818259090
C	-6.88889296388741	-4.62272982440812	-0.84686448853251
C	0.09781385845421	-4.40587708825490	0.40028844367858
C	-1.73021687646762	-3.63354179935030	1.95059887928174
C	-0.14757878152430	-4.49457736072060	-1.97719410709985
C	-2.25360644744659	-3.81446045907324	-3.18517364954383

C	-7.10908891490722	-1.00556603009798	4.01536306932482
H	-6.15042571746985	-2.16863162214568	2.49981724893198
C	-7.05851927406394	1.39478791555282	3.76558916058087
C	-4.61224522179274	5.13391649390337	-1.77684054773692
C	-6.19214952644395	3.17611085077938	-1.90434228462532
C	-3.10520211749969	5.49094884280414	0.04546542142268
C	-2.89137375373503	3.91679371251259	1.99991983064383
C	-8.20248413252972	-4.21599295155837	-0.76392623186105
H	-9.54243489852639	-2.55616473890869	-0.33572561627487
H	-6.64175291501135	-5.64100155972663	-1.16001218219648
C	0.64743813682950	-4.71784619981963	-0.84854847235215
H	0.68937064200570	-4.57689351448923	1.30291610513205
H	-2.77366613888548	-3.30143195911762	1.86257378582699
C	-0.95752683164198	-2.49766356989415	2.64199644026983
C	-1.74476953102169	-4.90824871047292	2.81216341942008
H	0.26649370672053	-4.72998643576485	-2.96214131536403
H	-3.19181220499473	-3.30478146090288	-2.92714795057991
C	-1.53296327902167	-2.90924123950485	-4.19688423489252
C	-2.61790710830916	-5.17186099968523	-3.81259716153588
C	-7.45153861886211	0.28615791098731	4.48251703053766
H	-7.39531899937602	-1.88577113379152	4.59675107125590
H	-7.29571156781462	2.40054366214202	4.12348194733561
C	-3.62711303341721	5.92339434468770	-1.17824935922370
H	-5.03349293249433	5.45748578476284	-2.73325447390124
H	-6.41963630897531	2.28337869714390	-1.30506095398430
C	-7.49096595013941	3.99770470248769	-1.98647542268839
C	-5.75066958285163	2.68153128751527	-3.29153471286023
H	-2.33036551762643	6.08799857593964	0.53386795579679
H	-3.28727824237234	2.93240678458613	2.28476498519237

C	-1.36761631876563	3.75304493333044	1.87557545218179
C	-3.25830396346418	4.90012763655496	3.12491260056579
H	-9.01161242317698	-4.90945649785935	-1.00623142131144
C	2.02256091821869	-5.36059790471684	-0.99724485007612
H	0.10417350310485	-2.75808959664804	2.79146636608357
H	-1.01182497836828	-1.57220279882737	2.05048408337184
H	-1.38754751414874	-2.28855559141355	3.63529197729772
H	-2.32615761378872	-5.71148745042391	2.33243206544681
H	-0.72753078068441	-5.29373889529774	2.99211653103118
H	-2.19771242182181	-4.70212570197171	3.79583866813796
H	-2.15729575668853	-2.77727122086147	-5.09587267794969
H	-1.36498862606209	-1.91737136208600	-3.75402506735382
H	-0.57048795831513	-3.33773196845611	-4.52386424530587
H	-1.71667416323496	-5.72809254001041	-4.12076284714449
H	-3.17462172258371	-5.80968592512856	-3.10809314068836
H	-3.24483366035886	-5.02955124498032	-4.70818932121609
H	-8.01186110729064	0.39998919095392	5.41391096436671
C	-3.15751857518200	7.21532349650331	-1.83494494533530
H	-7.82840954802153	4.31598815029446	-0.98721002482933
H	-7.35878669000968	4.90525471825897	-2.59830225762860
H	-8.29818277953532	3.40285283052540	-2.44525250389076
H	-6.54742231211778	2.07694183232476	-3.75582021031177
H	-5.53411955235617	3.52131630562323	-3.97287735084332
H	-4.85000507242990	2.05519767589035	-3.21342223727016
H	-0.94400543263371	3.40958870705095	2.83483544411480
H	-1.12668373266243	3.00501164829638	1.10581768240741
H	-0.87580834923352	4.70776370912672	1.62091833692833
H	-2.82857322508942	4.56970570127841	4.08529721976322
H	-2.87256560365821	5.91237295688113	2.91773406088803

H -4.34925880199058 4.98298530638905 3.25355513701250

H 2.35226392571780 -5.16563403977842 -2.03367240936610

C 3.09041777928183 -4.78149960138287 -0.05914890875411

C 1.92685653731622 -6.88865643756644 -0.83096024539975

H -3.67421745541438 7.28265741195345 -2.80844804148058

C -1.64701597517580 7.21200241870781 -2.11939586586131

C -3.56353774911585 8.45174165042881 -1.01514319172333

H 2.86428050013292 -4.99420249933400 0.99841588114666

H 4.07526822353592 -5.22684012091294 -0.27391306033463

H 3.18800747383884 -3.68946432172768 -0.16642420126067

H 1.59162526281381 -7.14982774631226 0.18637853095871

H 1.20510430993487 -7.32216687758067 -1.54001031633317

H 2.90548987439730 -7.36816401947474 -1.00006415133198

H -1.06202249490672 7.17263363792586 -1.18581974982934

H -1.35799119300574 6.34294647992299 -2.73115681998944

H -1.34757571033209 8.12610149939192 -2.65862299190829

H -3.27159056186533 9.38099604196348 -1.53241071496762

H -4.65169847711112 8.48027595306954 -0.84902111015732

H -3.07554842621531 8.45162507148547 -0.02647824913199

MS4S

C 6.65247584911701 1.21475433904985 0.64649463542909

O 7.85777970935428 -1.08400520841900 2.31919030651479

O 6.10977926848841 0.32142171229871 0.01225960174984

H 4.61671659274921 -0.18554999072774 -0.63973810891003

C 5.95482744658342 0.28381375649825 2.75134694951067

O 6.55127905879151 1.33581887089735 1.96842098681074

H 6.35919351204320 0.48009213775914 3.75591080194842

C 6.63568554393553 -1.07364075729479 2.36334320750084

N 5.83308477901416 -2.14322920458681 2.19785361329503

C	6.30206672905817	-3.51865618608506	1.89338345358522
C	2.95421950165103	-1.14569267218503	-0.47024798165072
O	3.72540085970792	-0.25406372210256	-1.07237629021677
O	3.30066047218372	-1.87831592407662	0.42813903528166
H	4.85434442326635	-1.95527443240495	1.98730695498299
C	4.44266859133426	0.38127481242565	2.83062445557475
C	3.68575921475205	1.23132621088257	2.01365788575483
C	3.77492135970720	-0.38193934858330	3.80416205612903
C	2.29333232848008	1.28215703505469	2.13348834914574
H	4.17629708267477	1.87156723332155	1.28026237711218
C	2.38514129081491	-0.33387541794348	3.92198079005577
H	4.34886034173687	-1.02558218038473	4.47610070364115
C	1.63606394400973	0.49217420906440	3.07902261740518
H	1.71141511037342	1.94274284303082	1.48760055028524
H	1.88516054462769	-0.94339953882659	4.67861621464668
H	0.54714156176414	0.52923635590496	3.15637110696065
C	7.47570565156758	2.28168114254045	0.02284684072122
C	7.61661252675725	2.28996771435119	-1.37402766770672
C	8.11819851182508	3.26808710075449	0.78877015158703
C	8.38279531563347	3.26753256251000	-2.00193916336966
H	7.12119227616243	1.51515838961341	-1.96134713128341
C	8.88608842901339	4.25109079430859	0.16926922967492
H	8.01705812271838	3.25767871435479	1.87414338989355
C	9.01407677163870	4.24506692852903	-1.22411209170710
H	8.49930561066512	3.27934991398064	-3.08644994471907
H	9.39009506760879	5.01983378430466	0.75649726490966
Cl	9.97574401577507	5.47012698882324	-2.00290593473486
C	7.30746076635774	-3.98718943178664	2.95801192788962
H	8.21503246696978	-3.37043239204612	2.95313714335151

H	6.85681757104297	-3.94229619633939	3.96225781719649
H	7.59161366503529	-5.03227474029669	2.75956648181035
C	5.05772392167790	-4.41809047389443	1.93761629303793
H	4.30055598450131	-4.08548184326140	1.21172432732229
H	5.33591600535295	-5.45535930118377	1.69898392705348
H	4.60129204892139	-4.40878329748397	2.94026426802153
C	6.93535456346351	-3.55938293105468	0.49090950225895
H	6.20389205126034	-3.25156273785652	-0.27223776043569
H	7.80331841454107	-2.88766809647343	0.43536577266661
H	7.27014627588373	-4.58184887694840	0.25405367240181
C	1.52145001757620	-1.16355846214502	-0.98200352585725
N	1.31173658818128	-0.52061035245826	-2.27316043250696
H	1.16824600924818	-2.20346292448556	-1.02997883825083
H	0.89129572938376	-0.64023365557194	-0.24345087524942
C	1.60887782009062	-1.04851471054808	-3.51711943023498
C	0.69074726784447	0.65068900319507	-2.43949949601413
C	1.14753394957002	-0.16076583728793	-4.44220125846458
H	2.11308770344184	-2.00185215250334	-3.63301453438958
N	0.58684770310617	0.89756318086036	-3.74684107249210
H	1.16335015169244	-0.19436111056625	-5.52728003460232
C	-0.23774170927222	1.96186317155975	-4.31044476589599
H	0.07034210018716	2.14592475702859	-5.34716833456319
H	-1.29258583386804	1.65353103715679	-4.26138350760343
H	-0.10171636791759	2.87931033059357	-3.72381144357049
H	0.26488409587116	1.23786205891264	-1.61744599112411
O	-0.99758368268447	1.01669570105158	-0.09360678404337
P	-2.10603945888476	0.27293653155790	-0.80677655612380
O	-2.31769669226463	-1.12337031133984	0.08822402621595
O	-3.59660788829769	0.97863960220220	-0.59958076576302

O	-1.98255128237105	-0.01661072422715	-2.27957330708023
C	-3.45497848754010	-1.87908642019530	-0.01553127585927
C	-4.19819235251037	1.06307570161796	0.62847026109230
C	-4.64985924922516	-1.39394419933535	0.50891168709330
C	-3.34034749803513	-3.17856419596159	-0.60201215567123
C	-4.67872228815337	-0.09167026093134	1.23890933457882
C	-4.38472022570932	2.36799310017134	1.18330983020186
C	-5.85174561490191	-2.16158032477088	0.30419277291788
C	-4.48344385173272	-3.94590285937906	-0.69879059831724
C	-2.01831608155894	-3.72220449009551	-1.06112804592485
C	-5.20592453027887	0.01111242434803	2.57528764107109
C	-4.98621001579495	2.45956292990107	2.42182724353727
C	-3.99709426956306	3.59541838079555	0.41212018540153
C	-7.15045319529915	-1.67656886441559	0.63323809853567
C	-5.75492631500470	-3.46053713969688	-0.29471852304401
H	-4.41804274570486	-4.95233451977512	-1.11992977544024
C	-1.06350207629767	-4.15571755159983	-0.10578659032158
C	-1.74334411139384	-3.86534592019317	-2.44301859489975
C	-5.54742667261752	-1.12469623139055	3.36412428389703
C	-5.36684069848655	1.30895413214018	3.16284648172904
H	-5.16227593322319	3.44571937422021	2.85873694046789
C	-4.76403352433598	3.98494800136474	-0.71289849633255
C	-2.89067856172082	4.38387035587676	0.81610788877789
C	-8.27726187094691	-2.44127203197121	0.41330524289258
H	-7.25395932482047	-0.67664293041711	1.05382257794717
C	-6.93393695345200	-4.23270796332781	-0.49031942708546
C	0.13196081927212	-4.73647578891184	-0.55070730197410
C	-1.31214472134853	-4.05302913126902	1.39871958169831
C	-0.53296505028614	-4.46059510150882	-2.83336200853749

C	-2.72996636572642	-3.43216864801987	-3.52710293431202
C	-6.04499813233819	-0.98445918815931	4.64320388482152
H	-5.39939887262377	-2.12222692688204	2.95008108203728
C	-5.89942590972661	1.42070573429566	4.47756228813380
C	-4.40908054909755	5.15039691148586	-1.40496056948285
C	-5.98918900124833	3.20140386365497	-1.18189318305100
C	-2.58471953492616	5.54384250686173	0.09168609617812
C	-2.02544462380627	4.02024358370445	2.02199133121251
C	-8.17200060373473	-3.73924122875971	-0.14223388446121
H	-9.26118912836906	-2.03823689093550	0.66631607142600
H	-6.83826732336483	-5.22570483059594	-0.93823331432433
C	0.41463953690315	-4.91409114493287	-1.91149011504105
H	0.85380352201458	-5.08237663736677	0.19392747772684
H	-2.30143537478152	-3.59820721890745	1.54502448349602
C	-0.29100133746909	-3.13690070321846	2.09313719335273
C	-1.36227671956658	-5.44209369651032	2.05915854156948
H	-0.33032981741749	-4.59354519105499	-3.90014924158387
H	-3.54151618273596	-2.87773832443941	-3.03606001826688
C	-2.10303672428552	-2.46874562204390	-4.54760534980396
C	-3.35723590491551	-4.65075028008079	-4.22771600089266
C	-6.23587247023967	0.30064241864031	5.20587149423762
H	-6.29018155329389	-1.87421384729603	5.22871240903329
H	-6.02525490969127	2.41923357482698	4.90518713938467
C	-3.32638098765607	5.94705786256983	-1.02386558808382
H	-5.00290834390132	5.45450970436901	-2.27207284130105
H	-6.08138185148451	2.30764055405385	-0.54992403233837
C	-7.28160095326999	4.01348146557818	-0.98752116325148
C	-5.84540894740965	2.70915874891465	-2.63099282889848
H	-1.73412639364178	6.15130068801859	0.41211379921918

H	-2.32466811409955	3.01640718677576	2.35447517702621
C	-0.53216538928265	3.93633493526707	1.66888416415059
C	-2.26354091276229	4.98604031667860	3.19632295349835
H	-9.07123733408122	-4.33847199475777	-0.30506195798194
C	1.68497064665077	-5.61642211301231	-2.37821031502267
H	0.73958235586532	-3.51462243736655	1.99077929354951
H	-0.32885662548742	-2.12086819700775	1.67372394201794
H	-0.51218938874285	-3.06391305389109	3.17080779695598
H	-2.11671038538375	-6.08676464695971	1.58116713325354
H	-0.39111193925885	-5.95983071083933	1.99544093301845
H	-1.61981134943356	-5.35260092303249	3.12731516957469
H	-2.86234477672460	-2.15628129086498	-5.28341740517639
H	-1.73275343248890	-1.56784618368297	-4.03753540389058
H	-1.27820885922950	-2.93974573015555	-5.10870884442327
H	-2.59616240117905	-5.24388176426571	-4.76210260626073
H	-3.85525259314701	-5.32206704907540	-3.51071958738246
H	-4.10902484757508	-4.32879615313484	-4.96703496753821
H	-6.63762624989393	0.39982009096961	6.21731078356317
C	-2.98669838167657	7.21716985909097	-1.79335582526224
H	-7.40822094532157	4.31815056510126	0.06346562065758
H	-7.28362989216032	4.92896132503800	-1.60208344170814
H	-8.16237937778453	3.41635774991961	-1.27652522416146
H	-6.71196696376874	2.08873156949546	-2.91328355878113
H	-5.79518750205334	3.54873624073252	-3.34424114598597
H	-4.93714490029915	2.09953064191687	-2.74781992359492
H	0.04785955062849	3.62167057771336	2.55236721173837
H	-0.37239755229664	3.19588833930675	0.87119269583120
H	-0.12691387020400	4.90933049835810	1.34481843457540
H	-1.67460679075434	4.68103532338535	4.07724319077634

H	-1.96628778001883	6.01551706856411	2.93514829174346
H	-3.32425363883420	5.01376632519429	3.49165179555005
H	1.65308384997042	-5.62906826671409	-3.48206152245423
C	2.96344290610526	-4.87006031120844	-1.96443732555862
C	1.72924998429665	-7.08020912639430	-1.90797466158380
H	-3.66888147748744	7.25487468071674	-2.66079372866494
C	-1.55151768901277	7.20371727527265	-2.34322900628448
C	-3.24702569515501	8.47904354468468	-0.95386507555240
H	3.05743926125593	-4.79738762898923	-0.86923250798144
H	3.86092193112151	-5.38548562910216	-2.34336072026573
H	2.97928861822867	-3.84243845574339	-2.36256112321424
H	1.78445432362884	-7.14653113126448	-0.80906054048425
H	0.83107654119665	-7.62930858826405	-2.23014016535215
H	2.61234933051361	-7.59759129116850	-2.31786504298906
H	-0.80699895818075	7.19358015034663	-1.53031557344109
H	-1.37324247002192	6.31575178584139	-2.97051281416954
H	-1.35841496932392	8.09962744012008	-2.95635174683283
H	-3.05968459831132	9.39151440977902	-1.54413555503219
H	-4.28805498806393	8.51114821070759	-0.59665807333603
H	-2.59062277271346	8.51255707186066	-0.06859434305113

TS4R

C	-6.76383033279434	0.98243137860740	-0.43340208564844
O	-6.65478391626129	1.62961539991348	0.94318115147434
O	-6.01894054904527	1.73215905512106	-1.28528008603846
H	-5.06805121424650	1.76227079995526	-1.02489653196635
C	-5.26015438681972	-0.22762676998512	0.83034316067841
O	-6.26323233795234	-0.28994282335526	-0.18486079511053
H	-4.25951070595371	-0.20164268453075	0.37842755170920
C	-5.56279603676862	1.12417969580738	1.48229041374211

N	-4.81323301982305	1.67592306388221	2.36219226723623
C	-5.10141793767233	2.96424232174316	3.05560979826636
C	-2.37874332603136	1.65773348175492	0.34615924814469
O	-3.32099239631715	1.83702772623527	-0.43866267870408
O	-2.46515084025883	1.40240979075655	1.60052323744613
H	-3.61006964483502	1.44799434017926	2.08962030601883
C	-5.35749778256365	-1.39764696057916	1.78404234893595
C	-6.58237023002626	-1.74903293998523	2.37108590971964
C	-4.20594132406008	-2.12710984216825	2.10263708200395
C	-6.65072216316692	-2.81894137911256	3.26382400358809
H	-7.48599233619606	-1.18595749667816	2.12574404467039
C	-4.27531193793627	-3.19663064114436	2.99999170428637
H	-3.24773007394519	-1.86147884667664	1.64848454147979
C	-5.49677601175662	-3.54423509459416	3.58053336298466
H	-7.60854349969605	-3.08935697124439	3.71504448295602
H	-3.37059915936819	-3.76132986260707	3.23695355144287
H	-5.55237846451778	-4.38271621925629	4.27906574280332
C	-8.21171737486998	0.96101703615753	-0.85068267250538
C	-8.77826936133051	2.09861081501697	-1.43969944423877
C	-9.00272237267012	-0.17375684777972	-0.63903001832466
C	-10.12348365766980	2.10933782258387	-1.80511332299351
H	-8.15794446051653	2.97772977318185	-1.61853238799839
C	-10.34846048544821	-0.17538956162301	-1.00814677533881
H	-8.55836586653884	-1.06742042789536	-0.20029498161608
C	-10.90267307084852	0.96918077577980	-1.58618419057073
H	-10.57099467916063	2.99247934846528	-2.26316056405620
H	-10.96771435887597	-1.06030027294109	-0.85498886443429
Cl	-12.58540309897652	0.97408805053766	-2.04756926290041
C	-6.49334796771480	2.91328917155795	3.70552626573934

H	-7.29450003078739	2.85822221721422	2.95579464423470
H	-6.57724663959181	2.04377048713263	4.37593328636825
H	-6.65188202478762	3.82175793539235	4.30595988722617
C	-4.02790830627069	3.10583864406338	4.14531385566250
H	-3.01786818661236	3.11741648270023	3.70904296455940
H	-4.17543249699073	4.04394596285183	4.70086880111253
H	-4.08194328115154	2.26871723948955	4.85803853931489
C	-5.00204944196705	4.13335146076782	2.05872817370692
H	-4.00577371080958	4.17085157236263	1.59339498012272
H	-5.75425066288280	4.04675774058875	1.26237777594597
H	-5.16699876706543	5.08522976803756	2.58654840403227
C	-0.93372066249579	1.72386132237552	-0.14761235525696
N	-0.82215164634262	1.82838237016741	-1.59746001590018
H	-0.42469199396083	2.58400077682855	0.31417409530499
H	-0.38615014852896	0.82042259188440	0.16581487298852
C	-1.06881624125186	2.93873324660012	-2.38245550200498
C	-0.38552424488156	0.83547612276384	-2.37846670570395
C	-0.75572396544856	2.59219779958678	-3.66390526199744
H	-1.43744219288663	3.86945653594384	-1.96599012908309
N	-0.33179075792109	1.27435421880162	-3.63798676420621
H	-0.78805875751189	3.16873655140489	-4.58317756855156
C	0.25054113540495	0.51203100031760	-4.74096575243319
H	0.35960165952641	1.17525406687496	-5.60734934902077
H	1.23708461564984	0.14804725166185	-4.41669892809293
H	-0.40228311059493	-0.33077716590227	-5.00845580608623
H	-0.05689017296765	-0.13526633940204	-1.99161809660725
O	1.05314758596707	-0.91419343408578	-0.53883875359093
P	2.38352337731841	-0.38823861599109	-1.02812844674083
O	2.95838809563008	0.54068402606569	0.24116653957939

O	3.55806991864446	-1.56299359623820	-1.07212918591944
O	2.46298858480533	0.34284185536697	-2.34214648203922
C	4.26510069203230	0.94292806038857	0.29072431375959
C	4.06592736116455	-2.13962384355158	0.06192407042997
C	5.26393080232839	0.00660820035542	0.54558753955444
C	4.53400905234433	2.34135127747959	0.15631205250661
C	4.89728025639095	-1.39747684841327	0.89703760716088
C	3.77447628963092	-3.52118702402252	0.28834271368921
C	6.63681288688397	0.43559076114510	0.47445357706467
C	5.85043325405048	2.75291405031505	0.19741584753265
C	3.41763704715426	3.33750018178445	0.03726192385182
C	5.36159679448162	-2.00492515479850	2.11888460446950
C	4.31548927242785	-4.11784019050962	1.40877981722982
C	2.93663989025954	-4.31616712381807	-0.66957144800365
C	7.73558693973348	-0.46950866907948	0.52581344414194
C	6.92438130830232	1.83058031965382	0.31038802164970
H	6.08218002358165	3.81842262026747	0.12188740723704
C	2.62846666908279	3.64643475411866	1.17399125776879
C	3.17450580703445	4.00602026926582	-1.18804066110220
C	6.06157003813355	-1.28639802509513	3.13106218740394
C	5.08063359181219	-3.39041375086187	2.35765140437621
H	4.13004721249177	-5.17971018340714	1.58894507034619
C	3.47892222865555	-4.72686579596404	-1.91035256467520
C	1.61843931278220	-4.69941992375263	-0.31426475297257
C	9.03729696275207	-0.01919586358998	0.45071112086303
H	7.53877204460889	-1.53816912259522	0.61215546103636
C	8.27810685018371	2.26533581937126	0.25630996317886
C	1.62509558060078	4.61982547915014	1.06491168130821
C	2.85659371233433	2.98452446657031	2.53282707295483

C	2.15949246247617	4.97296282206304	-1.24056568588572
C	3.99601283518226	3.73231271395665	-2.44769053107084
C	6.48905760687388	-1.90787462752828	4.28617609245546
H	6.25199783920491	-0.22229778881793	2.99380622927563
C	5.55340230434284	-4.00756643896115	3.54944008420910
C	2.70118066543296	-5.52848170767267	-2.75712046007208
C	4.89132471432822	-4.34673129899945	-2.35046519591376
C	0.88774427494507	-5.50590385897877	-1.19630547932703
C	0.97001232459973	-4.27196959323195	1.00340358041462
C	9.31687271730313	1.36336711057581	0.32761769384336
H	9.86053244236240	-0.73746227338341	0.48212582693281
H	8.47805792882522	3.33456320864684	0.14365779452451
C	1.37496127186220	5.30308936833154	-0.13089249730484
H	1.03769237427406	4.86323424640024	1.95380391130413
H	3.67898632646895	2.26465261848139	2.42321616791061
C	1.62853956428561	2.18821085891505	3.00438881757980
C	3.30530558781945	4.00667194660706	3.59184076614619
H	1.97926268300191	5.49864626913942	-2.18302460239211
H	4.63210697952231	2.85998730167437	-2.24472748393729
C	3.12319957209285	3.35863846938660	-3.65621215052439
C	4.92204902056569	4.91647929902061	-2.77722786398891
C	6.24755062545722	-3.28685845269402	4.49599640139907
H	7.01592404066776	-1.32802145196969	5.04831068961301
H	5.34076349936784	-5.06881606625630	3.70538238025614
C	1.40796602165119	-5.93688301331649	-2.42176355979381
H	3.12128437715382	-5.85377255495472	-3.71356084187468
H	5.33451938455626	-3.72632627289299	-1.55903749226616
C	5.79377321558338	-5.58355725090998	-2.49965723592176
C	4.88439806318574	-3.49665690613868	-3.63212164159610

H	-0.12555978270487	-5.80393280282807	-0.91433551589366
H	1.62080987311486	-3.51443088553578	1.46194338309023
C	-0.39810573200649	-3.60137166731766	0.79624131052070
C	0.86331588389736	-5.45040851306712	1.98729266696196
H	10.35244462211810	1.70889217734067	0.27740662246836
C	0.34710480853594	6.42562154716079	-0.22484945305468
H	0.75091672375474	2.83855373525474	3.15745269039442
H	1.36209950297175	1.41081099613921	2.27351300797002
H	1.83871669504606	1.68918468250317	3.96448716852692
H	4.20807472851990	4.54874656444038	3.26874933459133
H	2.52175974034758	4.75488537190507	3.79734622899622
H	3.53618142291708	3.49946257846931	4.54306704541899
H	3.76257458197981	3.13658673330423	-4.52646372867179
H	2.53549387384850	2.45752018656135	-3.42945118523317
H	2.44516188694772	4.17840310164198	-3.94783906914904
H	4.34447727429732	5.82567270958390	-3.01492893885253
H	5.58897177780811	5.15888495024901	-1.93492648982675
H	5.55260235317900	4.68365962654144	-3.65104642684483
H	6.59935347699239	-3.77049000264730	5.41067775078146
C	0.60264946909657	-6.82356774073958	-3.36291113364570
H	5.82830059119479	-6.17007064151526	-1.56780312202967
H	5.44063230491680	-6.25271016327899	-3.30171408005981
H	6.82460486701591	-5.28279077281146	-2.75013429217648
H	5.90958078174434	-3.18620263295936	-3.89403160869160
H	4.47916665617751	-4.05889521652884	-4.48998440063396
H	4.27850111881221	-2.58915804322251	-3.49456913957309
H	-0.78546124024559	-3.23692569060612	1.76363026283753
H	-0.29852574646252	-2.73827066533979	0.12313648036594
H	-1.14470777057864	-4.30116165795290	0.38479406898170

H	0.44043642736310	-5.11726030076552	2.94998622428403
H	0.20876132910813	-6.24439077214926	1.59001837269051
H	1.84594744573133	-5.90404303872192	2.19028672899722
H	0.12279731906108	6.56092996682233	-1.29836228269981
C	-0.97826240515762	6.11704793500884	0.48645423115658
C	0.93865364760267	7.75275662583973	0.28472995641859
H	1.23374348796280	-6.99788940924464	-4.25204344845746
C	-0.68625024334363	-6.13854488113011	-3.84569268273349
C	0.30026900812345	-8.19689279330263	-2.74077065739481
H	-0.84708745926619	6.03302164489723	1.57734415909457
H	-1.71049206322351	6.92118593978367	0.30884698903556
H	-1.42432595624098	5.17232048927998	0.13439263932893
H	1.18983502304978	7.68321547743713	1.35606556864309
H	1.86173886563291	8.01033299931343	-0.25681191154791
H	0.22251007569607	8.58159964114310	0.15687740974624
H	-1.37945668924011	-5.95033707552281	-3.00923483895226
H	-0.46871482588223	-5.16959894328994	-4.32186121885272
H	-1.21503622755848	-6.76992843672762	-4.57906508440774
H	-0.22782038256989	-8.84647332446532	-3.45863665872041
H	1.22578178689792	-8.70831241501940	-2.43367738878673
H	-0.33864665821931	-8.09868291153548	-1.84768522097213

MS4R

C	6.57462516569902	-0.89555007756519	-0.49230301700376
O	7.59472428758919	-1.74702018273859	2.47698578100957
O	5.65682572432314	-1.70800471082786	-0.47440344198490
H	4.03248160065710	-2.00168669007527	-0.33202285358113
C	6.00119510983310	-0.11664690453785	1.68795897033978
O	6.80174053480788	-0.03325512108671	0.48907575820988
H	4.94327802282706	-0.19269900683236	1.40433247441194

C	6.42861834137540	-1.38034696801744	2.48055958057092
N	5.42118962917960	-1.94485096629124	3.18169964480878
C	5.55064905575725	-3.10156411865822	4.09838901878770
C	2.35437944883051	-1.98971850137690	0.64011537668954
O	3.06354128376618	-2.21261305539410	-0.44915065633817
O	2.79344889355872	-1.59240371393747	1.69944959341688
H	4.47587814884891	-1.67029462335851	2.91970710050102
C	6.22980685154602	1.14722017539677	2.48560905598419
C	7.51466588642241	1.48835829047447	2.93704916707676
C	5.14951947469938	1.98686850160898	2.78597193843772
C	7.70917271012605	2.65737192953629	3.67388937707420
H	8.35486303752475	0.82800237486001	2.71253466618639
C	5.34602656628522	3.15401792194232	3.52968941651014
H	4.14846511128636	1.73204855354681	2.43040803818947
C	6.62633468475650	3.49147763877005	3.97335579698686
H	8.71222986151172	2.91836861735065	4.02051304350459
H	4.49489193048744	3.80165237735928	3.75411793654679
H	6.78244959813914	4.40515526379528	4.55244911863991
C	7.52847160848022	-0.76955712908988	-1.62251203240948
C	7.36730051345562	-1.60713306812831	-2.73735773174924
C	8.57725631357451	0.16417959512130	-1.60292376162856
C	8.23589975971160	-1.51631257303015	-3.82103070343671
H	6.55175217114645	-2.33208566132901	-2.74168677053924
C	9.45081218831939	0.26253169254975	-2.68298299990606
H	8.70461015556786	0.81211936661406	-0.73547709474773
C	9.27440172485210	-0.57857862002678	-3.78721662276797
H	8.11832914565855	-2.16362124986720	-4.69108126486459
H	10.26835784261673	0.98459711045576	-2.67680266414961
Cl	10.36419238974192	-0.45854408506882	-5.14007955764566

C	6.57343190527263	-2.78705054224517	5.20283275620342
H	7.57088749691852	-2.61100940058044	4.77894080608757
H	6.27018626675583	-1.89262442016953	5.76923907042735
H	6.63580733033787	-3.63362158285181	5.90403915183618
C	4.16461569646026	-3.31506127214810	4.72289438578783
H	3.40668841986563	-3.52828607500692	3.95206444362336
H	4.19079839709070	-4.16614658613627	5.41934870427752
H	3.84161331899263	-2.42363584014020	5.28350148777901
C	5.97385450923694	-4.35374663704195	3.30858009297772
H	5.23194463386211	-4.58945405130290	2.52901173630767
H	6.94860626900721	-4.19752457112068	2.82629157149044
H	6.05213926775882	-5.22208797346010	3.98174866272495
C	0.86647951962024	-2.26401763649018	0.47605463248933
N	0.48640044029439	-2.84254331696803	-0.80804063784434
H	0.53802068259752	-2.92771967517198	1.28822792764940
H	0.31179440295123	-1.31096176632097	0.57816928430529
C	0.56304370681010	-4.16833002317686	-1.19394921096010
C	-0.02574804609135	-2.13005345907694	-1.81824574798820
C	0.06954623329810	-4.23901252446729	-2.46437990650978
H	0.94849499308776	-4.94214838893894	-0.53713715975450
N	-0.29574411713198	-2.95505172802882	-2.83083235005689
H	-0.06650131683767	-5.08924736242742	-3.12596488575514
C	-0.93114851186043	-2.53846647311409	-4.08175188070581
H	-1.63555093238932	-3.31798838153172	-4.39794142090642
H	-1.48887299419765	-1.61300133632414	-3.87881633984514
H	-0.16972116787780	-2.38249113795281	-4.85952453748392
H	-0.24029336422118	-1.06139303422627	-1.75878394278925
O	-0.95139282393863	0.13430797616821	-0.25004220807758
P	-2.32874165731167	0.17534537137720	-0.87791258252002

O	-3.33812945601056	-0.49804376273651	0.27051289674423
O	-2.94890943994040	1.71434319488401	-0.92615218121777
O	-2.54010966771074	-0.43055802846499	-2.23953560567466
C	-4.68677323170532	-0.26519062740186	0.29893520274111
C	-3.15722068886488	2.42530411716080	0.22925409593473
C	-5.16074269798753	0.99285215922039	0.66460317842494
C	-5.55566136113076	-1.36734228717111	0.02308665520426
C	-4.20805196666618	2.06817280947382	1.07005367531278
C	-2.31748617459344	3.55682399494130	0.47417662598263
C	-6.58106835500662	1.23175425654016	0.62001839088188
C	-6.91698808026463	-1.15005947458736	0.09826027830413
C	-5.02680464096208	-2.72716241748270	-0.32744399629238
C	-4.34430644097361	2.75875921854997	2.32687096896135
C	-2.53569623104225	4.27753963671227	1.63111252671151
C	-1.26022267246658	3.97541516489287	-0.50471234666585
C	-7.16021746702058	2.52249544368827	0.79139144536875
C	-7.46405769641781	0.13304559220870	0.35910386221360
H	-7.60073350447716	-1.98359008373022	-0.08132431525720
C	-4.41178357391993	-3.53453644410815	0.66217980075868
C	-5.19619767675340	-3.23545861182578	-1.63968060356566
C	-5.25577647444725	2.35156474017938	3.34312312428291
C	-3.50443271267736	3.88908187464032	2.59378379114583
H	-1.92730213729691	5.16319236233956	1.83025783476907
C	-1.63193515715480	4.55078738007901	-1.74395672578037
C	0.11321536177501	3.83473039440787	-0.17937680405650
C	-8.52664254777242	2.70608423393481	0.74737472840423
H	-6.50812895314065	3.38139320327391	0.94811720164338
C	-8.87024794742356	0.35124150073050	0.33970807063641
C	-4.00542144812160	-4.83336336255571	0.32796318724968

C	-4.21033348993737	-3.05414786949476	2.09878086312781
C	-4.77085087324454	-4.54271379248923	-1.91902344620710
C	-5.83349164952977	-2.42200993094414	-2.76665983310526
C	-5.35405178266697	3.04036749667532	4.53429082989056
H	-5.87668640828847	1.47107660144169	3.17809962038544
C	-3.64434907202760	4.59094910427466	3.82380466339538
C	-0.62669573371161	4.97813076070706	-2.62195702641402
C	-3.09068864976111	4.75564577465537	-2.14952100132047
C	1.07567991366900	4.28757171741528	-1.09089526417318
C	0.58656471455953	3.19884263285207	1.12797140904693
C	-9.39624225614009	1.60928913859665	0.53370090583877
H	-8.94098457244584	3.70951497469900	0.87357727746055
H	-9.52721064750788	-0.50253796022136	0.15176571227105
C	-4.18307951506944	-5.36552290652783	-0.95436637205377
H	-3.55368254285659	-5.45458668330622	1.10577575750273
H	-4.59381560119404	-2.02717013768940	2.16898253723152
C	-2.72241992827235	-2.99749738741116	2.48372087184796
C	-5.01701736134569	-3.89840993027295	3.10042389527349
H	-4.91501007664899	-4.94058609684059	-2.92814306942065
H	-6.03486314983382	-1.41455923616513	-2.37863336559206
C	-4.88444646489721	-2.24350516864042	-3.96287759595885
C	-7.18110116352914	-3.02100522796676	-3.20578024790590
C	-4.55113127639128	4.18108796000846	4.77638009265729
H	-6.05577020213563	2.69926935978928	5.29970296882432
H	-3.00345133604201	5.45841119672212	4.00415360800909
C	0.73208089819315	4.86065243869974	-2.31981024678754
H	-0.91284146404913	5.42441309703718	-3.57886334498862
H	-3.72468506590056	4.32483880738940	-1.36248405597983
C	-3.44570968664587	6.25061491189228	-2.23415625360708

C	-3.43904198170516	4.02005226203657	-3.45365103734141
H	2.13256226299846	4.17996658176752	-0.83273710948318
H	-0.29132657391937	2.76009277221688	1.62155106442087
C	1.57345434307694	2.04473315745576	0.88813760754410
C	1.18534779102650	4.24502395122077	2.08503558495124
H	-10.47746041831083	1.76610950371561	0.50880181021486
C	-3.82228098157769	-6.80797676693710	-1.28922594170285
H	-2.25793966036784	-3.99826271083415	2.45887931943515
H	-2.17433916286728	-2.33306452720014	1.79935892725145
H	-2.60222386615789	-2.60273062710445	3.50602212188889
H	-6.08786951370216	-3.90857521442873	2.84321129876466
H	-4.67010598142598	-4.94465329935166	3.12624111886561
H	-4.91613308398904	-3.49013967175065	4.11951724232661
H	-5.36236714653527	-1.62033172793684	-4.73693562066964
H	-3.96220313675048	-1.73873398022192	-3.64101935669796
H	-4.62919085772111	-3.20941472842082	-4.43224061864222
H	-7.05558228591102	-4.02722378556130	-3.63944286923088
H	-7.88080049964568	-3.11065842111824	-2.35971542181837
H	-7.65529086398112	-2.38565928591088	-3.97181905807733
H	-4.64354328535402	4.72399739195302	5.72032023929859
C	1.79422928626920	5.34023444853099	-3.30053636063979
H	-3.24429410001899	6.76549882611023	-1.28122807522978
H	-2.86456891259839	6.76221716366448	-3.01911042655443
H	-4.51432968193207	6.38241708208591	-2.47205364731934
H	-4.51243435299941	4.13012659676139	-3.68067014497035
H	-2.87786740539603	4.42395046683568	-4.31265325813977
H	-3.21664582608770	2.94625566727104	-3.36873817240173
H	1.80304229515653	1.53812285647742	1.84074389274077
H	1.13003066140150	1.30341707075171	0.20878437983428

H	2.52580038440200	2.39877375455834	0.45936027886441
H	1.47356926749405	3.77852779959689	3.04235714895133
H	2.08674619941561	4.71256046402849	1.65392125656814
H	0.46949482881516	5.05177593157821	2.30657206908952
H	-3.82551632165212	-6.88746059321861	-2.39103307070784
C	-2.42399120383388	-7.21724441994096	-0.80491118822075
C	-4.89129316591122	-7.78144280735659	-0.76074679381648
H	1.25894591816061	5.70961955850014	-4.19279272313125
C	2.71451171177318	4.19766980435385	-3.76028915358698
C	2.60878797402183	6.51632597961033	-2.73718790217405
H	-2.35977219384482	-7.22201431680450	0.29540313499613
H	-2.17428151911227	-8.23404314107407	-1.14954450413254
H	-1.65196617286531	-6.52694957805739	-1.18052271979629
H	-4.94462679386693	-7.74728982555888	0.33998360767676
H	-5.88881838364034	-7.52521765304670	-1.14959539849317
H	-4.66379731934412	-8.81918934407483	-1.05671199717482
H	3.30070742077297	3.79094076178044	-2.91972441039151
H	2.13384806325339	3.36827861020186	-4.19317597452908
H	3.42870595576599	4.55082014664008	-4.52266645076065
H	3.33108073931633	6.88749174304880	-3.48326532781831
H	1.95386961004740	7.35365643616937	-2.44993390223709
H	3.17896209235957	6.21635928103961	-1.84243990410797

(1) Zhang, J.; Lin, S.-X.; Cheng, D.-J.; Liu, X.-Y.; Tan, B. *J. Am. Chem. Soc.*, **2015**, *137*, 14039–14042.
